# A Mechanical *sp* ^2^‐*Jail* for C_60_ Fullerene

**DOI:** 10.1002/anie.7486858

**Published:** 2026-09-11

**Authors:** A. Priscila Gia, Fernando G. Guijarro, Pau Armada, Daniel Aranda, Josefina Perles, Enrique Ortí, Juan Aragó, David González‐Rodríguez, Alberto de Juan

**Affiliations:** ^1^ Nanostructured Molecular Systems and Materials group Organic Chemistry Department Universidad Autónoma de Madrid Madrid Spain; ^2^ Institute of Molecular Science Universitat De València Paterna Spain; ^3^ Andalucía Tech Science Faculty Department of Physical Chemistry Universidad de Málaga Málaga Spain; ^4^ Single Crystal X‐ray Diffraction Laboratory SIdI Universidad Autónoma de Madrid Madrid Spain; ^5^ Institute for Advanced Research in Chemical Sciences (IAdChem) Universidad Autónoma de Madrid Madrid Spain

**Keywords:** fullerenes, host–guest chemistry, molecular recognition, porphyrin nanocages, self‐assembly

## Abstract

Being an iconic spherical molecule exclusively composed of *sp* ^2^ carbon atoms, the development of increasingly efficient synthetic receptors for C_60_ fullerene remains a pressing challenge in supramolecular chemistry. However, as tighter hosts that employ most of their cavity volumes to complementarily encapsulate C_60_ are developed, steric constraints may emerge that mechanically trap this ball‐shaped guest within their lumen. Here we explore this unprecedented scenario with a rigid bisporphyrin *sp^2^*‐receptor that acts as a “mechanical jail” for C_60_. This exceptional host presents the tightest 3D cavity reported for this spherical guest, occupying 77% of the internal volume and enabling strong π–π and CH⋯π interactions, which result in exceptionally high binding affinities (*K_a_* > 10^9^ M^−^^1^). Unlike related *cage* and *basket* analogues, encapsulation in this perfectly fitted host is kinetically hindered, leading to guest release dynamics that are extremely slow above 400 K and essentially suppressed at room temperature.

## Graphical abstract

A rigid bisporphyrin receptor with an exceptionally compact 3D cavity functions as a molecular “mechanical jail”, tightly enclosing C_60_ fullerene. Near‐perfect shape complementarity affords ultrahigh binding affinity while imposing a large kinetic barrier for guest exchange, rendering encapsulation effectively irreversible under ambient conditions.

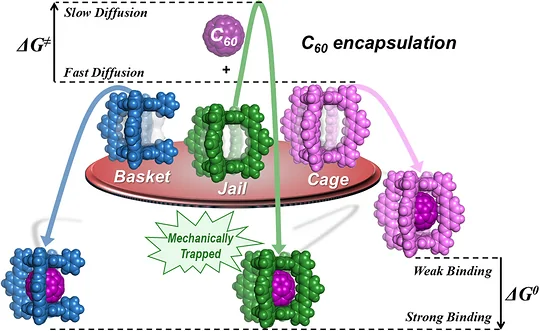

## 1 Introduction

The development of increasingly efficient synthetic receptors demands the optimization of recognition host cavities so that they match the corresponding guest shape tightly. Among different guest families, fullerenes, and in particular **C_60_**, are arguably the most iconic and widely studied. This is, on one hand, because of the diverse applications of these electroactive carbon allotropes in fields that span biomedicine [1, 2] and materials science [3, 4, 5]. However, inherently, the interest in **C**_**60**_ as a guest lies in its singular structure: it is an *I*_h_‐symmetric, nearly spherical molecule, possesses a relatively large surface area, is highly rigid and lacks conformational freedom, and is solely made of *sp* ^2^ carbon atoms, and thus devoid of dipolar groups.

From the vast number of examples found in the literature and collected in excellent reviews [6, 7, 8, 9, 10, 11, 12, 13, 14], it is generally agreed that, in order to create optimal **C_60_** hosts, several ingredients are required. Typically, fullerene receptors tend to maximize dispersion forces and thus employ large aromatic groups to establish stabilizing π–π stacking interactions. Since fullerenes are π‐electron‐poor entities, electron‐rich aromatics like acenes, porphyrins, or tetrathiafulvalenes (TTFs) are preferred to induce donor–acceptor interactions. Some authors have also invoked specific coordination interactions between metal species and the fullerene double bonds, as in Rh^III^ or Ir^II^ porphyrins, to significantly enhance binding strengths [15]. In addition, chalcogen⋯π [16] and CH⋯π [17, 18] interactions are known to stabilize host:**C_60_** complexes in solution and in the solid state, provided these hosts are able to surround the fullerene surface efficiently and establish a large number of these weak interactions. As a step further, designing multivalent receptors that include several of these interacting units, like in tweezer, cavitand, or macrocyclic structures, [8, 11, 12] introduces chelate cooperative effects that can significantly enhance binding affinities [19]. Preorganized receptors having rigid linkers connecting these units are preferred in order to maximize cooperativity. However, any deviation from a perfect fit in these rigid complexes may introduce strain, a destabilizing situation that, on the contrary, the use of flexible linkers can alleviate [11].

Having a globular silhouette, the concept of shape‐complementarity has probably been exploited for **C_60_** more than for any other guest molecule [7]. Indeed, receptors based on concave π‐conjugated molecules, such as cyclotriveratrylenes, calixarenes, subphthalocyanines, extended TTFs, corannulenes, and buckybowls in general, can supply larger π–π contact areas and thus higher association constants (typically, *K*_a_ > 10^2^ M^−1^) with **C_60_** [13, 20, 21]. More recently, this concept was developed further to arrive at cyclophane‐type “nanohoop” receptors, which are endowed with cyclic curved aromatic surfaces that function as molecular belts for fullerenes, resulting in extremely high binding affinities (often, *K*_a_ > 10^5^ M^−1^) [12]. With the aim of surrounding these ball‐shaped molecules even more efficiently and offering them a solvation sphere in multiple directions, barrel [22] and cage [10] assemblies emerge as the ideal receptors. Recent computational simulations that utilize an evolutionary algorithm to identify the best‐suited *cage* hosts for **C_60_** suggest that a matching cavity size, a high symmetry, and a rigid host structure are key general elements to achieve strong binding interactions [23]. Thus, with the idea in mind to wrap as much as possible the round **C_60_** π‐surface, several host:**C_60_** systems have reached thermodynamic stabilities that can even exceed *K*_a_ > 10^9^ M^−1^, a limit above which detection of binding interactions by even the most sensitive techniques starts to be less reliable [24].

However, as tighter receptors are developed that employ most of their cavity volume to complementarily encapsulate **C_60_**, kinetic effects are likely to arise as a result of steric constraints, which would mechanically impede free diffusion of this round guest in and out through the receptor's windows. This is an unexplored scenario that we analyze in this work with a highly complementary bisporphyrin host (compound **1** **_Zn_** in Figure 1) that, with the exception of the peripheral solubilizing groups, is only composed of *sp* ^2^ C and N atoms. The pseudo‐spherical cavity of this *sp* ^2^‐receptor can be filled by *ca*. 77% upon encapsulation of **C_60_**, which is, to our knowledge, the tightest 3D match ever reported, establishing both π–π and CH⋯π interactions with the fullerene surface. As a consequence, this host not only exhibits record association strengths with **C_60_**, enrolling the short list of fullerene receptors where *K*_a_ > 10^9^ M^−1^ [23], but also affords a unique kinetic trap for this guest and, once encapsulated, **C_60_** cannot escape freely at room temperature, being mechanically locked inside **1_Zn_**.

**FIGURE 1:**
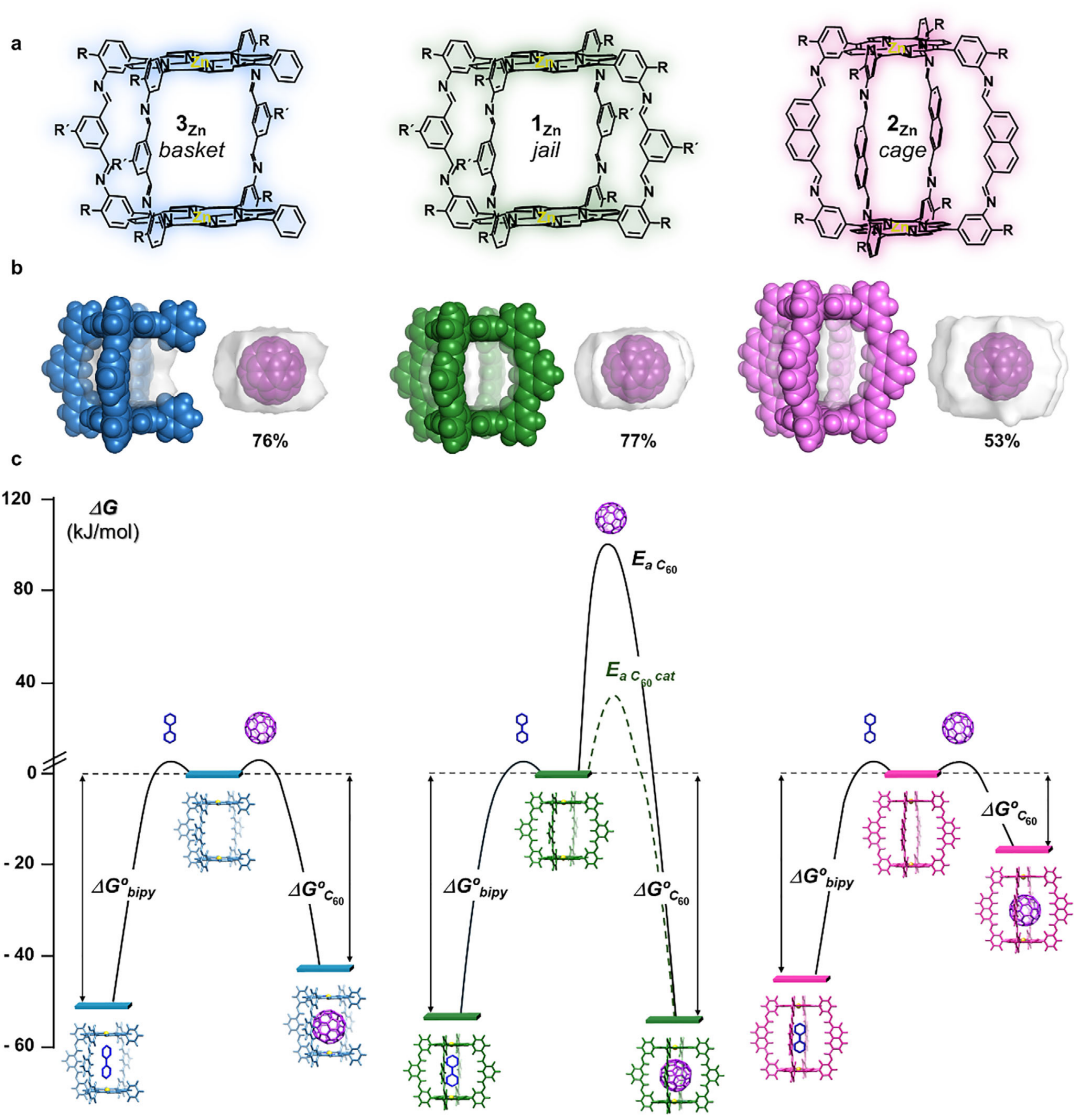
(a) Chemical structure (R = OC_6_H_13;_ R′ = OC_12_H_25_) and (b) computational models of *basket* **3_Zn_**, *jail* **1_Zn_**, and *cage* **2_Zn_** showing (on the right) the void inner cavities filled with fullerene **C_60_** and the % free‐space occupancy. (c) Energy landscape, including experimentally determined Gibbs free energies (Δ*G°*) and activation energies (*E_a_*), associated with the encapsulation of **C_60_** and ***bipy*** into hosts **3_Zn_**, **1_Zn_**, and **2_Zn_**.

## 2 Results and Discussion

The synthesis of the rigid bisporphyrin receptor **1_Zn_** is extraordinarily simple and efficient, and can be scaled up to gram amounts, as described in our previous work [25]. It involves the dynamic condensation of a tetraaminoporphyrin and a 1,3‐diformylbenzene linker, followed by metallation with Zn(OAc)_2_. Compounds **2_Zn_**, endowed with a larger internal cavity, and **3_Zn_**, where one of the four linkers in **1_Zn_** has been formally removed, can be produced in a similar way from a 2,7‐diformylnaphthalene analogue or from an unsymmetrically substituted A_3_B triaminoporphyrin derivative, respectively. More details about the synthesis and characterization of novel compounds **2_Zn_** and **3_Zn_** are found in the Supporting Information. Please note that, from here on and in this particular manuscript, we will use the terms *jail* for **1_Zn_**, *cage* for **2_Zn_**, and *basket* for **3_Zn_** to distinguish the structure, on one hand, and the very different **C_60_** association/dissociation kinetics, on the other, displayed by these three rigid cofacial bisporphyrin receptors, as described below.

In a first attempt to complex **C_60_** within the **1_Zn_** cavity, both the bisporphyrin and the fullerene were mixed in a 1:1 ratio in toluene‐D_8_ (Figure 2a). The ^1^H NMR spectra of **1_Zn_** disclosed absolutely no change even after 24 h at 298 K (Figure S24). Only after 185 h did a new set of signals become barely perceptible over the baseline. The same mixture was then heated to 373 K and, after *ca*. 48 h, two sets of signals were observed in a 1:1 ratio: the original **1_Zn_** signals and a new growing set attributed to a different *jail* species (Figures 2b, S24, and S25). Prolonged heating over >144‒480 h (6‒20 days, depending on concentration) resulted in the full transformation of **1_Zn_** into this new species, which disclosed the same *D*_4h_ symmetry as the precursor *jail*. Over the same period of time at 373 K, the original ^13^C signal of **C_60_** at 143.4 ppm progressively disappeared at the expense of a new signal at 140.5 ppm (Figures 2c and S26), which was assigned to encapsulated **C_60_**. MALDI‐TOF mass spectrometry analysis of aliquots taken at different time intervals exhibited a new prominent peak at *m/z* = 4127.8, which confirmed the inclusion of **C_60_** into the **1_Zn_** receptor (Figures 2d and S27). **C_60_** complexation also produced relevant electronic perturbations in the ground and excited states of **1_Zn_**. The porphyrin Soret band was found to undergo a red shift from 427 to 437 nm, while emission intensity was totally quenched when **C_60_** was included in the cavity (Figure 2e). This is likely due to photoinduced electron transfer from the macrocycle to the **C**_**60**_ guest [26, 27, 28], as supported by the pronounced charge‐transfer character of the HOMO→LUMO excitation revealed by theoretical calculations (see Figure S29). However, the ground‐state redox features of **C_60_** and **1_Zn_** were not significantly perturbed when assembled together [29], as inferred from voltammetry measurements (Figure S28 and Table S1) and theoretical calculations (Figure S29). The product **C_60_** **@** **1_Zn_** could be isolated in pure form by precipitation upon addition of methanol. Suitable single crystals for xX‐ray diffraction were obtained from **C_60_** @**1_Zn_** analogues by slow cooling in toluene/DMSO mixtures, confirming the structure of the proposed inclusion complex (see Figures 3a, S36–S38, and Table S5 for further details).

**FIGURE 2:**
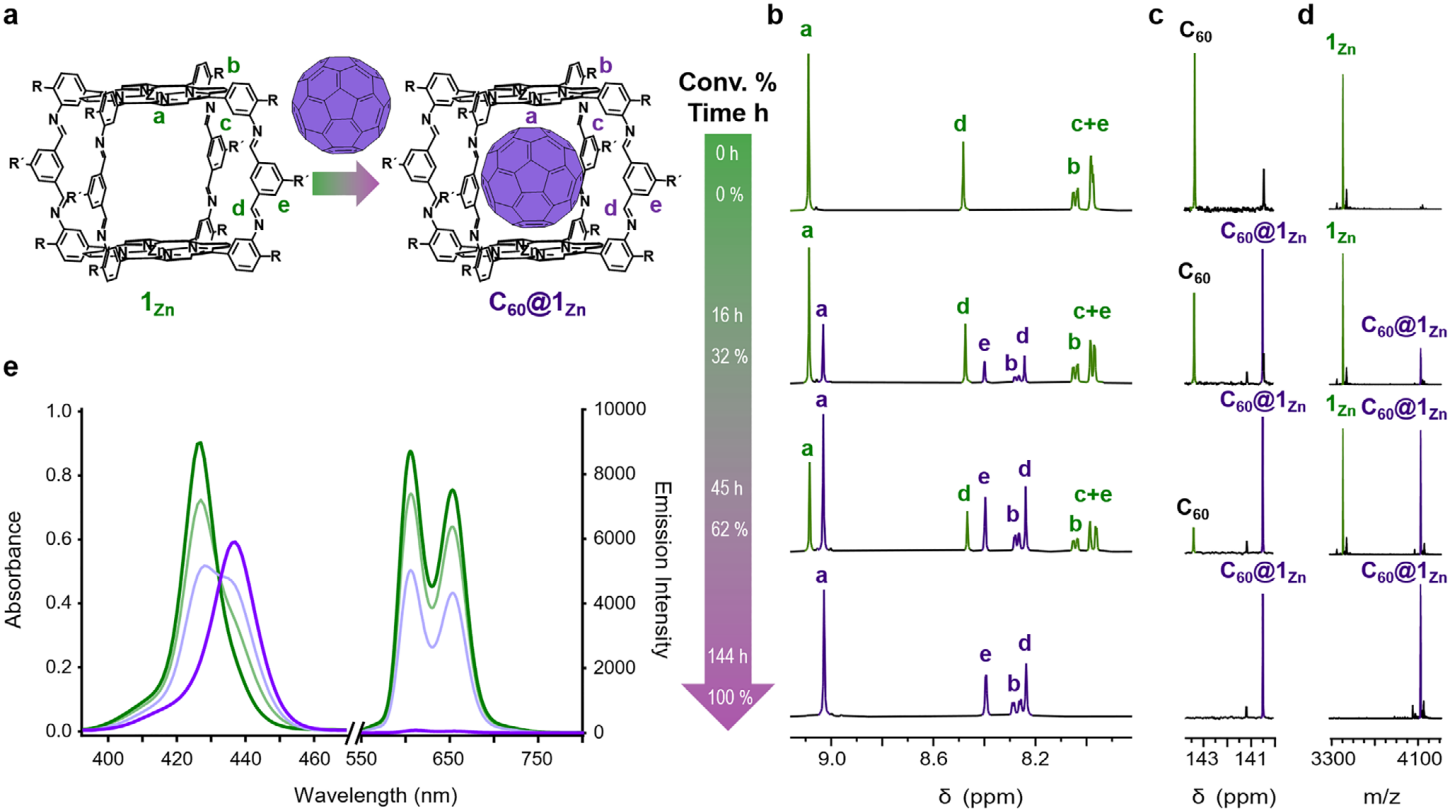
(a) **C_60_** fullerene encapsulation into **1_Zn_** in toluene‐D_8_ at 373 K monitored as a function of time by (b) ^1^H NMR, (c) ^13^C NMR, and (d) MALDI‐TOF MS, as well as by (e) absorption and emission (*λ*_exc_ = 427 nm) spectroscopies (see also Figure S24).

**FIGURE 3:**
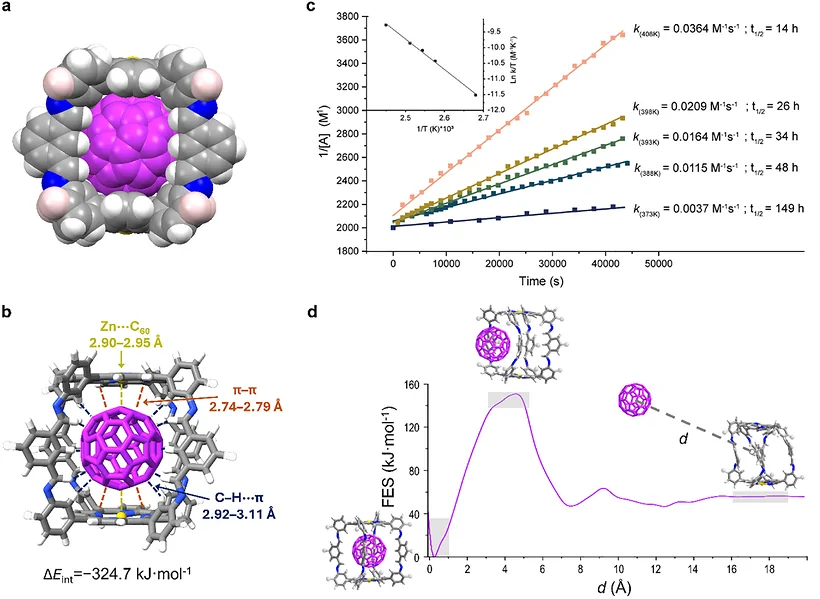
(a) Solid state x‐ray structure of **C_60_@1_Zn_** in space‐filling (van der Waals) style (see Supporting Information for further details). Note that the solubilizing alkyl chains have been omitted for clarity. (b) Representation of the DFT‐optimized structure of **C_60_@1_Zn_** highlighting distances associated with noncovalent forces and the interaction energy (Δ*E*_int_) between **C_60_** and the **1_Zn_** *jail*. (c) Temperature‐dependent kinetic experiments for **C_60_** encapsulation by the **1_Zn_** *jail*, showing the calculated rate constants (*k*) and half‐lifetimes (*t*_1/2_). The Eyring plot shown in the inset allowed calculation of the transition enthalpy (Δ*H* ^≠^) and entropy (Δ*S* ^≠^). (d) Free energy surface (FES) profile for the unbinding process of **C_60_@1_Zn_** as a function of the distance (*d*) between the center of mass of **C_60_** and the Zn–Zn centroid computed along a well‐tempered metadynamics simulation (400 ns). Representative structures along the energy profile are depicted.

In sharp contrast to the **1_Zn_** *jail*, when the **2_Zn_** *cage*, which enjoys a larger cavity volume (*V_c_* = 1117 Å^3^ for **2_Zn_** *vs*. *V_c_* = 764 Å^3^ for **1_Zn_**; see Figure S34) and broader windows connecting it to the external medium, or the **3_Zn_** *basket* (*V_c_* = 773 Å^3^ for **3_Zn_**), which presents an “open door” around the missing fourth linker, were mixed with **C**_**60**_ under similar conditions, signs of intermolecular interactions appeared instantly (Figures S39–S41), and no further changes were recorded with time. However, these signs were very different for **2_Zn_** and **3_Zn_**. Upon addition of increasing amounts (> 25 eqs.) of **C**_**60**_, the **2_Zn_** *cage* displayed a progressive shift of the ^1^H NMR signals (Figure S39), indicating weak interactions with the fullerene and a fast NMR exchange between bound and unbound receptors. On the contrary, every substoichiometric addition of **C**_**60**_ to **3_Zn_** was enough to produce a new species in slow NMR exchange with the empty receptor (Figure S39). COSY, NOESY, and ^13^C NMR (Figures S41–S43), as well as MALDI‐TOF MS analysis (Figure S45), together with absorption and emission spectroscopy (Figure S46), additionally confirmed the encapsulation of C_60_ within this *basket*‐shaped host, yielding **3_Zn_** ·**C**_**60**_. Two main conclusions can be drawn from these control experiments that compare the interaction of the **1_Zn_** *jail*, the **2_Zn_** *cage*, and the **3_Zn_** *basket* with **C**_**60**_. First, from the thermodynamic viewpoint, it is clear that the internal cavities generated in **1_Zn_** and **3_Zn_** have the ideal dimensions and inter‐porphyrin distance to bind tightly to **C**_**60**_, leading to quantitative formation of the host:guest complex when reaching the 1:1 stoichiometry within the NMR concentration range. In contrast, the slightly larger size of **2_Zn_** 's cavity impedes the establishment of multiple interactions with the **C_60_** sphere, resulting in binding affinities that are many orders of magnitude lower (see below), which underscores the importance of the fullerene “wrapping” effect. Second, attending to kinetic considerations, it looks that the “windows” in between the linkers connecting the porphyrin macrocycles in **1_Zn_** are too rigid, on one hand, and small, on the other, to allow free diffusion of **C_60_** into the central *jail* cavity. The larger windows in **2_Zn_** and **3_Zn_** enable the entrance of the buckyball into the internal cavities of these receptors with negligible activation energies.

To get more insights into the structure and energetics of the different supramolecular complexes, optimization of the **C_60_@1_Zn_**, **2_Zn_·C_60_**, and **3_Zn_·C_60_** systems was performed at the B3LYP‐D3/cc‐pVDZ level (Figures 3b, S30–S32, and Tables S2–S4). Interaction energies (Δ*E*_int_), calculated as the energy difference between the total energy of the host:guest complex and the energies of its two components at the optimized complex geometry, were also estimated (see Figure S33). Complex **C_60_@1_Zn_** (see Figure 3a,b) shows encapsulated **C_60_** stabilized by multiple non‐covalent contacts involving both the porphyrin platforms (Zn⋯C_60_ and porphyrin⋯C_60_) and the linkers (CH⋯C_60_), with a predicted Δ*E*_int_ value of –324.7 kJ·mol^−1^. More than half of this interaction energy (53.6%) comes from π–π interactions between the **C_60_** guest and the two porphyrins, along with the Zn⋯C_60_ contacts. Δ*E*_int_ between the linkers and **C_60_**, which mainly involves CH⋯π interactions, accounts for 46.4% (11.6% per linker). The absence of one linker in **3_Zn_** provides more flexibility to the **3_Zn_·C_60_** *basket* complex, and the distance between the phenyl rings in *meso* positions that are not covalently connected is shortened (10.23 Å) compared to the three linkers (∼11.95 Å). This structural response is responsible for similar CH···C_60_ contacts in both **C_60_@1_Zn_** and **3_Zn_·C_60_** (Tables S2 and S4) and for almost identical Δ*E*_int_ values (–324.7 and –319.6 kJ·mol^−1^, respectively). The lack of one linker is energetically compensated by a slight rise in the interaction of **C_60_** with the other three linkers (12.6% per linker) and with the two unlinked phenyl rings in *meso* positions (7.8%) (Figure S33). In contrast, in the **2_Zn_·C_60_** complex, the replacement of benzene by naphthalene moieties leads to an expanded structure where **C_60_** interacts preferentially with one of the two porphyrins (Figure S31 and Table S3), showing dissimilar Zn⋯C_60_ distances of 2.93 and 5.59 Å. The naphthalene linkers have now less effective CH⋯π interactions, with contacts in a wider range (2.86 to 4.97 Å) compared to **C_60_@1_Zn_** and **3_Zn_·C_60_** (2.85–3.11 Å, Tables S2–S4). As a result, a significantly lower Δ*E*_int_ value of –229.1 kJ·mol^−1^ is obtained for **2_Zn_·C_60_**, which can be dissected into dominant CH⋯π interactions between **C_60_** and the naphthalene linkers (57.5%, *ca*. 14.4% per linker), and less relevant C_60_⋯porphyrin interactions (38.7% and 3.8% with the close and distant aromatic rings, respectively). Due to the highly rigid nature of all three hosts and the fullerene guest, we expect the enthalpic term of the Gibbs free energy of binding to be dominant over the entropic term. However, because of the technical difficulties arising from the remarkably high association constants and the frozen kinetics of the **C_60_** + **1_Zn_** encapsulation, as detailed below, these thermodynamic components could not be experimentally determined.

In order to calculate the kinetic constant of the slow association process between **1_Zn_** and **C_60_** (*k*_a_), we first corroborated that this supramolecular reaction was indeed bimolecular, first order in [**1_Zn_**] and [**C_60_**]. To this end, we confirmed that the initial reaction rates (conversion < 15%) were directly proportional to the concentration of each of these two reagents in kinetic experiments where the concentration of the other reagent was kept constant (Figure S47). Then, *k*_a_ was determined at 5 different temperatures within the 373‒408 K range in *o*‐xylene‐D_10_ (Figures 3c and S48; and Table S7). The analysis of these data through an Eyring plot (inset in Figure 3c) supplied the activation enthalpy and entropy of the association process as Δ*H* **^‡^** = 79.6 kJ·mol^−1^ and Δ*S* **^‡^** = −79.2 J·mol^−1^ K^−1^. The transition state activation free energy for **C_60_** inclusion within **1_Zn_** was calculated at 373 K as Δ*G* ^≠^ = 109.4 kJ·mol^−1^. This is a remarkably high value for a supramolecular process [30, 31], comparable to the activation energy barriers reported for rotaxane deslipping processes [32, 33] and for the formation of hemicarceplexes [34, 35], thereby accounting for the exceptionally long encapsulation times observed experimentally for **1_Zn_**. As a matter of fact, the exchange dynamics between *basket* **3_Zn_** and complex **3_Zn_·C_60_** is already remarkably slow, despite the open window available in the structure. EXSY NMR experiments (Figure S44) revealed a **C_60_** exchange rate constant between empty and filled **3_Zn_** of *k_ex_* = 0.20 ± 0.02 s^−1^ in toluene‐D_8_ at 298 K.

To understand further the thermodynamic and kinetic behavior for the encapsulation of **C_60_** by the *jail*, *cage*, and *basket* hosts at the molecular level, well‐tempered metadynamics (WT‐MetaD) simulations were performed to estimate the free energy surface (FES) of the unbinding process for the three **C_60_@1_Zn_**, **2_Zn_·C_60_**, and **3_Zn_·C_60_** complexes (Figures 3d, S49, and S50, see the Supporting Information for further details). The association process is thermodynamically favorable in all three cases, with binding free energies (Δ*G*) of 53.7, 22.3, and 43.4 kJ·mol^−1^ for **C_60_@1_Zn_**, **2_Zn_·C_60_**, and **3_Zn_·C_60_** complexes, respectively, which are in excellent agreement with those experimentally reported in Figure 1 (see above). The FES profiles reveal that the insertion of **C_60_** is particularly hampered in **1_Zn_** with a significant energy barrier (*ca*. Δ*G* ^≠^ = 100.1 kJ·mol^−1^). For **2_Zn_** and **3_Zn_**, in contrast, the **C_60_** binding process is almost barrierless and thus basically dependent on diffusion (Figure S49). Consequently, the fast/slow exchange differences observed in NMR experiments between the **2_Zn_·C_60_** and **3_Zn_·C_60_** complexes (Figure S39) should be then attributed to the stronger interactions established between **C**_**60**_ and the **3_Zn_** *basket* compared to the **2_Zn_** *cage*, once **C_60_** enters the host.

We reasoned that the addition to **1_Zn_** of small amounts of a strong acid like TFA, which can induce the reorganization of the dynamic imine bonds and thus the transient detachment of these linkers, should result in the accelerated inclusion of **C_60_** into the **1_Zn_** *jail*. For instance, in the presence of 0.1 eq. of TFA, complete **C_60_** insertion into **1_Zn_** took place in about 1 h at mM NMR concentrations at 298 K, while the addition of 0.5 eq. required just a few minutes (Figure S51). This accelerated kinetics in the presence of TFA allowed us to monitor **C_60_** inclusion at 298 K by absorption spectroscopy at lower µM concentrations within a reasonable time window (< 24 h), so as to analyze and compare the kinetics of this catalyzed process at different TFA ratios (Figure S52) and temperatures (Figure S53). It is interesting to note that fullerene encapsulation can also be accomplished during the formation of **1_Zn_** from their amino and aldehyde precursors, leading directly to the **C_60_@1_Zn_** complex. However, a major difference between this TFA‐catalyzed synthesis from the precursors and the TFA‐catalyzed reorganization of the preformed **1_Zn_** *jail* in the presence of **C_60_** is that the former demanded prolonged heating at 373 K, whereas the latter occurs readily at room temperature. This observation suggests that **C_60_** inclusion into **1_Zn_** does not require the full dissociation of the *jail*’s components, but probably the transient acid‐catalyzed detachment of a few imine bonds is sufficient to access a structure where the fullerene can diffuse freely into the *jail*.

Once **C_60_** is accommodated inside **1_Zn_**, and due to the tiny windows present in this *sp* ^2^‐receptor (calculated in the range of 10.8–12.7 Å) in comparison to the fullerene size, **C_60_** release was even slower, reaching half‐lives (*t*_1/2_) on the scale of weeks even at very high temperatures (> 400 K). This is due to an extremely high activation energy (estimated at 153.8 kJ·mol^−1^) and explains why **1_Zn_** can be regarded as a kinetic *jail* for the fullerene. This deactivated exchange introduced critical limitations in the quantitative analysis of the kinetic and thermodynamic parameters governing this assembly process. On one hand, due to the strong binding established between **1_Zn_** and **C_60_**, (partial) dissociation of the complex would only be achieved at extremely low nM concentrations, a concentration range where even highly sensitive techniques like fluorescence spectroscopy start to be less reliable. On the other hand, the equilibrium under these conditions could only be reached by constant heating at temperatures well above 400 K during several months. These practical restrictions were, however, absent for the **3_Zn_·C_60_** complex, for which we could calculate the association constant by absorption and emission spectroscopy through titration experiments in toluene (Figures S55‐S56). Even at concentrations as low as 10^−7^ M the equilibrium was shifted to the complex side, but these techniques allowed to derive values of $K_{C_{60}}$ = 9.4 × 10^7^ M^−1^ (Δ*G* = −45.5 kJ·mol^−1^) for the formation of the **3_Zn_·C_60_** complex, a magnitude that was taken as a lower threshold for the **C_60_** **@** **1_Zn_** association.

An alternative, more feasible way to expel **C_60_** from the **1_Zn_** *jail* is to employ higher mM concentrations and set up a competition with 4,4′‐bipyridine (***bipy***). As determined in previous work [26], this ditopic heterocyclic guest can bind very strongly to both Zn^II^ centers inside **1_Zn_** due to a remarkably high chelate cooperativity, reaching association constants as high as ***K***_***bipy***_ = 1.2 × 10^8^ M^−1^ in chloroform and ***K***_***bipy***_ = 2.1 × 10^9^ M^−1^ (Δ*G* = –53.2 kJ·mol^−1^) in toluene. In order to avoid kinetic effects, we initially tested this competition between ***bipy*** and **C_60_** with the **3_Zn_** *basket*. First, the complex **3_Zn_·*bipy*** was characterized, and the association constant between ***bipy*** and **3_Zn_** was calculated as ***K***_***bipy***_ = 7.1 × 10^8^ M^−1^ (Δ*G* = −50.5 kJ·mol^−1^) in toluene (see Figures S57–S62). Then, different competition experiments were set up to assess the relative binding strength of ***bipy*** and **C_60_** to the **3_Zn_** *basket* (Figures S63 and S64). The analysis of these competitions reveals that the equilibrium constant for the formation of **3_Zn_·*bipy*** is ca. 20 times larger than that of **3_Zn_·C_60_**, which coincides with the association constant ratio (***K***_***bipy***_/$K_{C_{60}}$) determined in separate experiments, as indicated above. Subsequently, the competition between ***bipy*** and **C_60_** to bind inside **1_Zn_** was analyzed in the same way (Figure S65). As shown in Figure S66, the addition of 1 eq. of ***bipy*** to the **C_60_** **@** **1_Zn_** complex resulted in some instantaneous chemical shifts in the ^1^H NMR spectrum of the *jail*. However, these are not due to the substitution of **C_60_** by ***bipy*** inside the cage, but rather to coordination of ***bipy*** to the Zn^II^ centers outside the *jail*. Aside from these initial shifts, no relevant changes were detected in the ^1^H NMR spectrum of this mixture over the course of 10 days at 298 K. In order to record NMR changes in this mixture and eventually reach equilibrium within a reasonable timeframe, the samples were continuously heated to 413 K in *o*‐xylene‐D_10_ (Figure S66). In these conditions, the equilibrium situation was reached after *ca*. 30 days, resulting in a 41:59 mixture of **1_Zn_·*bipy*** and **C_60_** **@** **1_Zn_** when **1_Zn_**, ***bipy*** and **C_60_** were mixed in a 1:1:1 ratio at 5.0 × 10^−4^ M concentration. Alternatively, the equilibrium could be attained over the course of 24 h by addition of 0.1 eq. of TFA (Figures S67–S69), resulting in a similar composition. As further detailed in Figures S65–S69, the analysis of the different competition equilibria led to an association constant for the **C_60_** **@** **1_Zn_** complex of $K_{C_{60}}$ = 2.7 × 10^9^ M^−1^ (Δ*G* = −53.8 kJ·mol^−1^). This value, amongst the highest reported in the literature for **C_60_** binding by synthetic hosts, is more than one order of magnitude higher than the one obtained for the **3_Zn_·C_60_** complex. This highlights the contribution of the CH⋯π interactions established by each of the four linkers to stabilize the whole assembly, which amounts approximately to ΔΔ*G* = −8.3 kJ·mol^−1^ (15.4% of total Δ*G*), which is in concordance with the Δ*E*_int_ per linker (11.6%) proposed above from theoretical calculations.

## 3 Conclusion

In this work, we have demonstrated that the rigid *sp* ^2^‐receptor **1_Zn_** functions as an exceptionally efficient and mechanically constraining host for **C_60_**. Its highly complementary, pseudo‐spherical cavity enables broad surface contact with the fullerene, producing one of the strongest reported binding affinities for **C_60_** among synthetic hosts. Theoretical calculations reveal a similar weight played by π–π stacking and Zn⋯C_60_ interactions, on one side, and CH⋯π contacts, on the other, in stabilizing the supramolecular complex. More importantly, the encapsulation process is governed by unusually high kinetic barriers, leading to extremely slow guest exchange events above 400 K and resulting in a genuine kinetic trap for **C_60_** at room temperature. Comparisons with the related **2_Zn_** *cage* and **3_Zn_** *basket* underscore the decisive role of window size, cavity fit, and linker rigidity in controlling both thermodynamic stability and kinetic accessibility. Furthermore, acid‐induced imine dynamics reveals a pathway for modulating encapsulation rates without compromising structural integrity. Altogether, these findings provide fundamental insights into the design of mechanically restricted host–guest systems and open new avenues for controlling guest capture, release, and stabilization using structurally preorganized molecular architectures.

## Author Contributions

**A. Priscila Gia**: investigation, methodology, validation, formal analysis, writing – review and editing, data curation. **Fernando G. Guijarro**: investigation, methodology, validation, formal analysis, data curation. **Pau Armada**: investigation, formal analysis. **Daniel Aranda**: investigation, formal analysis. **Josefina Perles**: investigation, data curation. **Enrique Ortí**: writing – review and editing, supervision. **Juan Aragó**: supervision, writing – review and editing, validation. **David González‐Rodríguez**: resources, supervision, project administration, writing – review and editing, writing – original draft, funding acquisition, conceptualization. **Alberto de Juan**: conceptualization, investigation, resources, supervision, methodology, validation, writing – original draft, writing – review and editing, project administration, data curation.

## Conflicts of Interest

The authors declare no conflicts of interest.

## Supporting information



Additional experimental and computational details can be found online in the Supporting Information section.Crystallographic data for the structure reported in this paper have been deposited within the Cambridge Crystallographic Data Centre as CSD‐2550583.**Supporting File**: anie74747‐sup‐0001‐SuppMat.pdf.

## Data Availability

The data that support the findings of this study are available from the corresponding author upon reasonable request.

## References

[1] V. Biju , “Chemical Modifications and Bioconjugate Reactions of Nanomaterials for Sensing, Imaging, Drug Delivery and Therapy,” Chemical Society Reviews 43 (2014): 744–764, 10.1039/C3CS60273G. 24220322

[2] Z. Markovic and V. Trajkovic , “Biomedical Potential of the Reactive Oxygen Species Generation and Quenching by Fullerenes (C_60_),” Biomaterials 29 (2008): 3561–3573, 10.1016/j.biomaterials.2008.05.005. 18534675

[3] F. Wudl , “Fullerene Materials,” Journal of Materials Chemistry 12 (2002): 1959–1963, 10.1039/b201196d.

[4] A. A. Popov , S. Yang , and L. Dunsch , “Endohedral Fullerenes,” Chemical Reviews 113 (2013): 5989–6113, 10.1021/cr300297r. 23635015

[5] Y. Fang , C. Bi , D. Wang , and J. Huang , “The Functions of Fullerenes in Hybrid Perovskite Solar Cells,” ACS Energy Letters 2 (2017): 782–794, 10.1021/acsenergylett.6b00657.

[6] “Supramolecular Chemistry of Fullerenes and Carbon Nanotubes”, Eds.: N. Martín , J. F. Nierengarten (Wiley‐VCH, 2012).

[7] E. M. Pérez and N. Martín , “Curves Ahead: Molecular Receptors for Fullerenes Based on Concave–Convex Complementarity,” Chemical Society Reviews 37 (2008): 1512–1519, 10.1039/b802589b. 18648677

[8] D. Canevet , E. M. Pérez , and N. Martín , “Wraparound Hosts for Fullerenes: Tailored Macrocycles and Cages,” Angewandte Chemie 50 (2011): 9248–9259, 10.1002/anie.201101297. 21901802

[9] K. Tashiro and T. Aida , “Metalloporphyrin Hosts for Supramolecular Chemistry of Fullerenes,” Chemical Society Reviews 36 (2007): 189–197, 10.1039/B614883M. 17264922

[10] C. García‐Simón , M. Costas , and X. Ribas , “Metallosupramolecular Receptors for Fullerene Binding and Release,” Chemical Society Reviews 45 (2016): 40–62, 10.1039/c5cs00315f. 26456881

[11] A. S. Oshchepkov , “Buckybowl Molecular Tweezers for Recognition of Fullerenes,” ChemPhysChem 25 (2024): e202400435, 10.1002/cphc.202400435. 38775747

[12] X. Chang , Y. Xu , and M. von Delius , “Recent Advances in Supramolecular Fullerene Chemistry,” Chemical Society Reviews 53 (2024): 47–83, 10.1039/D2CS00937D. PMC10759306 37853792

[13] W. Song and X. Shao , “Buckybowl‐Based Fullerene Receptors,” Chemistry – A European Journal 31 (2025): e202403383, 10.1002/chem.202403383. 39446344

[14] C. Yu , Y. Jin , and W. Zhang , “Shape‐Persistent Arylene Ethynylene Organic Hosts for Fullerenes,” Chemical Record 15 (2015): 97–106, 10.1002/tcr.201402076. 25464153

[15] M. Yanagisawa , K. Tashiro , M. Yamasaki , and T. Aida , “Hosting Fullerenes by Dynamic Bond Formation With an Iridium Porphyrin Cyclic Dimer: a “Chemical Friction” for Rotary Guest Motions,” Journal of the American Chemical Society 129 (2007): 11912–11913, 10.1021/ja0747526. 17845049

[16] H. Shimizu , J. D. Cojal González , M. Hasegawa , et al., “Synthesis, Structures, and Photophysical Properties of π‐Expanded Oligothiophene 8‐mers and Their Saturn‐Like C_60_ Complexes,” Journal of the American Chemical Society 137 (2015): 3877–3885, 10.1021/jacs.5b00291. 25699988

[17] Y. Yamamoto , E. Tsurumaki , K. Wakamatsu , and S. Toyota , “Nano‐Saturn: Experimental Evidence of Complex Formation of an Anthracene Cyclic Ring With C_60_ ,” Angewandte Chemie International Edition 57 (2018): 8199–8202, 10.1002/anie.201804430. 29846033

[18] S.‐Z. Zhan , J.‐H. Li , G.‐H. Zhang , et al., “Coordination Disk‐Type Nano‐Saturn Complexes,” Chemical Communications 56 (2020): 3325–3328, 10.1039/D0CC00532K. 32083264

[19] L. K. S. von Krbek , C. A. Schalley , and P. Thordarson , “Assessing Cooperativity in Supramolecular Systems,” Chemical Society Reviews 46 (2017): 2622–2637, 10.1039/c7cs00063d. 28352870

[20] C. G. Claessens , D. González‐Rodríguez , R. S. Iglesias , and T. Torres , “Convex–Convex and Concave–Convex Interactions Between C_60_ and Non‐Planar Aromatic Subphthalocyanine Macrocycle in Both Covalent and Supramolecular Arrays,” Comptes Rendus Chimie 9 (2006): 1094–1099, 10.1016/j.crci.2005.12.006.

[21] J. Calbo , A. de Juan , J. Aragó , et al., “Understanding the Affinity of Bis‐exTTF Macrocyclic Receptors Towards Fullerene Recognition,” Physical Chemistry Chemical Physics 21 (2019): 11670–11675, 10.1039/C9CP01735F. 31125037

[22] R. Banerjee , D. Chakraborty , and P. S. Mukherjee , “Molecular Barrels as Potential Hosts: From Synthesis to Applications,” Journal of the American Chemical Society 145 (2023): 7692–7711, 10.1021/jacs.3c01084. 36976105

[23] M. Miklitz , L. Turcani , R. L. Greenaway , and K. E. Jelfs , “Computational Discovery of Molecular C_60_ Encapsulants With an Evolutionary Algorithm,” Communications Chemistry 3 (2020): 10, 10.1038/s42004-020-0255-8. PMC9814092 36703408

[24] T. Matsuno , S. Sato , R. Iizuka , and H. Isobe , “Molecular Recognition in Curved π‐systems: Effects of π‐Lengthening of Tubular Molecules on Thermodynamics and Structures,” Chemical Science 6 (2015): 909–916, 10.1039/C4SC02812K. PMC5811110 29560176

[25] A. P. Gia , A. de Juan , D. Aranda , et al., “Highly Rigid, yet Conformationally Adaptable, Bisporphyrin *sp* ^2^‐Cage Receptors Afford Outstanding Binding Affinities, Chelate Cooperativities, and Substrate Selectivities,” Journal of the American Chemical Society 147 (2025): 918–931, 10.1021/jacs.4c13756. PMC11726566 39700308

[26] D. Wróbel and A. Graja , “Photoinduced Electron Transfer Processes in Fullerene–Organic Chromophore Systems,” Coordination Chemistry Reviews 255 (2011): 2555–2577, 10.1016/j.ccr.2010.12.026.

[27] D. González‐Rodríguez , E. Carbonell , G. D. M. Rojas , C. A. Castellanos , D. M. Guldi , and T. Torres , “Activating Multistep Charge‐Transfer Processes in Fullerene−Subphthalocyanine−Ferrocene Molecular Hybrids as a Function of π−π Orbital Overlap,” Journal of the American Chemical Society 132 (2010): 16488–16500, 10.1021/ja105864r. 21033683

[28] M. García‐Iglesias , K. Peuntinger , A. Kahnt , et al., “Supramolecular Assembly of Multicomponent Photoactive Systems via Cooperatively Coupled Equilibria,” Journal of the American Chemical Society 135 (2013): 19311–19318, 10.1021/ja410114d. 24328274

[29] D. A. Rothschild , W. P. Kopcha , A. Tran , J. Zhang , and M. C. Lipke , “Gram‐Scale Synthesis of a Covalent Nanocage That Preserves the Redox Properties of Encapsulated Fullerenes,” Chemical Science 13 (2022): 5325–5332, 10.1039/D2SC00445C. PMC9093146 35655559

[30] M. D. Pluth and K. N. Raymond , “Reversible Guest Exchange Mechanisms in Supramolecular Host–Guest Assemblies,” Chemical Society Reviews 36 (2007): 161–171, 10.1039/B603168B. 17264920

[31] S. Akine and Y. Sakata , “Control of Guest Binding Kinetics in Macrocycles and Molecular Cages,” Chemistry Letters 49 (2020): 428–441, 10.1246/cl.200017.

[32] A. Affeld , G. M. Hübner , C. Seel , and C. A. Schalley , “Rotaxane or Pseudorotaxane? Effects of Small Structural Variations on the Deslipping Kinetics of Rotaxanes With Stopper Groups of Intermediate Size,” European Journal of Organic Chemistry 2001 (2001): 2877–2890, 10.1002/1099-0690(200108)2001:15<2877::AID-EJOC2877>3.0.CO;2-R.

[33] P. Linnartz and C. A. Schalley , “Deslipping of Rotaxanes With Axle Center Pieces of Different Lengths,” Supramolecular Chemistry 16 (2004): 263–267, 10.1080/1061027042000204010.

[34] M.‐J. Li , C.‐H. Huang , C.‐C. Lai , and S.‐H. Chiu , “Hemicarceplex Formation With a Cyclotriveratrylene‐Based Molecular Cage Allows Isolation of High‐Purity (≥99.0%) C_70_ Directly From Fullerene Extracts,” Organic Letters 14 (2012): 6146–6149, 10.1021/ol3027957. 23228019

[35] K.‐S. Liu , M.‐J. Li , C.‐C. Lai , and S.‐H. Chiu , “Incarceration of Higher‐Order Fullerenes Within Cyclotriveratrylene‐Based Hemicarcerands Allows Selective Isolation of C_76_, C_78_, and C_84_ From a Commercial Fullerene Mixture,” Chemistry – A European Journal 22 (2016): 17468–17476, 10.1002/chem.201603451. 27778390

