# Supplementary material for: A Mechanical sp 2‐Jail for C60 Fullerene
Source: Angew Chem Int Ed Engl. 2026 Sep 11;65(44):e7486858. doi: 10.1002/anie.7486858 (PMC13703911; doi:10.1002/anie.7486858)
Supplement: Supplementary file 1 [file ANIE-65-e7486858-s001.pdf]

SUPPORTING INFORMATION FOR:

"A Mechanical *sp*^{2}-*Jail* for C_{60} Fullerene"

A. Priscila Gia,[a]+ Fernando G. Guijarro,[a]+ Pau Armada,[b] Daniel Aranda,[b,c] Josefina Perles,[d] Enrique Ortí,[b] Juan Aragó,*[b] David González-Rodríguez*[a,e] and Alberto de Juan*[a,e]

[a] A. Priscila Gia, Dr. Fernando G. Guijarro, Prof. Dr. David González-Rodríguez* and Dr. Alberto de Juan*
Nanostructured Molecular Systems and Materials group, Organic Chemistry Department,
Universidad Autónoma de Madrid,
28049 Madrid, Spain
E-mail: juan.arago@uv.es, david.gonzalez.rodriguez@uam.es, alberto.dejuan@uam.es

[b] Pau Armada, Dr. Daniel Aranda, Prof. Dr. Enrique Ortí, Dr. Juan Aragó*
Institute of Molecular Science,
Universitat de València,
Catedrático José Beltrán 2, 46980 Paterna (Valencia), Spain

[c] Dr. Daniel Aranda
Andalucía Tech, Science Faculty, Department of Physical Chemistry,
Universidad de Málaga,
29071 Málaga, Spain de València

[d] Dr. Josefina Perles
Single Crystal X-ray Diffraction Laboratory, SidI,
Universidad Autónoma de Madrid
28049 Madrid, Spain

[e] Prof. Dr. David González-Rodríguez* and Dr. Alberto de Juan*
Institute for Advanced Research in Chemical Sciences (IAdChem),
Universidad Autónoma de Madrid,
28049 Madrid, Spain

[+] Contributed equally to this work

TABLE OF CONTENTS

General Methods S3

S0. Synthetic Procedures S4

S1. Encapsulation of C_{60} fullerene into 1_{Zn} as a function of time at different temperatures S30

S2. Encapsulation of C_{60} fullerene into 1_{Zn}. Characterization of the $C_{60}@1_{Zn}$ complex. S31

S3. Density functional theory calculations on $C_{60}@1_{Zn}$, $2_{Zn}·C_{60}$ and $3_{Zn}·C_{60}$ S35

S4. Solid state crystal structure of $C_{60}@1'_{Zn}$. S43

S5. Comparison of C_{60} encapsulation into 2_{Zn} and 3_{Zn}. S48

S6. Characterization of C_{60} fullerene encapsulation into 3_{Zn}. S50

S7. Determination of the partial and global reaction orders for C_{60} fullerene encapsulation into 1_{Zn}. S57

S8. Determination of the kinetic constants (*k*) at different temperatures and the activation energy barrier (E_a) for C_{60} fullerene encapsulation into 1_{Zn}. S58

S9. Well-Tempered Metadynamics Simulations S61

S10. C_{60} fullerene encapsulation into 1_{Zn} activated via acid catalysis with TFA S64

S11. Thermodynamic characterization of Host-Guest complex: $3_{Zn}·C_{60}$. S67

S12. Thermodynamic characterization of Host-Guest complex: 3_{Zn}·*bipy*. S69

S13. Competition experiments between *bipy* and C_{60} to bind inside 3_{Zn}. S74

S14. Competition experiments between *bipy* and C_{60} to bind inside 1_{Zn} in the presence of TFA. S76

References S81

General Methods

Mass Spectrometry (MS) and **High Resolution-Mass Spectrometry (HRMS)** MALDI-TOF spectra were obtained from a BRUKER ULTRAFLEX III instrument equipped with a nitrogen laser operating at 337 nm, and APCI and ESI spectra were obtained from a BRUKER MAXIS II instrument. **NMR** spectra were recorded with *BRUKER AV-II* 300 MHz, *BRUKER AV* 500 MHz or *BRUKER DRX* 500 MHz instruments. 2D ^{1}H-^{1}H NOESY experiments were performed using a mixing time (τ_m) of 0.4 s. The temperature was actively controlled at 298 K. Chemical shifts are measured in ppm using the signals of the deuterated solvent as the internal standard [$CDCl_3$ calibrated at 7.26 (^{1}H) and 77.16 ppm (^{13}C), DMSO-d_6 calibrated at 2.50 (^{1}H) and 39.5 ppm (^{13}C), and toluene-calibrated at 7.09 (^{1}H) and 137.86 ppm (^{13}C)]. **Manual column chromatography** was carried out on silica gel *Merck-60* (230-400 mesh, 60 Å), and TLC on aluminum sheets precoated with silica gel 60 F254 (Merck). **Automatic column chromatography** was carried out on CombiFlash® Rf 150-Teledyne Isco. **UV-Visible** experiments were conducted using a *JASCO V-660* apparatus. **Emission spectra** were recorded in a *JASCO FP-8600* equipment using excitation and emission bandwidths of 5–10 nm in both cases, and a 1000 ms response. In both instruments the temperature was controlled using a *JASCO* Peltier thermostated cell holder at 298 K, adjustable temperature slope, and accuracy of ± 0.1 K. **X-ray structures** were obtained from *Rigaku XtaLAB Synergy R* diffractometer equipped with a *HyPix-Arc 100º* Curved X-ray detector irradiating with a rotator anode (Cu Kα, λ = 1.54184) at low temperature (150 K).

Starting materials

Chemicals were purchased from commercial suppliers and used without further purification. Solid hygroscopic reagents were dried in a vacuum oven before use. Pyrrole was distilled prior to be used. The synthetic procedure and the characterization data of compounds **6**, **7**, **8**, **9,** and the cage $\mathbf{1_{Zn}}$ shown in Scheme S1 have been previously reported. [1,2]

S0. Synthetic Procedures

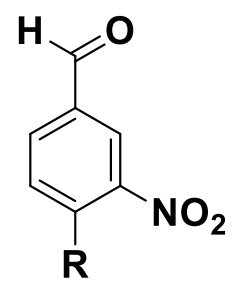

6

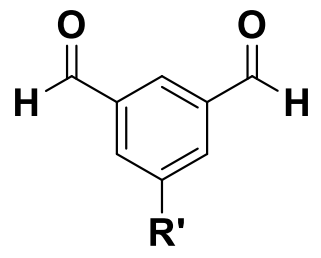

7

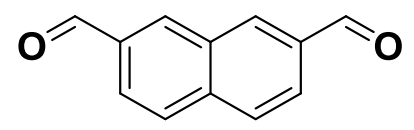

8

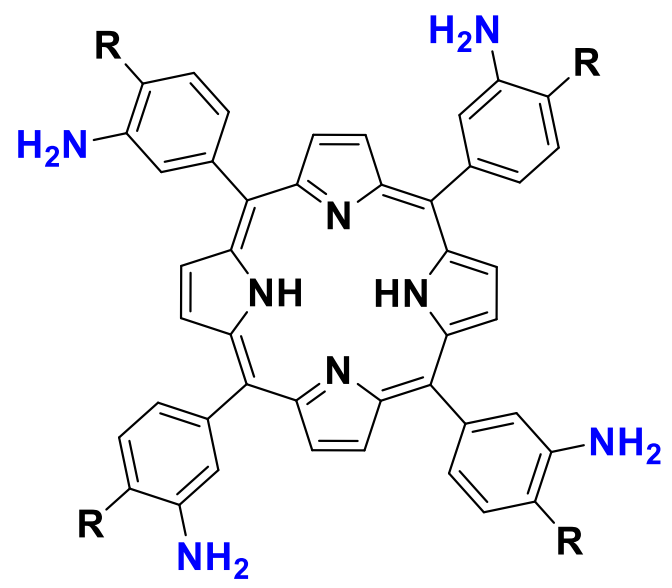

9

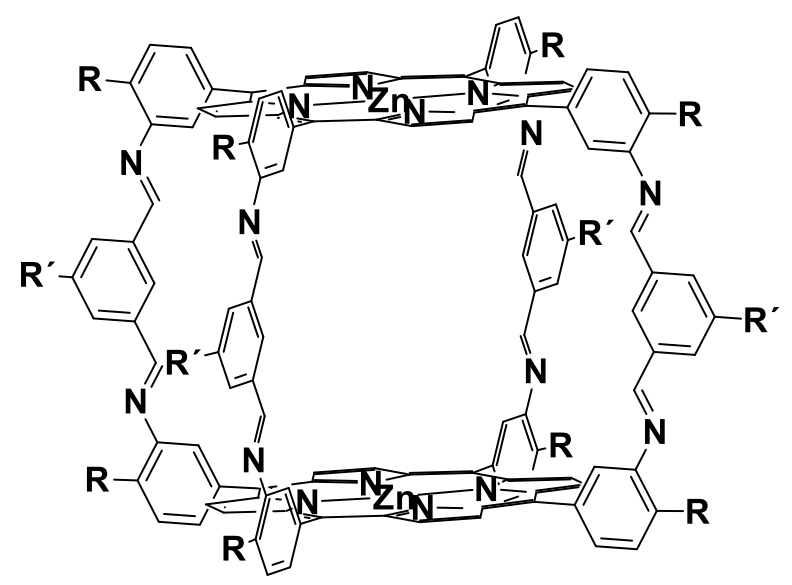

$\mathbf{1_{Zn}}$

R = OC_6H_{13} ; R' = $OC_{12}H_{25}$

Scheme S1. Compounds previously described.

Synthesis of tetra-amino-Zn-porphyrin 10.

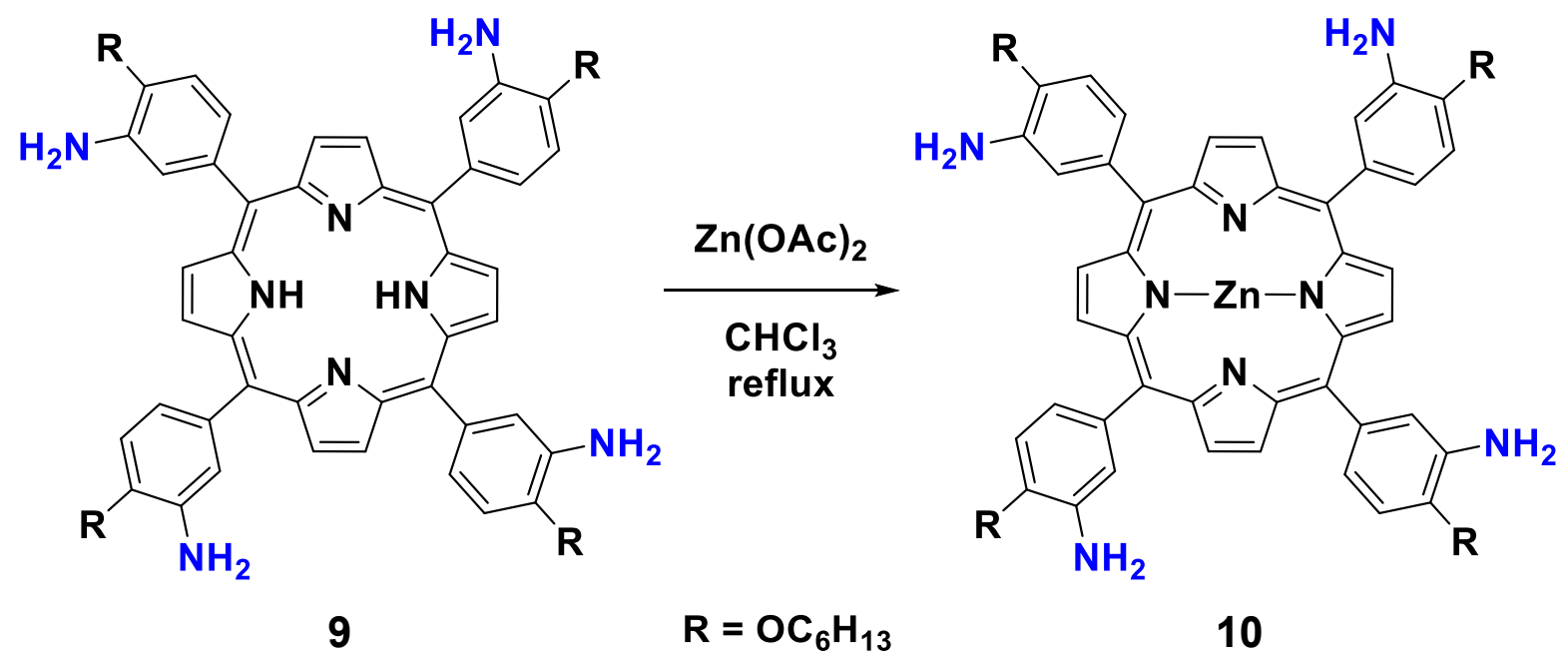

Scheme S2. Synthesis of tetra-amino porphyrin **10**.

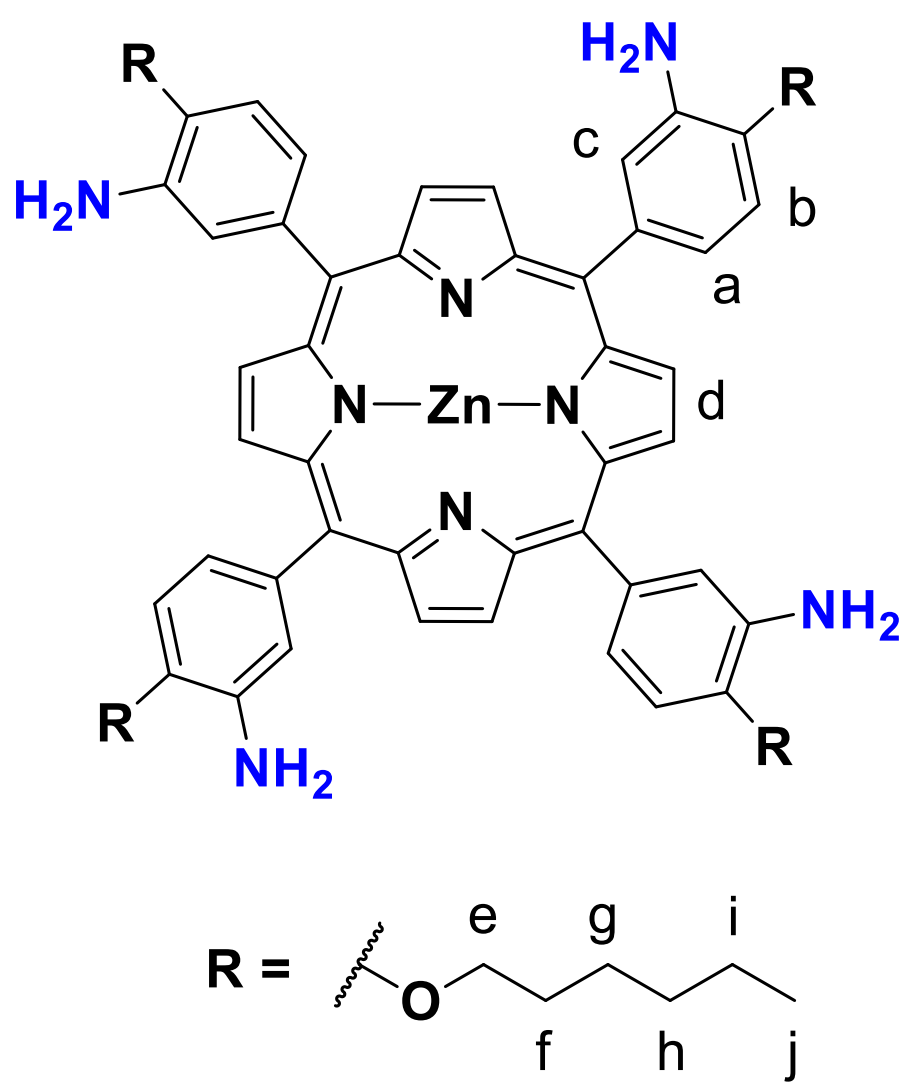

10. To a solution of **9** (200.0 mg, 0.186 mmol, 1 eq) in $CHCl_3$ (100 mL) $Zn(OAc)_2$ (49.0 mg, 0.232 mmol, 1.25 eq) was added and heated at reflux under inert atmosphere overnight. Then, MeOH was added helping to crash out a purple solid and the suspension was filtered and the solid rinsed with MeOH, obtaining the desired compound as a purple solid (210.0 mg, 99 %).

^{1}H NMR (300 MHz, THF-d_8) δ (ppm) = 8.90 (s, 8H, H^d), 7.50 (d, J = 2.1 Hz, 4H, H^c), 7.37 (dd, J = 8.0, 2.1 Hz, 4H, H^a), 7.10 (d, J = 8.0, 4H, H^b), 4.53 (s, 8H, **NH_2**), 4.27 (t, J = 6.4 Hz, 8H, H^e), 2.01 (q, J = 6.4 Hz, 8H, H^f), 1.70 – 1.62 (m, 8H, H^g), 1.54 – 1.45 (m, 16H, H^{h+i}), 1.01 (t, J = 6.5 Hz, 12H, H^j).

^{13}C NMR (75 MHz, THF-d_8) δ (ppm) = 152.0, 147.7, 138.2, 137.2, 132.8, 125.8, 123.2, 122.5, 110.5, 69.9, 33.6, 31.5, 27.9, 24.5, 15.4.

HRMS (MALDI, DCTB+PPGNa 1000): Mass (*m/z*) calculated for $C_{68}H_{80}N_8O_4Zn$ $[M]^+$: 1136.5589; found: 1136.5574.

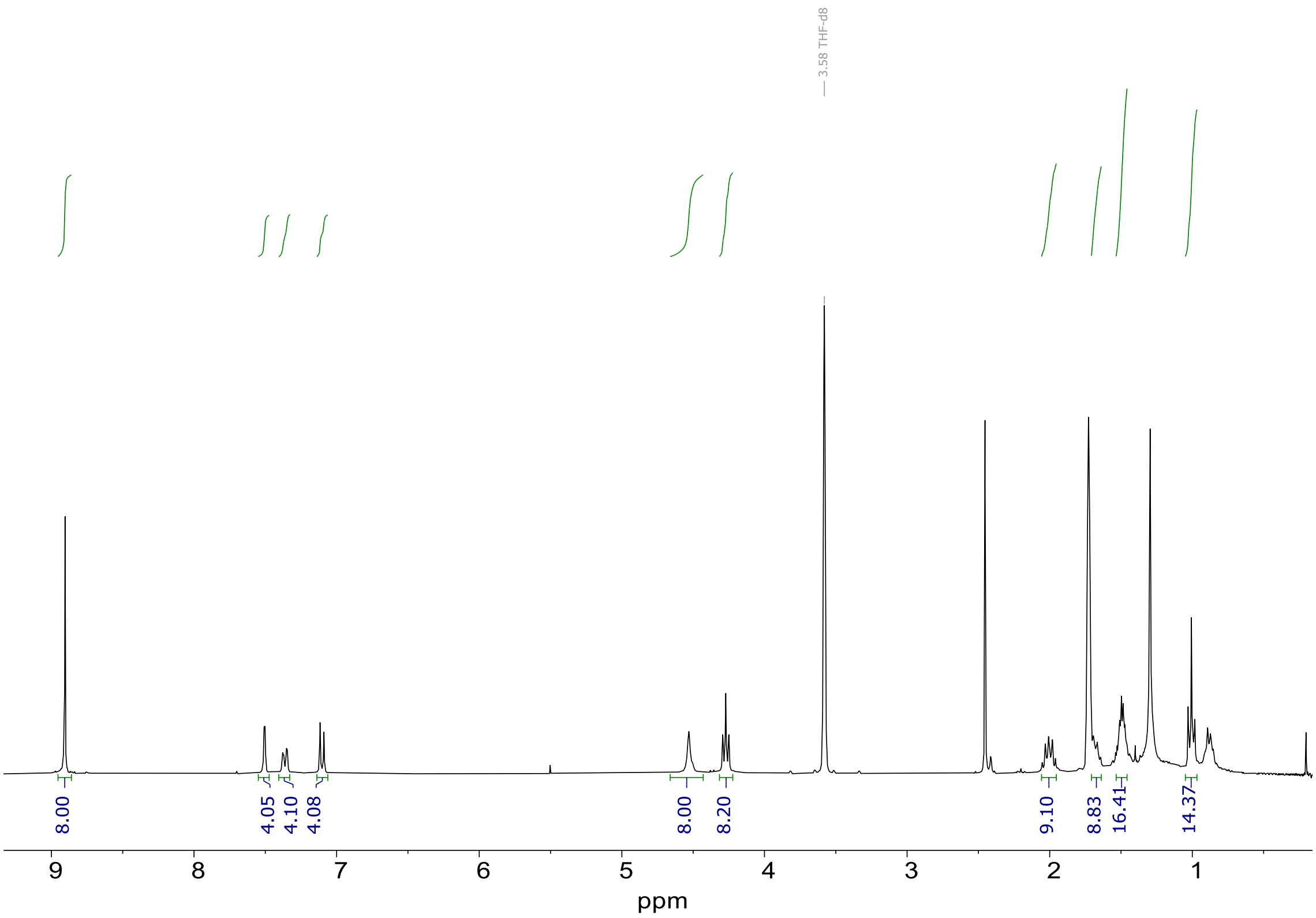

Figure S1. ^{1}H NMR spectrum of compound **10** in THF-d_8 at 298 K.

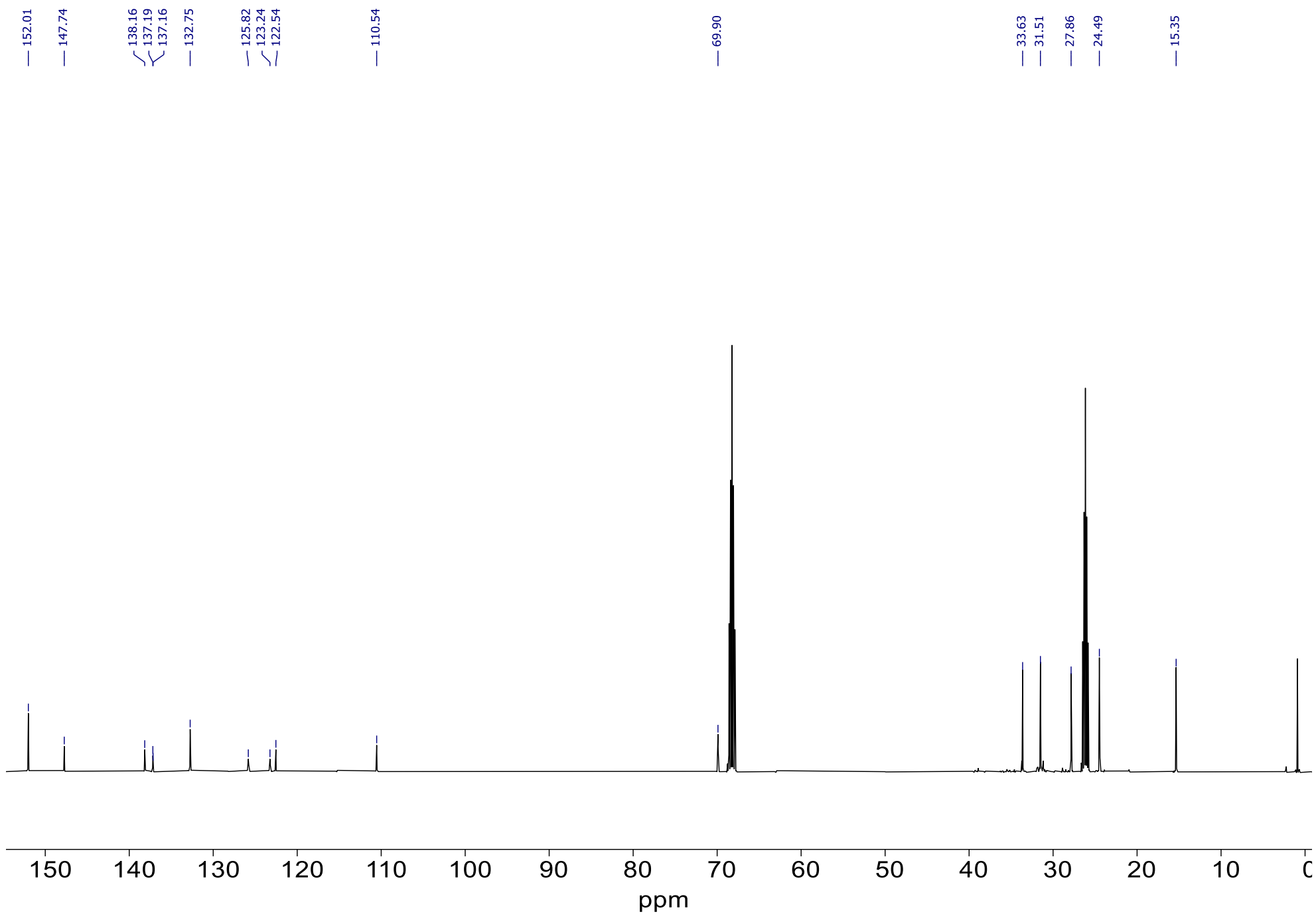

Figure S2. ^{13}C NMR spectrum of compound **10** in THF-d_8 at 298 K.

Synthesis of cage 2_{Zn}.

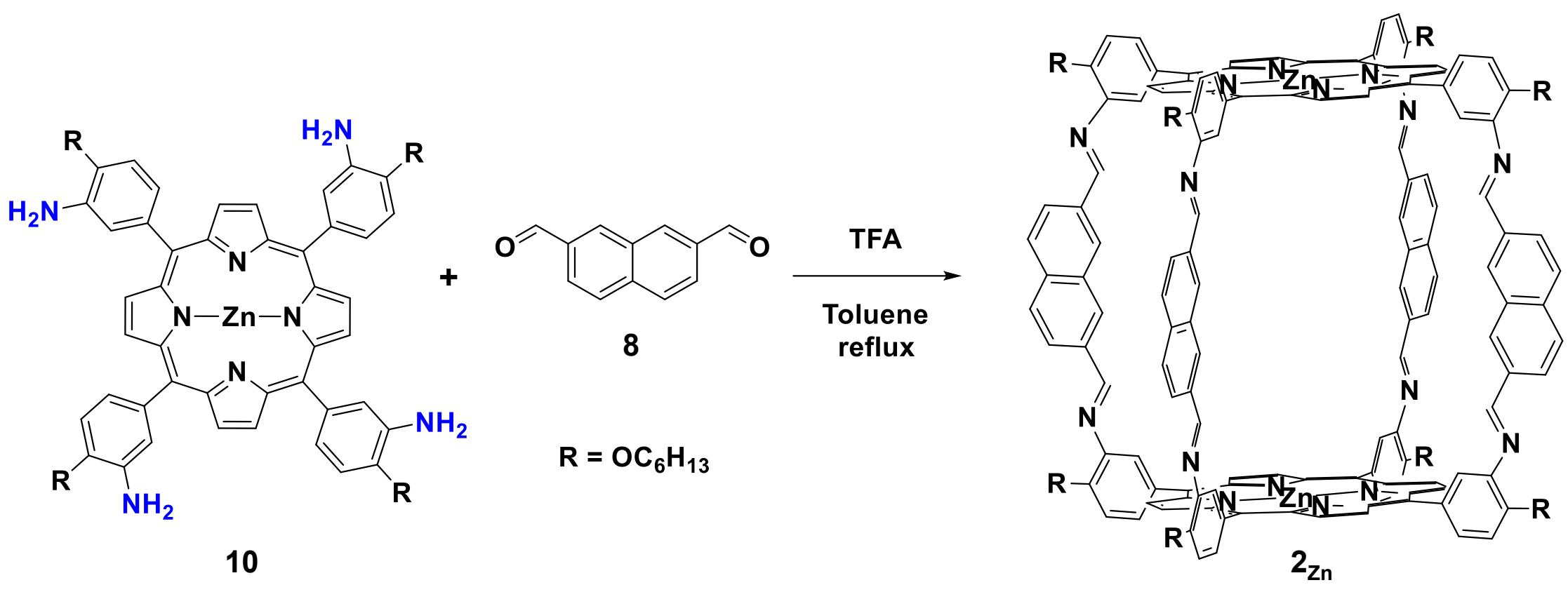

Scheme S3. Synthesis of the porphyrin cage $\mathbf{2}_{Zn}$.

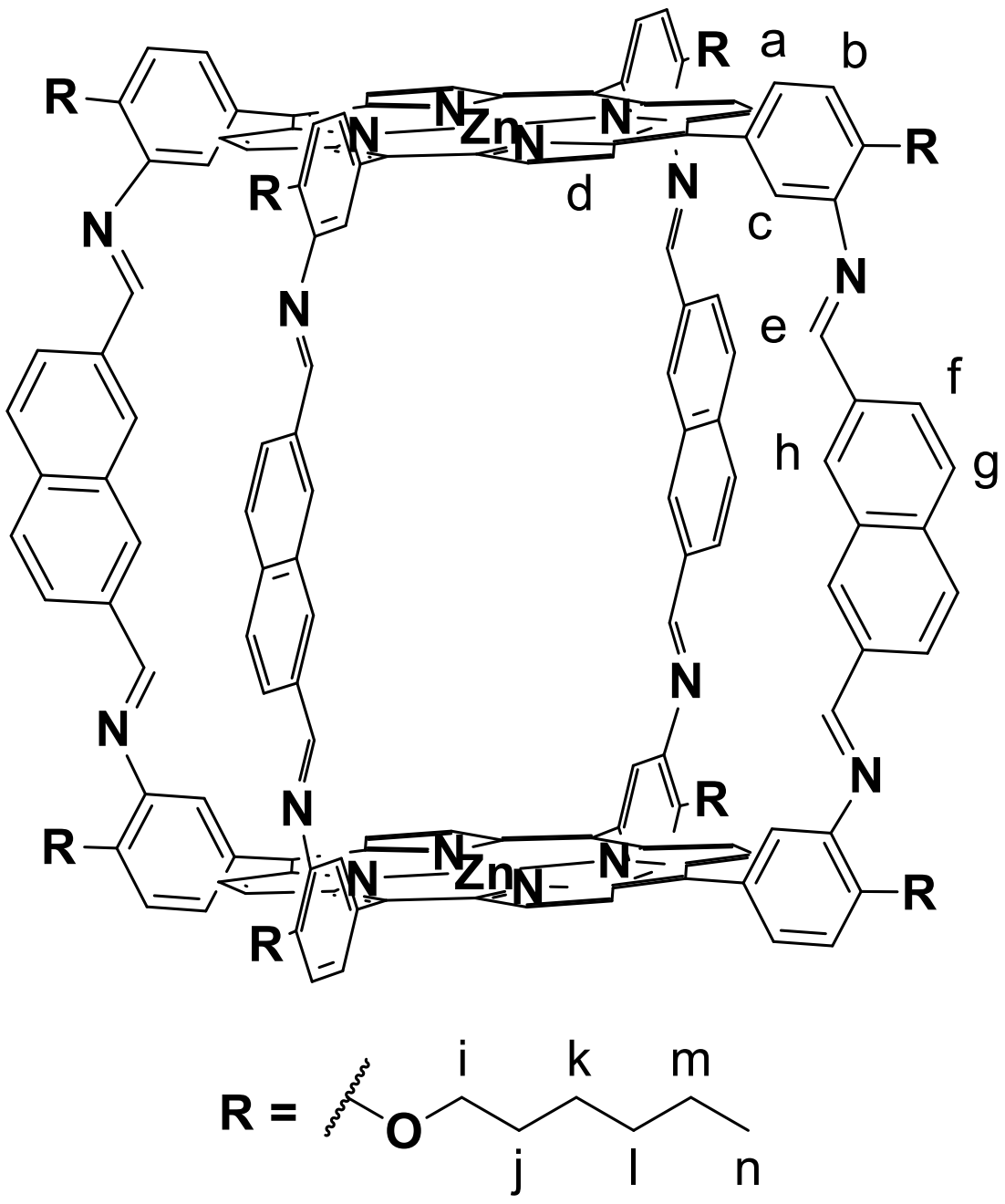

Molecular cage $\mathbf{2_{Zn}}$. To a solution of porphyrin **10** (60.65 mg, 0.053 mmol, 1 eq) and linker **8** (19.77 mg, 0.107 mmol, 2 eq) in toluene (36 mL), TFA (0.027 mmol, 0.5 eq) was added under inert atmosphere. The mixture was heated at reflux, overnight. Then, MeCN was added helping to crash out a purple solid and the suspension was filtered and the solid rinsed with MeCN. Afterwards, the obtained solid was purified by column chromatography using basic aluminium oxide as stationary phase and a DCM as the eluent, obtaining the desired compound as a purple solid (42.10 mg, 55 %).

^{1}H NMR (300 MHz, toluene-d_8) δ (ppm) = 9.20 (s, 16H, H^d), 8.51 (d, *J* = 8.8 Hz, 8H, H^f), 8.31 (s, 8H, H^e), 8.22 (dd, *J* = 7.9, 2.2 Hz, 8H, H^a), 7.94 (d, *J* = 2.2 Hz, 8H, H^c), 7.40 (d, *J* = 8.8 Hz, 8H, H^g), 7.21 – 7.17 (m, 16H, H^{b+h}), 4.15 (t, *J* = 6.3 Hz, 16H, H^i), 1.94 – 1.85 (m, 16H, H^j), 1.64 – 1.55 (m, 16H, H^k), 1.39 – 1.33 (m, 32H, H^{l+m}), 0.98 – 0.92 (m, 24H, H^n).

^{13}C NMR (75 MHz, toluene-d_8) *δ* (ppm) = 160.0, 153.4, 151.4, 141.5, 137.1, 136.0, 133.5, 132.7, 132.4, 132.2, 127.2, 125.8, 121.0, 113.8, 70.8, 32.3, 30.5, 26.6, 23.2, 14.2.

HRMS (MALDI): Mass m/z calculated for $C_{184}H_{176}N_{16}O_8Zn_2$ $[M]^+$ 2865.2435, found: 2865.2456.

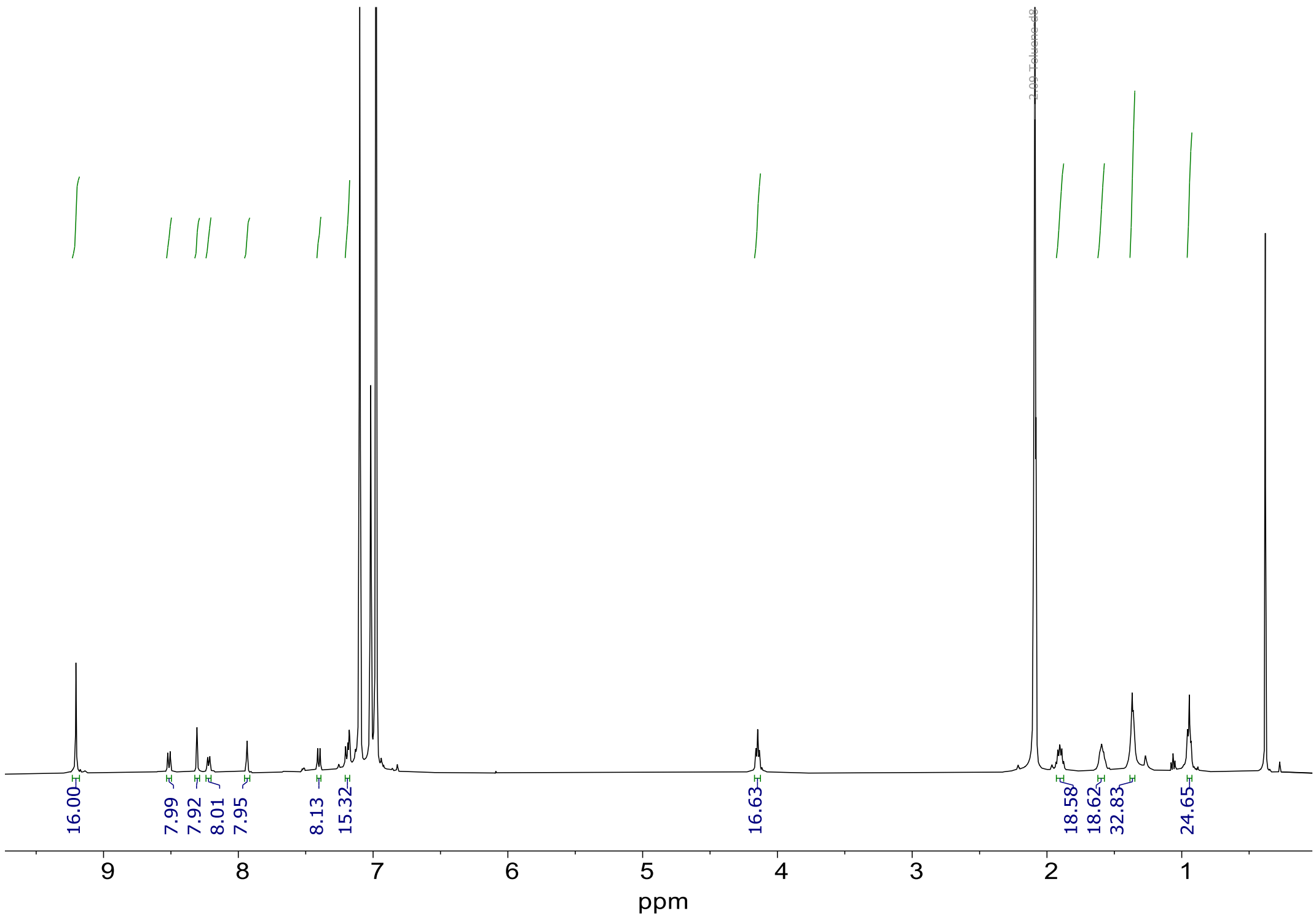

Figure S3. ^{1}H NMR spectrum of cage $\mathbf{2_{Zn}}$ in toluene-d_8 at 298 K.

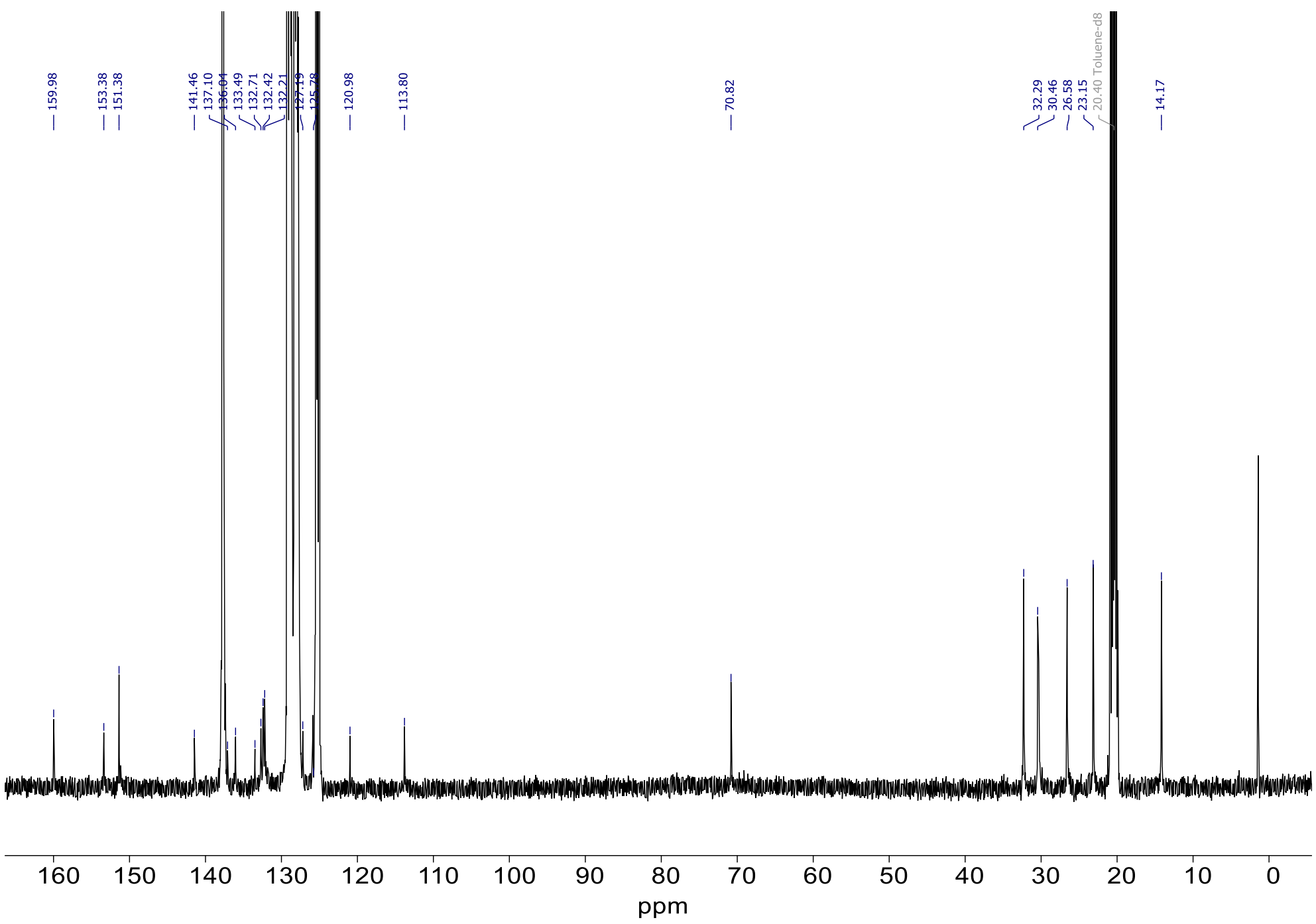

Figure S4. ^{13}C NMR spectrum of cage $\mathbf{2_{Zn}}$ in toluene-d_8 at 373 K.

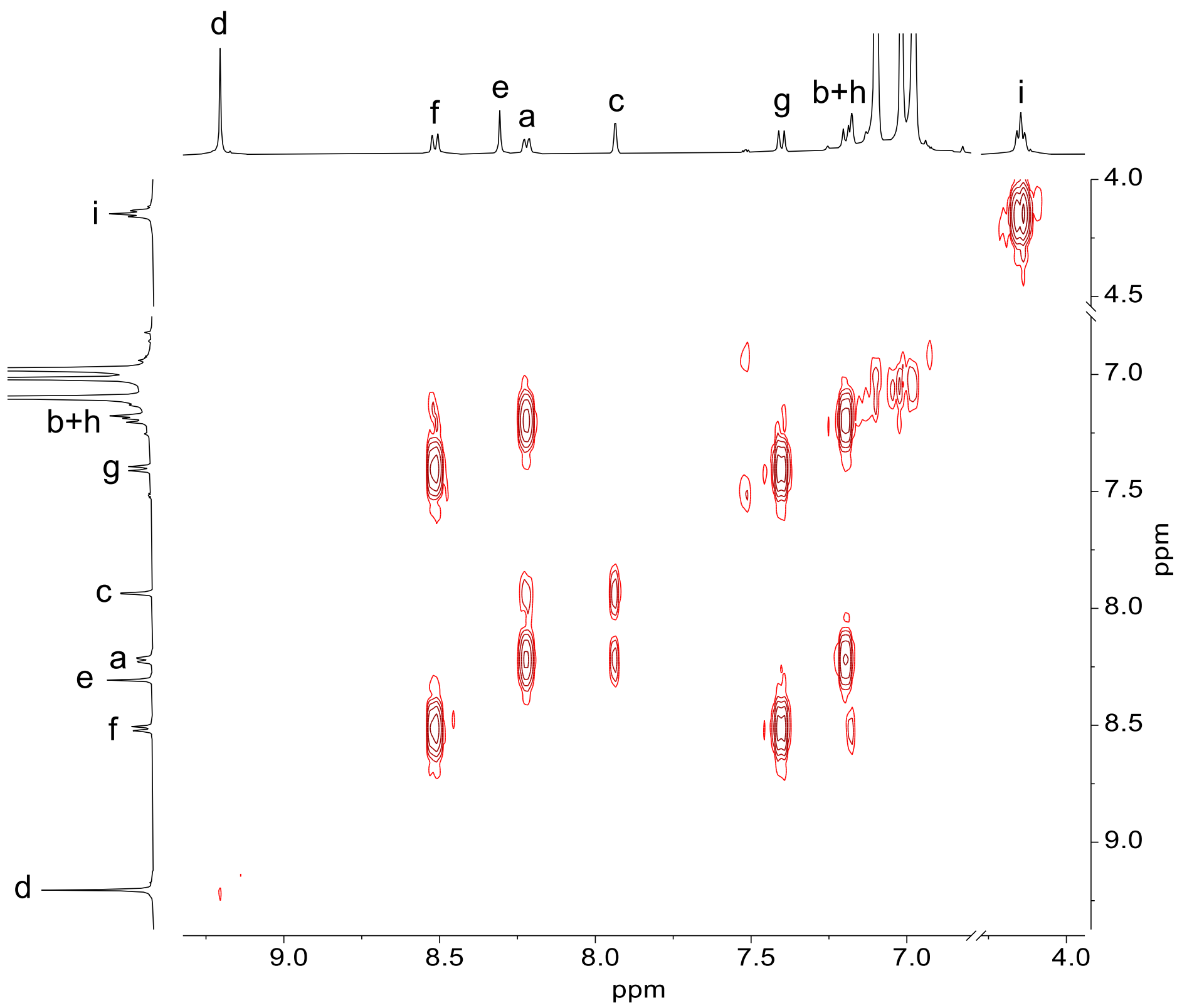

Figure S5. ^{1}H-^{1}H COSY NMR spectrum of cage **2$_{Zn}$** in toluene-d$_8$ at 298 K.

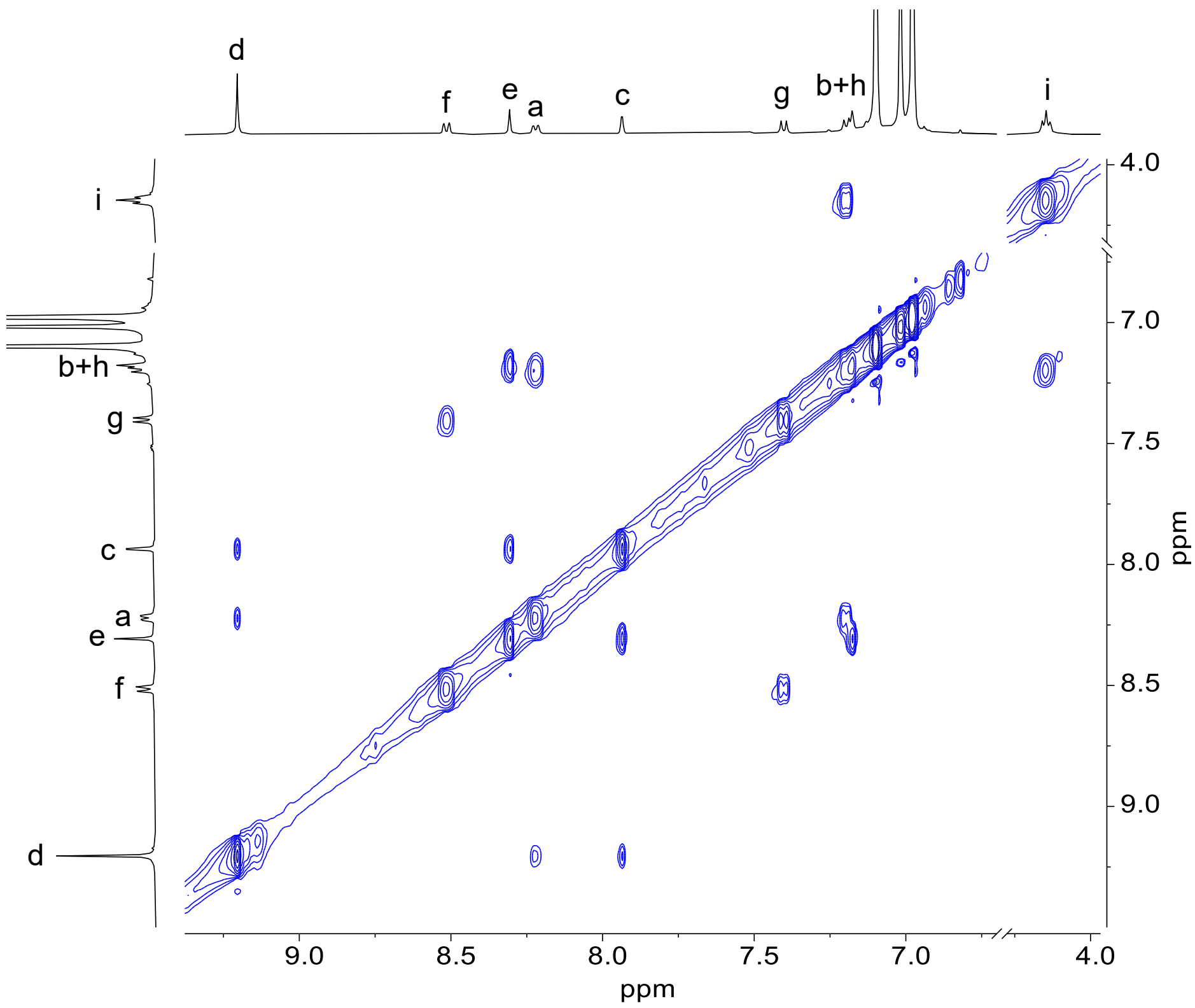

Figure S6. 2D ^{1}H-^{1}H NOESY NMR spectrum of cage **2$_{Zn}$** in toluene-d$_8$ at 298 K.

Synthesis of AB3-symmetric tri-amino-Zn-porphyrin 5.

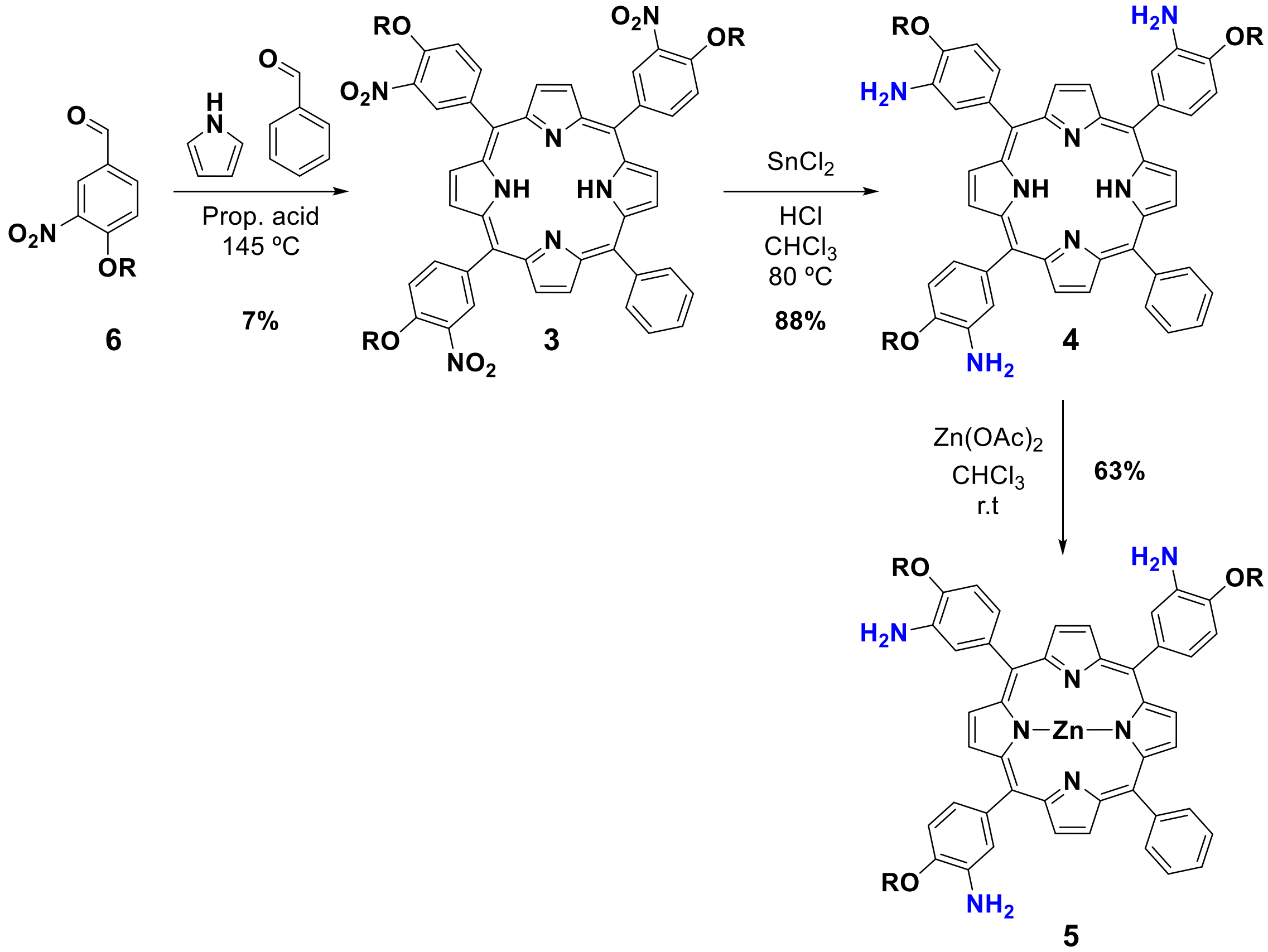

R = OC_6H_{13}

Scheme S4. Synthesis of tri-amino porphyrin **5**.

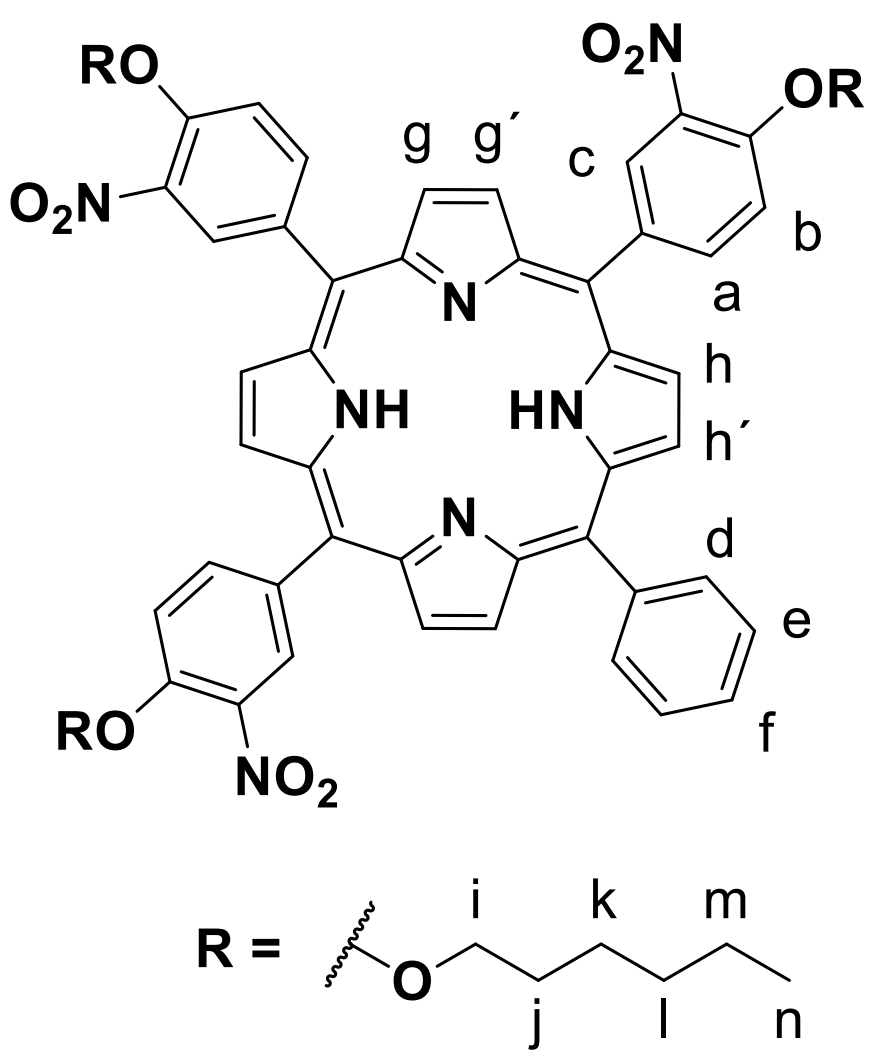

3. Benzaldehyde (0.538 mL, 5.29 mmol, 1 eq) and **6** (4.0 g, 15.88 mmol, 3 eq) were dissolved in propionic acid (67 mL) under inert atmosphere. Upon heating at 145 ºC, fresh distilled pyrrole (1.47 mL, 21.18 mmol, 4 eq) was added and the mixture was stirred at this temperature, in dark, for 3 hours. Then, the heating source was removed, and the mixture stirred at room temperature, overnight. Upon the reaction was completed, the solvent was removed under vacuum and the product was filtrated through silica in 100% $CHCl_3$ and subsequently purified by a chromatography column using a mixture of cyclohexane:$CHCl_3$ (90:10 to 67:33) as eluent, achieving the product **3** as a purple solid (0.389 g, Y = 7%).

^{1}H NMR (300 MHz, $CDCl_3$) δ (ppm) = 8.96 – 8.80 (m, 8H, $H^{g+g'+h+h'}$), 8.68 (d, J = 2.2 Hz, 3H, H^c), 8.29 (d, J = 8.5 Hz, 3H, H^a), 8.22 (d, J = 6.8 Hz, 2H, H^d), 7.79 (m, 3H, H^{e+f}), 7.40 (d, J = 8.5 Hz, 3H, H^b), 4.37 (t, J = 6.3 Hz, 6H, H^i), 2.02 (q, J = 6.3 Hz, 6H, H^j), 1.67 (m, 6H, H^k), 1.52 – 1.42 (m, 12H, H^{l+m}), 1.01 (m, 9H, H^n), –2.82 (s, 2H, **NH**).

^{13}C NMR (75 MHz, $CDCl_3$) δ (ppm) = 152.6, 141.8, 139.2, 138.7, 134.7, 134.0, 131.0 (pyrr), 130.5, 128.2, 127.0, 121.5, 117.6, 117.3, 112.9, 77.4, 70.3, 31.7, 29.2, 25.8, 22.8, 14.2.

HRMS (MALDI, DCTB+PPGNa 1000): Mass m/z calculated for $C_{62}H_{63}N_7O_9$ $[M]^+$ 1049.4682, found: 1049.4673.

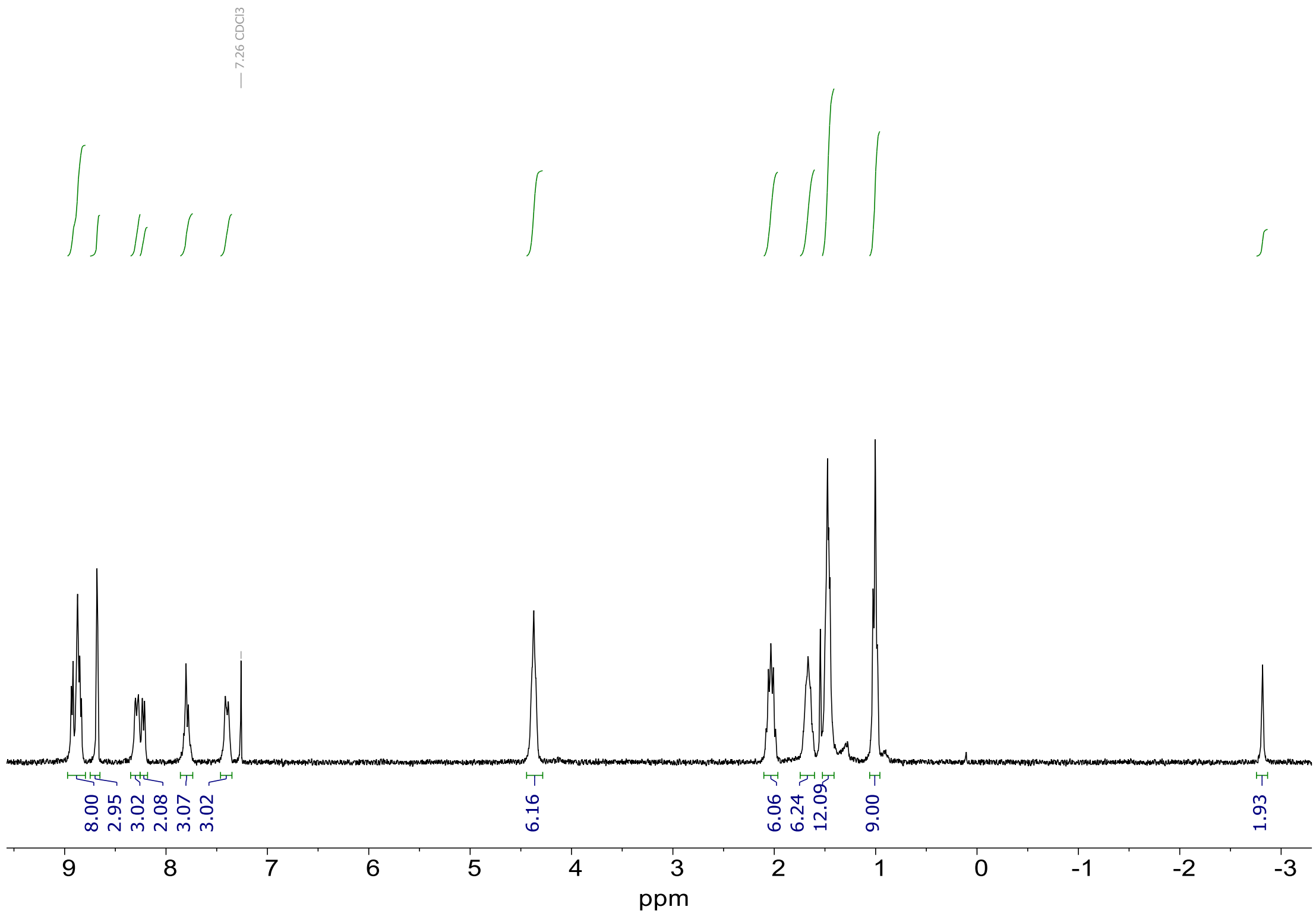

Figure S7. ^{1}H NMR spectrum of compound **3** in $CDCl_3$ at 298 K.

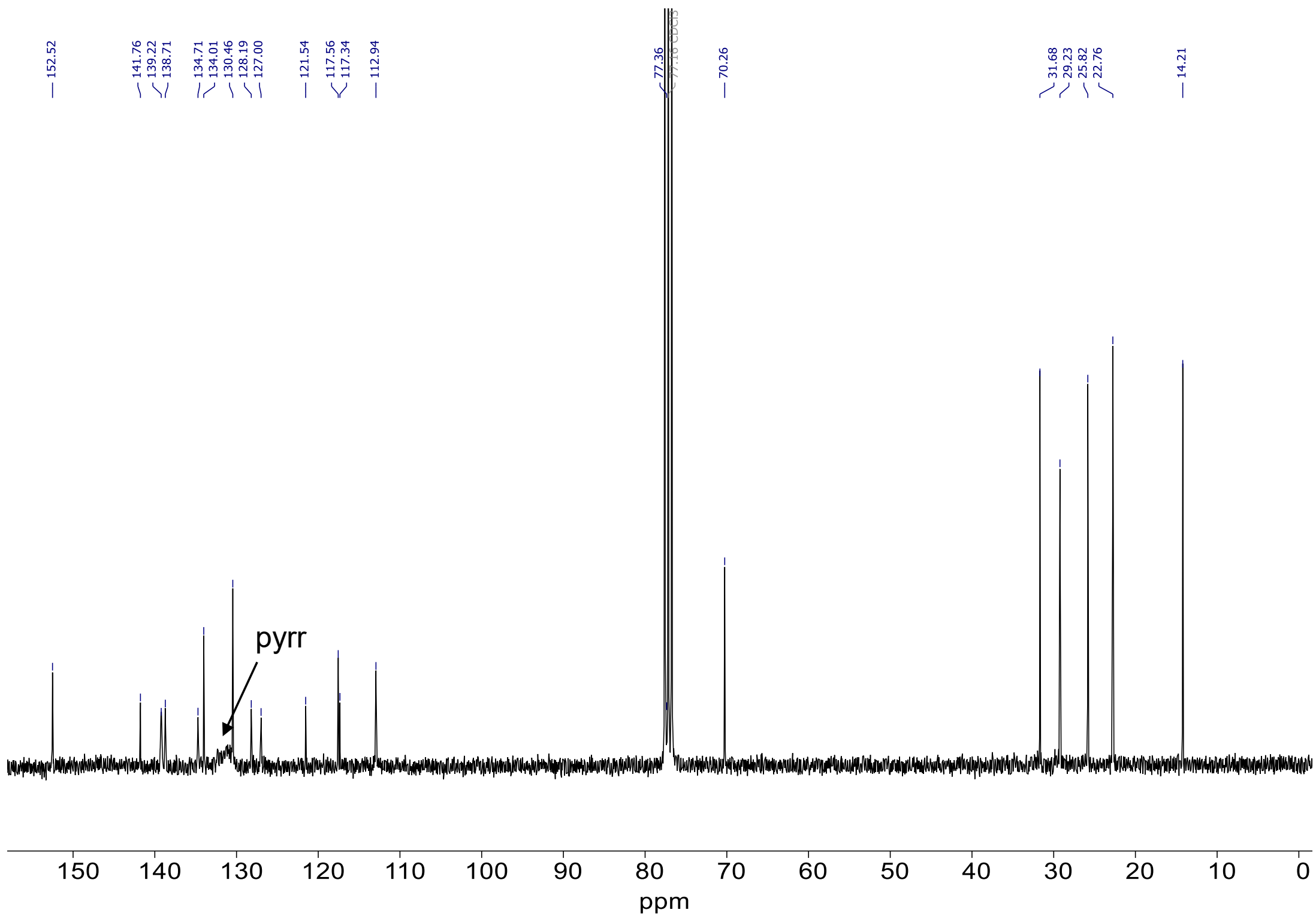

Figure S8. ^{13}C NMR spectrum of compound **3** in $CDCl_3$ at 298 K. The "pyrr" label refers to the pyrrolic carbon atoms of the target porphyrin.

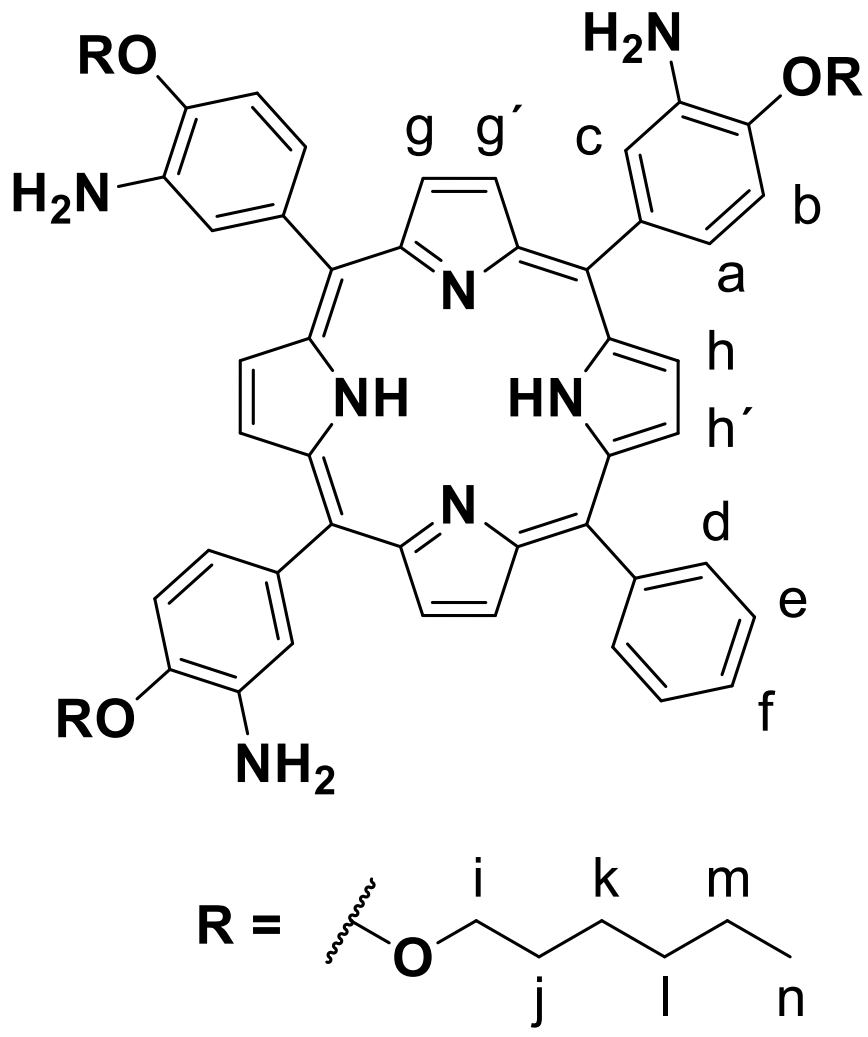

4. Compound **3** (167 mg, 0.16 mmol, 1 eq.) and $SnCl_2$ (482 mg, 2.54 mmol, 16 eq.) were dissolved in $CHCl_3$ (43 mL) under inert atmosphere. Commercial HCl (2.38 mL, 37%) was injected, and the mixture was heated at reflux, in dark, overnight. Upon this time, the reaction crude was washed several times with aqueous solution of NaOH (2M) until the organic layer colour changed from green to red-purple, and then the organic layer was dried with Na_2SO_4, the solid was filtered, and the solvent removed under vacuum. The resulting solid was purified by silica chromatography column using a mixture of cyclohexane:EtOAc (90:10 to 67:33) as eluent, achieving the desired product **4** as a purple solid (130 mg, Y = 85%).

^{1}H NMR (300 MHz, $CDCl_3$) *δ* (ppm) = 9.02 (s, 6H, $H^{g+g'+h}$), 8.91 (d, *J* = 4.3 Hz, 2H, $H^{h'}$), 8.30 (d, *J* = 7.2 Hz, 2H, H^d), 7.83 – 7.75 (m, 3H, H^{e+f}), 7.66 (d, *J* = 2.1 Hz, 3H, H^c), 7.55 (d, *J* = 8.1, 2.1 Hz, 3H, H^a), 7.04 (d, *J* = 8.1 Hz, 3H, H^b), 4.17 (t, *J* = 6.7 Hz, 6H, H^i), 4.06 (s, 6H, **NH_2**), 2.05 – 1.94 (m, 6H, H^j), 1.68 – 1.57 (m, 6H, H^k), 1.56 – 1.41 (m, 12H, H^{l-m}),1.03 – 0.98 (m, 9H, H^n), –2.60 (s, 2H, **NH**).

^{13}C NMR (75 MHz, $CDCl_3$) *δ* (ppm) = 146.7, 142.6, 135.0 (x2), 134.7, 134.3, 131.1 (pyrr), 127.7, 126.7, 125.9, 122.1, 120.5, 120.4, 119.6, 109.5, 68.6, 31.8, 29.6, 26.1, 22.8, 14.3.

HRMS (MALDI, DCTB): Mass (*m/z*) calculated for $C_{62}H_{69}N_7O_3$ $[M]^+$: 959.5456; found: 959.5443.

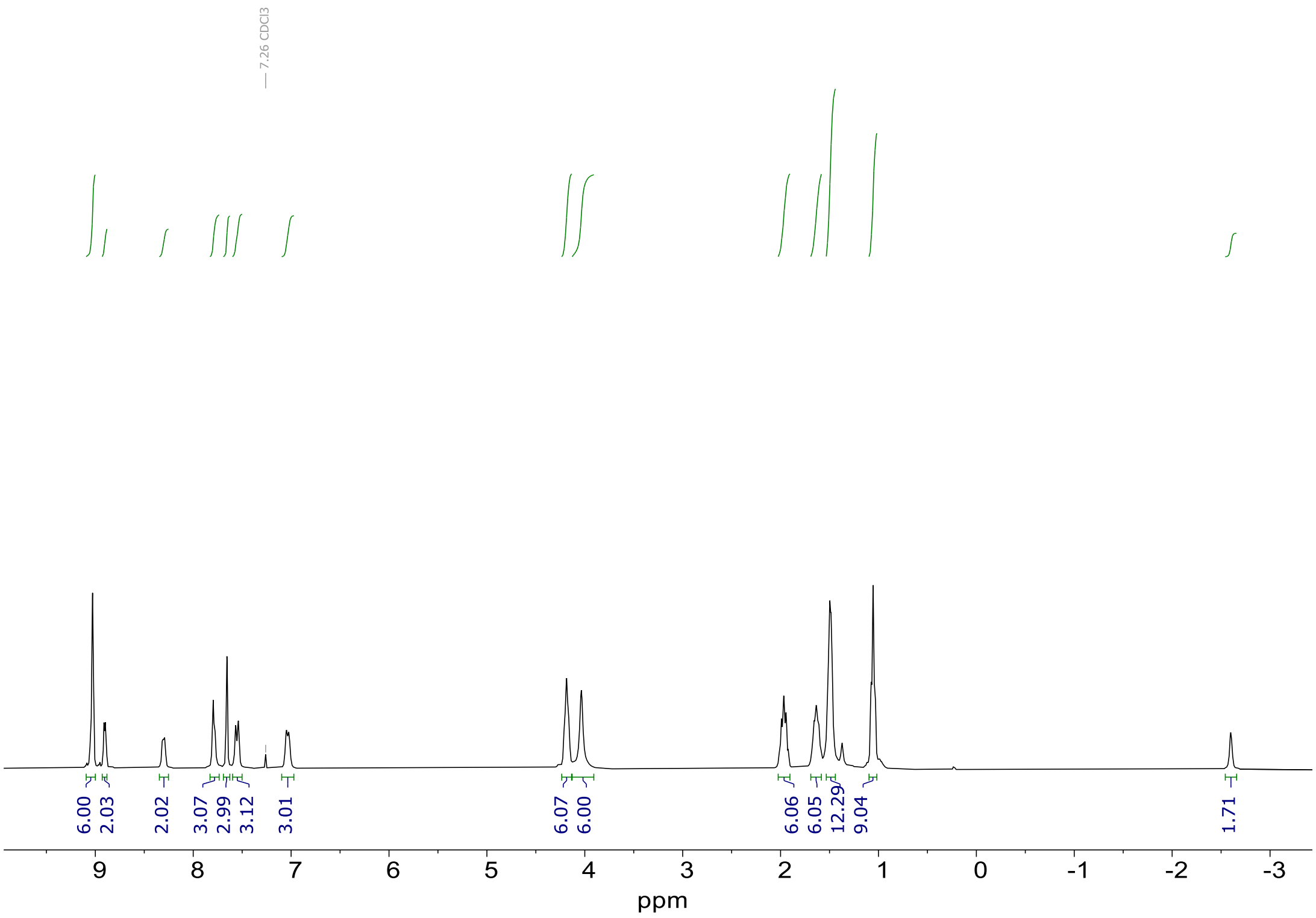

Figure S9. ^{1}H NMR spectrum of compound **4** in $CDCl_3$ at 298 K.

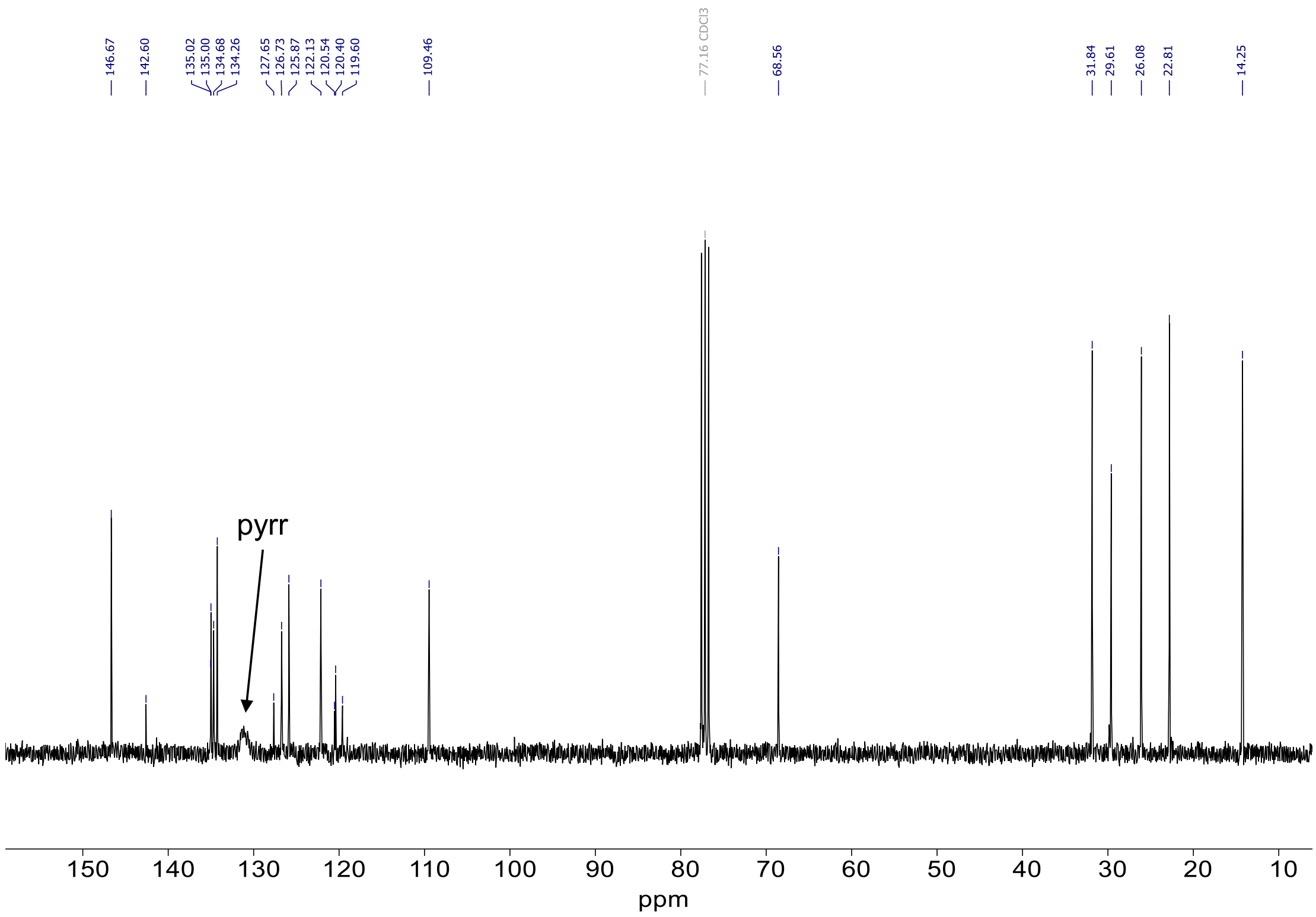

Figure S10. ^{13}C NMR spectrum of compound **4** in $CDCl_3$ at 298 K. The “pyrr” label refers to the pyrrolic carbon atoms of the target porphyrin.

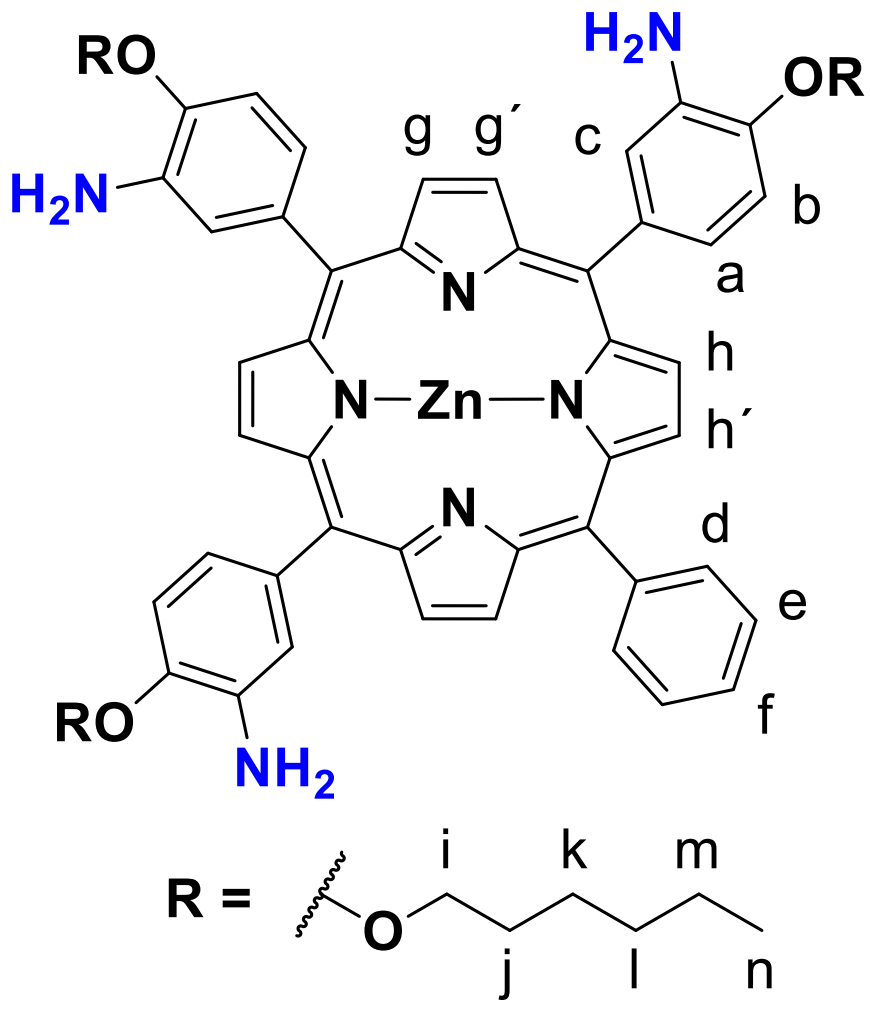

5. To a solution of **4** (62 mg, 0.064 mmol, 1 eq) in $CHCl_3$ (10 mL) $Zn(OAc)_2$ (35.6 mg, 0.194 mmol, 3.0 eq) was added and stirred under inert atmosphere during 6 hours at room temperature. The compound was purified by silica chromatography column using a mixture of cyclohexane:EtOAc (88:12 to 67:33) as eluent, achieving the desired product **5** as a green solid (41 mg, Y = 63%).

^{1}H NMR (300 MHz, THF-d_8) δ (ppm) = 9.00 – 8.90 (m, 6H, $H^{g+g'+h}$), 8.75 (d, J = 4.4 Hz, 2H, $H^{h'}$), 8.18 (d, J = 7.1 Hz, 2H, H^d), 7.75 – 7.67 (m, 3H, H^{e+f}), 7.51 (s, 3H, H^c), 7.37 (d, J = 7.8 Hz, 3H, H^b), 7.11 (dd, J = 7.8, 1.8 Hz, 3H, H^a), 4.54 (s, 6H, $\mathbf{NH_2}$), 4.26 (t, J = 6.3 Hz, 6H, H^i), 2.09 – 1.94 (m, 6H, H^j), 1.70 – 1.61 (m, 6H, H^k), 1.59 – 1.41 (m, 12H, H^{l-m}), 1.06 – 0.95 (m, 9H, H^n).

^{13}C NMR (75 MHz, THF-d_8) δ (ppm) = 151.6, 151.5, 151.4, 150.8, 147.1, 145.2, 137.4, 137.3, 136.6, 135.5, 132.6, 132.3 (x2), 131.5, 128.0, 127.2, 125.1 (x2), 122.6 (x2), 122.4, 122.2, 120.6, 109.9, 69.3, 68.1, 33.0, 30.8, 27.2, 26.0, 23.8, 16.7.

HRMS (MALDI, DCTB+PPGNa 1000): Mass (m/z) calculated for $C_{62}H_{67}N_7O_3Zn$ $[M]^+$: 1021.4591; found: 1021.4555.

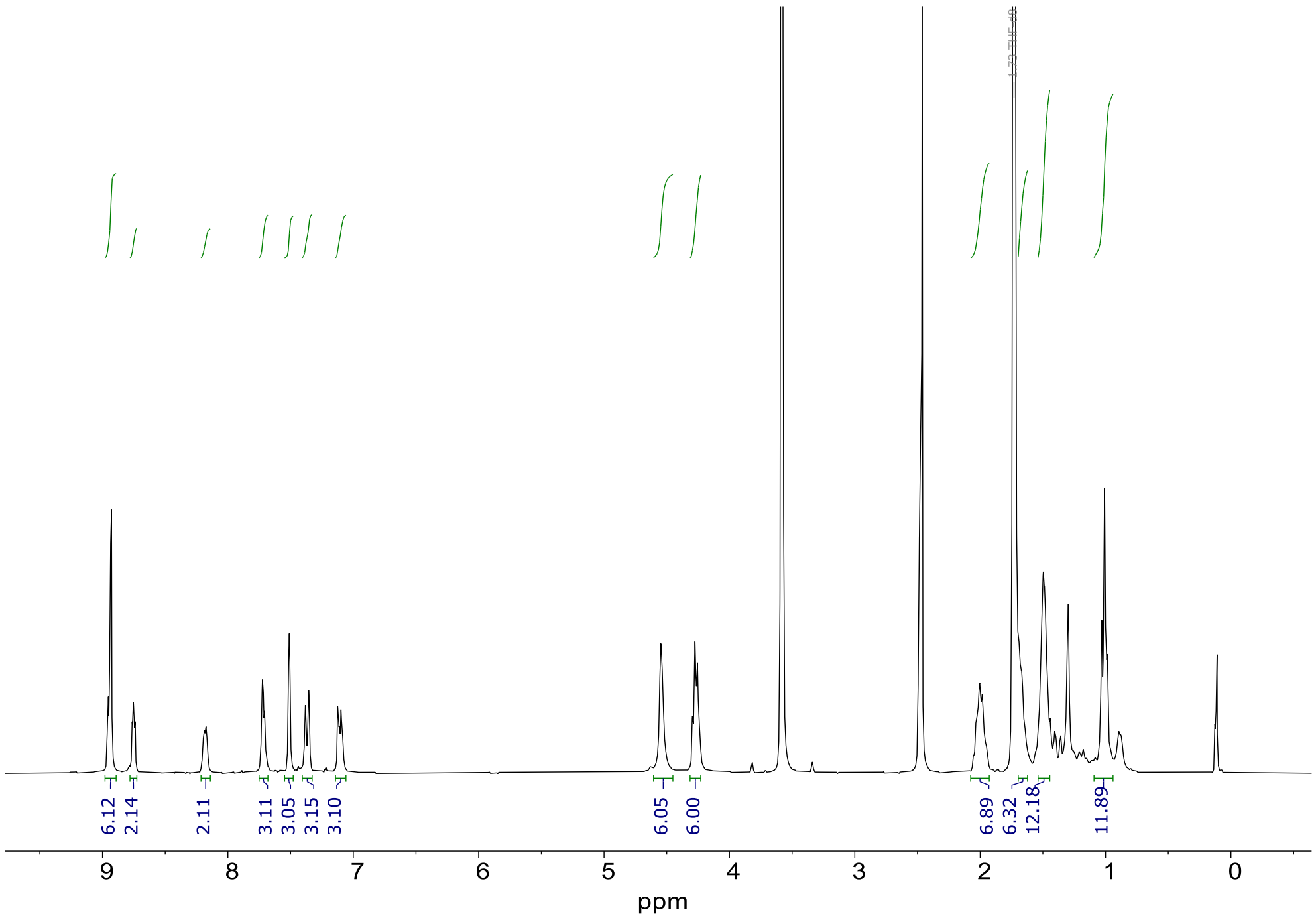

Figure S11. ^{1}H NMR spectrum of compound **5** in THF-d$_8$ at 298 K.

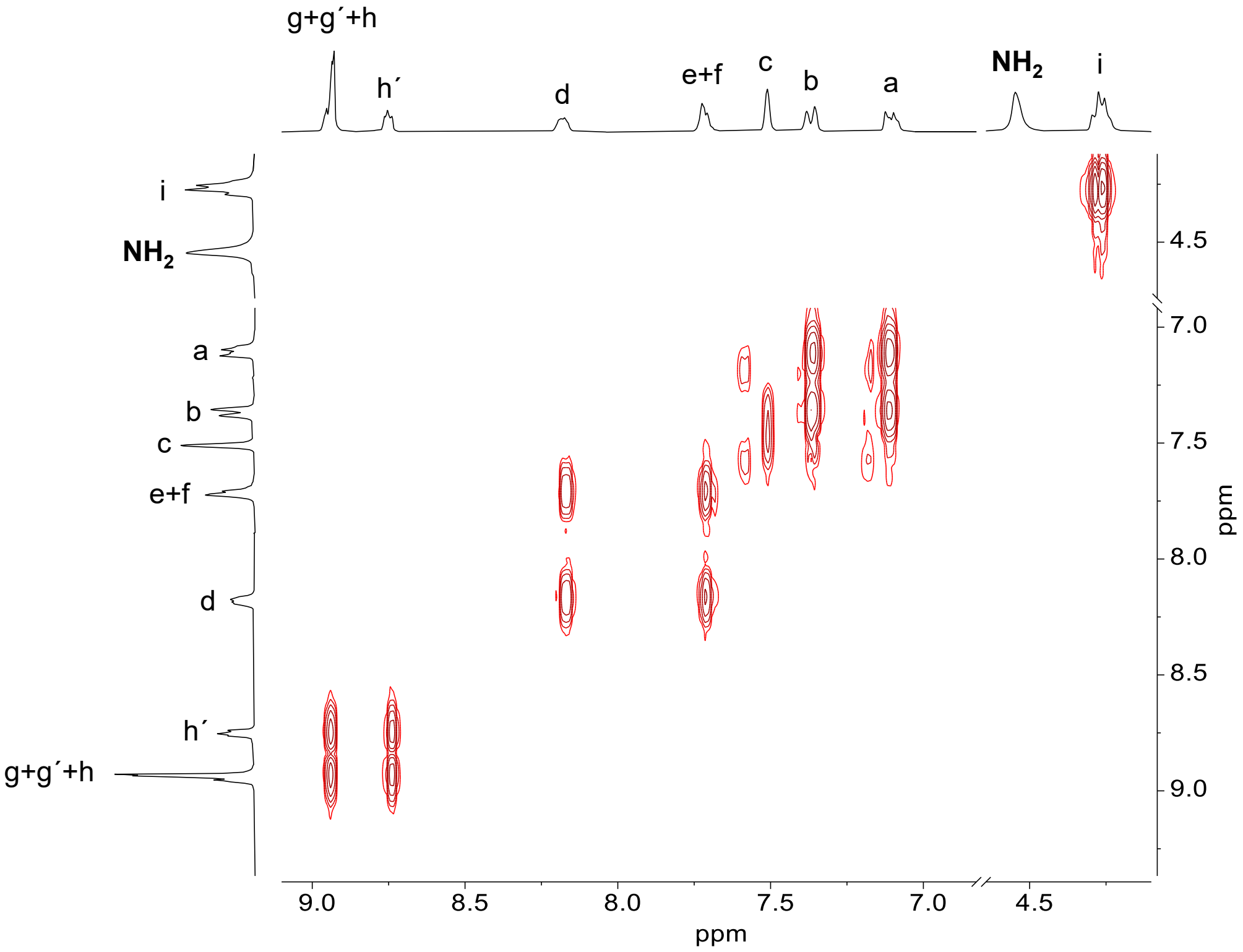

Figure S12. ^{1}H-^{1}H COSY NMR spectrum of **5** in THF-d$_8$ at 298 K.

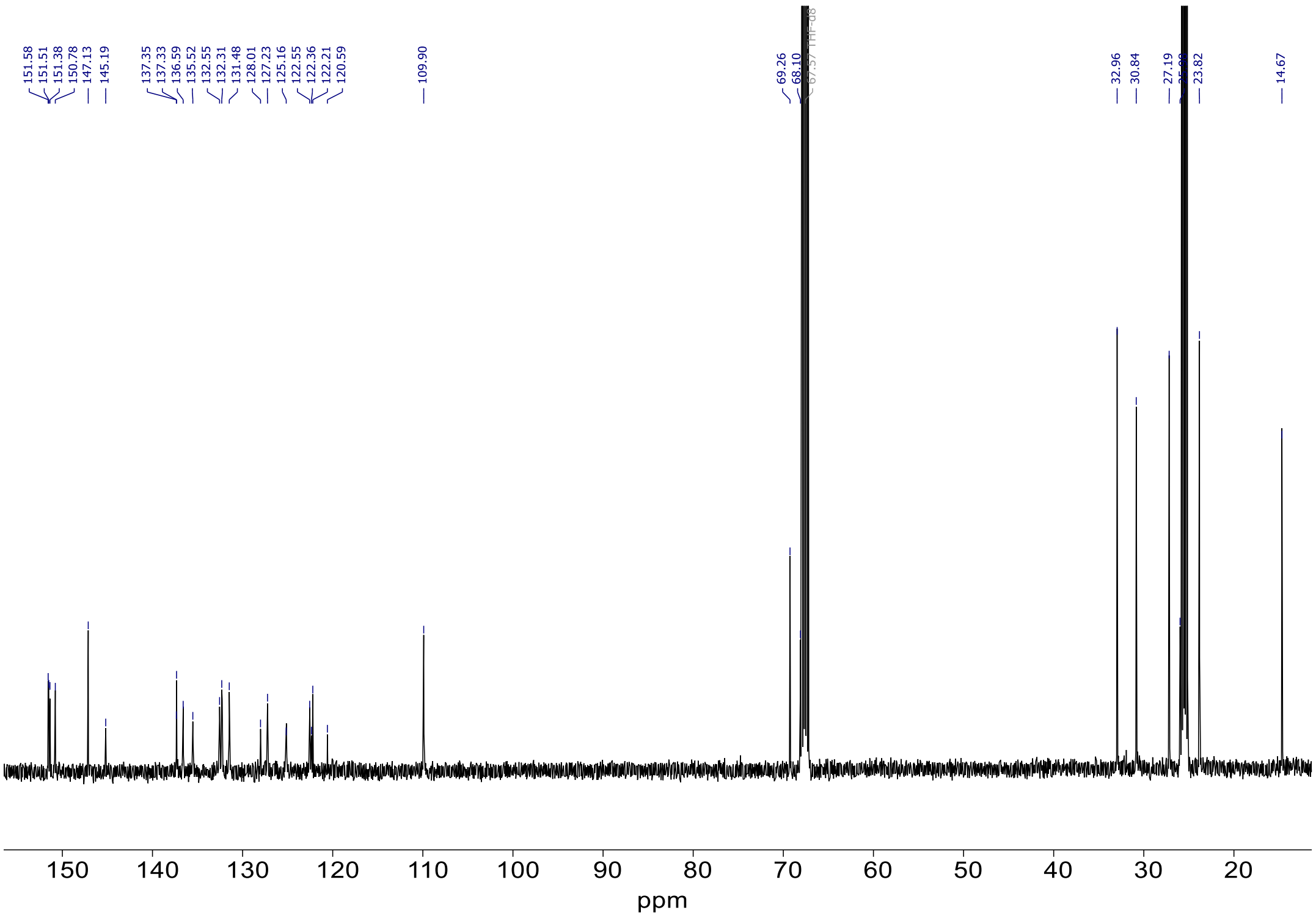

Figure S13. ^{13}C NMR spectrum of compound **5** in THF-d_8 at 298 K.

Synthesis of *basket* 3_{Zn}.

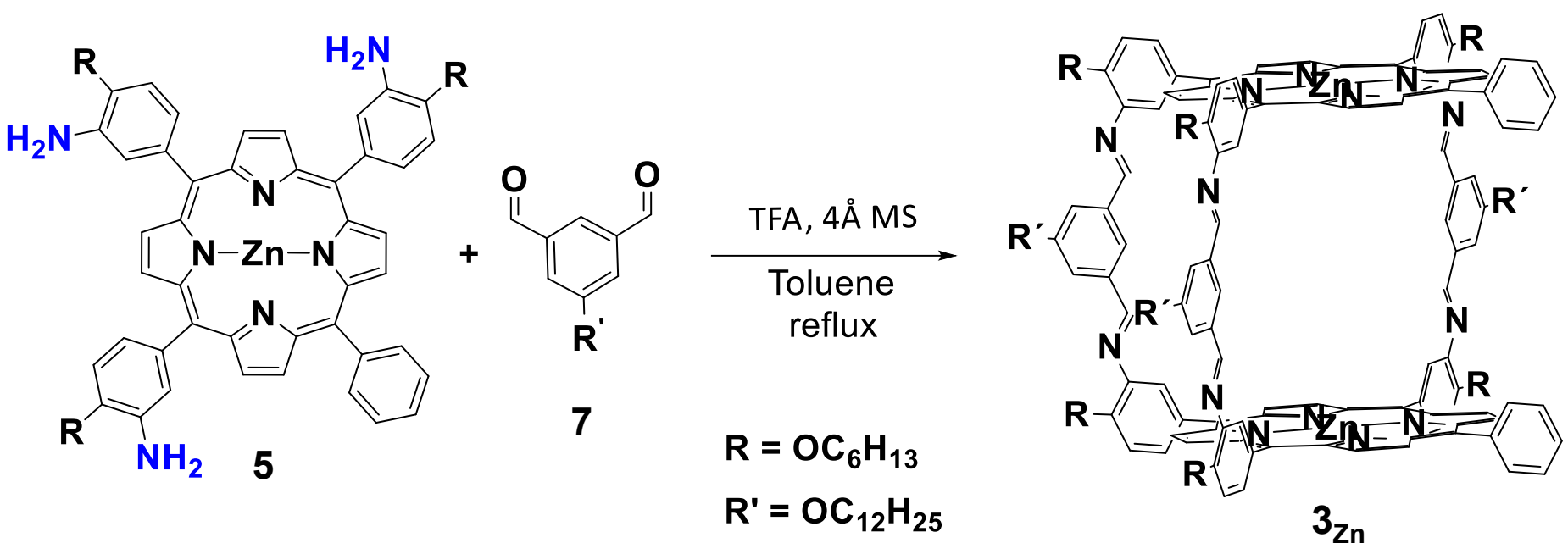

Scheme S5. Synthesis of the molecular *basket* $\mathbf{3_{Zn}}$.

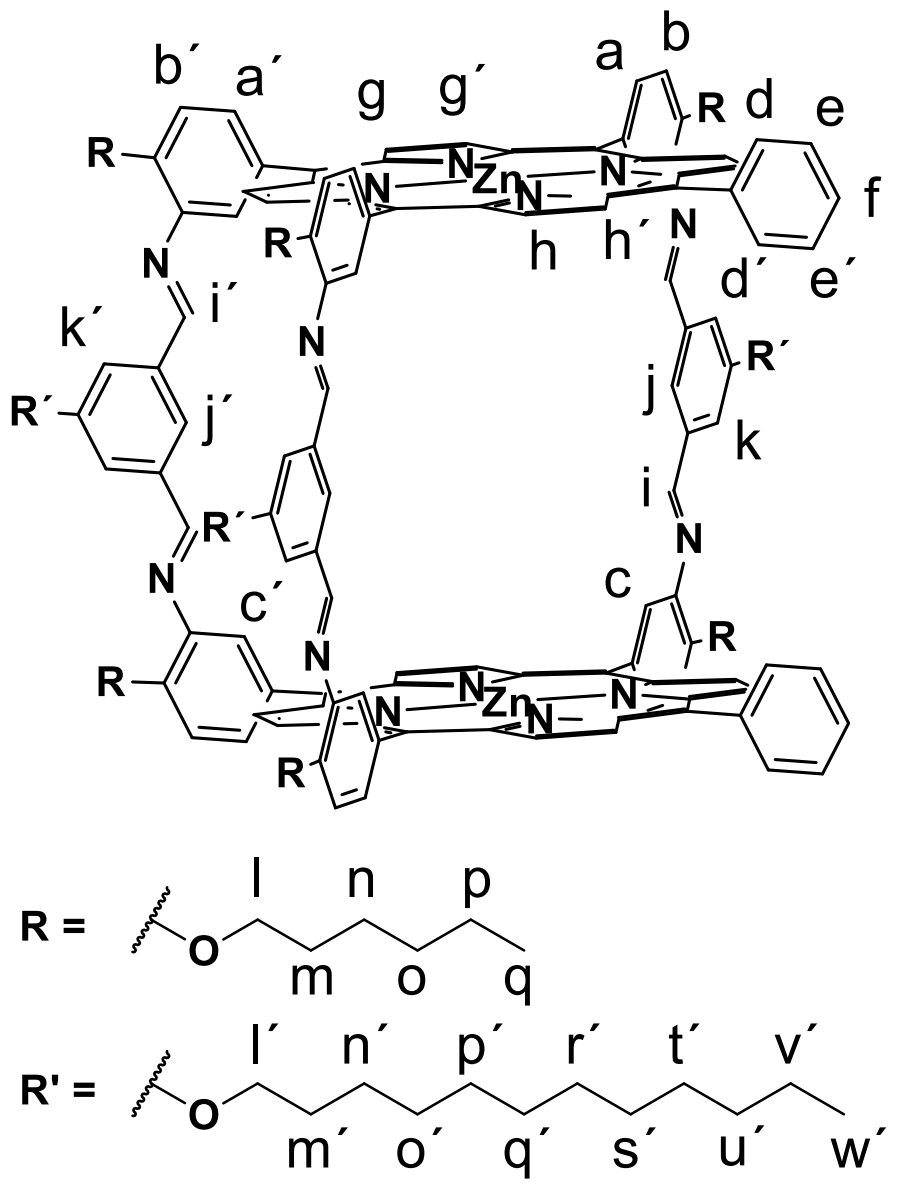

3_{Zn}. To a solution of porphyrin **5** (35.18 mg, 0.034 mmol, 1 eq) and linker **7** (16.42 mg, 0.052 mmol, 1.5 eq) in toluene (8.6 mL), TFA (0.017 mmol, 0.5 eq) was added under inert atmosphere. The mixture was heated at reflux overnight. Then, TEA (0.034 mmol, 1.0 eq) was added to neutralize the reaction. Afterwards, MeCN was added, helping to crash out a purple solid, the suspension was filtered, and the solid rinsed with MeCN, obtaining the desired compound as a purple solid (14.0 mg, 28%).

^{1}H NMR (300 MHz, toluene-d$_8$) δ (ppm) = 9.11 (td, J = 6.7, 1.9 Hz, 8H, $H^{g,g´}$), 9.02 (d, J = 4.6 Hz, 4H, H^{h}), 8.84 (d, J = 4.6 Hz, 4H, $H^{h´}$), 8.52 (s, 2H, $H^{i´}$), 8.45 (s, 4H, H^{i}), 8.12 – 8.00 (m, 12H, $H^{a,\ c´,\ k,\ d\ or\ d´}$), 8.00 – 7.93 (m, 6H, $H^{a´,\ k´,\ d\ or\ d´}$), 7.90 (d, J = 2.2 Hz, 4H, H^{c}), 7.48 (s, 1H, $H^{j´}$), 7.43 (m, 4H, $H^{e,e´}$), 7.35 (m, 2H, H^{f}), 7.31 (s, 2H, H^{j}), 4.05 (m, 12H, H^{l}), 3.88 (m, 6H, $H^{l´}$), 1.87 – 1.77 (m, 12H, H^{m}), 1.75 – 1.68 (m, 6H, $H^{m´}$), 1.61 – 1.52 (m, 12H, H^{n}), 1.43 – 1.22 (m, 78H, $H^{o,p,\ n´-v´}$), 1.00 – 0.94 (m, 27H, $H^{q,w´}$).

^{13}C NMR (75 MHz, toluene-d$_8$) δ (ppm) = 161.0, 160.5 (x2), 152.3, 152.1, 150.7, 150.6, 150.3, 143.69, 140.2, 140.1, 139.0, 137.2, 136.6, 134.6, 132.7, 132.6, 132.0, 131.9, 127.5, 127.4, 126.6 (x2), 124.7, 120.9, 120.4 (x2), 116.5, 116.3, 111.9, 69.4 (x2), 68.3, 32.5, 32.2, 32.1, 30.3 (x3), 30.1 (x2), 30.0 (x3), 29.9, 26.6, 26.5 (x2), 23.2 (x2), 14.4 (x2).

HRMS (MALDI, DCTB+PPGNa 2700 matrix): Mass m/z calculated for $C_{184}H_{212}N_{14}O_9Zn_2$ $[M+H]^+$ 2889.5139, found: 2889.5183.

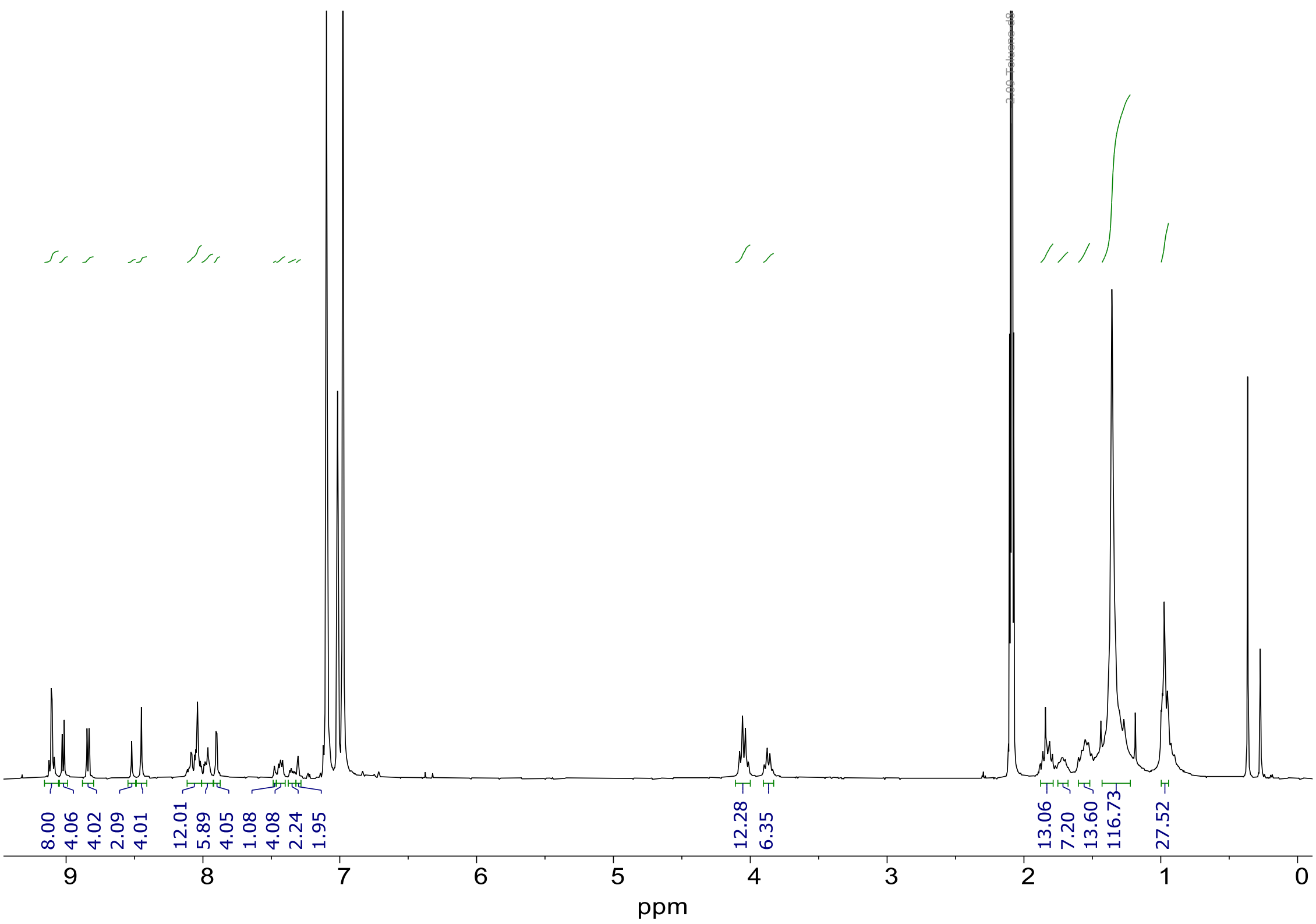

Figure S14. ^{1}H NMR spectrum of molecular $\mathbf{3_{Zn}}$ in toluene-d_8 at 298 K.

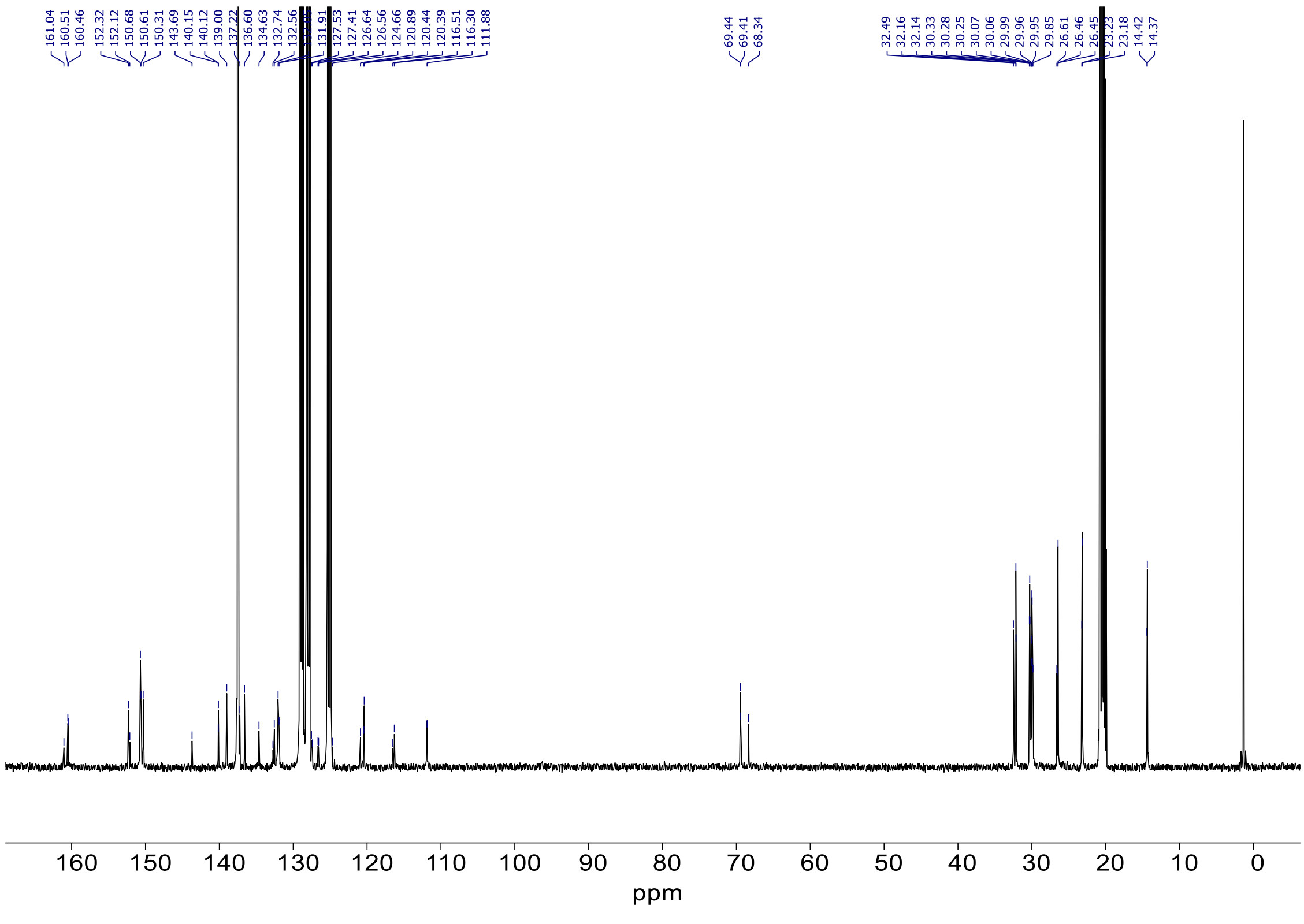

Figure S15. ^{13}C NMR spectrum of compound $\mathbf{3_{Zn}}$ in toluene-d_8 at 298 K.

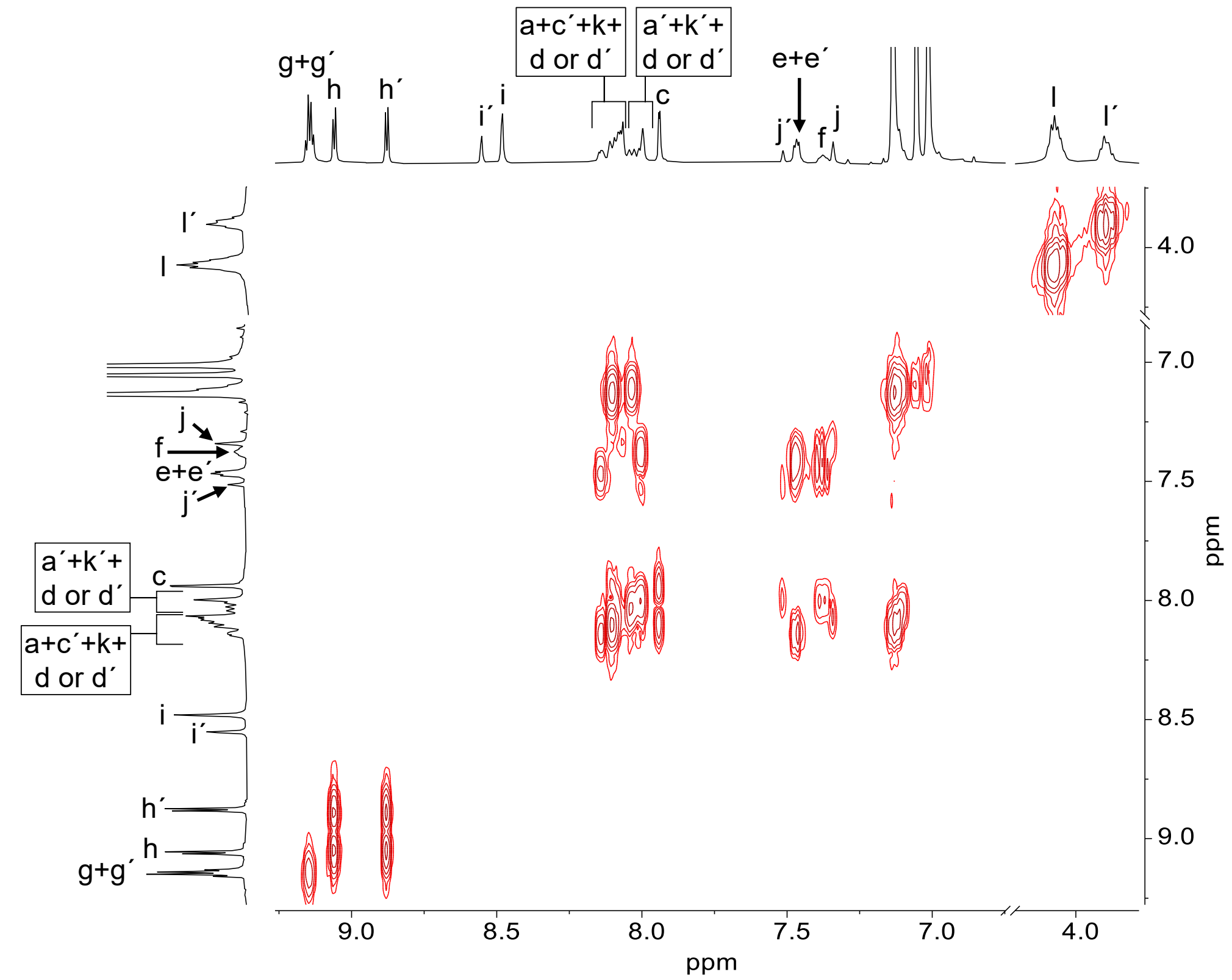

Figure S16. ^{1}H-^{1}H COSY NMR spectrum of $\mathbf{3}_{Zn}$ in toluene-d_8 at 298 K.

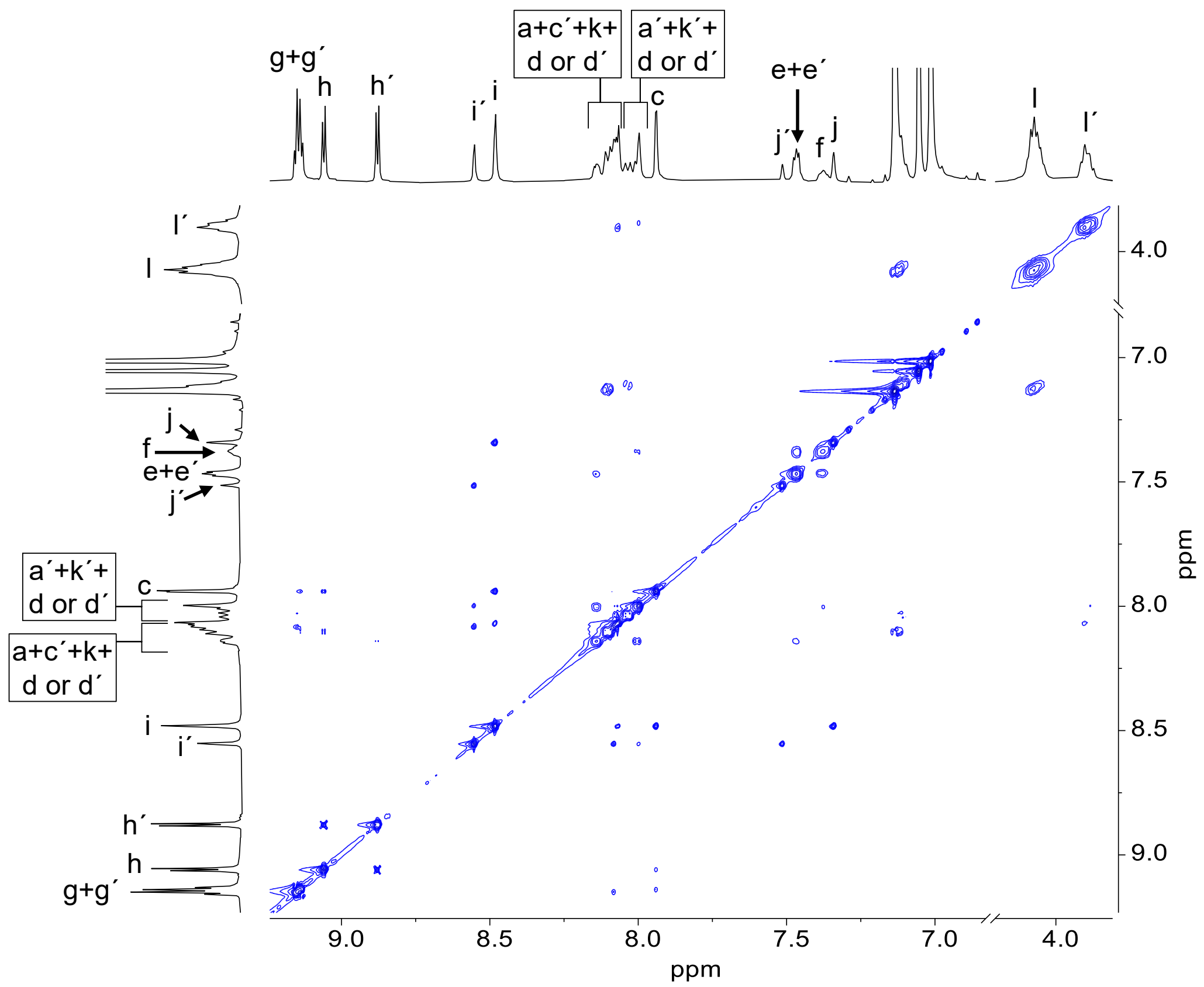

Figure S17. 2D ^{1}H-^{1}H NOESY NMR spectrum of $\mathbf{3}_{Zn}$ in toluene-d_8 at 298 K.

Synthesis of cage 1'$_{Zn}$.

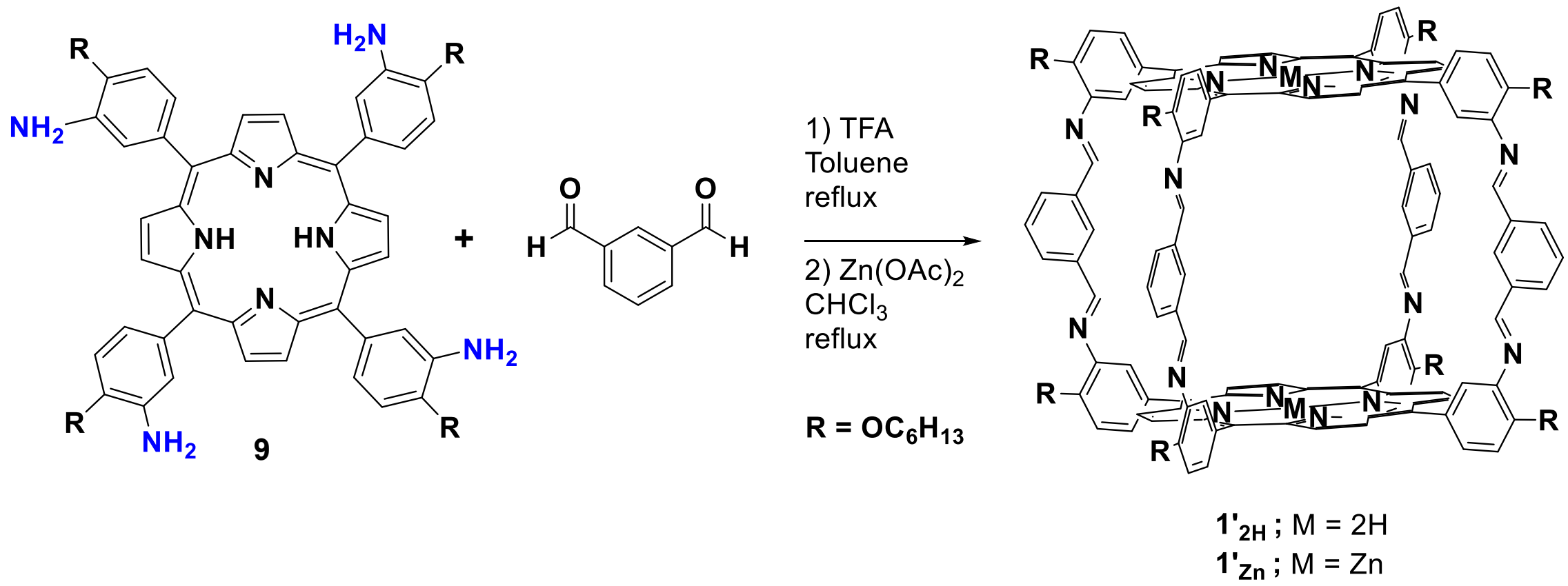

Scheme S6. Synthesis of the porphyrin cage **1'$_{Zn}$**.

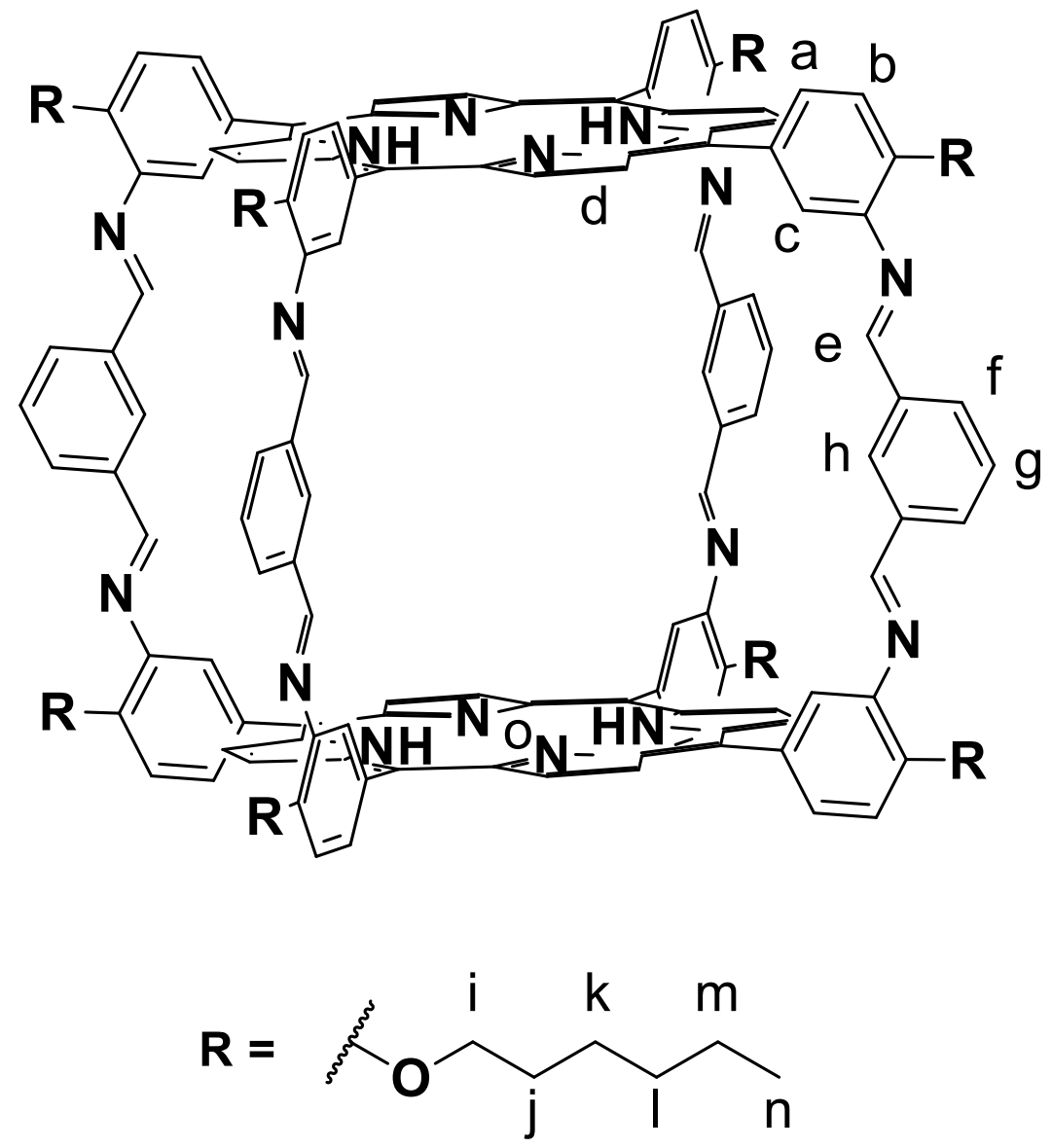

1'$_{2H}$. To a solution of porphyrin **9** (73.33 mg, 0.068 mmol, 1 eq) and commercial isophthalaldehyde linker (18.29 mg, 0.136 mmol, 2 eq) in toluene (17 mL), TFA (0.068 mmol, 1 eq) was added under inert atmosphere. The mixture was heated at reflux, overnight. Then, TEA (0.102 mmol, 1.5 eq) was added to neutralize the reaction. Then, MeOH was added, helping to crash out a purple solid, the suspension was filtered, and the solid rinsed with MeOH, obtaining the desired compound as a purple solid (72.00 mg, 83 %).

^{1}H NMR (300 MHz, $CDCl_3$) δ (ppm) = 8.78 (s, 16H, H^d), 8.34 (s, 8H, H^e), 8.31 (dd, J = 1.7, 7.9 Hz, 8H, H^f), 8.01 (dd, J = 8.2, 2.2 Hz, 8H, H^a), 7.59 (t, J = 7.9 Hz, 4H, H^g), 7.54 (d, J = 2.2 Hz, 8H, H^c), 7.37 (t, J = 1.7 Hz, 4H, H^h), 7.25 (d, J = 8.2 Hz, 8H, H^b), 4.30 (t, J = 6.5 Hz, 16H, H^i), 2.01 (q, J = 6.5 Hz, 16H, H^j), 1.69 – 1.57 (m, 16H, H^k), 1.53 – 1.37 (m, 32H, H^{l+m}), 0.96 (t, J = 6.9 Hz, 24H, H^n), -3.03 (s, 4H, H^o).

^{13}C NMR (75 MHz, $CDCl_3$) δ (ppm) = 160.1, 152.2, 139.5, 136.8, 135.1, 132.5, 132.4, 129.9, 129.4, 126.9, 119.4, 111.6, 69.7, 31.9, 29.6, 26.0, 22.8, 14.2.

HRMS (MALDI, DCTB + **PEGNa 2000 + PEGNa 3400):** Mass (*m/z*) calculated for $C_{168}H_{172}N_{16}O_8$ $[M]^+$: 2451.3500; found: 2541.3539.

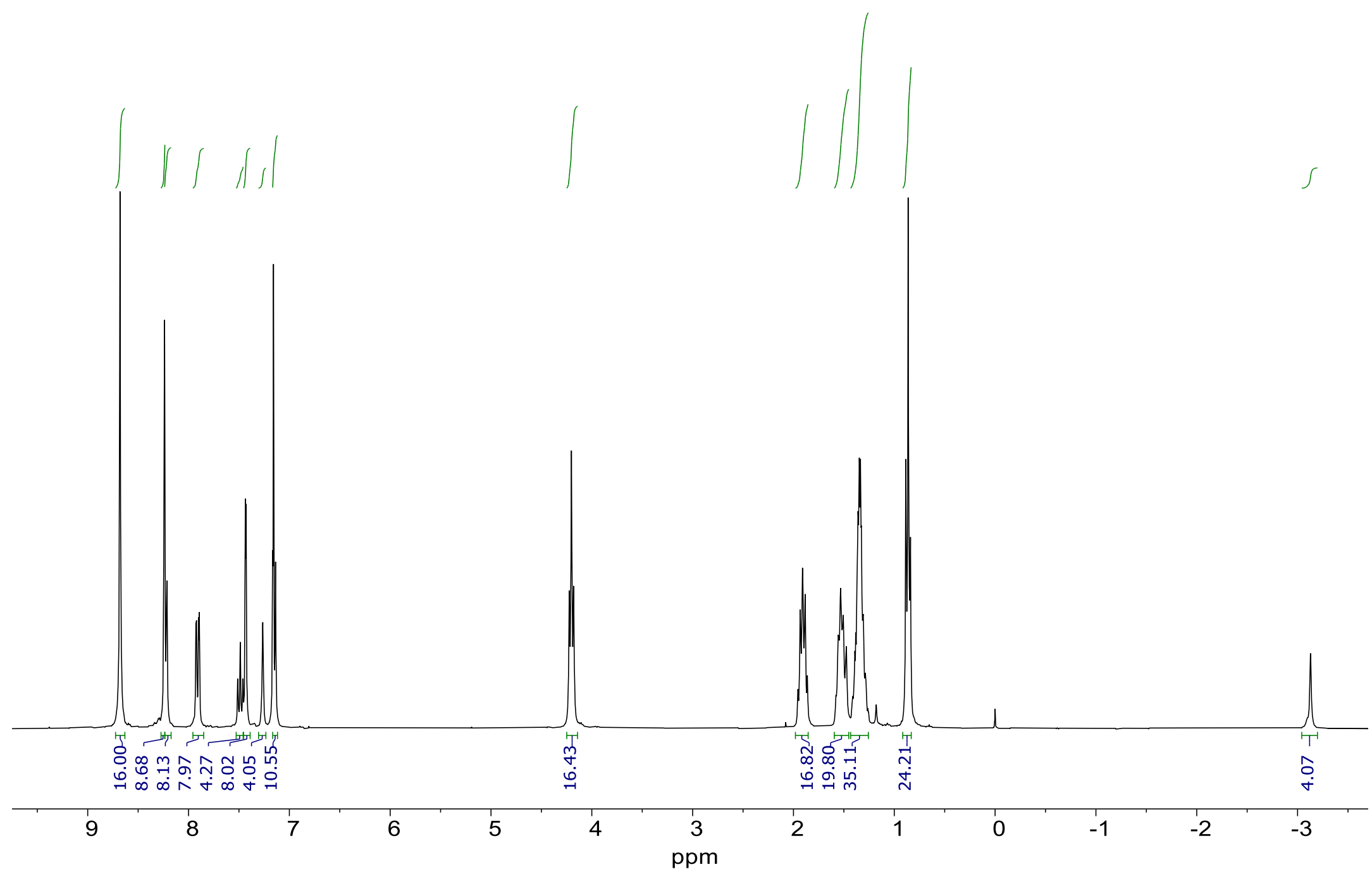

Figure S18. ^{1}H NMR spectrum of cage **1'$_{2H}$** in $CDCl_3$ at 298 K.

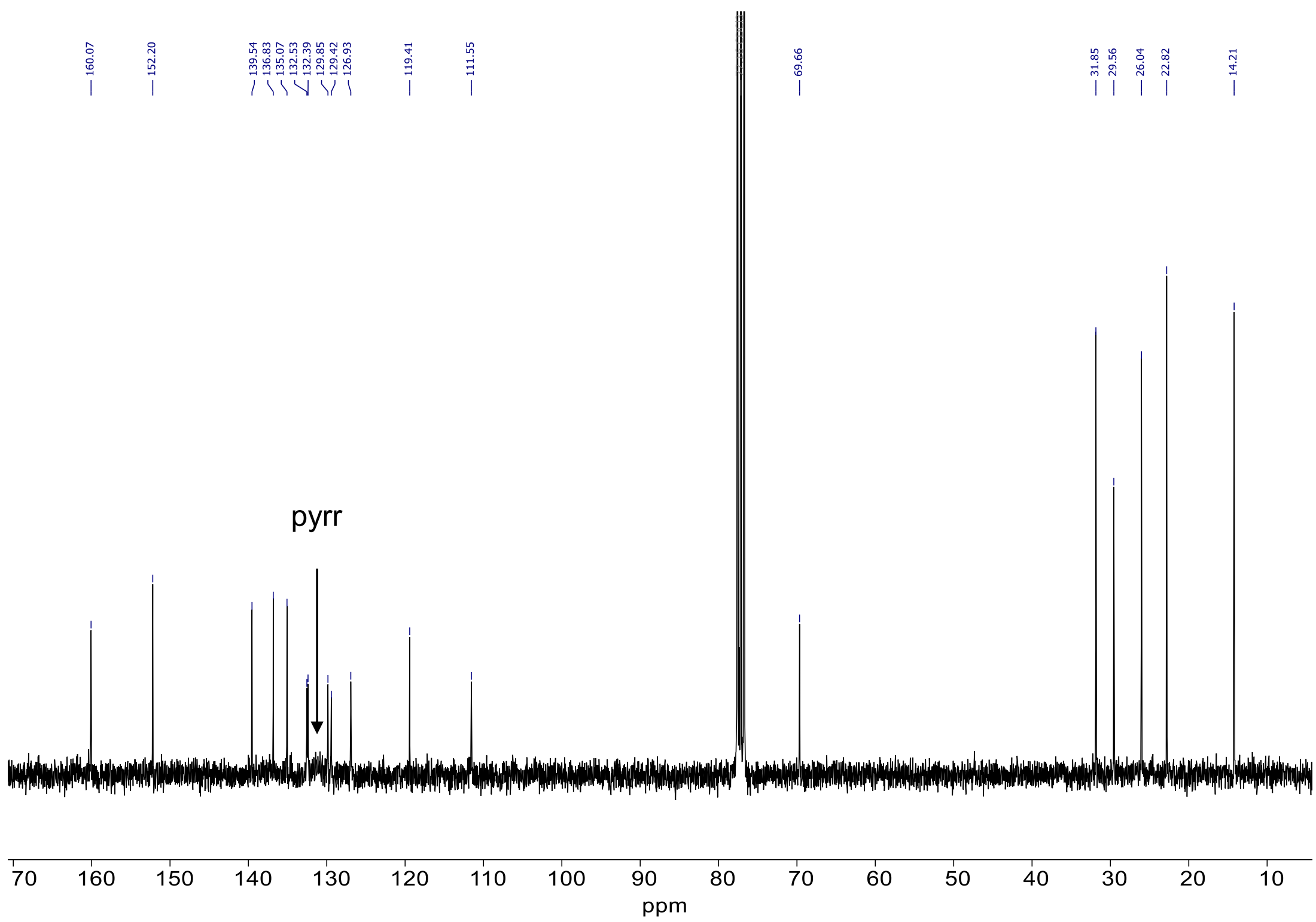

Figure S19. ^{13}C NMR spectrum of cage **1'$_{2H}$** in $CDCl_3$ at 298 K. The "pyrr" label refers to the pyrrolic carbon atoms of the target porphyrin.

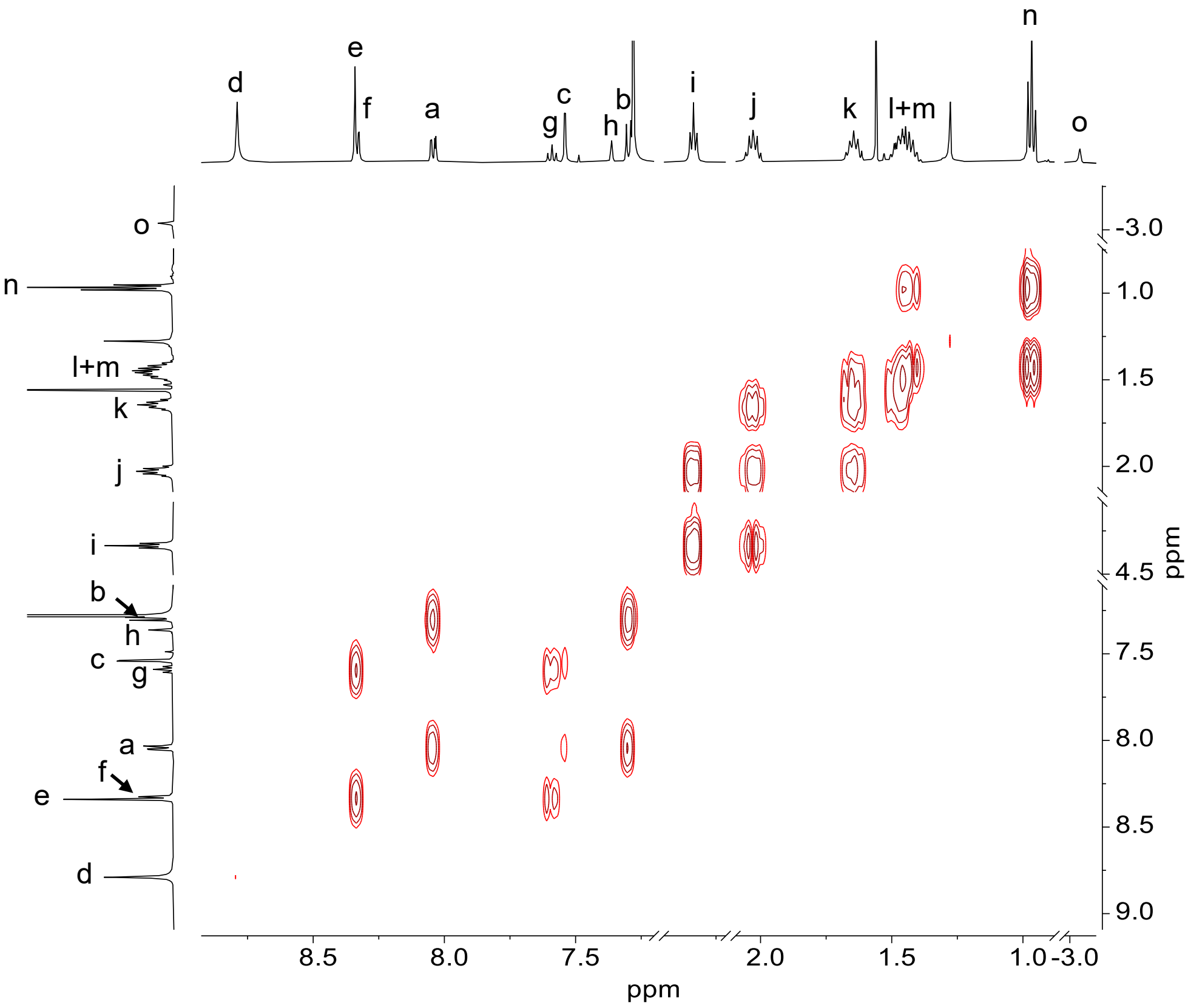

Figure S20. ^{1}H-^{1}H COSY NMR spectrum of cage **1'$_{2H}$** in $CDCl_3$ at 298 K.

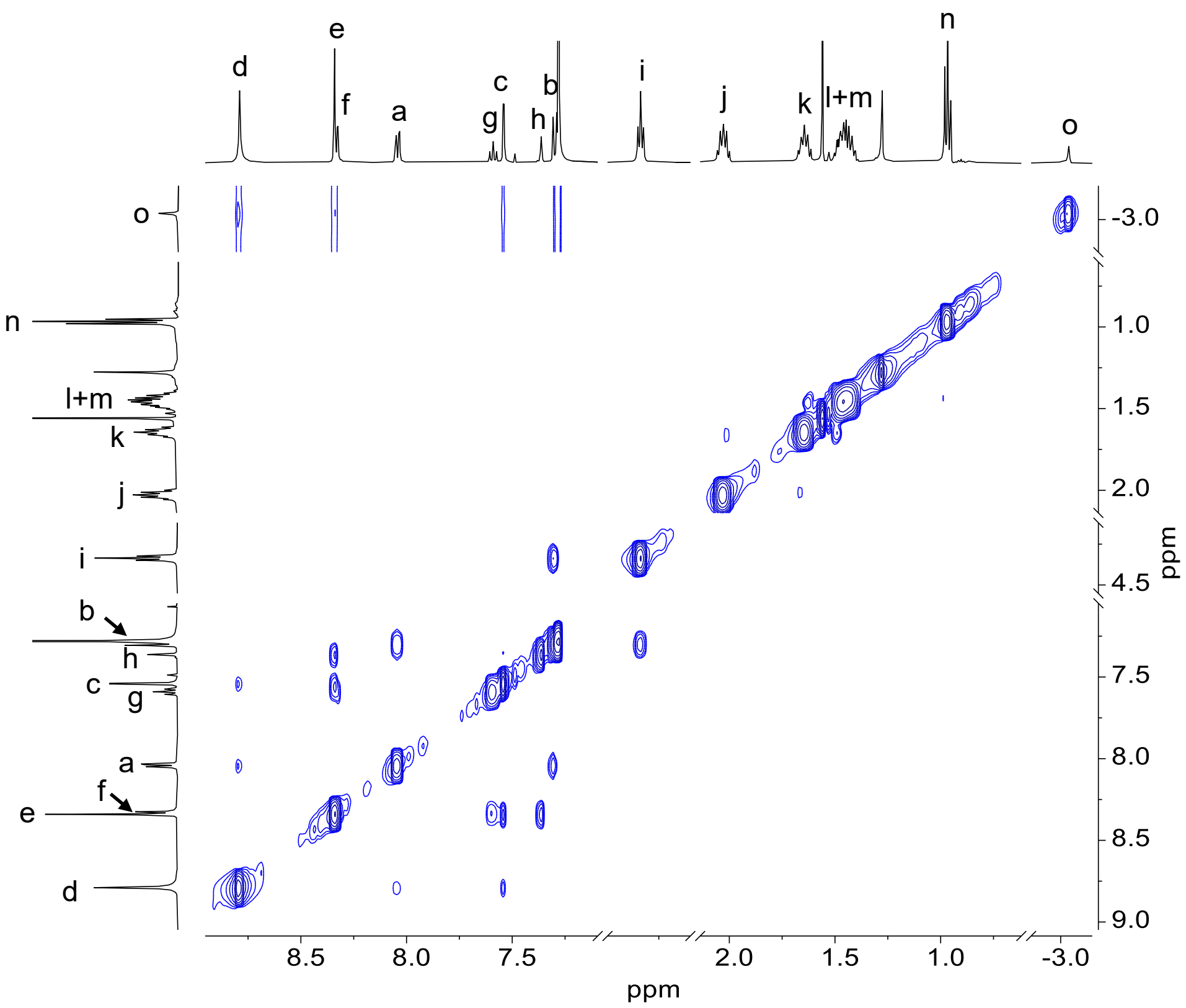

Figure S21. 2D ^{1}H-^{1}H NOESY NMR spectrum of cage **1'$_{2H}$** in $CDCl_3$ at 298 K.

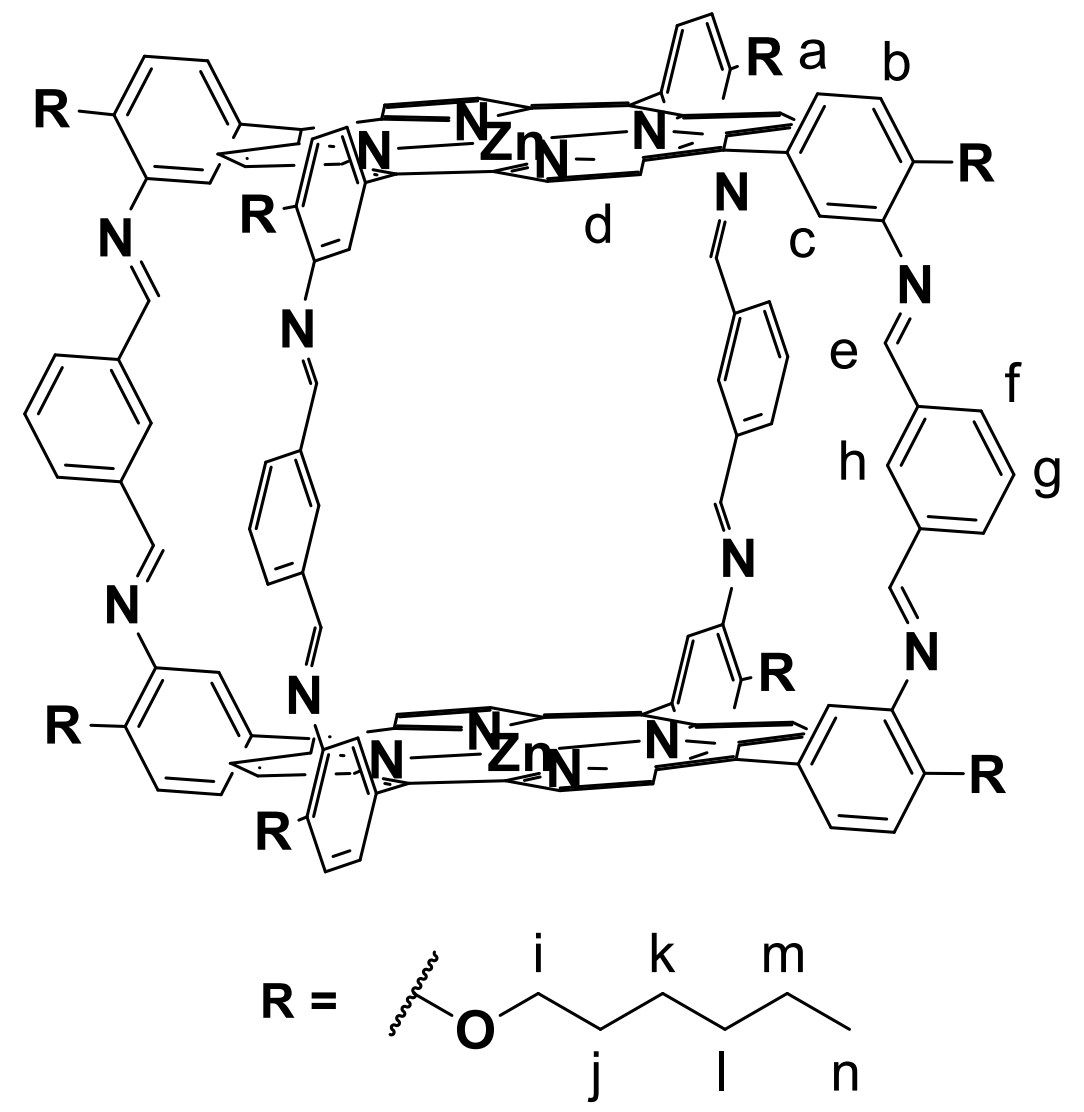

1’$_{Zn}$. To a solution **1’$_{2H}$** (56.40 mg, 0.022 mmol, 1 eq) in $CHCl_3$ (22 mL) $Zn(OAc)_2$ (8.95 mg, 0.049 mmol, 2.2 eq) was added and heated at reflux under inert atmosphere during XX hours. Then, MeOH was added, helping to crash out a purple solid, the suspension was filtered, and the solid rinsed with MeOH, obtaining the desired compound as a purple solid (57.30 mg, 98 %).

^{1}H NMR (300 MHz, $CDCl_3$) δ (ppm) = 8.98 (s, 16H, H^d), 8.34 (dd, J = 1.5, 7.9 Hz, 8H, H^f), 8.28 (s, 8H, H^e), 8.03 (dd, J = 2.2, 8.2 Hz, 8H, H^a), 7.56 (t, J = 7.9 Hz, 4H, H^g), 7.52 (d, J = 2.2 Hz, 8H, H^c), 7.28 (d, J = 8.2 Hz, 8H, H^b), 7.21 (t, J = 1.5 Hz, 4H, H^h), 4.32 (t, J = 6.6 Hz, 16H, H^i), 2.01 (q, J = 6.6 Hz, 16H, H^j), 1.69 – 1.57 (m, 16H, H^k), 1.47 – 1.38 (m, 32H, H^{l+m}), 0.96 (t, J = 6.8 Hz, 24H, H^n),

^{13}C NMR (75 MHz, $CDCl_3$) δ (ppm) = 159.5, 152.0, 150.4, 139.3, 136.7, 135.4, 132.5, 132.1, 131.9, 129.5, 129.3, 126.4, 120.3, 111.3, 69.5, 31.7, 29.7, 29.4, 25.9, 22.7, 14.1.

HRMS (MALDI, DCTB + **PPGNa 2000 + PPGNa 2700):** Mass (*m/z*) calculated for $C_{168}H_{168}N_{16}O_8$ Zn_2 $[M]^+$: 2665.1800; found: 2665.1809.

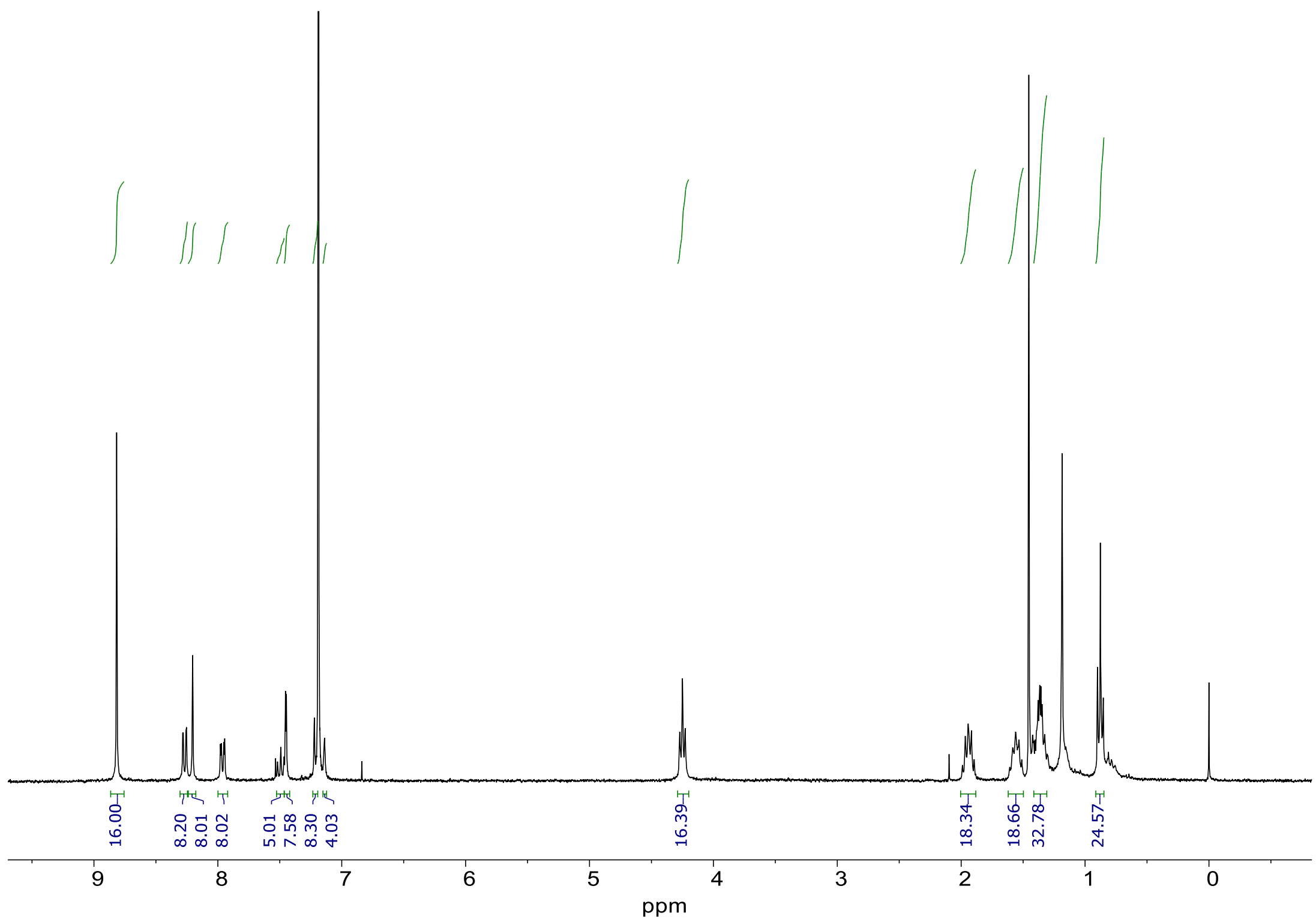

Figure S22. ^{1}H NMR spectrum of cage $\mathbf{1'_{Zn}}$ in $CDCl_3$ at 298 K.

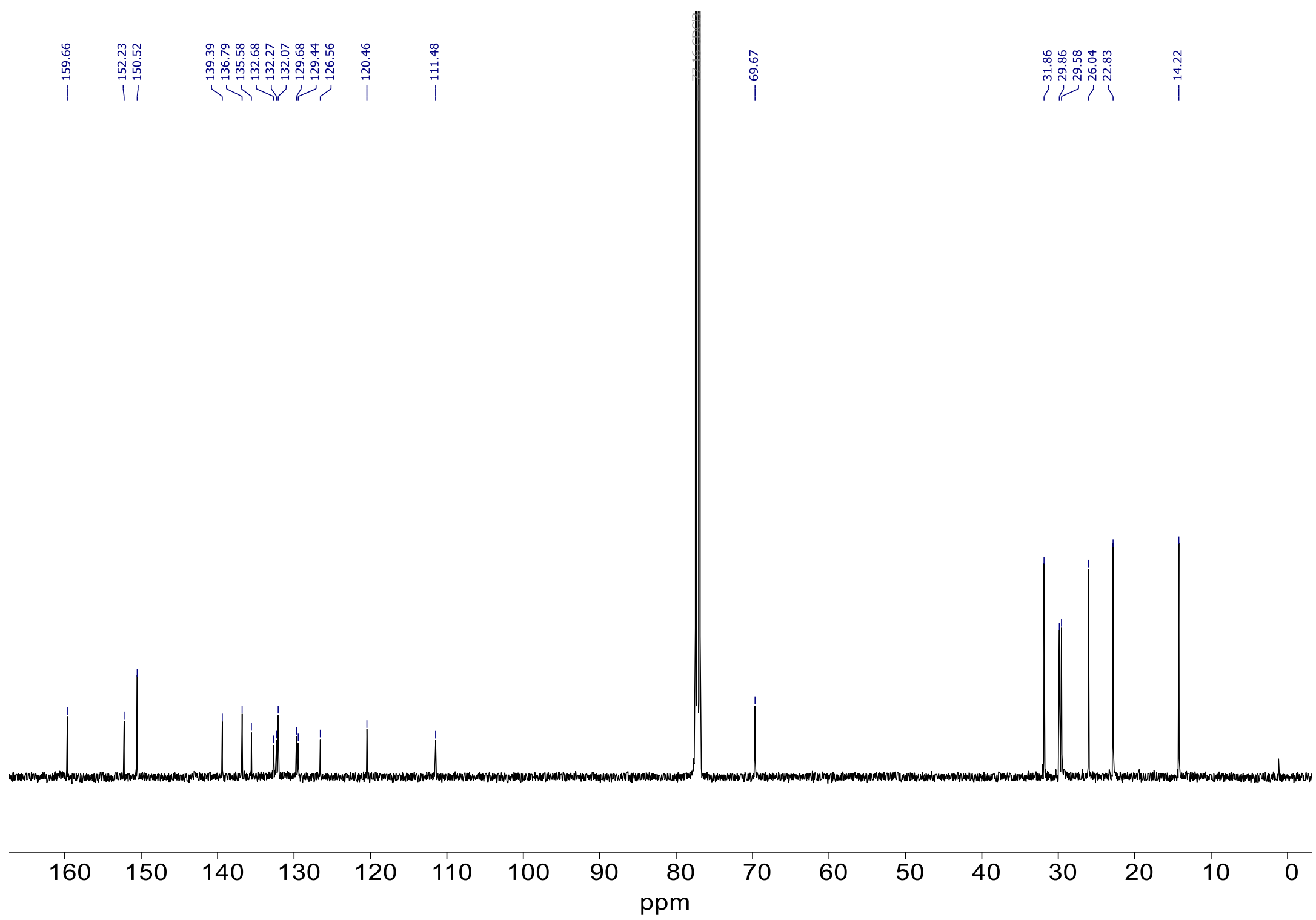

Figure S23. ^{13}C NMR spectrum of cage $\mathbf{1'_{Zn}}$ in $CDCl_3$ at 298 K.

S1. Encapsulation of C_{60} fullerene into 1_{Zn} as a function of time at different temperatures.

Fullerene encapsulation was preliminarily monitored as a function of time by ^{1}H NMR. In a typical experiment, an equimolecular mixture of *jail* **1**$_{Zn}$ and **C**$_{60}$ fullerene (5.0 × 10^{-4} M) in toluene-d$_8$ was placed in a sealed NMR tube, recording the conversion of the encapsulation process at the corresponding temperature by ^{1}H NMR.

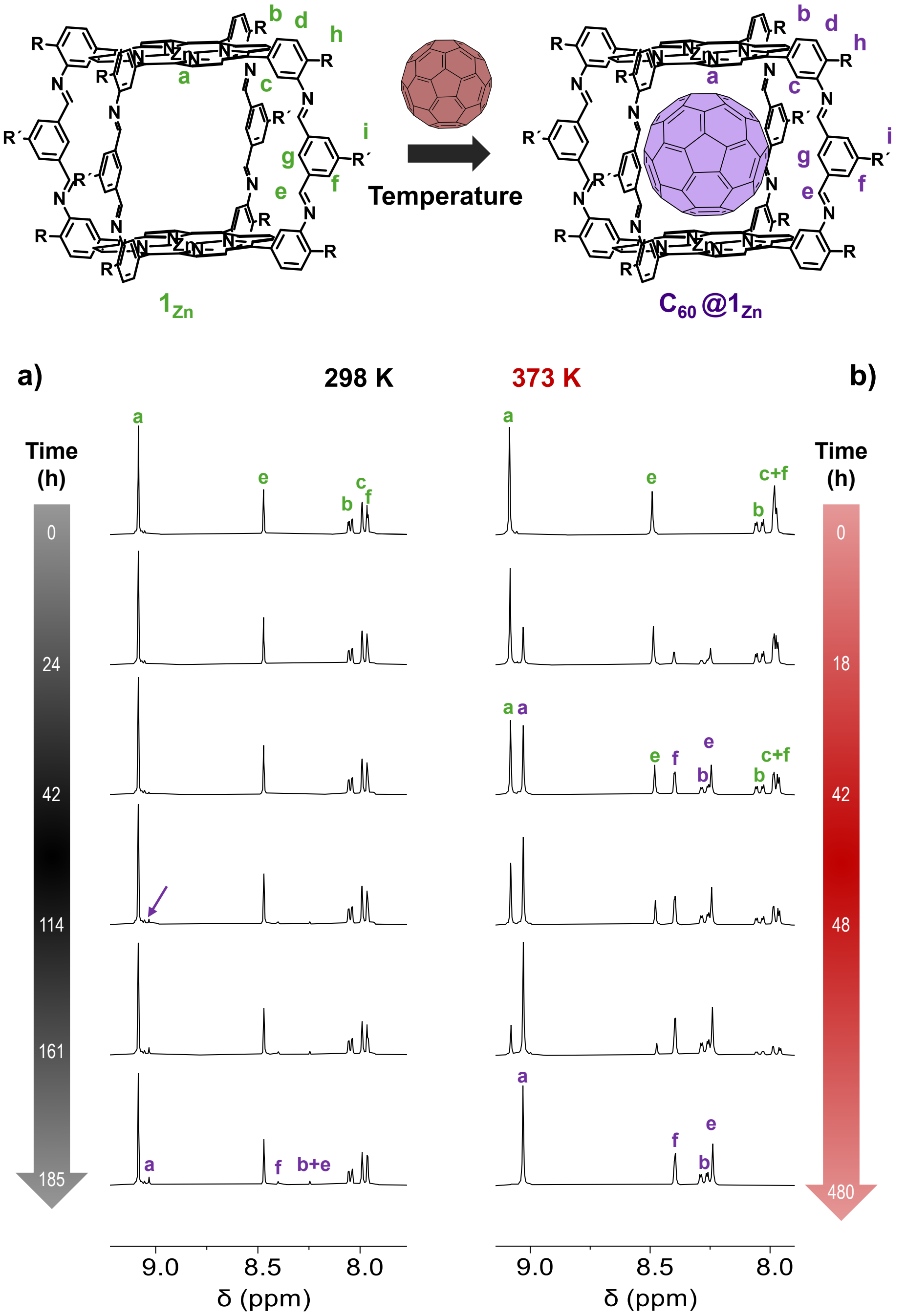

Figure S24. ^{1}H NMR spectra region showing the progressive encapsulation of **C**$_{60}$ by the **1**$_{Zn}$ *jail* (C (**C**$_{60}$) = 5.0 × 10^{-4} M; C (**1**$_{Zn}$) = 5.0 × 10^{-4} M) in toluene-d$_8$ after heating at 298 K (a) and 373 K (b), for the indicated period of time. The most representative proton signals are labelled in green (for the empty *jail*) and purple (for the filled *jail*).

S2. Encapsulation of C_{60} fullerene into 1_{Zn}. Characterization of the $C_{60}@1_{Zn}$ complex.

Fullerene encapsulation was next monitored by ^{1}H, and ^{13}C NMR, MALDI-TOF MS, UV-vis and fluorescence spectroscopies. The NMR experiments were performed in a sealed NMR tube charged with equimolar amounts of *jail* **1**$_{Zn}$ and fullerene **C**$_{60}$ (1.0×10^{-3} M) in toluene-d$_8$. The NMR tube was heated at 373 K in an oil bath. The encapsulation evolution was checked then at different times by ^{1}H NMR measured at 298 K. At conversions of 0, 32, 62, and 100%, ^{13}C NMR spectra were also recorded. Additionally, aliquots were taken from the NMR tube for their analysis by MS and UV-vis and fluorescence spectroscopies upon dilution of the samples.

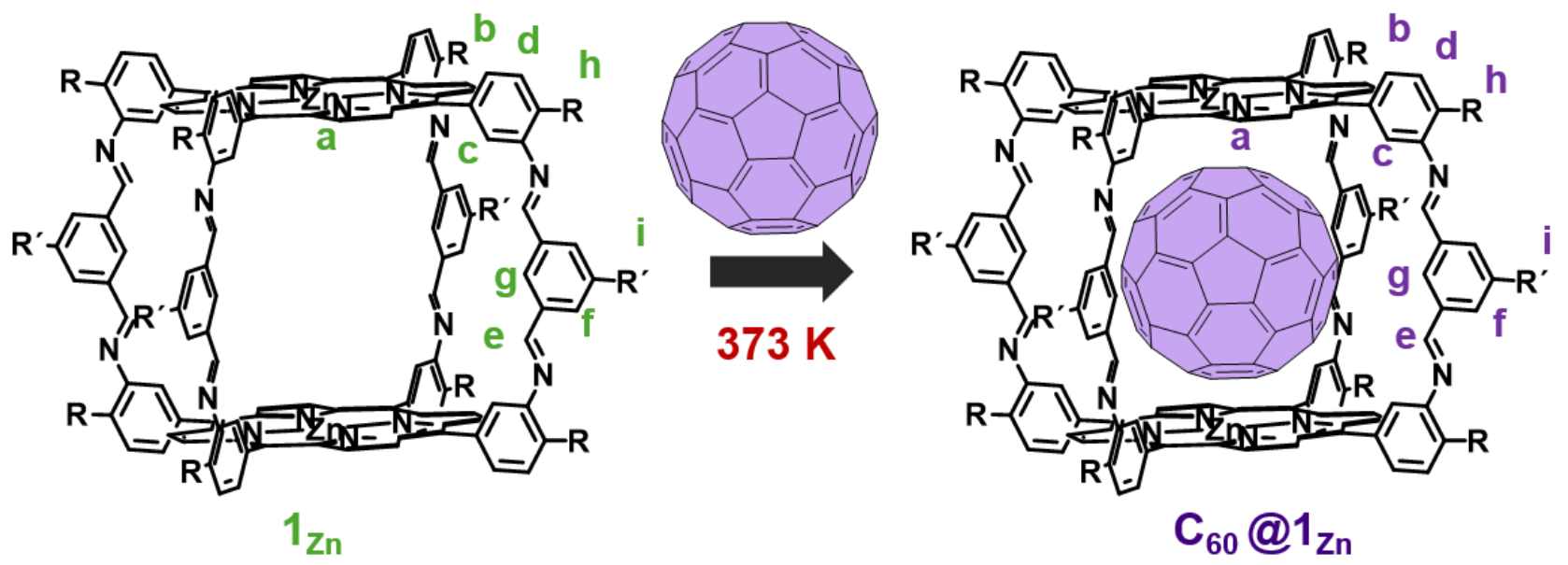

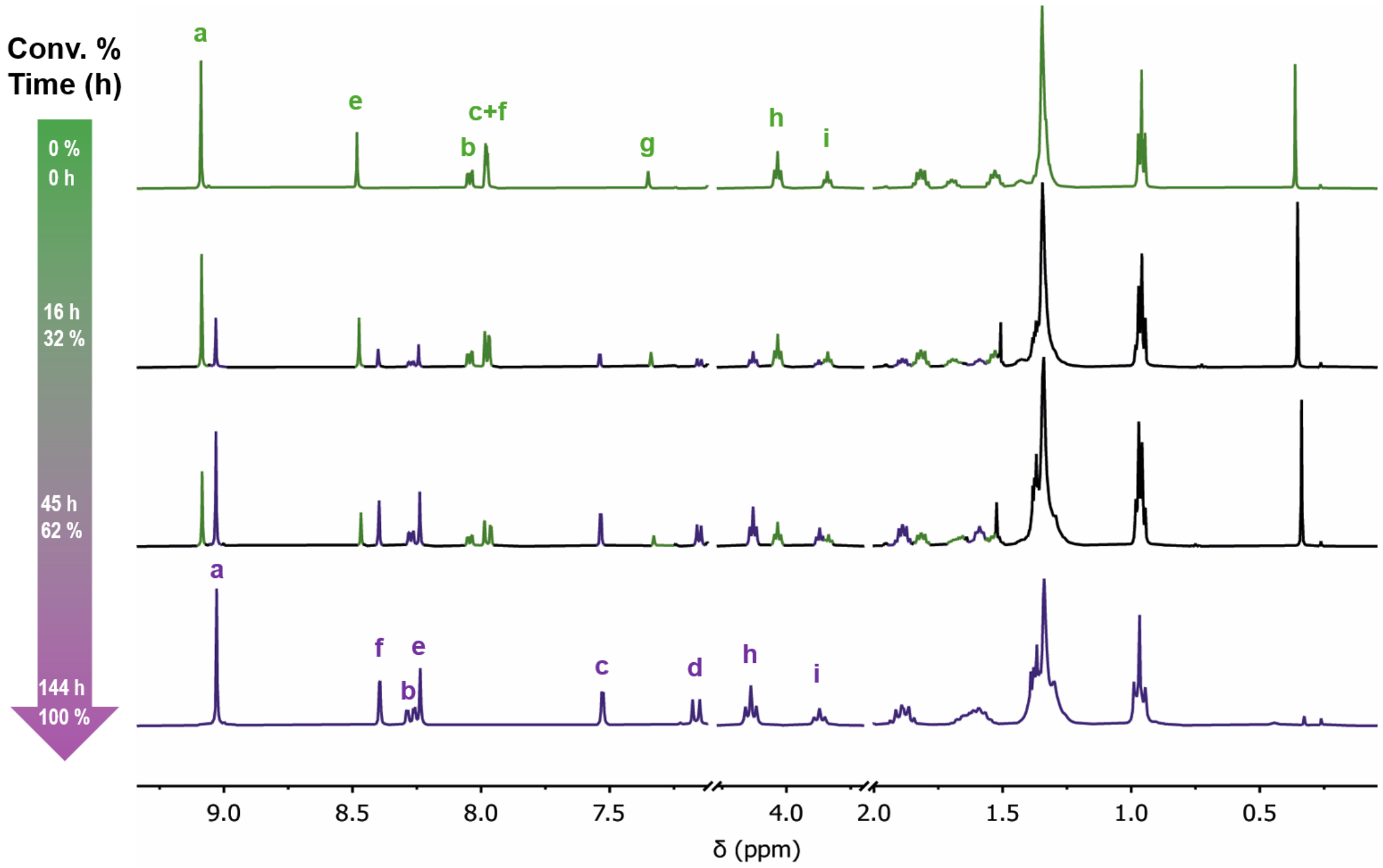

Figure S25. ^{1}H NMR spectra showing the progressive encapsulation of **C**$_{60}$ by the **1**$_{Zn}$ *jail* (C (**C**$_{60}$) = 1.0×10^{-3} M; C (**1**$_{Zn}$) = 1.0×10^{3} M) in toluene-d$_8$ after heating at 373K for the indicated period of time. The most representative proton signals are labelled in green (for the empty *jail*) and purple (for the filled *jail*).

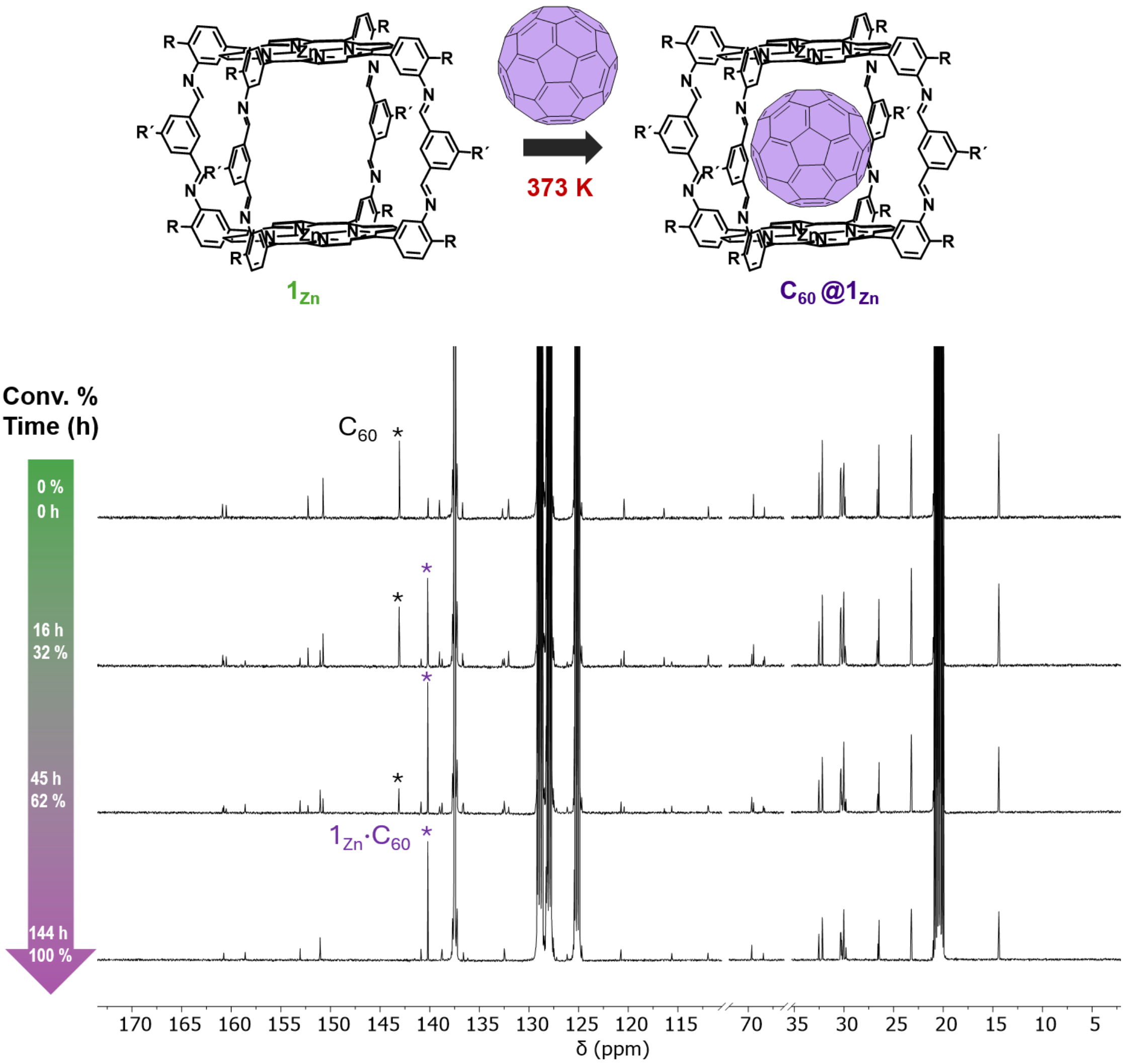

Figure S26. ^{13}C NMR spectra showing the progressive encapsulation of $\mathbf{C_{60}}$ by the $\mathbf{1_{Zn}}$ *jail* (C ($\mathbf{C_{60}}$) = 1.0 × 10^{-3} M; C ($\mathbf{1_{Zn}}$) = 1.0 × 10^{-3} M) in toluene-d_8 after heating at 373K for the indicated period of time. The $\mathbf{C_{60}}$ ^{13}C NMR peak is marked at each spectrum, indicating the fullerene encapsulation.

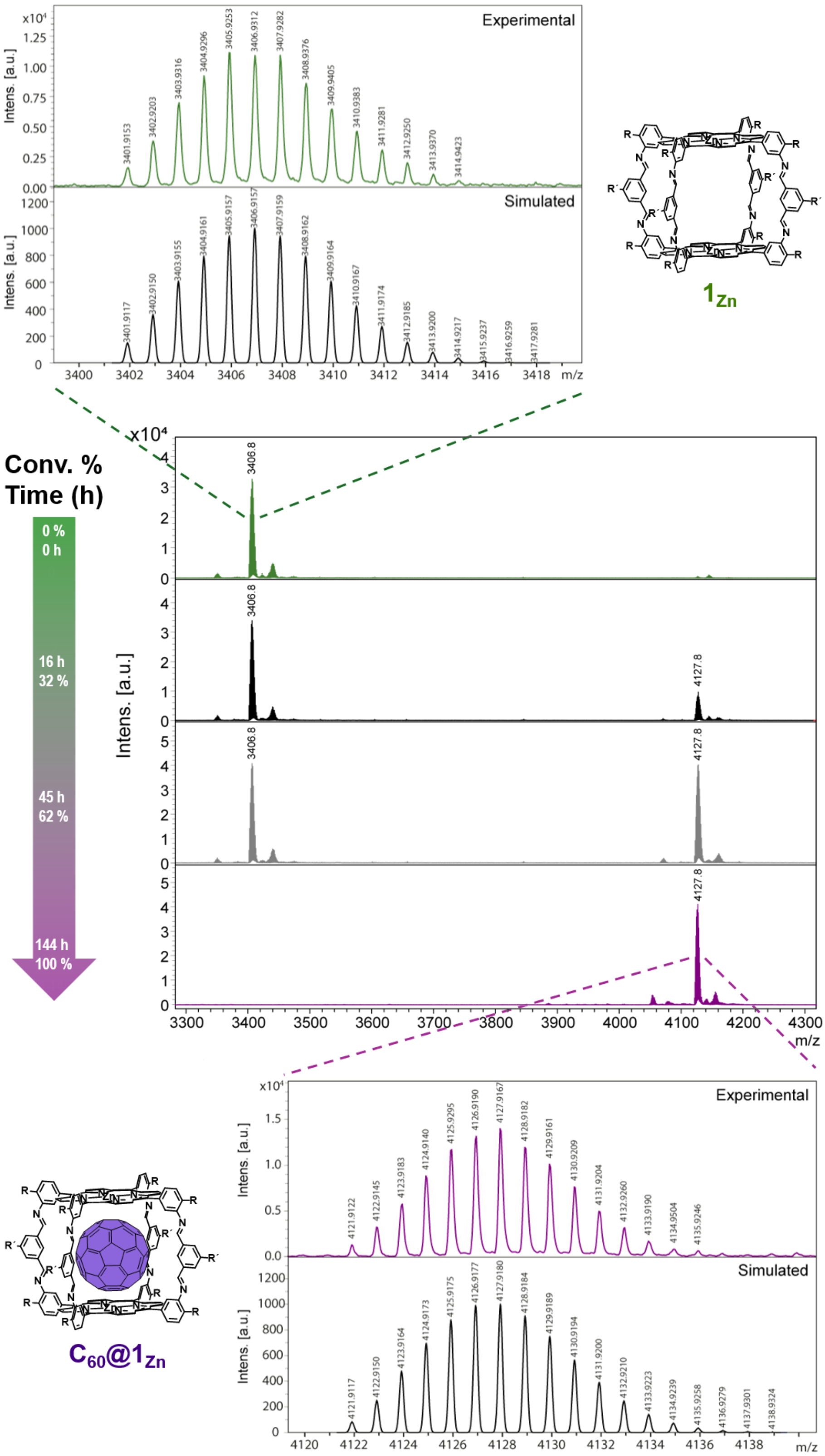

Figure S27. MALDI-TOF mass spectra of different aliquots showing the progressive encapsulation of $\mathbf{C_{60}}$ within the $\mathbf{1_{Zn}}$ *jail* (C ($\mathbf{C_{60}}$) = 1.0 × 10^{-3} M; C ($\mathbf{1_{Zn}}$) = 1.0 × 10^{-3} M) in toluene after heating at 373 K. Peaks at 3406.8 and 4127.8 are the most abundant isotopic peaks and correspond to the molecular formula $C_{216}H_{264}N_{16}O_{12}Zn_2$ $[M]^+$ ($\mathbf{1_{Zn}}$) and $C_{276}H_{264}N_{16}O_{12}Zn_2$ $[M]^+$ ($\mathbf{C_{60}@1_{Zn}}\cdot$), respectively.

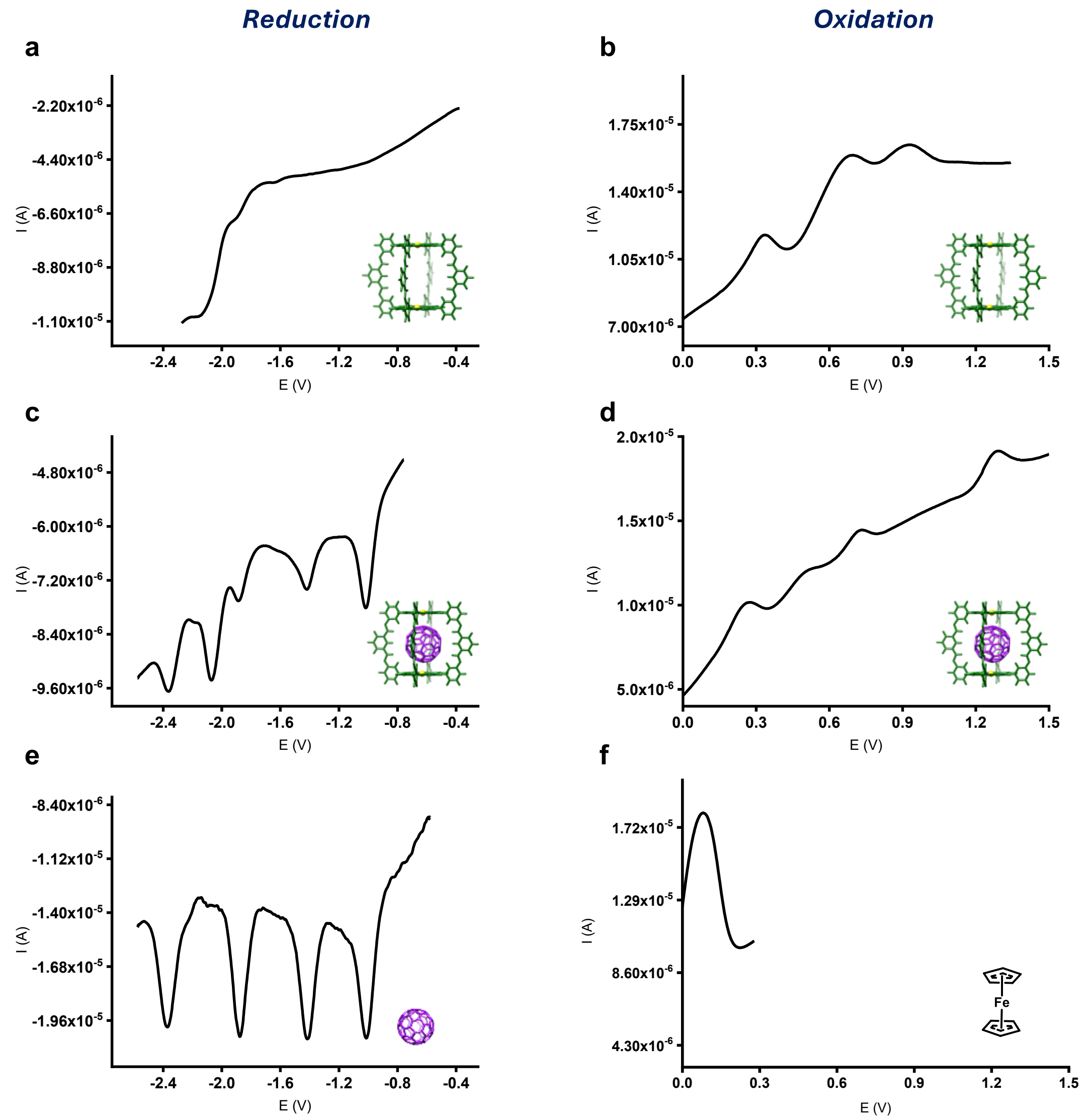

Figure S28. Reduction and oxidation square wave (SW) voltammograms of (a,b) *jail* $\mathbf{1_{Zn}}$, (c,d) the $\mathbf{C_{60}@1_{Zn}}$ complex, as well as (e) $\mathbf{C_{60}}$, and (f) ferrocene references. The experiments were performed in degassed *o*-dichlorobenzene under a nitrogen flow. All the voltammograms were obtained in the presence of the ferrocene reference.

Table S1. Oxidation and reduction potentials obtained from SW voltammetry for compound $\mathbf{1_{Zn}}$, the $\mathbf{C_{60}@1_{Zn}}$ complex, $\mathbf{C_{60}}$, and ferrocene.

	E_4^{red}	E_3^{red}	E_2^{red}	E_1^{red}	E_2^{red}	E_1^{red}	E_1^{ox}	E_1^{ox}	E_2^{ox}	E_3^{ox}
$\mathbf{1_{Zn}}$			-2.17	-1.91			0.28	0.69	0.93	
$\mathbf{C_{60}@1_{Zn}}$	-2.36	-1.88	-2.06		-1.42	-1.02	0.29	0.50	0.74	1.29
$\mathbf{C_{60}}$	-2.38	-1.87			-1.41	-1.01				
**ferrocene**							0.08			

S3. Density functional theory calculations on C_{60}@1_{Zn}, 2_{Zn}·C_{60} and 3_{Zn}·C_{60}

Electronic structure calculations, ground state optimizations, and single points calculations were performed using density functional theory (DFT) employing the hybrid B3LYP density functional,[3] the Grimme's empirical dispersion D3 version together with the Becke-Johnson damping function (GD3BJ),[4,5] and the cc-pVDZ basis set[6] as implemented in the A.03 revision of the Gaussian 16 software package.[7] The inner cavity volumes of the **1_{Zn}**, **2_{Zn}**, and **3_{Zn}** hosts were calculated from the optimized structures of the **C_{60}@1_{Zn}**, **2_{Zn}·C_{60}**, and **3_{Zn}·C_{60}** complexes by removing **C_{60}** and using the *CageCavityCalc* plugin for Python.[8]

Figure S29 shows the frontier molecular orbitals calculated for the **C_{60}@1_{Zn}** complex, the isolated **1_{Zn}** *jail*, and the **C_{60}** guest. Minor perturbations are predicted for the HOMO and LUMO energies upon complex formation $(\Delta E^{HOMO}_{C_{60}@1_{Zn}\text{-}1_{Zn}} = 0.04\ \text{eV}$ and $\Delta E^{LUMO}_{C_{60}@1_{Zn}\text{-}C_{60}} = 0.20\ \text{eV})$, supporting the weak electronic interaction between the host and the guest in the ground electronic state. The topology of the frontier molecular orbitals, fully localized on the **1_{Zn}** *jail* (HOMO) and **C_{60}** (LUMO), clearly indicates that the HOMO→LUMO excitation would imply a significant charge transfer from the *jail* to **C_{60}**.

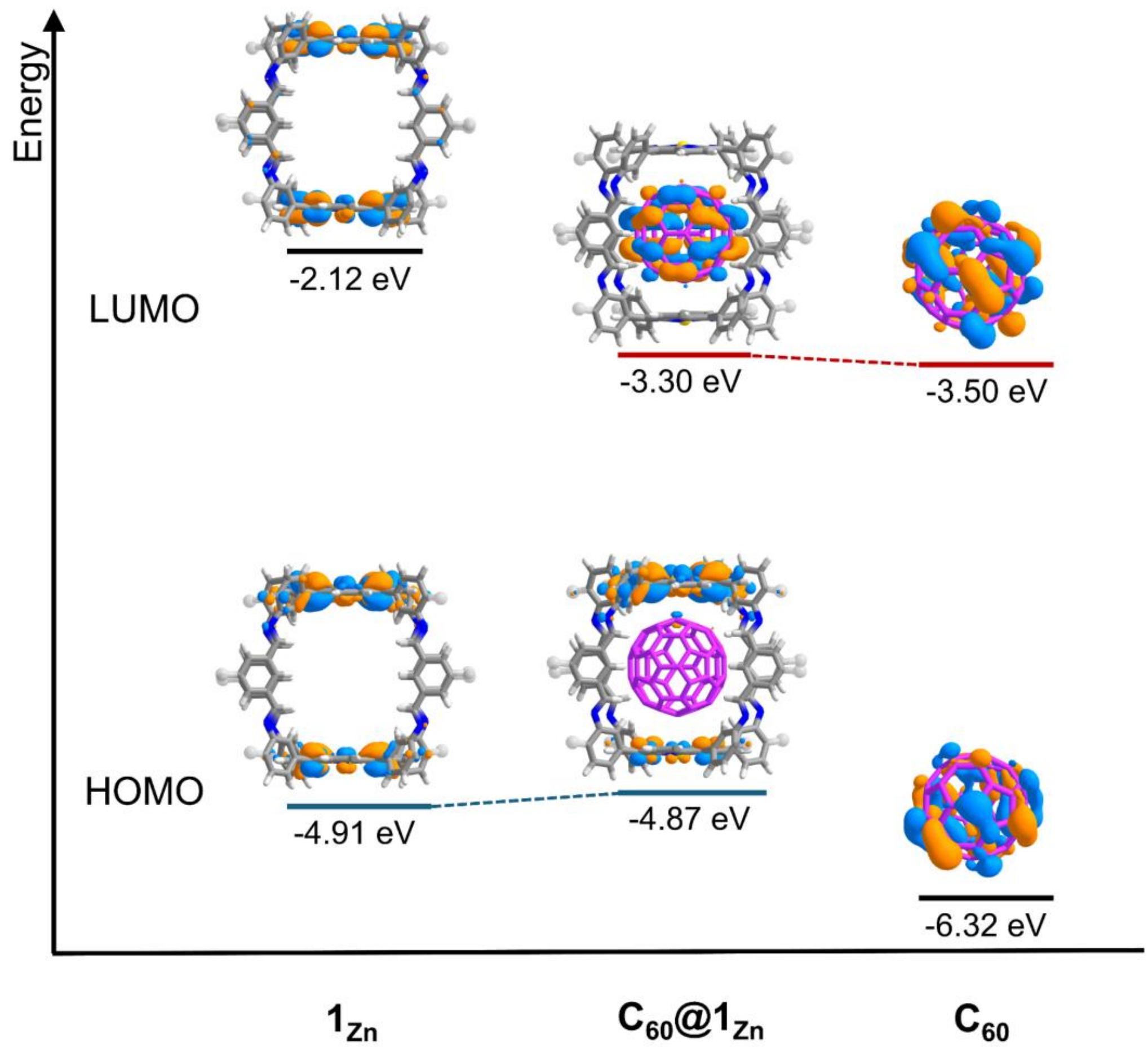

Figure S29. B3LYP-D3/cc-pVDZ energy diagram showing the orbital isosurfaces (contour value = 0.02 bohr^{-3}) calculated for the HOMO/LUMO of **C_{60}@1_{Zn}**, the isolated **1_{Zn}** host, and the **C_{60}** guest.

Figures S30-32 show the DFT-optimized structures of the three host:guest complexes from different viewpoints (front, side, and top), providing a clearer and more comprehensive visualization of its three-dimensional spatial arrangement.

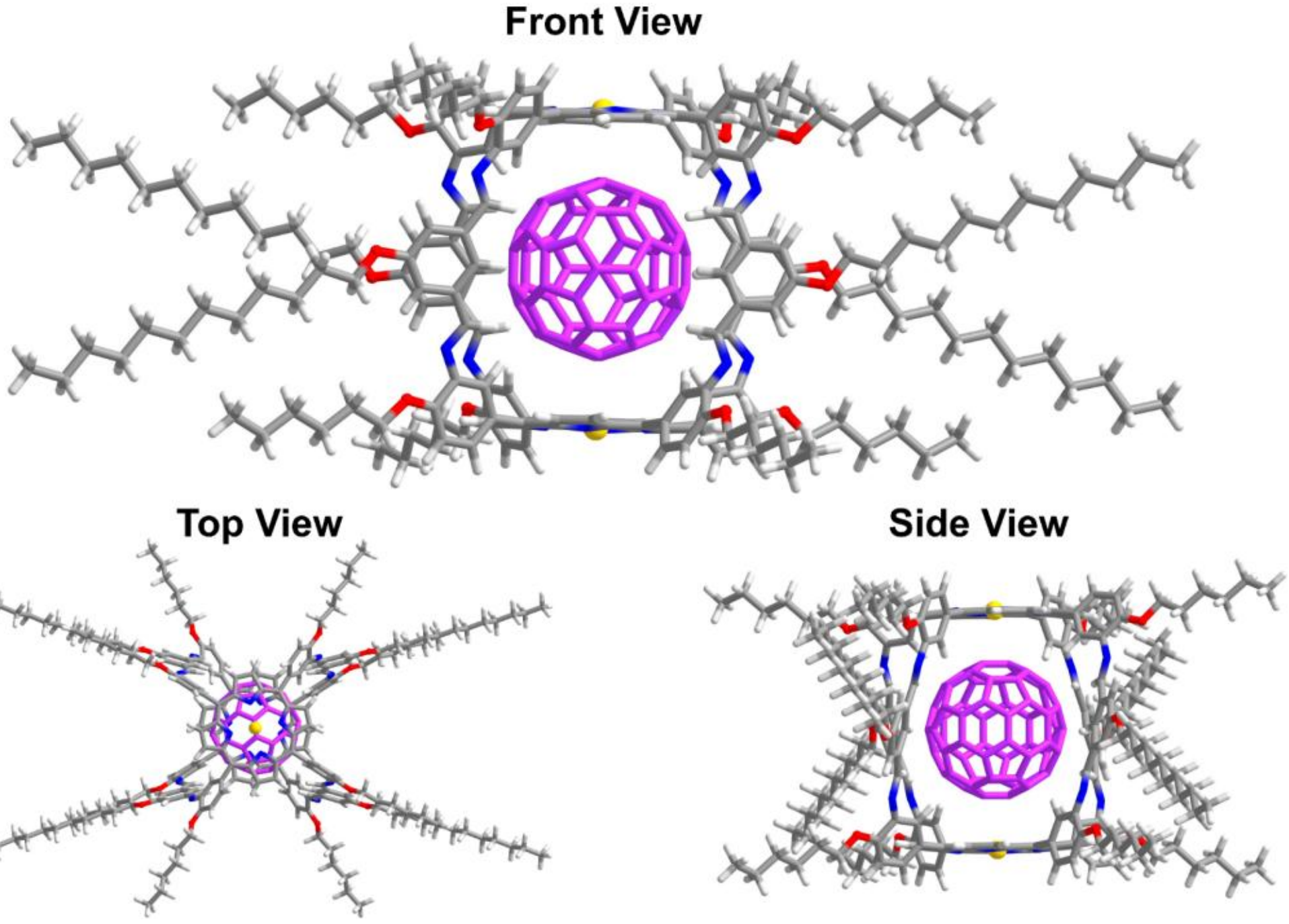

Figure S30. Front, top, and side views of the B3LYP-D3/cc-pVDZ-optimized structure calculated for the **C_{60}@1_{Zn}** complex.

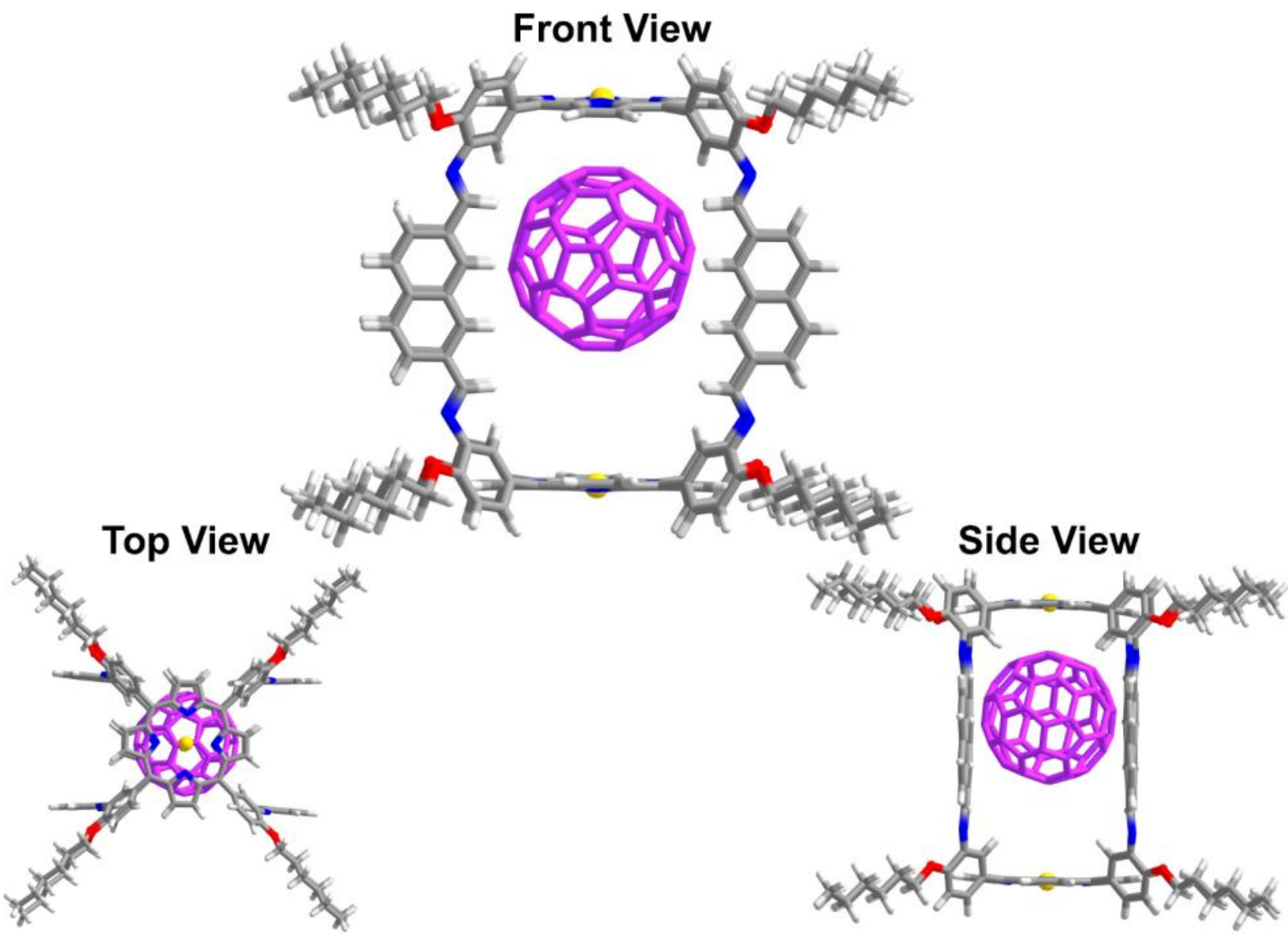

Figure S31. Front, top, and side views of the B3LYP-D3/cc-pVDZ-optimized structure calculated for the **2_{Zn}·C_{60}** complex.

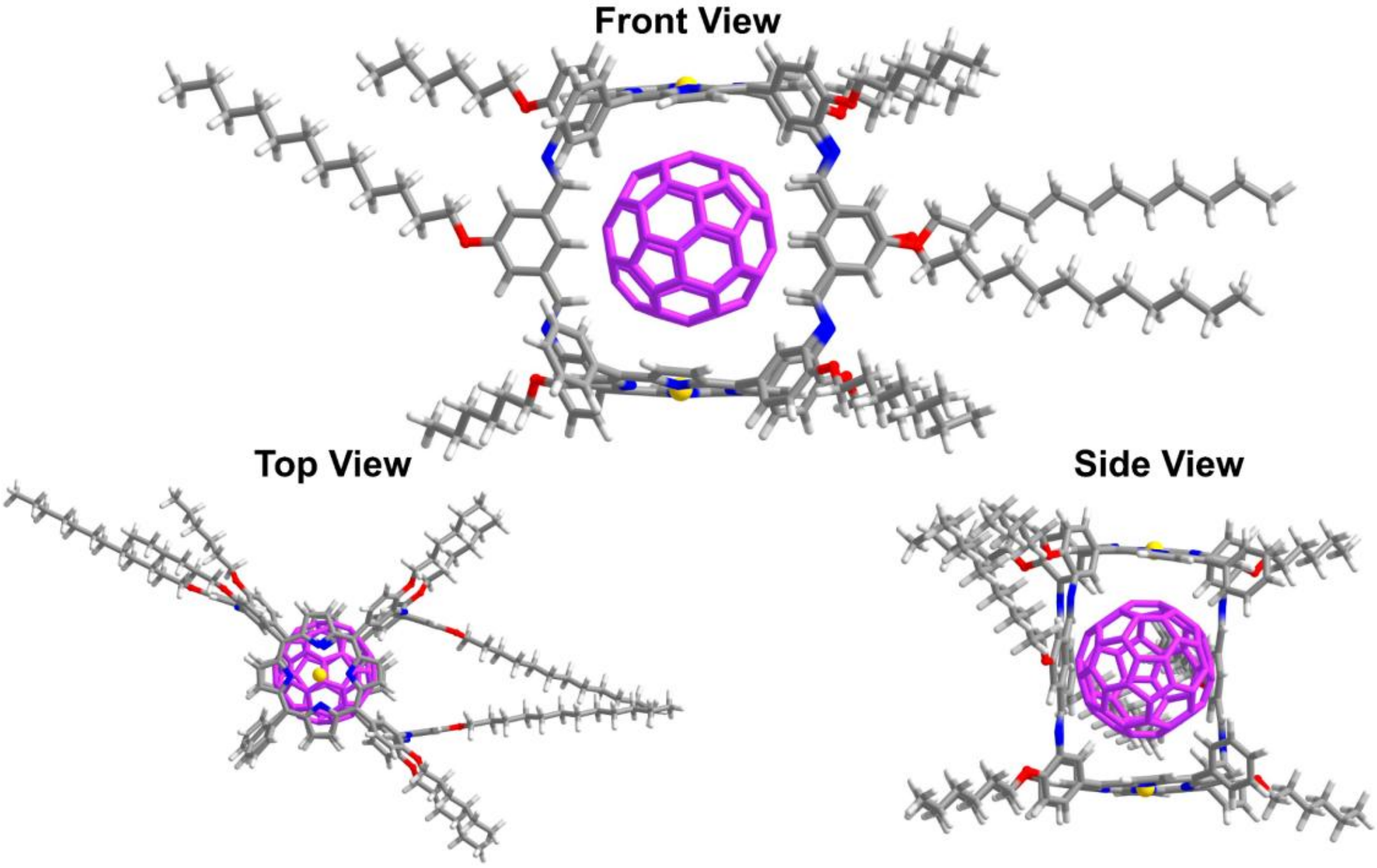

Figure S32. Front, top, and side views of the B3LYP-D3/cc-pVDZ-optimized structure calculated for the $\mathbf{3_{Zn}{\cdot}C_{60}}$ complex.

Interaction energies (ΔE_{int}) between the host and the guest were calculated for the three complexes as the energy difference between the total energy of the host:guest complex AB and the energies of its two components at the optimized geometry of the complex using the expression $\Delta E_{\mathrm{int}}(AB) = E(AB) - E(A^{AB}) - E(B^{AB})$. The ΔE_{int} values were analyzed using a fragment-based approach to perform an energy dissection in terms of interactions between specific molecular fragments (see Figure S33). Single-point energy calculations were performed on the interacting fragments at the same level of theory as the geometry optimizations. Due to the introduction of hydrogen atoms to cap the fragments, the sum of the interaction energies between individual fragments slightly overestimates the interaction energy calculated for the host:guest complex. Accordingly, relative contributions in the main text are reported as percentages referenced to the sum of the fragments interaction energies.

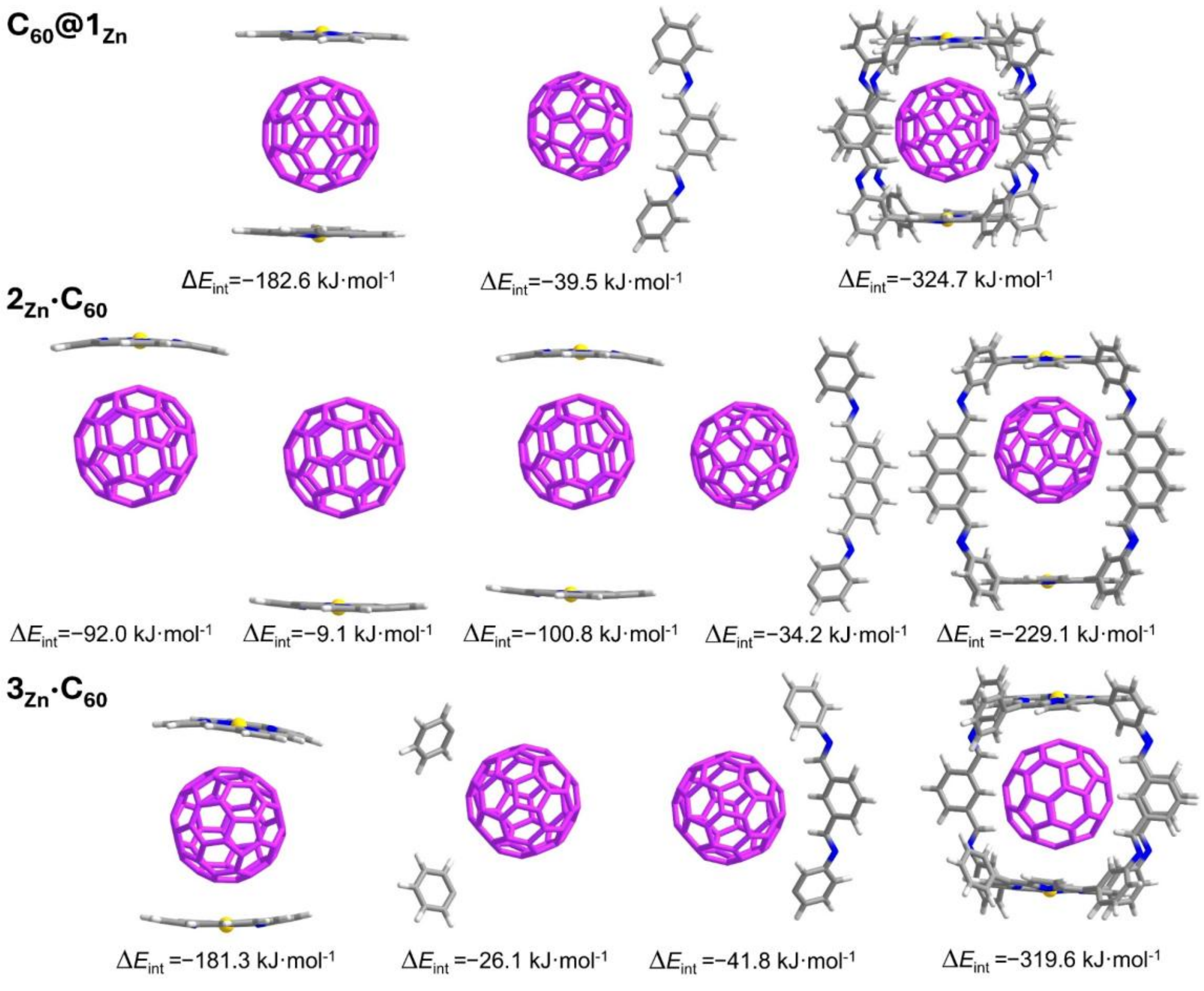

Figure S33. Representation of the molecular structures, extracted from the B3LYP-D3/cc-pVDZ-optimized geometries of the host:guest complexes, used for single-point calculations of the interaction energies (ΔE_{int}).

The key distances defining the structure and the most relevant intermolecular interactions taking place for each optimized supramolecular host:guest assembly are listed in Tables S2-4. For **C_{60}@1_{Zn}** and **3_{Zn}·C_{60}**, the porphyrin units remain at similar distances, with comparable Zn···Zn distances of 12.63 Å for **C_{60}@1_{Zn}** and 12.60 Å for **3_{Zn}·C_{60}**, and short Zn···C_{60} contacts in the 2.85–2.95 Å range. In both cases, the porphyrin moieties adopt a concave shape with the Zn atoms protruding out the cavity to better wrap **C_{60}**. To maximize the interaction with the guest, the porphyrin planes are significantly rotated in **C_{60}@1_{Zn}** (24.8°) but remain nearly eclipsed in **3_{Zn}·C_{60}** (4.9°). The absence of one linker in **3_{Zn}·C_{60}** allows for a more pronounced and asymmetric bending of the porphyrin planes, which is reflected in a slight reduction of the porphyrin centroid-centroid distance from 12.34 Å in **C_{60}@1_{Zn}** to 12.24 Å in **3_{Zn}·C_{60}**.

Differences between both the *jail* **C_{60}@1_{Zn}** and the *basket* **3_{Zn}·C_{60}** become slightly more pronounced when looking at the linkers connecting the two porphyrins (see Tables S2 and S4). For **C_{60}@1_{Zn}**, the averaged linker length is 12.09 Å, and a similar value is found for the linkers of **3_{Zn}·C_{60}** (11.95 Å). In contrast, the phenyl rings in *meso* positions, that are not covalently connected, are placed at a much shorter distance of 10.23 Å, giving rise to a more flexible and folded cavity. This structural feature is reflected in the gate dimensions of the hosts, with **C_{60}@1_{Zn}** presenting a smaller gate of 10.78 Å and a larger one of 12.71 Å, whereas **3_{Zn}·C_{60}** exhibiting a significantly enlarged gate of 16.48 Å corresponding to the position lacking a linker. Despite this flexibility, the resulting CH···C_{60} contacts show similar distances. For instance, the CH···C_{60} contact involving the phenyl ring of the linkers (CH_1···C_{60}) is set at 2.92 and 2.85 Å for **C_{60}@1_{Zn}** and **3_{Zn}·C_{60}**, respectively, whereas the corresponding CH···C_{60} contacts involving the imine groups (CH_2···C_{60}) are set at 3.14 and 3.04 Å. Similar CH···C_{60} contacts are computed for the benzene units directly attached to the porphyrins, with distances of 3.11 Å and 2.99 Å for *jail* and *basket*, respectively.

These subtle geometric differences result in slightly distinct cavity sizes for the two complexes. The more rigid architecture of the *jail* defines a compact cavity with a volume of 764 $Å^3$, whereas the increased adaptability of *basket* leads to a slightly larger cavity of 773 $Å^3$, making them suitable hosts for **C_{60}** (see Figure S34).

The replacement of benzene units by naphthalene moieties in the **2_{Zn}** *cage* leads to significantly longer linkers and to an expanded structure with a Zn···Zn separation of 15.34 Å where the two porphyrin units are nearly eclipsed, with a small rotational offset of 1.9°. Therefore, **C_{60}** interacts preferentially with one of the two porphyrins (see Table S3), the Zn···C_{60} distances being of 2.93 and 5.59 Å, respectively. The naphthalene linkers have an average length of 14.47 Å and partially interact with **C_{60}**, establishing CH···C_{60} contacts from 2.86 to 3.25 Å with naphthalene and from 3.05 up to 4.58 Å with the imine groups. Similarly, CH···C_{60} contacts involving the benzene units attached to the porphyrins vary from 2.92 to 4.97 Å. The longer linkers result in two gates, a smaller of 9.10 Å, due to the nearly eclipsed naphthalene in *meso*, and a larger one of 13.64 Å. The expanded structure of the *cage* provides a substantially larger cavity volume of 1117 $Å^3$ (see Figure S34), allowing increased mobility of **C_{60}**.

Table S2. Relevant average distances for the B3LYP-D3/cc-pVDZ-optimized **C_{60}@1_{Zn}** supramolecular complex.

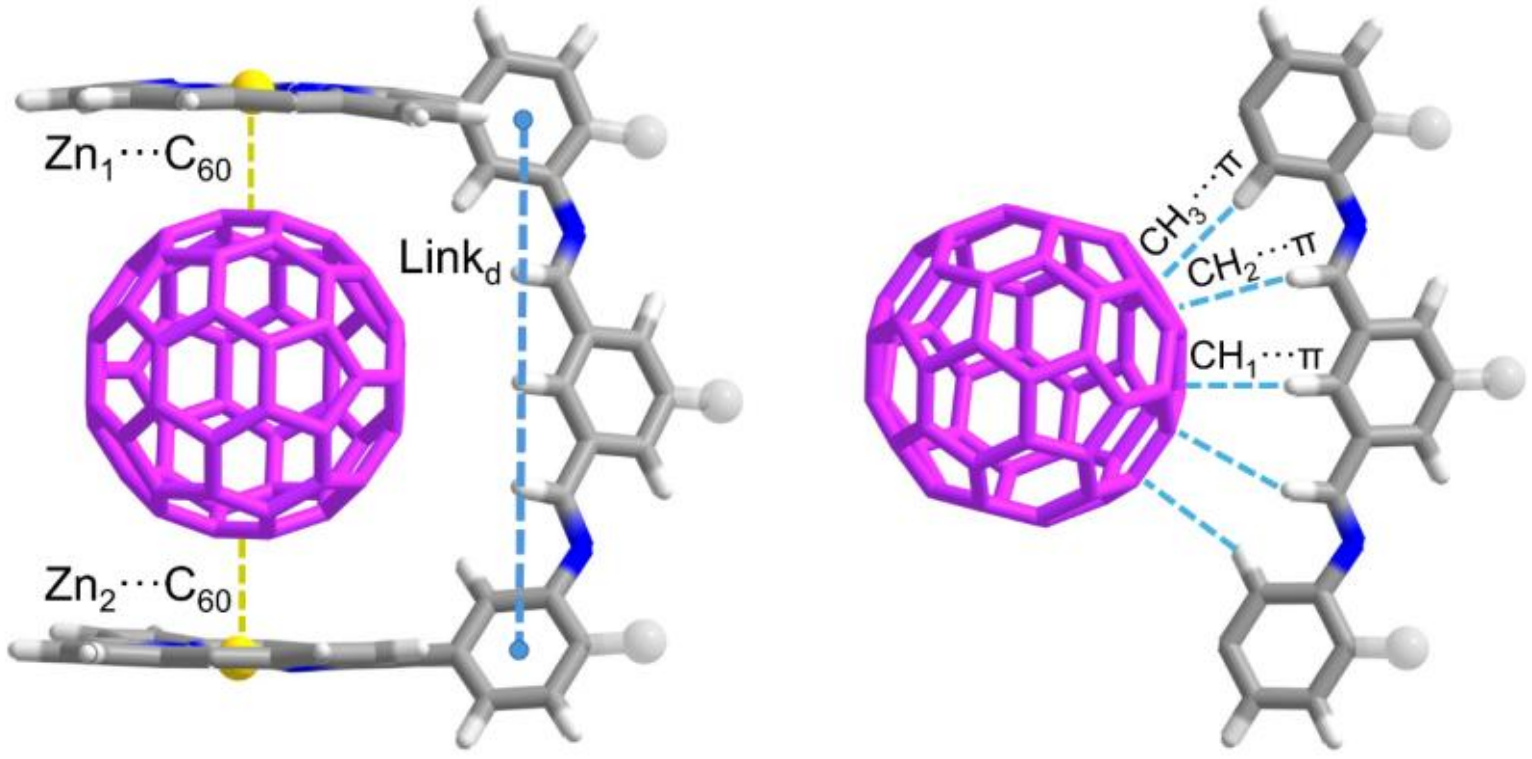

Parameter	Distance (Å)
$Zn_1\cdots Zn_2$	12.63
$Porphyrin_{centroid}\cdots Porphyrin_{centroid}$	12.34
$Zn_1\cdots C_{60}$	2.95
$Zn_2\cdots C_{60}$	2.90
$Link_d$	12.09
$CH_1\cdots C_{60}$	2.92
$CH_2\cdots C_{60}$	3.14
$CH_3\cdots C_{60}$	3.11

Table S3. Relevant average distances for the B3LYP-D3/cc-pVDZ-optimized **2_{Zn}·C_{60}** supramolecular complex.

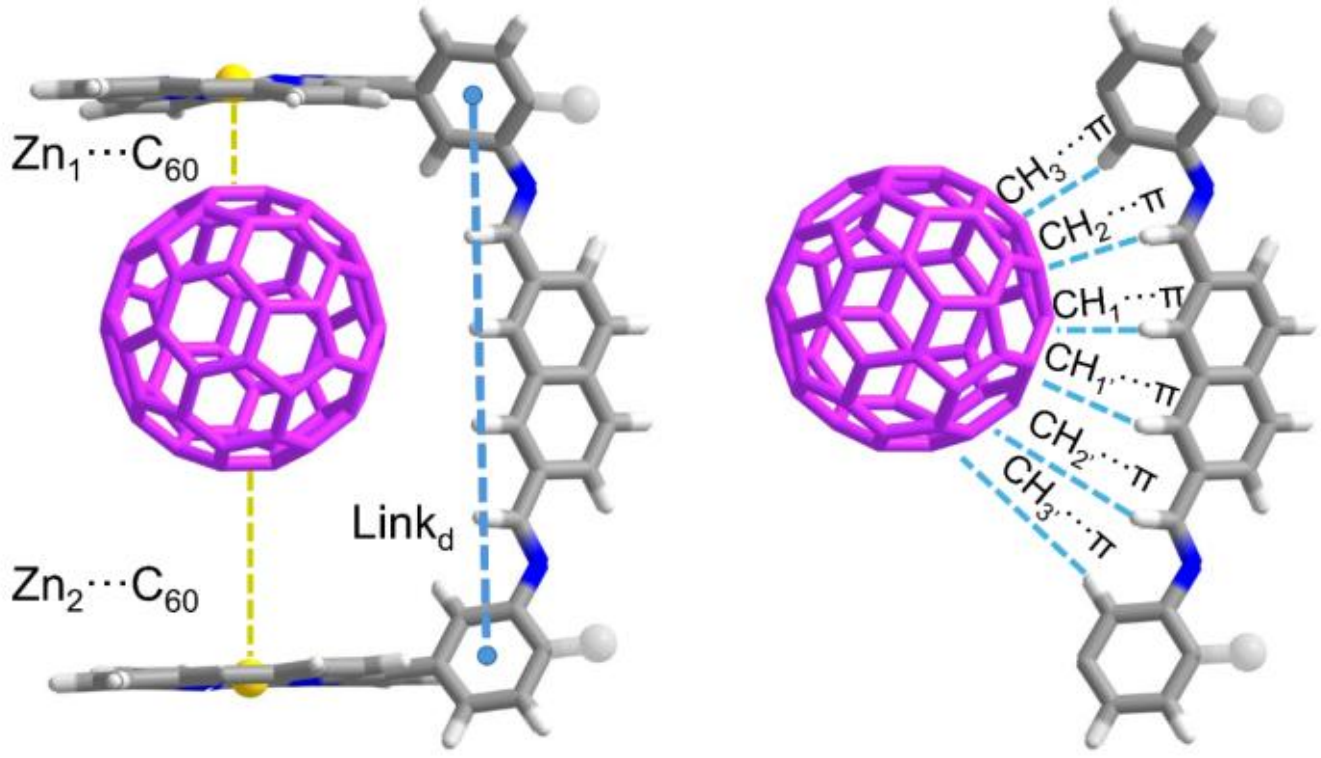

**Parameter**	**Distance (Å)**
$Zn_1 \cdots Zn_2$	15.34
$Porphyrin_{centroid} \cdots Porphyrin_{centroid}$	15.25
$Zn_1 \cdots C_{60}$	2.93
$Zn_2 \cdots C_{60}$	5.59
$Link_d$	14.47
$CH_1 \cdots C_{60}$	2.86
$CH_{1'} \cdots C_{60}$	3.25
$CH_2 \cdots C_{60}$	3.05
$CH_{2'} \cdots C_{60}$	4.58
$CH_3 \cdots C_{60}$	2.92
$CH_{3'} \cdots C_{60}$	4.97

Table S4. Relevant average distances for the B3LYP-D3/cc-pVDZ-optimized $\mathbf{3_{Zn}{\cdot}C_{60}}$ supramolecular complex.

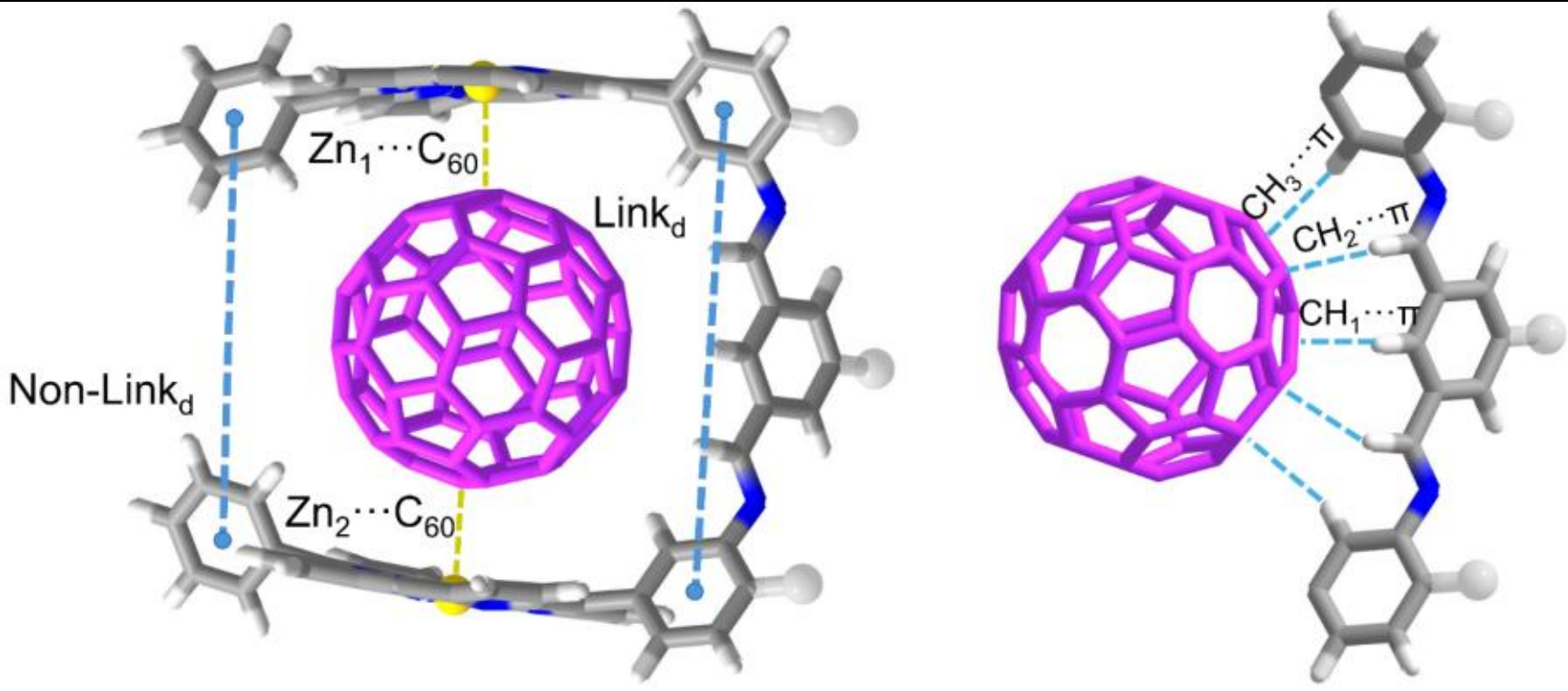

Parameter	Distance (Å)
$Zn_1{\cdots}Zn_2$	12.60
$Porphyrin_{centroid}{\cdots}Porphyrin_{centroid}$	12.24
$Zn_1{\cdots}C_{60}$	2.85
$Zn_2{\cdots}C_{60}$	2.93
$Link_d$	11.95
$No\text{-}Link_d$	10.23
$CH_1{\cdots}C_{60}$	2.85
$CH_2{\cdots}C_{60}$	3.04
$CH_3{\cdots}C_{60}$	2.99

Cavity volumes were computed using the *CageCavityCalc* program with default parameters (grid spacing = 1.0 Å; probe radius = 2.0 Å) on the B3LYP-optimized structures of the $\mathbf{C_{60}@1_{Zn}}$, $\mathbf{2_{Zn}{\cdot}C_{60}}$, and $\mathbf{3_{Zn}{\cdot}C_{60}}$ after removal of $\mathbf{C_{60}}$, yielding values of 764, 773, and 1117 Å^3 for *jail*, *basket*, and *cage*, respectively (see Figure S34).

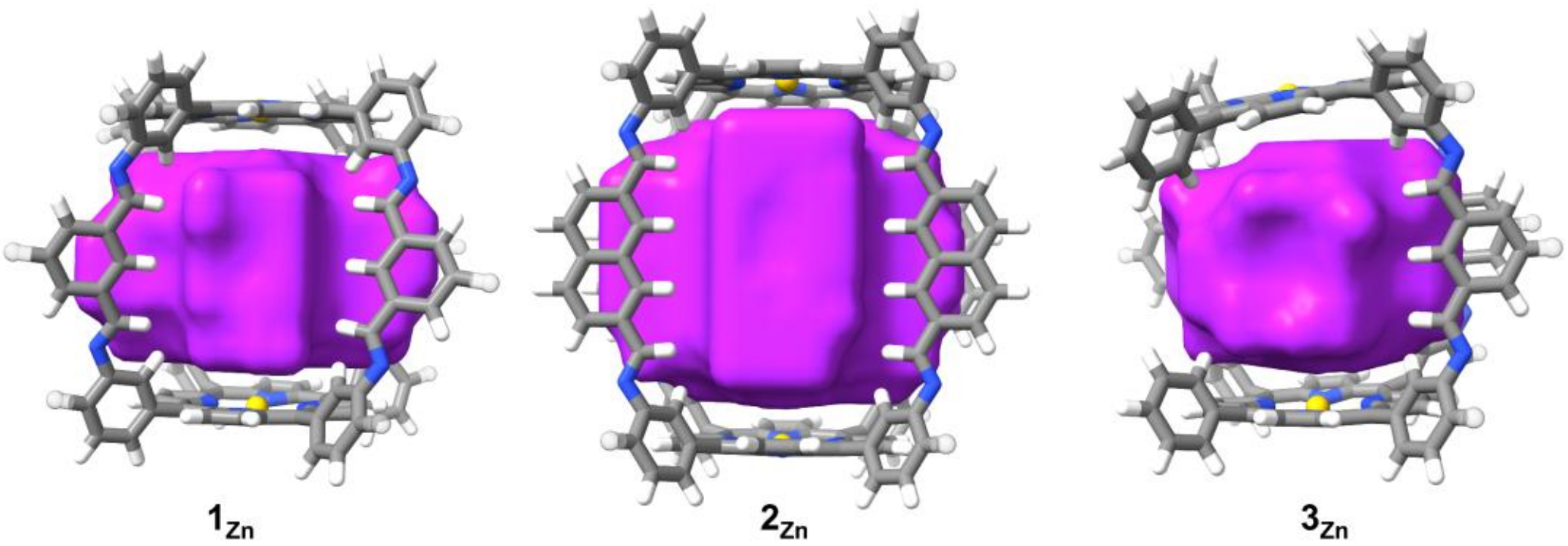

Figure S34. Cavity representations of the optimized $\mathbf{C_{60}@1_{Zn}}$, $\mathbf{2_{Zn}{\cdot}C_{60}}$, and $\mathbf{3_{Zn}{\cdot}C_{60}}$ structures after removal of $\mathbf{C_{60}}$, using the *CageCavityCalc* plugin.

S4. Solid state crystal structure of C_{60}@**1'**$_{Zn}$.

A cage related to **1**$_{Zn}$, but lacking the solubilizing $OC_{12}H_{25}$ alkoxy chains placed at the linker (**1'**$_{Zn}$) was prepared in order to obtain suitable crystals for X-ray diffraction.

The complex **C_{60}@1'**$_{Zn}$ was obtained through the encapsulation of the fullerene **C_{60}**, using the following method: to a solution of cage **1'**$_{Zn}$ (1 eq) and fullerene **C_{60}** (1.5 eq) in toluene, TFA (0.25 eq) was added under inert atmosphere. The mixture was heated at reflux, overnight. Then, TEA (0.5 eq) was added to neutralize the reaction. Later, MeCN was added helping to crash out a purple solid and the suspension was filtered and the solid rinsed with MeCN. Afterwards, the obtained solid was purified by column chromatography using basic aluminium oxide as stationary phase and a toluene:DCM mixture (100:0 to 0:100) as the eluent, obtaining the desired compound as a dark green solid.

The complexation of **C_{60}@1'**$_{Zn}$ was monitored by ^{1}H and ^{13}C NMR (Figure S35). In the left ^{1}H NMR stacked spectra, some signals corresponding to the **1'**$_{Zn}$ cavity are highlighted, showing an abrupt change in the chemical shift due to the presence of **C_{60}**. Furthermore, in the right ^{13}C NMR stacked spectra, an intense signal appears, confirming the incorporation of the fullerene into the cage, leading to the complex **C_{60}@1'**$_{Zn}$.

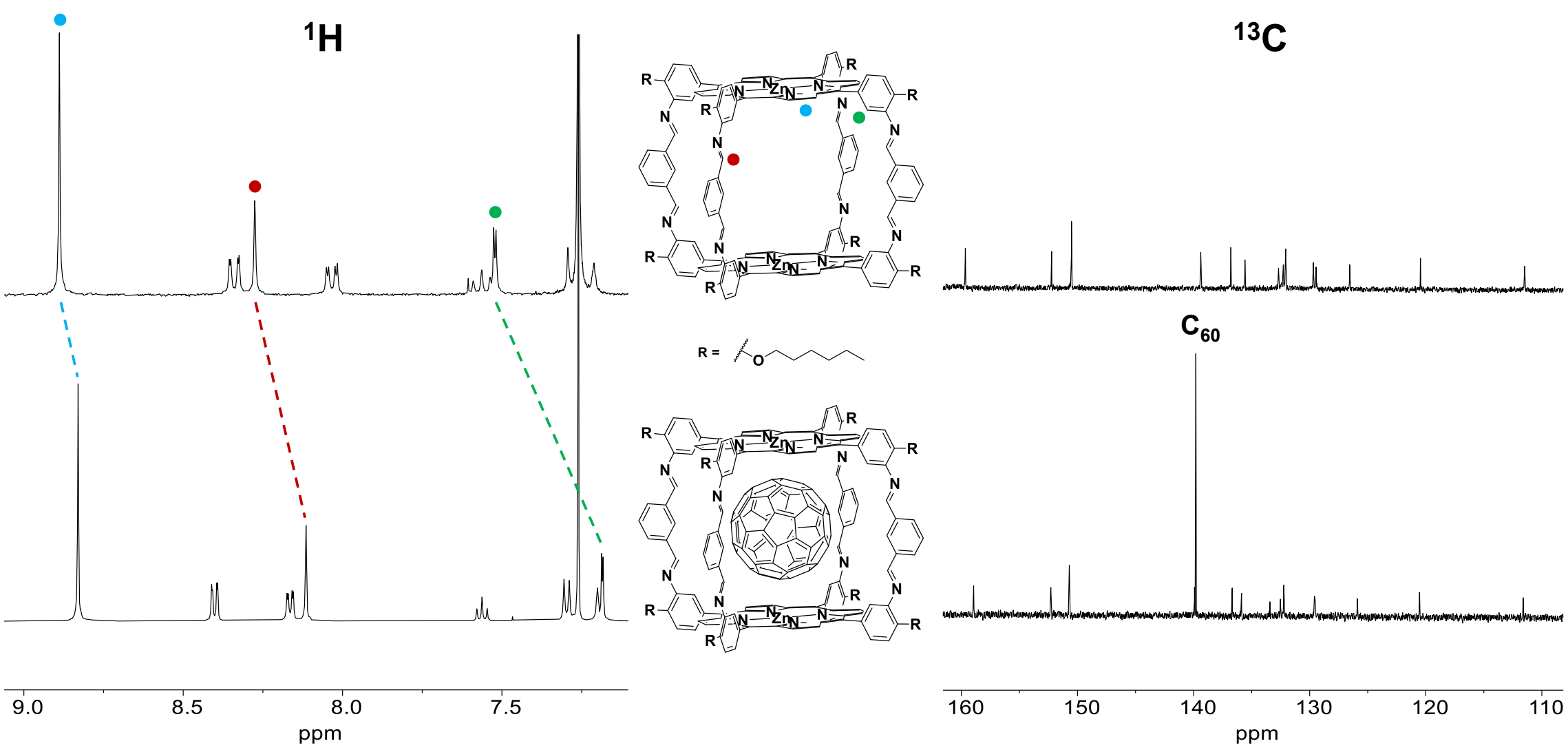

Figure S35. Comparison of ^{1}H and ^{13}C NMR spectra showing the encapsulation of **C_{60}** by cage **1'**$_{Zn}$ in $CDCl_3$ at 298 K.

Single dark purple prism-shaped crystals of **C_{60}@1’$_{Zn}$**were obtained by slow cooling a solution in a toluene/ DMSO solvent mixture. These crystals are highly unstable out of the mother liquor due to the loss of solvent.

A suitable crystal of dimensions 0.15×0.10×0.03 mm^3 was selected and mounted on a MiTeGen micromount to be placed in XtaLAB Synergy R, diffractometer equipped with a Cu-rotating anode source and a HyPix-Arc 100 detector. The crystal was kept at T = 200.00(10) K during data collection, and the structure was solved with the ShelXT (Sheldrick, 2015) structure solution program using the Intrinsic Phasing solution method using Olex2 (Dolomanov et al., 2009),[9] as the graphical interface. The model was refined with version 2019/3 of XL (Sheldrick, 2008) using Least Squares minimization.

The molecular cage **C_{60}@1’$_{Zn}$** crystallizes in the monoclinic *C2/c* space group with an asymmetric unit containing half a cage (one porphyrin unit, two linkers), and half **C_{60}.** The crystal structure presents one molecule of DMSO coordinated to each Zn atom through the outer face of the cage (hidden in some of the figures for clarity).

Lateral chains are heavily disordered due to the absence of strong directional supramolecular bonds. This disorder is especially strong for the two chains attached to C24 and C56, where only two of the alternative positions from the first atoms could be located in the electron density map. The fullerene C_{60} molecule also displays a high degree of disorder and was modelled with the aid of geometrical constraints.

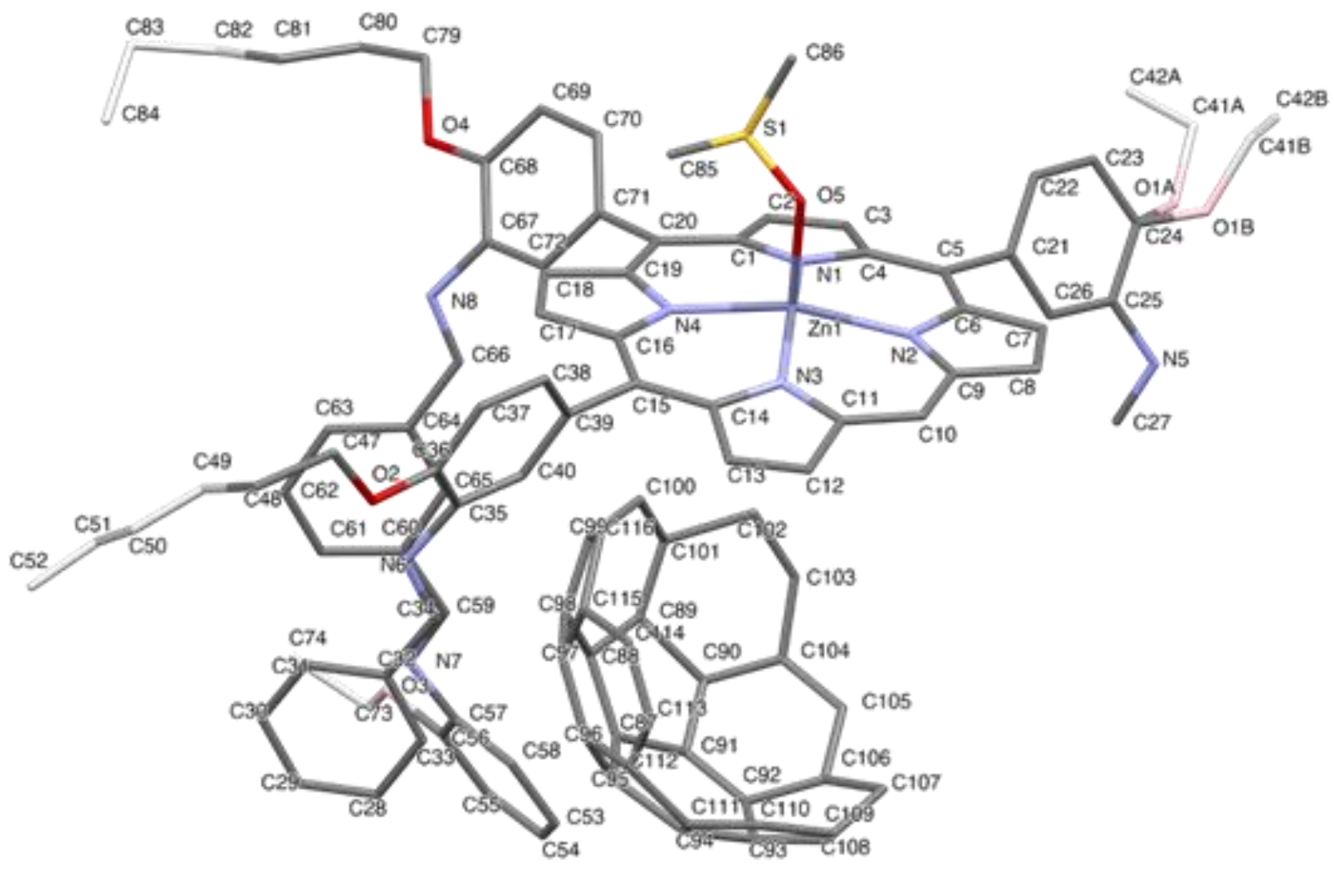

Figure S36. Asymmetric unit of **C_{60}@1’$_{Zn}$** where hydrogen atoms omitted for clarity. Heavily disordered atoms are depicted in a lighter shade to indicate that these positions do not contain all the expected electron density for the corresponding atoms.

Furthermore, **C_{60}@1’$_{Zn}$** cocrystallizes with highly disordered solvent molecules that could not be included in the model; consequently, a solvent mask was calculated using SQUEEZE, and 1208 electrons were found in a volume of 71760 Å^3 in two voids per unit cell. This is consistent with the presence of 4.8 molecules of toluene per asymmetric unit, which account for 1200 electrons.

Table S5. Crystal and structure refinement data of C_{60}@1'$_{Zn}$.

CCDC number	2550583
Empirical formula	$C_{245.5}H_{160}N_{16}O_9S_2Zn_2$
Formula weight	3672.75
Temperature [K]	200.00(10)
Crystal system	monoclinic
Space group	*C*2/*c*
a [Å]	31.1075(4)
b [Å]	20.48669(18)
c [Å]	34.5828(3)
α [°]	90
β [°]	106.8341(11)
γ [°]	90
Volume [Å^3]	21094.8(4)
Z	4
ρ_{calc} [g/cm^3]	1.156
μ [mm^{-1}]	0.952
λ	1.542 (Cu Kα)
2Θ range for data collection/°	2.618 to 68.394
Data/restraints/parameters	19234/1085/1042
Goodness-of-fit on F^2	1.035
R [I>=2σ (I)]	0.1150
wR_2	0.3565

The different views of the solid-state crystal structure of **C_{60}@1'$_{Zn}$** shown in Figure S36 reveal that the cage preserves the so-called *extended* imine conformation exhibited by the empty *jail* **1$_{Zn}$** (please, see our previous work).[1] Moreover, the D_{4h}-symmetry observed for **C_{60}@1'$_{Zn}$** in solution experiments was reduced to pseudo-D_{2h} in the solid state, showing now two kinds of "windows" corresponding to the front (square shaped) and side (rectangular shaped) views. This phenomenon was also experienced by the empty *jail* **1$_{Zn}$**.[1]

Interestingly, the porphyrins present in the empty **1$_{Zn}$** host exhibited a convex shape, with the Zn atoms disposed slightly out of the porphyrin plane towards the inner cavity. In contrast, the porphyrins in the solid state **C_{60}@1'$_{Zn}$** complex swap from convex to concave, placing the Zn atoms towards the exterior of the *jail*, and thus exhibiting now a complementary geometry to the convex surface of **C_{60}**. In accordance, the Zn···Zn distance increased from 12.02 Å to 13.31 Å upon fullerene encapsulation.

The spacefill representation of the crystal structure (Figure S37) shows a perfect geometry and size complementarity between the *jail* and fullerene **C_{60}**. Furthermore, this representation shows that the *jail* "windows" are narrower than the **C_{60}** diameter, which hamper the entry and exit of the fullerene to and from the host, unless the "windows" experience some kind of deformation, thus being responsible for the kinetic effects observed in the solution experiments.

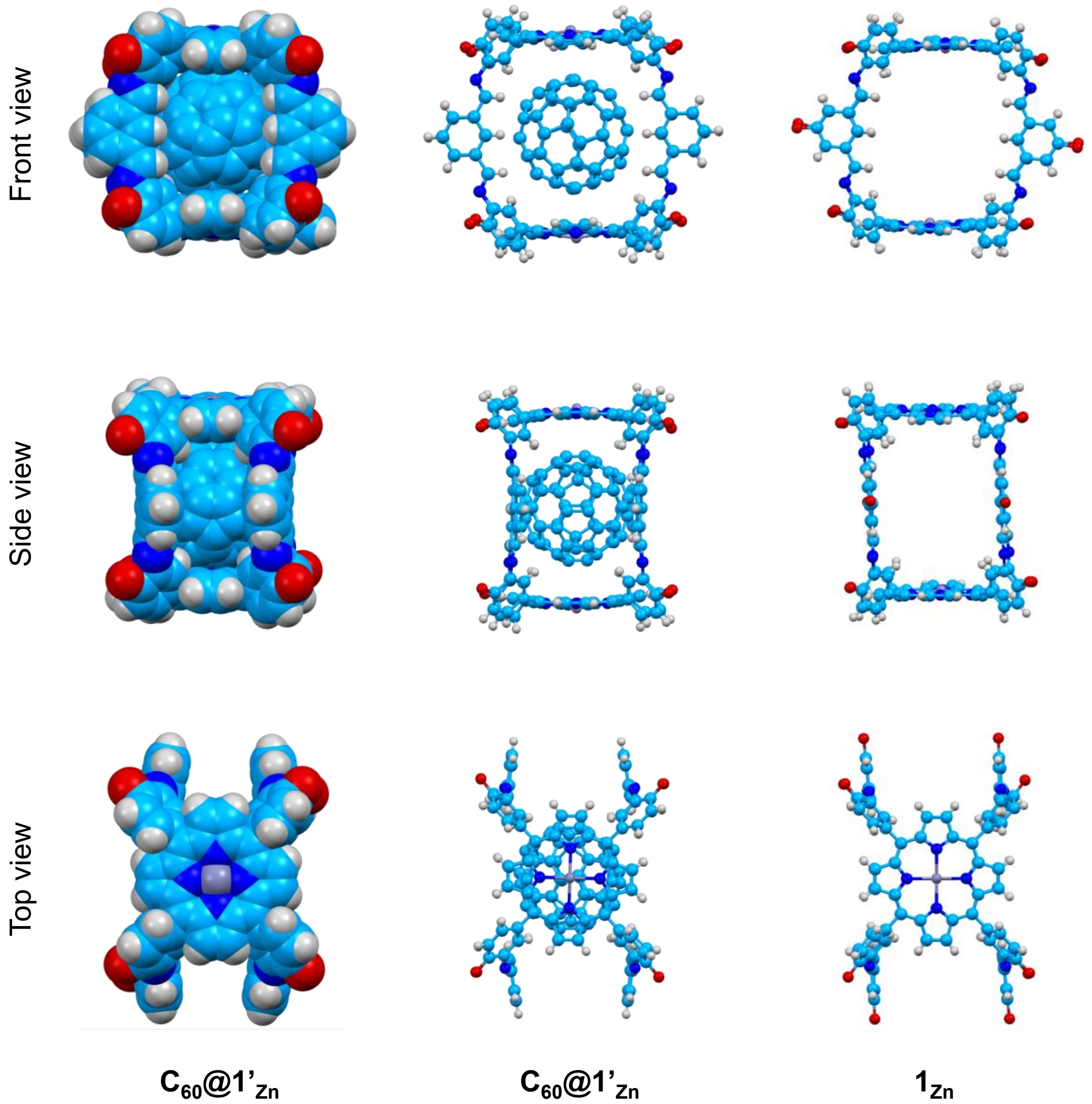

Figure S37. Crystal structure of complex **C_{60}@1'$_{Zn}$**, showing the front, side, and top views in both ball and sticks, as well as spacefill styles. The solubilizing alkyl chains attached to the porphyrin aryl groups, as well as DMSO molecules coordinated to the Zn atoms were omitted for clarity.

The packing of molecular *jail* **C_{60}@1'$_{Zn}$** resulted in a 3D structure where the solubilizing alkyl chains were placed in the inter-space left in between cages, helping to "glue" the structure together (Figure S38). The molecular *jail*s form columnar structures where one cage is on top of another forming a staircase-like structure, probably because the Zn atoms, being coordinated to DMSO molecules, cannot be directly one on top of the other.

View along *a* axis

View along *b* axis

View along *c* axis

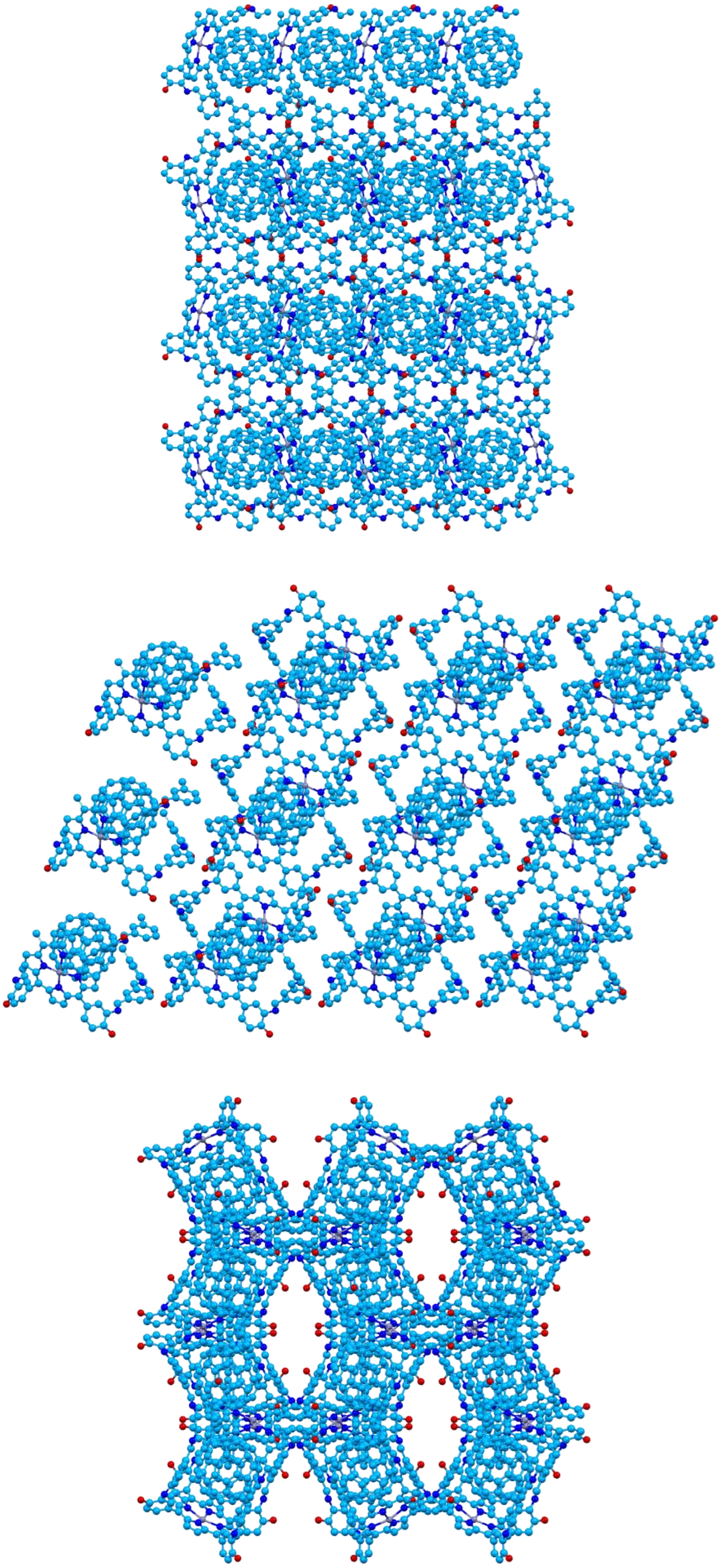

Figure S38. Different packing views of the crystal structure of complex **C_{60}@1'$_{Zn}$**. The solubilizing alkyl chains attached to the porphyrin aryl groups, hydrogen atoms, as well as DMSO molecules coordinated to the Zn atoms were omitted for clarity.

S5. Comparison of C_{60} encapsulation into 2_{Zn} and 3_{Zn}.

Qualitative ^{1}H NMR titration experiments to explore the formation of $\mathbf{2_{Zn}\cdot C_{60}}$ and $\mathbf{3_{Zn}\cdot C_{60}}$ complexes were performed by addition of increasing amounts of $\mathbf{C_{60}}$ to solutions of $\mathbf{2_{Zn}}$ (2.0×10^{-5} M) and $\mathbf{3_{Zn}}$ (1.0×10^{-3} M) in toluene-d_8 at 298 K (Figure S39). Please note that the $\mathbf{2_{Zn}\cdot C_{60}}$ complex showed fast exchange at the NMR timescale while the $\mathbf{3_{Zn}\cdot C_{60}}$ complex exhibited slow exchange.

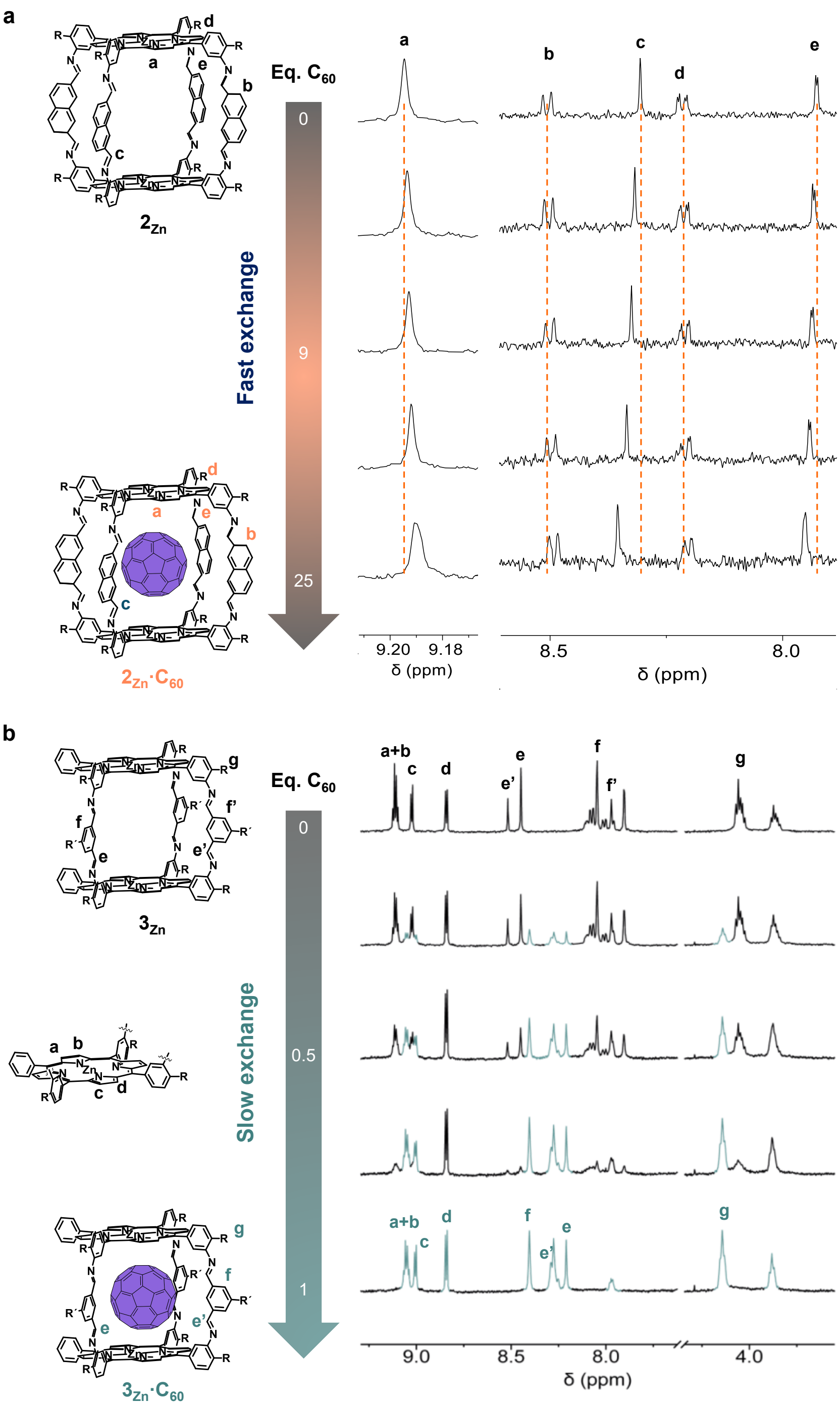

Figure S39. Selected regions of the ^{1}H NMR spectra corresponding to the host-guest titration experiments of (a) $\mathbf{2_{Zn}}$ ($C = 2.0 \times 10^{-5}$ M), and (b) $\mathbf{3_{Zn}}$ ($C = 1.0 \times 10^{-3}$ M) towards fullerene $\mathbf{C_{60}}$ in toluene-d_8 at 298 K. The

most representative proton signals of the empty host are labelled in black, while the host-guest complexes are labelled in orange (**a**) or blue (**b**).

S6. Characterization of C_{60} fullerene encapsulation into 3_{Zn}.

Fullerene encapsulation into $\mathbf{3_{Zn}}$ was studied by ^{1}H NMR (Figure S40; see also Figure S39), ^{13}C NMR (Figure S41), COSY NMR (Figure S42), NOESY NMR (Figure S43), EXSY NMR (Figure S44), MALDI-TOF MS (Figure S45), and UV-vis and fluorescence spectroscopies (Figure S46), comparing the empty host and the supramolecular complex.

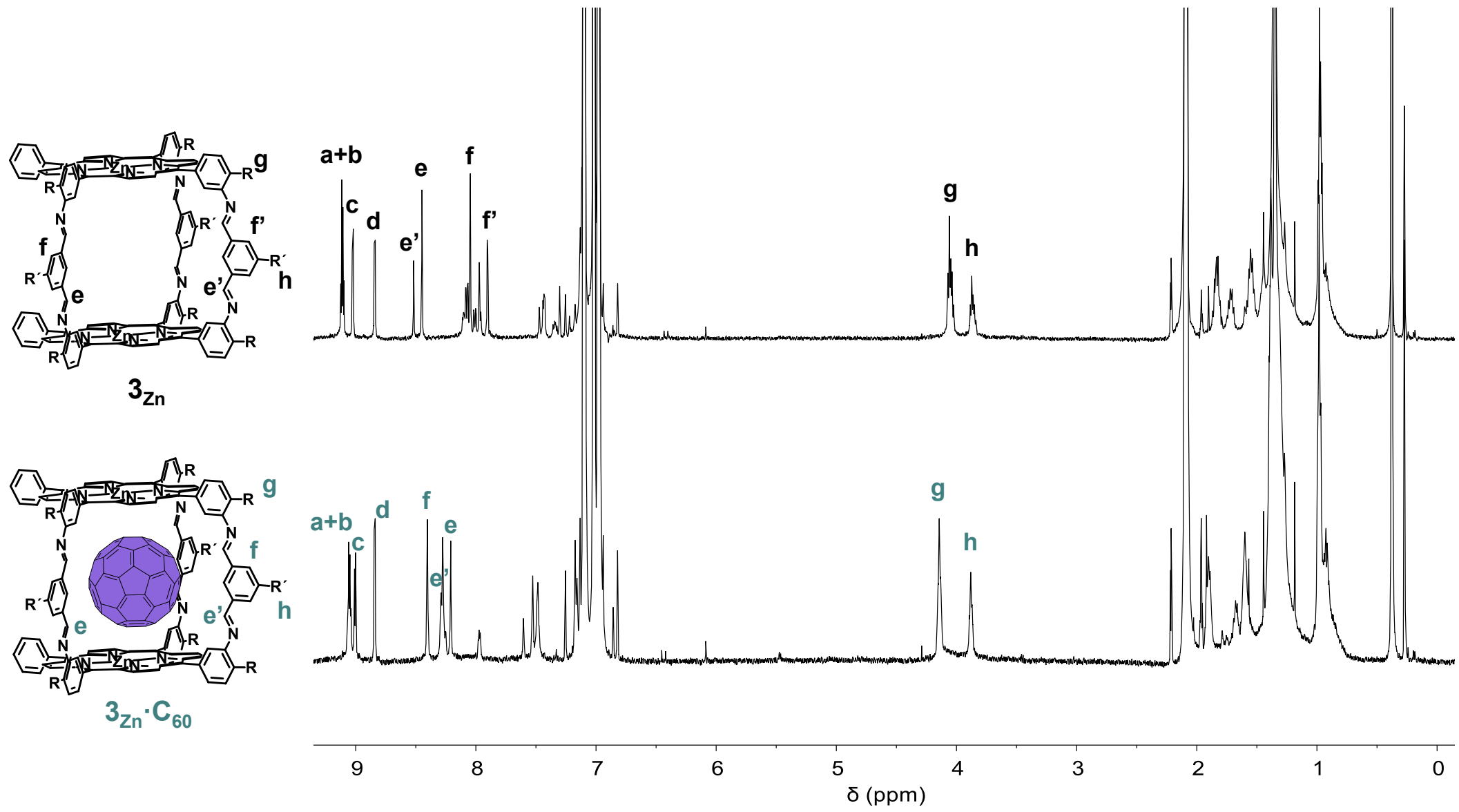

Figure S40. ^{1}H NMR spectra showing the encapsulation of $\mathbf{C_{60}}$ by the $\mathbf{3_{Zn}}$ *basket* (C ($\mathbf{C_{60}}$) = 5.0 × 10^{-4} M; C ($\mathbf{1_{Zn}}$) = 5.0 × 10^{-4} M) in toluene-d_8 at 298 K. The most representative proton signals are labelled in black (for the empty *basket*) and turquoise (for the supramolecular complex).

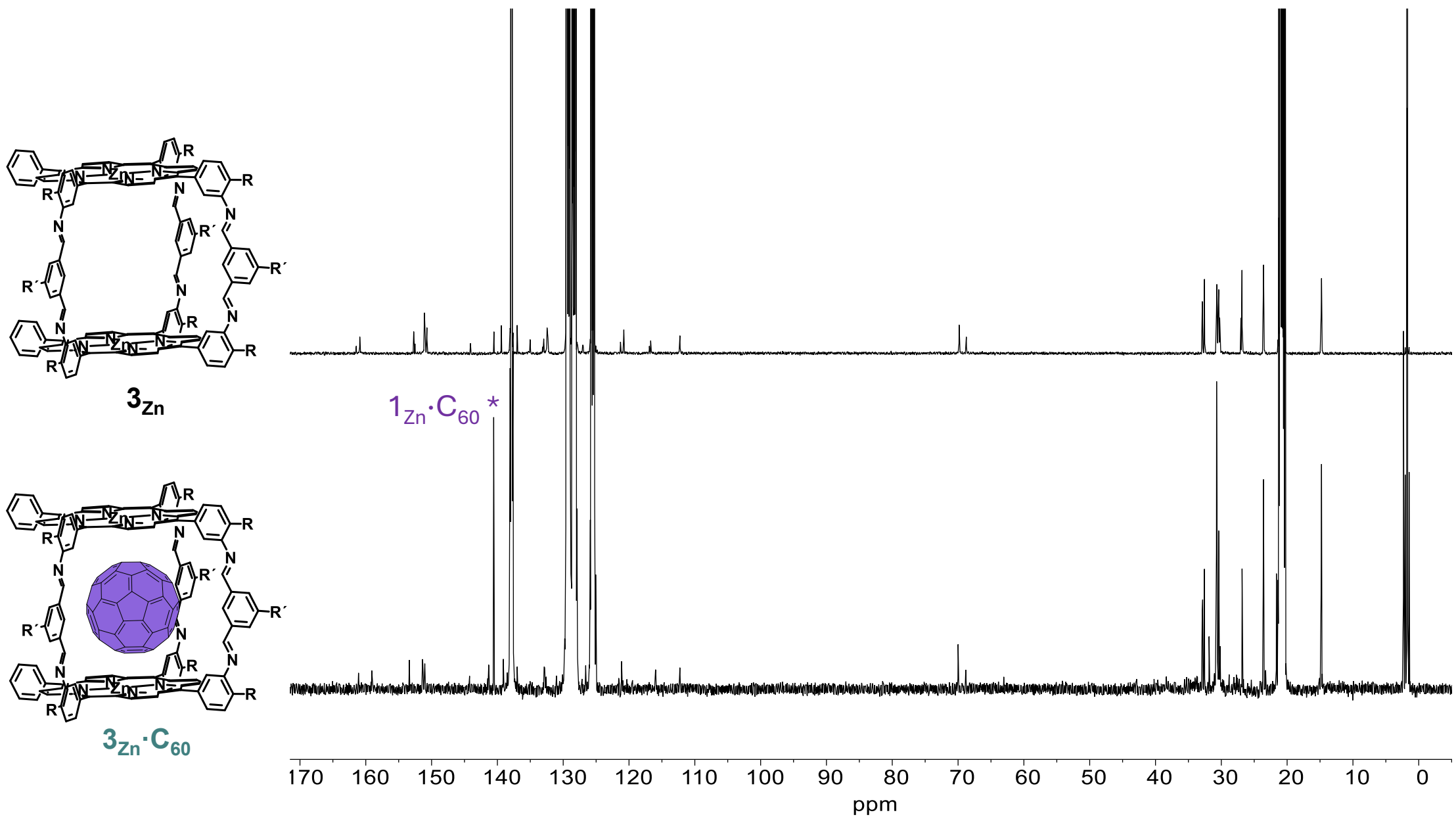

Figure S41. ^{13}C NMR spectra showing the encapsulation of $\mathbf{C_{60}}$ by $\mathbf{3_{Zn}}$ *basket* (C ($\mathbf{C_{60}}$) = 5.0 × 10^{-4} M; C ($\mathbf{1_{Zn}}$) = 5.0 × 10^{-4} M) in toluene-d_8 at 298K. The $\mathbf{C_{60}}$ ^{13}C NMR peak is marked, indicating the fullerene encapsulation, for the $\mathbf{3_{Zn}{\cdot}C_{60}}$ complex.

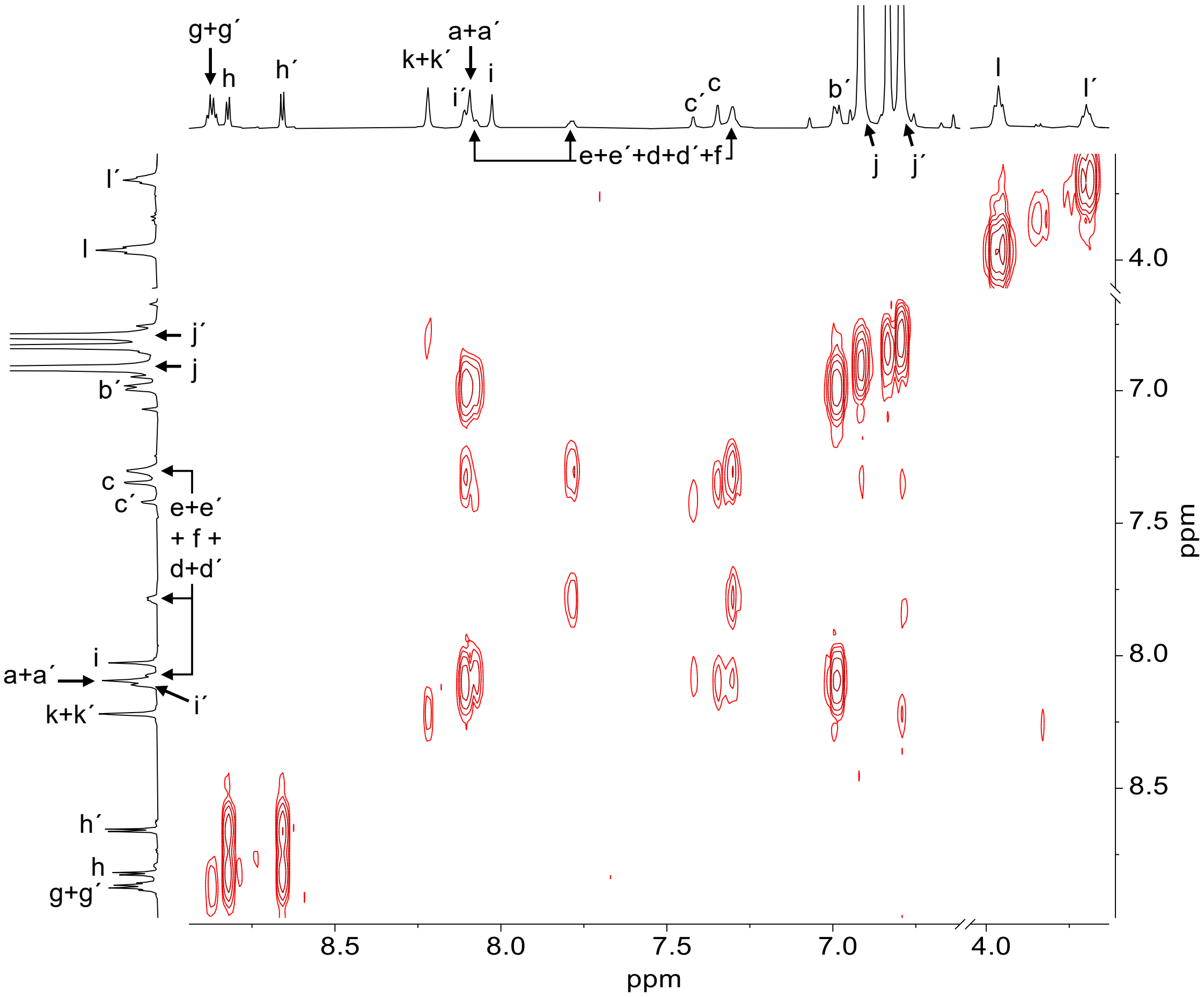

Figure S42. ^{1}H-^{1}H COSY NMR spectrum of $\mathbf{3_{Zn}{\cdot}C_{60}}$ in toluene-d_8 at 298 K.

In the NOESY spectrum of $\mathbf{3_{Zn}{\cdot}C_{60}}$, the imine protons ($H_{i/i'}$) showed cross-peaks with protons $H_{c/c'}$ and $H_{j/j'}$(green dotted circles), but no correlation with the aromatic protons of the linker that point to the outside of the cage cavity was observed, suggesting that $\mathbf{3_{Zn}{\cdot}C_{60}}$ presents exclusively the so-called "extended conformation" (please, see our previous work).[1]

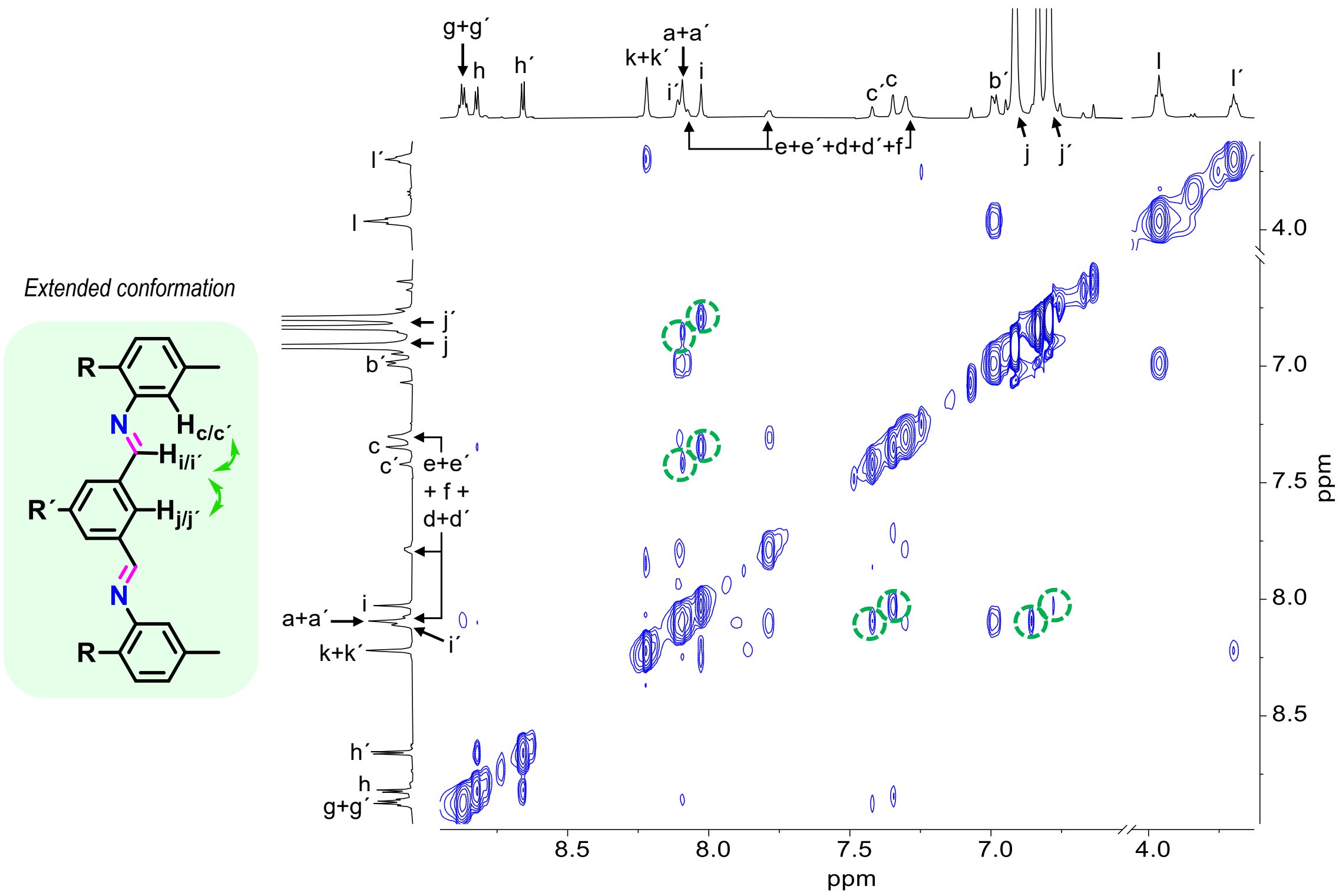

Figure S43. 2D ^{1}H-^{1}H NOESY NMR spectrum of $\mathbf{3_{Zn}{\cdot}C_{60}}$ in toluene-d$_8$ at 298 K.

EXSY Experiments

As shown in the NMR titration of **3Zn** with **C60** (Figure S39b), at substoichiometric amounts of guest, empty and bound *basket*s are clearly detected in slow exchange at the NMR timescale. These slow processes allow the performance of 2D EXSY experiments. Figure S44 shows the non-uniform sampling (NUS) 2D NOESY spectra of a 1:0.61 mixture of **3Zn**:**3Zn·C60** (C_{TOTAL} = 6.0 × 10^{-4} M; C (**3Zn**) = 2.3 × 10^{-4} M; C (**3Zn·C60**) = 3.7 × 10^{-4} M) in toluene D_8 at a mixing time of τ_m = 0.0 s (Figure S44a), τ_m = 3500 ms (Figure S44b). The latter parameter was chosen based on preliminary 1D NOE experiments at different mixing times and already demonstrates the extremely slow nature of the exchange process, in comparison to other supramolecular systems.

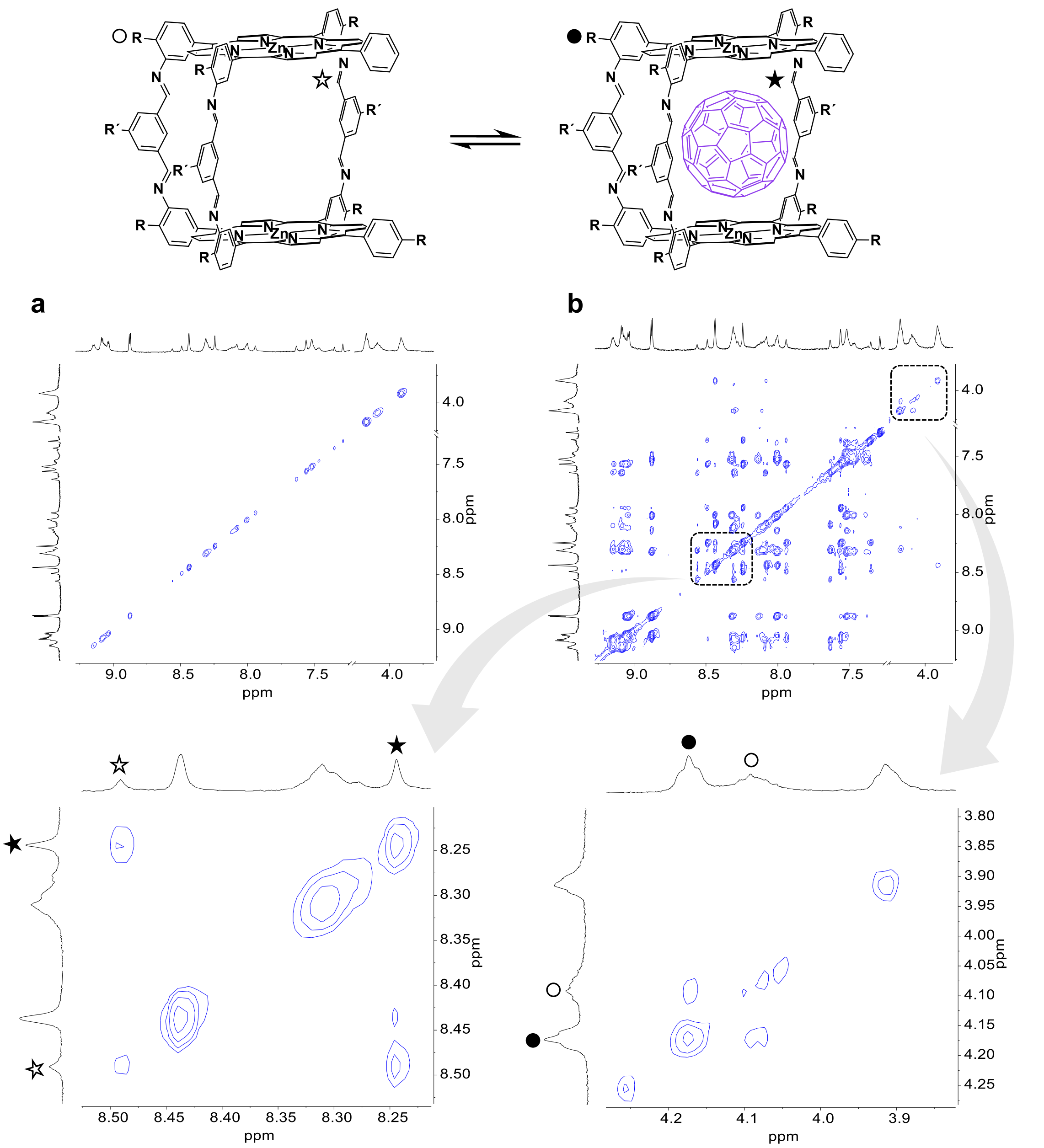

Figure S44. 2D EXSY spectra of **3Zn** with 0.61 eq. of **C60** with a mixing time of (**a**) τ_m = 0.0 s and (**b**) τ_m = 3.5 s in toluene-d_8 at 298 K.

To calculate the exchange rate constants, the signal corresponding to porphyrin alkoxy protons (empy and filled black circles, in the case of the free host and 1:1 complex, respectively) were selected and analyzed at τ_m = 3.5 s and τ_m = 0.0 s using two methods:

a) Using the equations shown below, where *k* is the exchange rate constant, τ_m is the mixing time, X_A and X_B are the molar fractions of molecules in states A and B, respectively, I_{AA} and I_{BB} are the diagonal peak intensities, and I_{AB} and I_{BA} are the cross-peak intensities. A value for *k* was determined, which is the sum of the forward (association; k_1) and backward (dissociation; k_{-1}) pseudo-first order rate constants for the assembly process.

$$k = \frac{1}{\tau_m} ln \frac{r+1}{r-1} \qquad r = 4X_A X_B \frac{I_{AA} + I_{BB}}{I_{AB} + I_{BA}} - (X_A - X_B)^2$$

b) Using the software EXSY Calc (from MestreLab Research), which affords a quantitative analysis of the NMR cross-peaks intensities obtained in EXSY experiments to calculate the magnetization exchange rates of the exchange equilibrium.

The exchange rate constants obtained by both methods at different mixing times in toluene-d_8 at 298 K are similar and are summarized in the following Table S6.

Table S6. Kinetic exchange rate constants (*k*) obtained from methods a and b.

	**Method a**	**Method b**
**τ_m** ms	***k*** s^{-1}	***k*** s^{-1}
3500	**0.181**	**0.211**

The calculated *k* values from both methods are similar, in between 0.18 and 0.21 s^{-1}. An average value of **k_{ex} = 0.20 ± 0.02 s^{-1}** was taken for the **3_{Zn}·C_{60}-3_{Zn}** exchange rate constant in toluene-d_8 at 298 K.

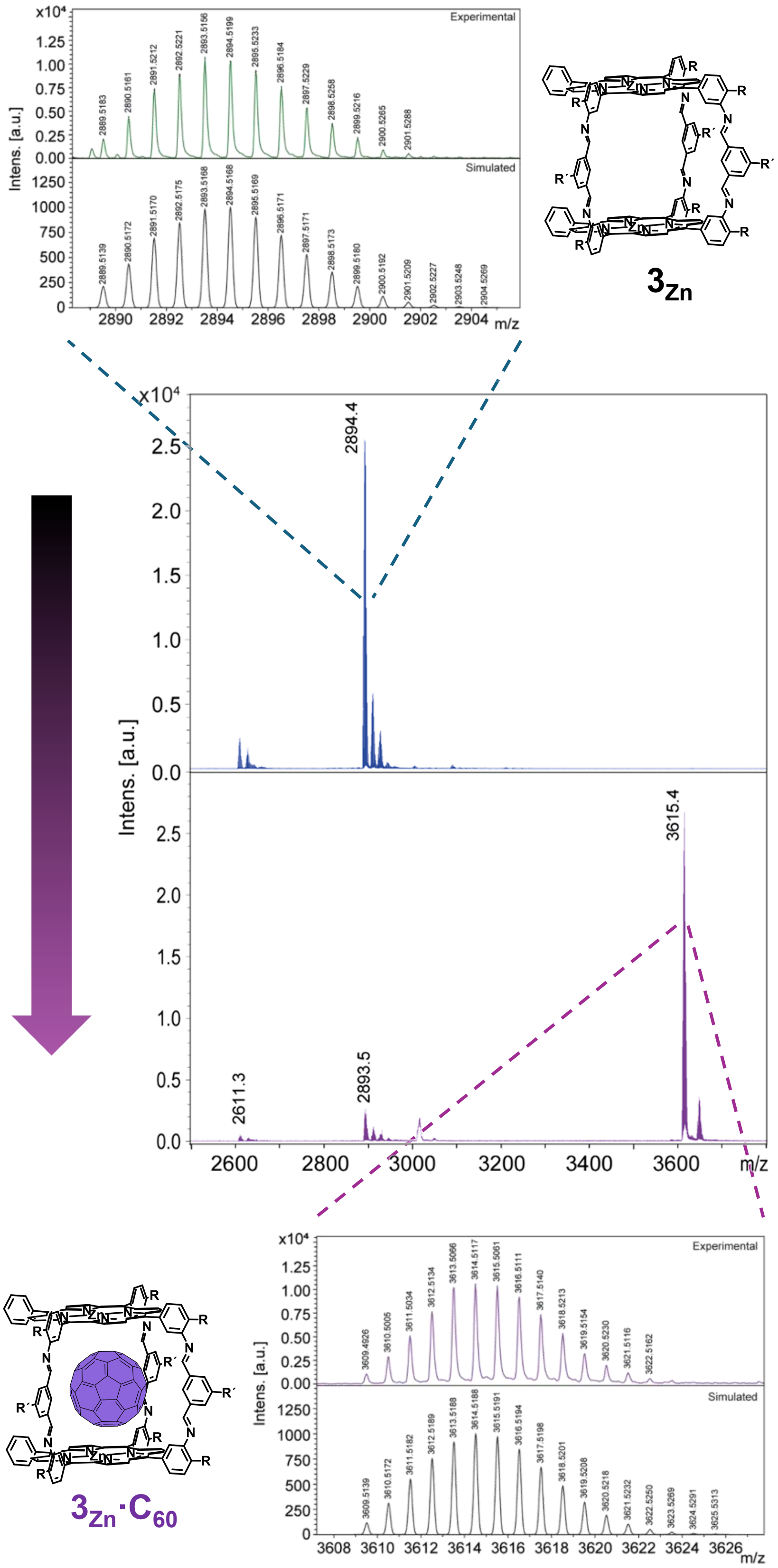

Figure S45. MALDI mass spectra of $\mathbf{3}_{\mathbf{Zn}}$ and $\mathbf{3}_{\mathbf{Zn}}\cdot\mathbf{C}_{\mathbf{60}}$ complex. Peaks at 2894.4 and 3615.4 are the most abundant isotopic peaks and correspond to the molecular formula $C_{184}H_{212}N_{14}O_9Zn_2$ $[M]^+$ ($\mathbf{3}_{\mathbf{Zn}}$) and $C_{244}H_{212}N_{14}O_9Zn_2$ $[M]^+$ ($\mathbf{3}_{\mathbf{Zn}}\cdot\mathbf{C}_{\mathbf{60}}$) $[M]^+$, respectively.

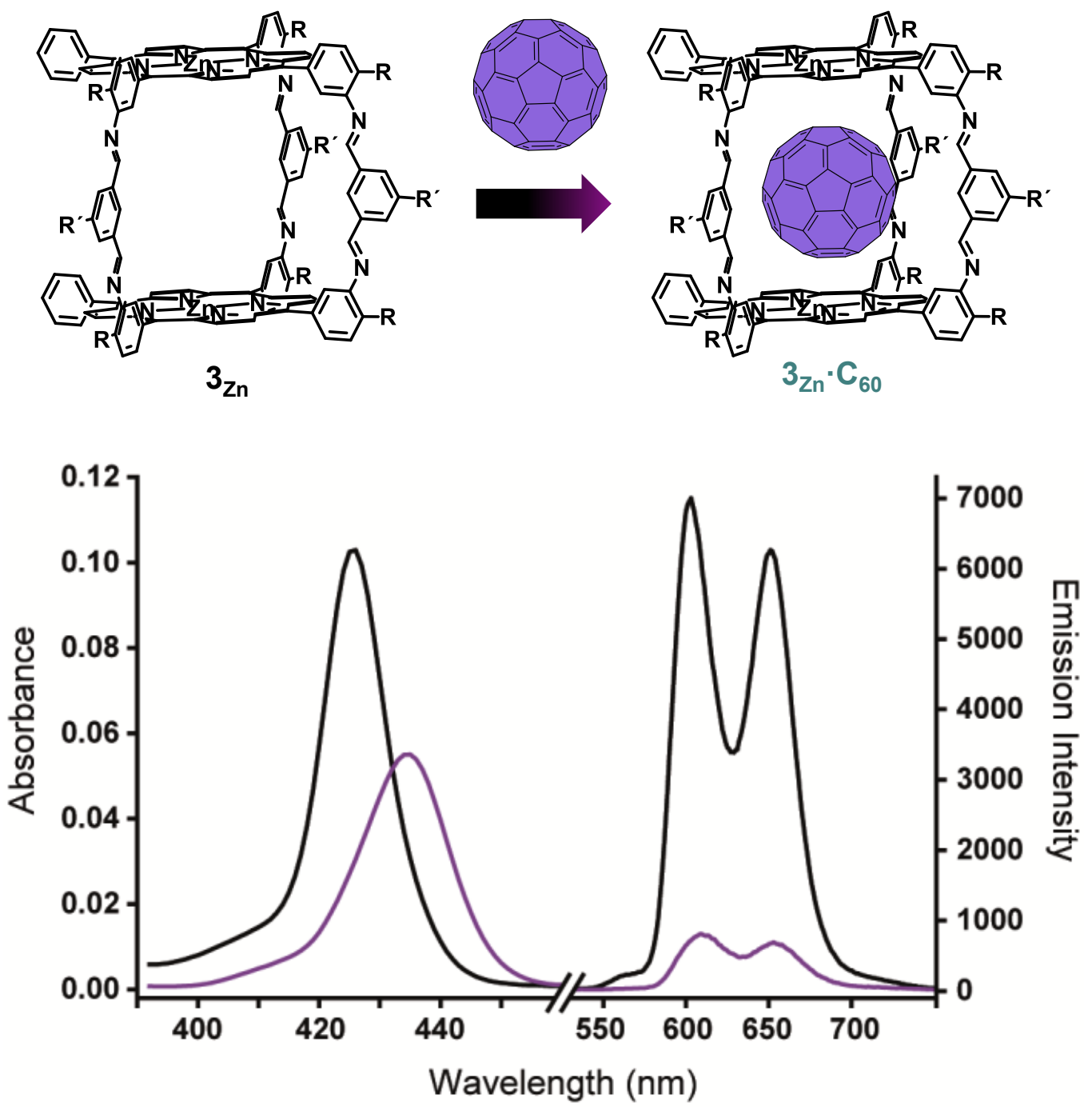

Figure S46. Absorption and emission spectra of **3_{Zn}** (black) and the **3_{Zn}·C_{60}** complex (purple) showing the encapsulation of **C_{60}** by the **3_{Zn}** *basket* (C(**3_{Zn}**) = 1.0 × 10^{-7} M) upon the addition of 7 equivalents of **C_{60}** in toluene at 298 K. Note a bathochromic shift of the porphyrin Soret band as well as an emission quenching upon the encapsulation of **C_{60}**.

S7. Determination of the partial and global reaction orders for C_{60} fullerene encapsulation into $\mathbf{1_{Zn}}$.

Two independent sets of four encapsulation experiments were carried out to determine the kinetic partial reaction order with respect to *jail* 1_{Zn} and C_{60}. The kinetic experiments were performed at 403 K and monitored by ^{1}H NMR at 298 K in toluene-d_8. In the first set of experiments different amounts of *jail* **1**$_{\mathbf{Zn}}$ (1.3, 0.66, 0.33, and 0.16 equivalents) were employed maintaining constant the concentration of $\mathbf{C_{60}}$ (1.5 × 10^{-3} M) to calculate the reaction order with respect to *jail* **1**$_{\mathbf{Zn}}$ (Figure S47a), while in the second set different amounts of $\mathbf{C_{60}}$ (3, 1.5, 0.75, and 0.37 equivalents) were employed maintaining constant the concentration of *jail* **1**$_{\mathbf{Zn}}$ (1.0 × 10^{-3} M) to calculate the reaction order with respect to $\mathbf{C_{60}}$ (Figure S47b).

Then, by plotting the growing concentration of $\mathbf{C_{60}@1_{Zn}}$, calculated from the conversion values obtained by ^{1}H NMR, *vs* time and performing a linear fitting of the data, the initial formation rates of $\mathbf{C_{60}@1_{Zn}}$ were obtained for each experiment. Finally, these values were represented *vs* [$\mathbf{1_{Zn}}$] (Figure S47c) or *vs* [$\mathbf{C_{60}}$] (Figure S47d). The data was fitted to a linear equation affording good R-square values (0.988 and 0.999, respectively). These results demonstrate that the encapsulation of $\mathbf{C_{60}}$ by *jail* $\mathbf{1_{Zn}}$ occurs with partial reaction orders of 1 with respect to both *jail* $\mathbf{1_{Zn}}$ and $\mathbf{C_{60}}$. Therefore, the sum of the partial orders affords a global reaction order of 2.

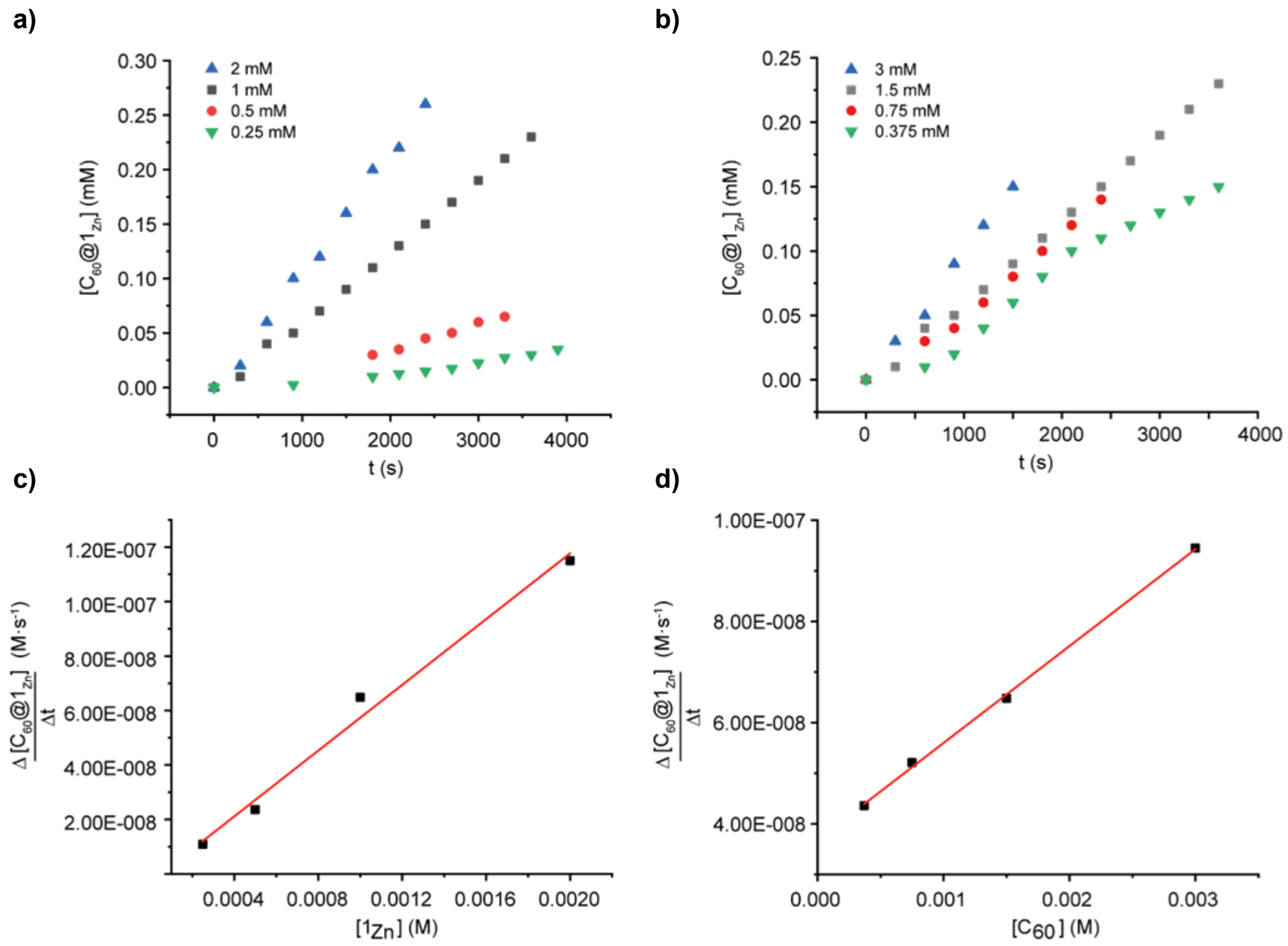

Figure S47. Determination of partial reaction order in the formation of supramolecular complex $\mathbf{C_{60}@1_{Zn}}$. Representation of product concentration ($\mathbf{C_{60}@1_{Zn}}$) *vs* time, at a constant concentration of: a) $\mathbf{C_{60}}$ (1.5 × 10^{-3} M) and b) $\mathbf{1_{Zn}}$ (1.0 × 10^{-3} M), where the slope is the initial formation rate. Note that the experiments were monitored up to 15% conversion. The concentration of *jail* $\mathbf{1_{Zn}}$ and $\mathbf{C_{60}}$ employed in a) and b), respectively, are included in the inset legend. Plot of the formation rate of $\mathbf{C_{60}@1_{Zn}}$ *vs* [$\mathbf{1_{Zn}}$] (c) and [$\mathbf{C_{60}}$] (d) at a constant concentration of $\mathbf{C_{60}}$ and $\mathbf{1_{Zn}}$, respectively.

S8. Determination of the kinetic constants (*k*) at different temperatures and the activation energy barrier (E_a) for C_{60} fullerene encapsulation into $\mathbf{1_{Zn}}$.

Temperature-dependent kinetic experiments were performed in sealed NMR tubes charged with *jail* $\mathbf{1_{Zn}}$ (0.5×10^{-3} M) and fullerene $\mathbf{C_{60}}$ (0.5×10^{-3} M) in *o*-xylene-d_{10}. The use of toluene-d_8 afforded similar qualitative results, but required longer periods of NMR time (*i.e.*, several days at constant heating), which limited the practical viability of these experiments in this solvent. The NMR tubes in *o*-xylene-d_{10} were heated at 373, 388, 393, 498, and 408 K in NMR magnet, monitoring by ^{1}H NMR the encapsulation evolution respect to the time of reaction (Figure S48a). The $\mathbf{C_{60}@1_{Zn}}$: $\mathbf{1_{Zn}}$ ratio was calculated from the NMR signal correspond to the *β*-pyrrolic protons of the porphyrins, allowing to know the concentration of all both *jail* $\mathbf{1_{Zn}}$ and $\mathbf{C_{60}}$ at each time.

To calculate the kinetic constants from the data obtained in these experiments, a second order rate law must be employed (A + B → C), as demonstrated in section S6. However, since the experiments were performed using identical concentrations of both reagents $\mathbf{1_{Zn}}$ and $\mathbf{C_{60}}$, the reaction can be treated as a 2A → C process, allowing the use of the following equation:[10]

Eq. (1): $\frac{1}{[A]} = kt + \frac{1}{[A]_0}$

Where $[A]_0$ is the initial concentration of $\mathbf{1_{Zn}}$ and $\mathbf{C_{60}}$, and [A] is the concentration of $\mathbf{1_{Zn}}$ and $\mathbf{C_{60}}$ at each time during the encapsulation process. Therefore, by plotting 1/[A] vs time (in seconds) and fitting the data to a linear equation, the kinetic constant can be determined as the slope of the resulting line. These data are collected in Table S7.

The experimental activation parameters ($\Delta H^‡$ and $\Delta S^‡$) were calculated using the Eyring equation:[10]

Eq. (2): $ln(\frac{k}{T}) = ln(\frac{\kappa k_B}{h}) - \left(\frac{\Delta H^‡}{R}\right)\left(\frac{1}{T}\right) + \frac{\Delta S^‡}{R}$

Where *k* is the kinetic constant, *h* is Planck´s constant (J·s^{-1}), k_B is Boltzmann´s constant (J·K^{-1}), $\boldsymbol{\kappa}$ is the transmission coefficient ($\boldsymbol{\kappa}$ = 1, as an approximation), T is the temperature (K), and *R* is the universal gas constant (J·K^{-1}·mol^{-1}). Thus, the graphical representation of ln(*k*/T) versus 1/T (Figure S48c and Table S7), known as the Eyring plot, and by fitting the data to a linear equation (r^2 = 0.995), provided $\Delta H^‡$ and $\Delta S^‡$ values of 79.56 kJ·mol^{-1} and –79.22 J·mol^{-1}·K^{-1}, respectively, calculated from the slope (-9569.70 ± 400.96) K and the intercept (14.12 ± 1.02).

Transition state free energy values ($\Delta G^{\neq}$) shown in Figure S48d, were calculated by using the following equation:[10]

Eq. (3): $\Delta G‡ = -RT \ln(\frac{kh}{\kappa k_B})$

All the variables shown in Eq. 3 were defined above.

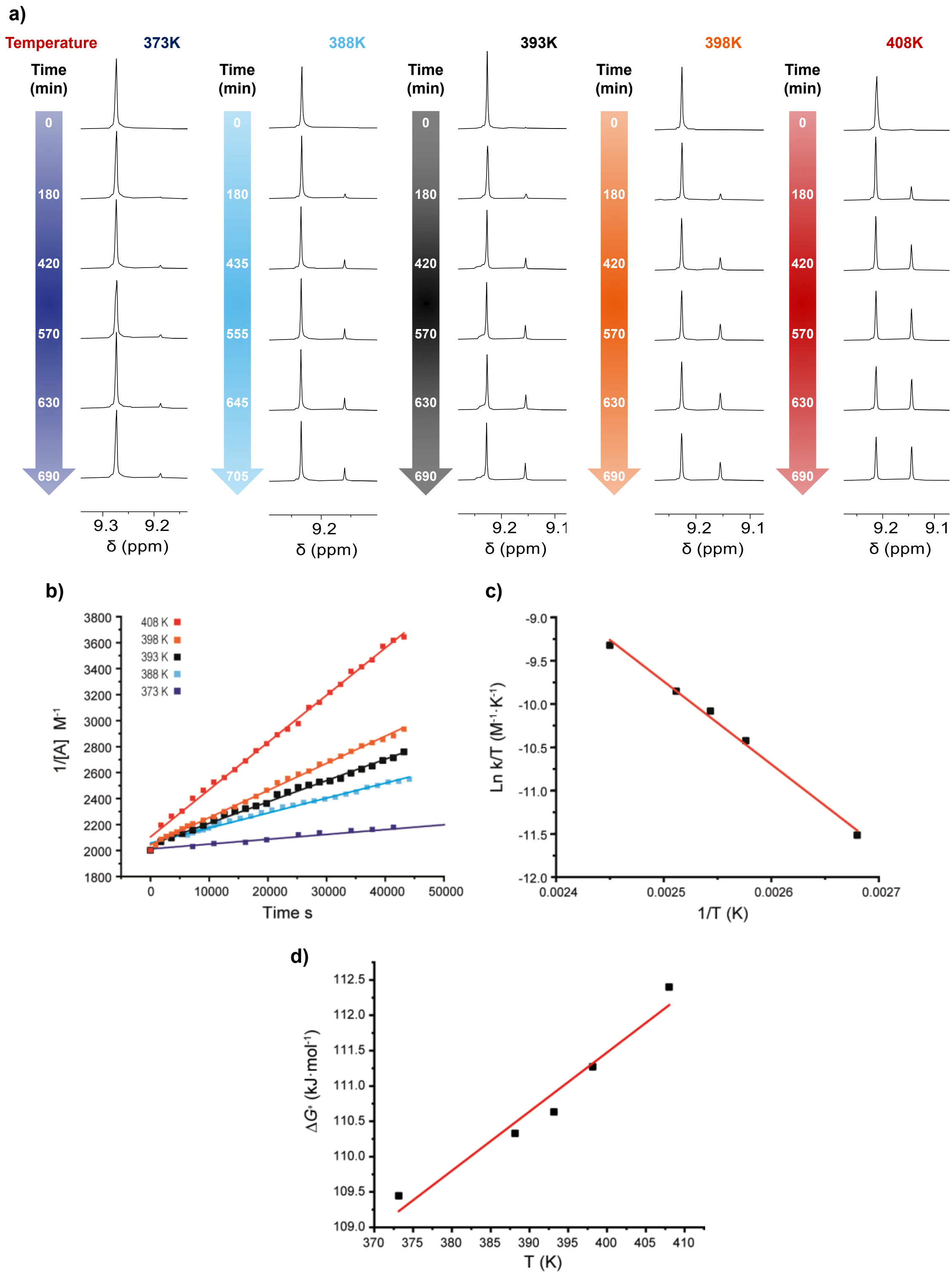

Figure S48. a) Representative ^{1}H NMR spectra of a selected region showing the progressive encapsulation of $\mathbf{C_{60}}$ by $\mathbf{1_{Zn}}$ *jail* (C ($\mathbf{C_{60}}$) = 0.5 × 10^{-3} M; C ($\mathbf{1_{Zn}}$) = 0.5 × 10^{-3} M) in *o*-xylene-D_{10} after heating at 373, 388, 393, 398, and 408 K. b) Comparison of the kinetic data following Eq. 1 in the experiments carried out at different temperatures. c) Eyring plot used to calculate the activation parameters ($\Delta H^{‡}$ = **79.56 kJ·mol^{-1}** and $\Delta S^{‡}$ = **–79.56 J·mol^{-1}·K^{-1}**) of the encapsulation process leading to $\mathbf{C_{60}@1_{Zn}}$. d) Plot showing the dependence of the transition state free energy $\underline{\Delta} G^{‡}$ as a function of temperature T (K).

Table S7. Kinetic data obtained from the temperature-dependent experiments shown in Figures S48a,b. These data were employed to build Figures S48c,d.

**T** (K)	***k*** (M^{-1} s^{-1})	**Error**	**Pearson´s r**	**1/T** (K^{-1})	**Ln(*k/T*)** ($s^{-1}\cdot K^{-1}$)	**Δ*G*‡** ($kJ\cdot mol^{-1}$)
**373**	0.00372	0.00011	0.994	0.00268	-11.51	109.44
**388**	0.0115	0.0002	0.994	0.00258	-10.43	110.33
**393**	0.0164	0.0003	0.997	0.00254	-10.08	110.63
**398**	0.0209	0.0002	0.998	0.00251	-9.85	111.27
**408**	0.0364	0.0004	0.998	0.00245	-9.32	112.40

S9. Well-Tempered Metadynamics Simulations

Free energy surfaces (FES) for the unbinding processes of the host:guest complexes were computed using well-tempered metadynamics (WT-MetaD).[11,12] Simulations were carried out for 400 ns using the high-performance molecular dynamics software GROMACS 2025.3[13] interfaced with the PLUMED 2.9 open-source library.[14,15] All simulations were initiated from the bound configurations, in which $\mathbf{C_{60}}$ is encapsulated within the *jail*, *cage*, and *basket* hosts. The systems were energy-minimized using a steepest descent algorithm followed by an equilibration step under NPT conditions in a simulation box of 40 × 80 × 100 $Å^3$ solvated with explicit toluene molecules and under periodic boundary conditions. The collective variable (CV) used to calculate the profile was defined as the distance between the Zn–Zn centroid of the host and the center of mass (COM) of $\mathbf{C_{60}}$. This coordinate was selected to describe the host:guest dissociation pathway. The metadynamics gaussian bias potentials were deposited every 1 ps with an initial height of 1 kJ·mol^{-1} and a width of 0.03 nm. A bias factor of 8 was employed for $\mathbf{C_{60}@1_{Zn}}$, whereas a lower bias factor of 5 was used for $\mathbf{2_{Zn}·C_{60}}$ and $\mathbf{3_{Zn}·C_{60}}$. This small bias factor allows for a more controlled bias deposition in order to preserve the barrierless dissociation behaviour experimentally observed for $\mathbf{2_{Zn}·C_{60}}$ and $\mathbf{3_{Zn}·C_{60}}$, thus avoiding the over-smoothing of the profile in the host:guest dissociation. Finally, the FES was reconstructed using the *sum_hills* utility implemented in PLUMED (see Figure S49), and convergence was evaluated by monitoring the FES for the host:guest dissociation along the simulation time every 50 s (see Figure S50). After 300 ns the free energy profiles remained mainly constant and convergence is achieved.

The *jail*, *cage*, *basket*, $\mathbf{C_{60}}$, and toluene molecules were parametrized following the JOYCE3 protocol[16] where bonded parameters were derived by fitting to the quantum mechanical Hessian matrix calculated at the B3LYP-D3/cc-pVDZ DFT level, ensuring accurate reproduction of bond stretching, angle bending, and improper torsional contributions. Moreover, partial atomic charges were obtained from electrostatic potential (ESP) calculations at the same level of theory, computed using the Merz–Singh–Kollman scheme[17,18] as implemented in Gaussian 16. The last intermolecular terms, the non-bonded interactions, were described using Lennard–Jones potentials with van der Waals parameters taken from the OPLS force field database.[19]

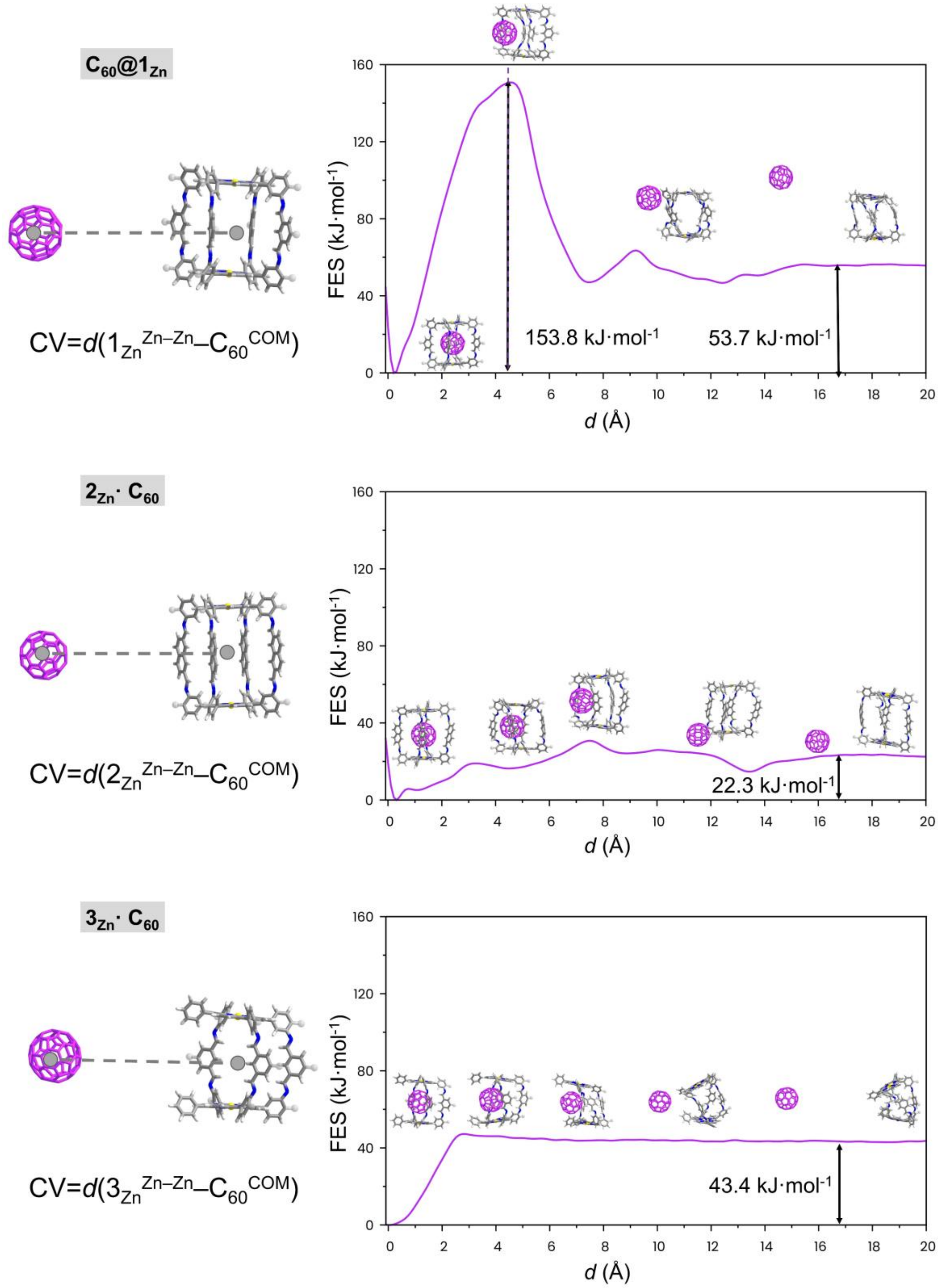

Figure S49. (Left) Representation of the collective variable (CV), defined as the distance between the center of mass (COM) of $\mathbf{C_{60}}$ and the Zn-Zn centroid of the host (*jail*, *cage*, or *basket*). (Right) Free energy surface (FES) for the unbinding process of $\mathbf{C_{60}@1_{Zn}}$, $\mathbf{2_{Zn}{\cdot}C_{60}}$, and $\mathbf{3_{Zn}{\cdot}C_{60}}$ computed from 400 ns WT-MetaD simulations along the defined CV.

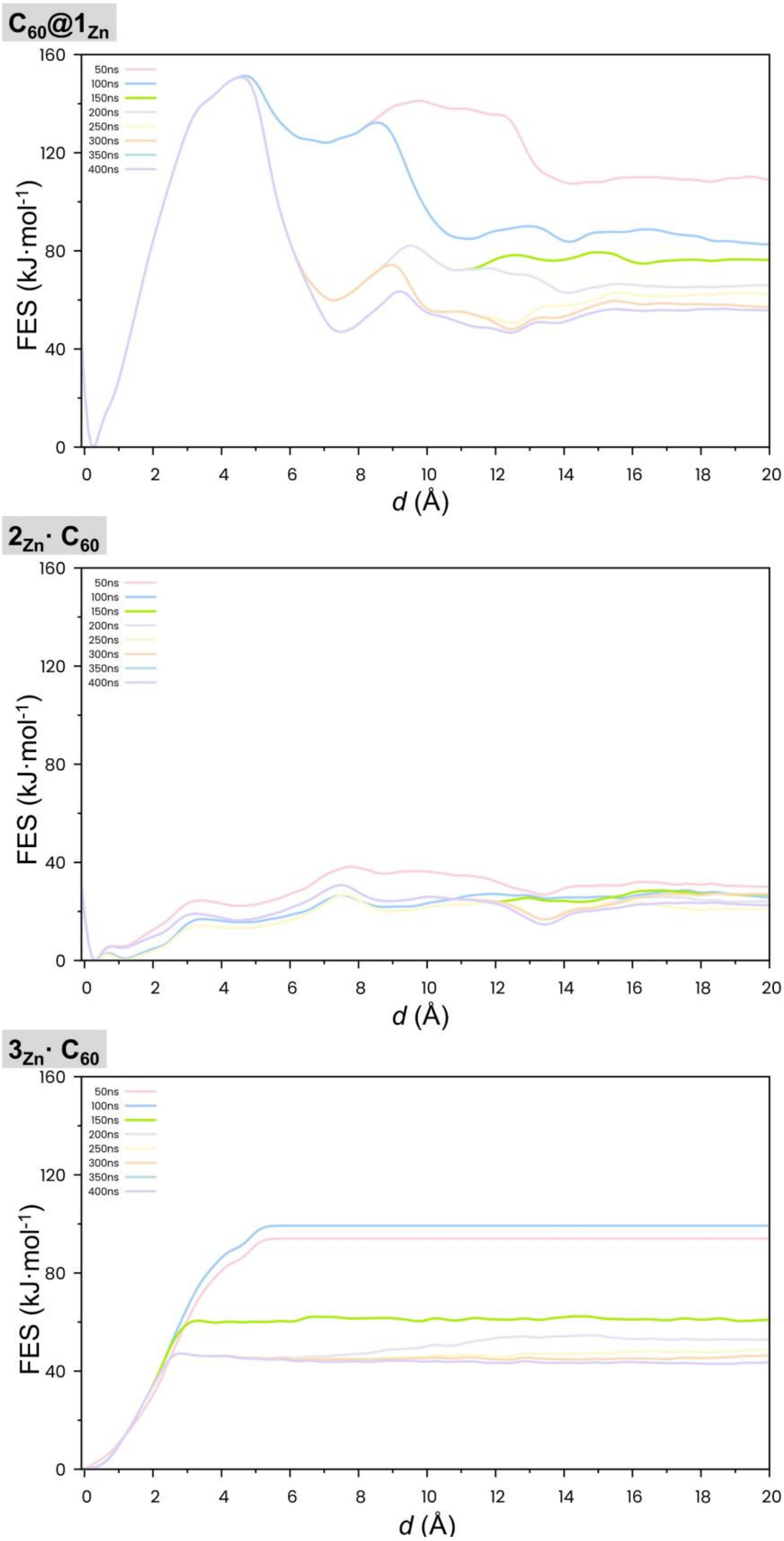

Figure S50. Representation of the free energy surface (FES) over the distance between the Zn–Zn centroid and the COM of $\mathbf{C_{60}}$, every 50 ns during a simulation of 400 ns for the $\mathbf{C_{60}@1_{Zn}}$, $\mathbf{2_{Zn}{\cdot}C_{60}}$ and $\mathbf{3_{Zn}{\cdot}C_{60}}$ complexes to show the convergence of the dissociation process.

S10. C_{60} fullerene encapsulation into 1_{Zn} activated via acid catalysis with TFA.

A. NMR experiments at high concentrations.

Catalyst-dependent kinetic experiments were performed in sealed NMR tubes charged with *jail* $\mathbf{1_{Zn}}$ (1.0×10^{-3} M) and fullerene $\mathbf{C_{60}}$ (1.5×10^{-3} M), and different amounts of trifluoroacetic acid (TFA: 0.10, 0.25, 0.40, and 0.50 eq.) at 298 K in toluene-d_8. The encapsulation conversion was determined by ^{1}H NMR, monitoring the signal corresponding to the β-pyrrolic protons of the porphyrins (Figure S51). The reaction profiles obtained from these experiments were qualitatively compared in Figure S52, showing that the presence of TFA accelerates the encapsulation with respect to the non-catalyzed encapsulation from weeks (Figure S24) to almost 1 hour (0.10 eq. of TFA) or even a few minutes when the TFA amount was increased (0.50 eq. of TFA). It should be noted that leaving these mixtures with high TFA content for prolonged times (i.e. several days) in solution results in the slow degradation of $\mathbf{1_{Zn}}$.

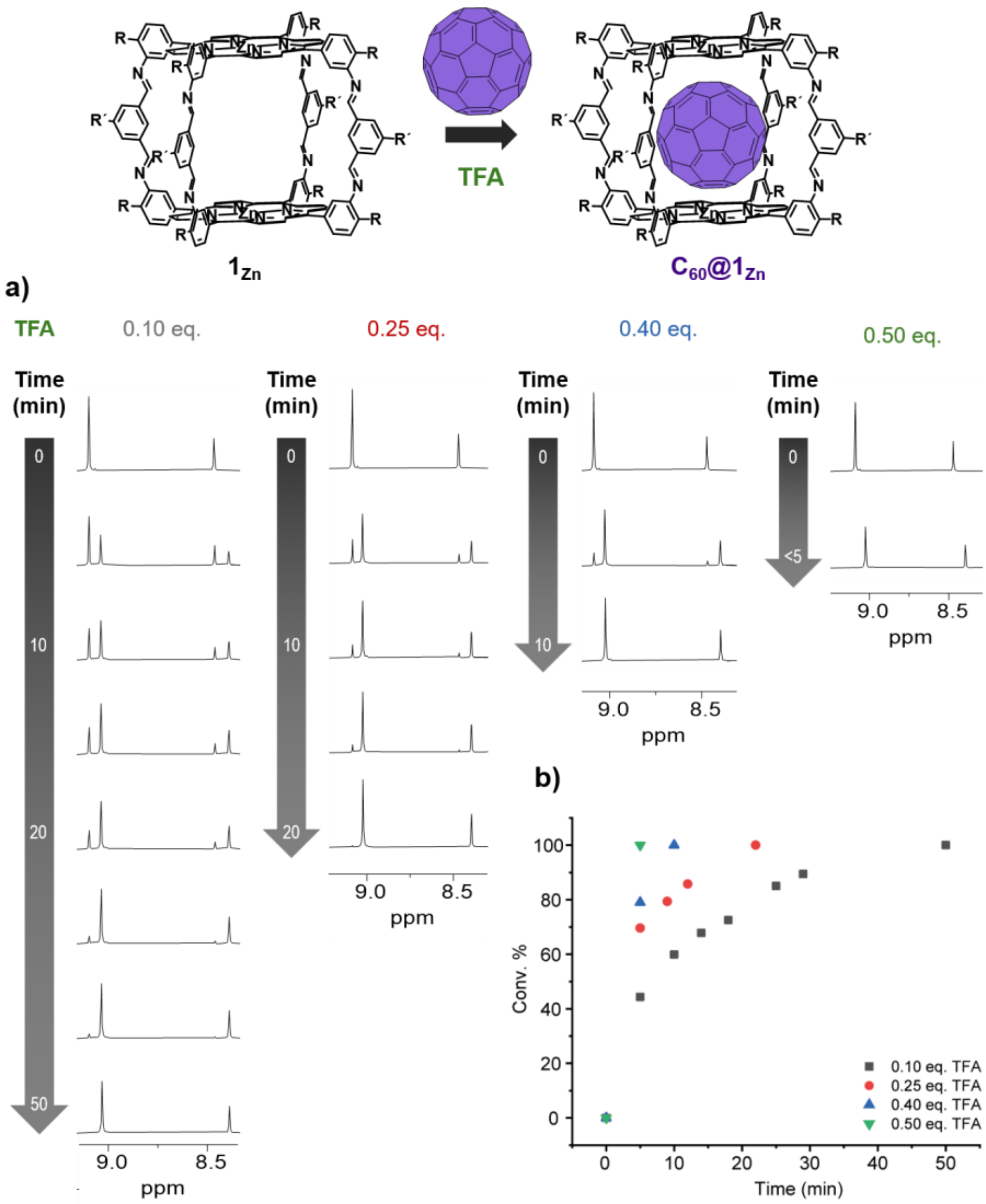

Figure S51. Fullerene C_{60} encapsulation activated by the addition of TFA as catalyst. a) ^{1}H NMR spectra in toluene-d_8 showing the progressive encapsulation of $\mathbf{C_{60}}$ by the $\mathbf{1_{Zn}}$ *jail* (C ($\mathbf{C_{60}}$) = 1.5×10^{-3} M; C ($\mathbf{1_{Zn}}$) = 1.0×10^{-3} M) in the presence of 0.1, 0.25, 0.4, or 0.5 equiv. of TFA at 298 K. b) Encapsulation profiles (conversion vs time) are shown in the graph.

***B. UV-vis experiments at low concentrations*.**

Catalyst-dependent kinetic experiments under diluted conditions were performed by combining equimolar amounts of *jail* $\mathbf{1_{Zn}}$ and fullerene $\mathbf{C_{60}}$ (1.0×10^{-6} M), and adding different amounts of TFA (1.0, 2.5, 5.0, 7.5, and 10.0 eq.) at 298 K in spectroscopic grade toluene. The progress of the reaction was monitored by UV-vis spectroscopy (Figure S52a-e). The reaction profiles obtained from these experiments, compared in Figure S52f, revealed that, as happened at higher concentrations of the reagents, the reaction rate increases with a higher content of TFA in solution.

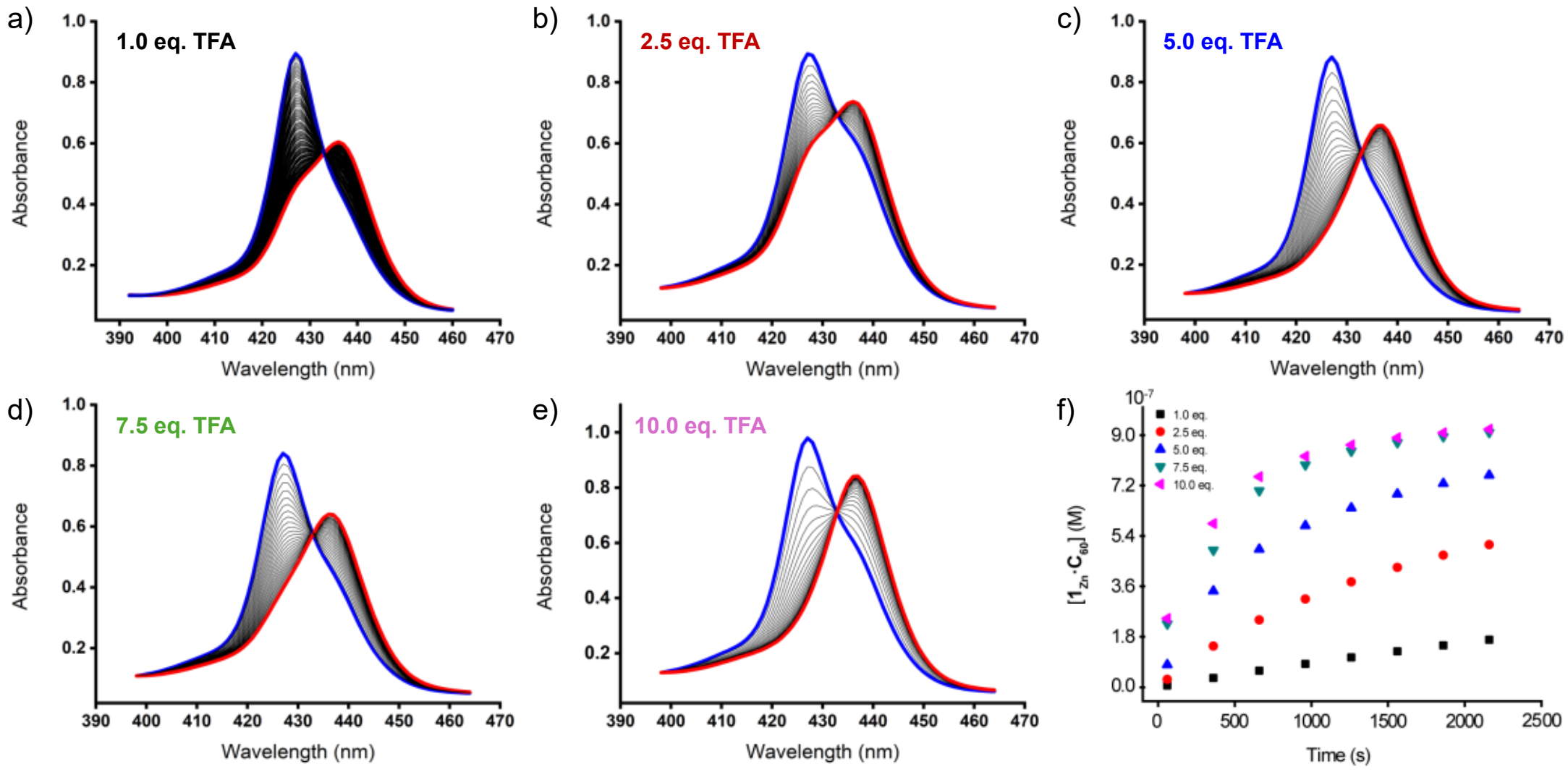

Figure S52. a-e) Kinetic experiments as a function of the TFA equivalents added, conducted at low concentrations of both $\mathbf{1_{Zn}}$ and $\mathbf{C_{60}}$ (1.0×10^{-6} M), and monitored by UV-Vis spectroscopy in toluene at 298 K. f) Reaction profiles monitoring the formation rate of $\mathbf{C_{60}@1_{Zn}}$ as a function of the amount of TFA in solution.

Next, temperature-dependent kinetic experiments under diluted conditions were performed by combining equimolar amounts of *jail* $\mathbf{1_{Zn}}$ and fullerene $\mathbf{C_{60}}$ (1.0×10^{-6} M) and 2.5 equivalents of TFA in spectroscopic grade toluene. The temperature of the experiments was fixed at 293, 298, 308, and 318 K and the progress of the reaction monitored by UV-vis spectroscopy (Figures S53a-d). The reaction profiles compared in Figure S53e indicate an increment of the reaction rate with increasing temperature.

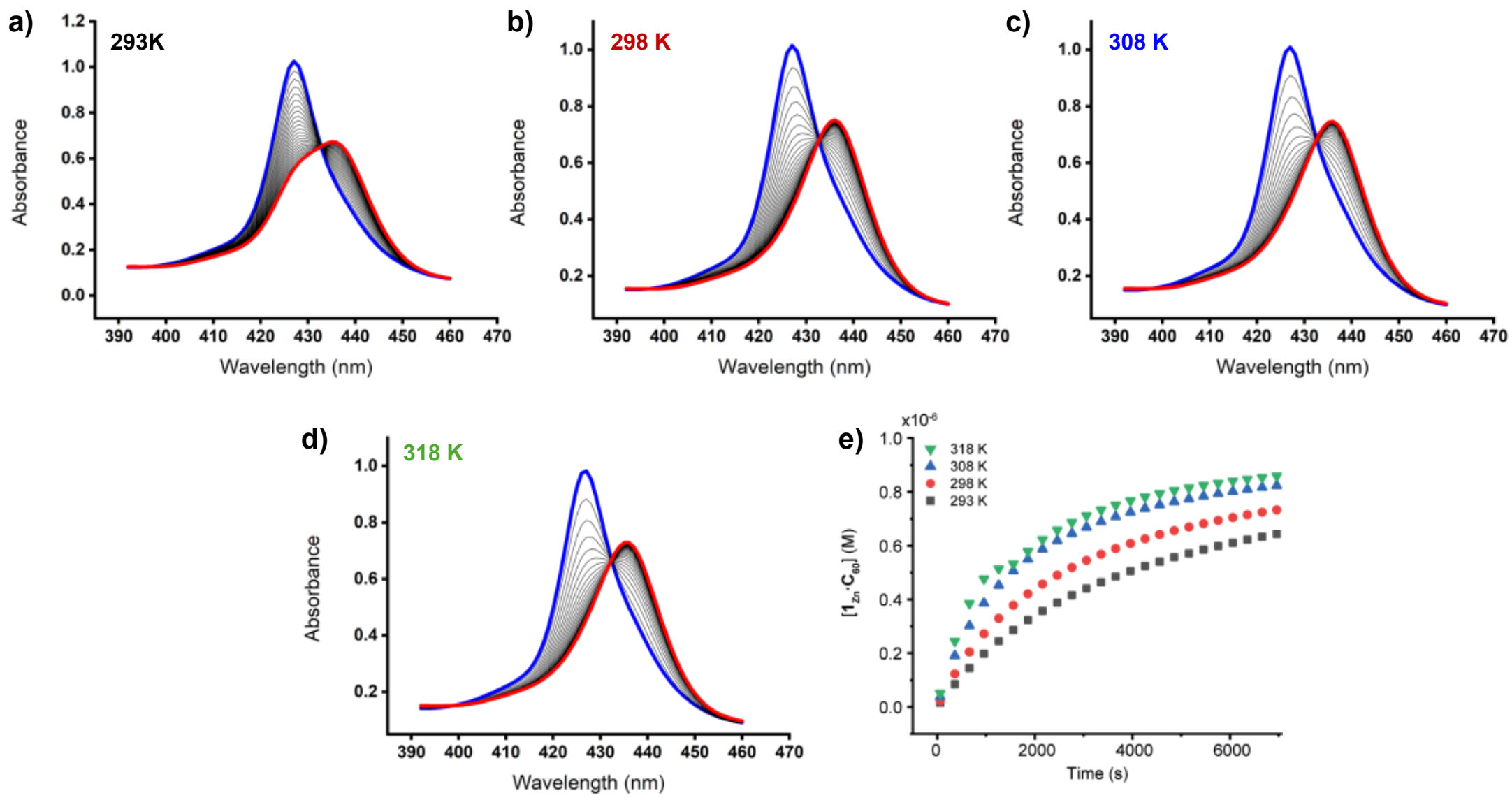

Figure S53. a-d) Temperature-dependent Kinetic experiments in the presence of TFA ($1.0 \cdot \times 10^{-6}$ M), conducted at low concentrations of both $\mathbf{1_{Zn}}$ and $\mathbf{C_{60}}$ (1.0×10^{-6} M and $1.5 \times 10^{-}$ M, respectively), monitored by UV-Vis spectroscopy in toluene. e) Reaction profiles showing the formation rate of $\mathbf{C_{60}@1_{Zn}}$ as a function of temperature.

S11. Thermodynamic characterization of Host-Guest complex: $\mathbf{3_{Zn}{\cdot}C_{60}}$.

The binding of $\mathbf{C_{60}}$ to $\mathbf{3_{Zn}}$ was studied in toluene at 298 K at high concentrations by ^{1}H NMR (Figure S54; see also Figures S39-46) and at low concentrations by absorption (Figure S55) or fluorescence emission (Figure S56) spectroscopy. The binding constant is too high to be calculated by NMR, and every addition of $\mathbf{C_{60}}$ produced quantitative amounts of the $\mathbf{3_{Zn}{\cdot}C_{60}}$ complex.

^{1}H NMR Titrations

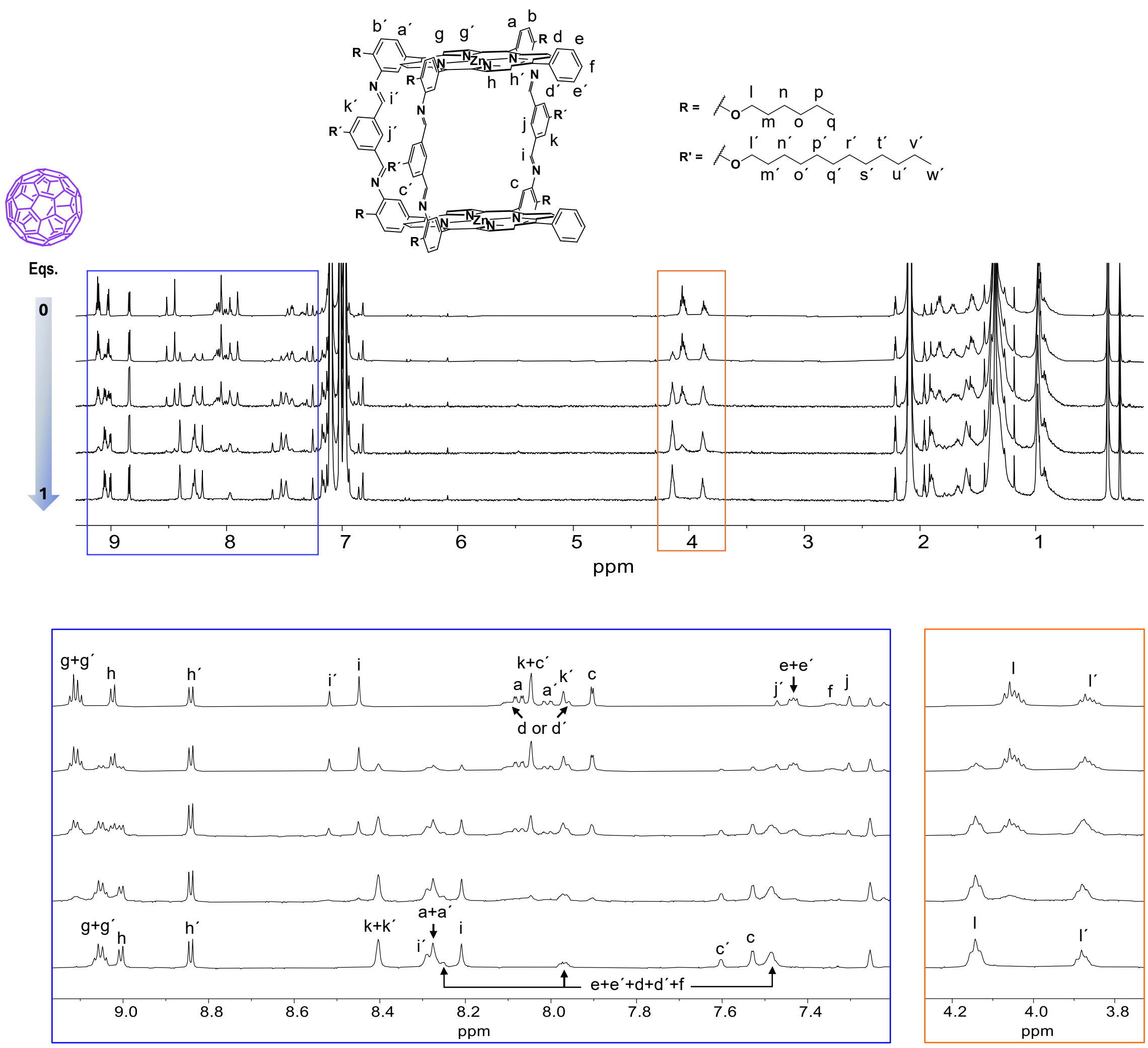

Figure S54. ^{1}H NMR changes recorded along the titration of $\mathbf{3_{Zn}}$ with increasing amounts of $\mathbf{C_{60}}$ in toluene-d_8 at 298 K. Note the slow exchange behavior observed between the unbound species and the supramolecular complex.

UV-vis and Fluorescence Titrations

However, the binding constant could be calculated through absorption (Figure S55) or emission fluorescence (Figure S56) titration measurements at lower concentrations, which allowed us to determine the binding constant between **3$_{Zn}$** and **C$_{60}$** in toluene by fitting the binding isotherms to a 1:1 model.

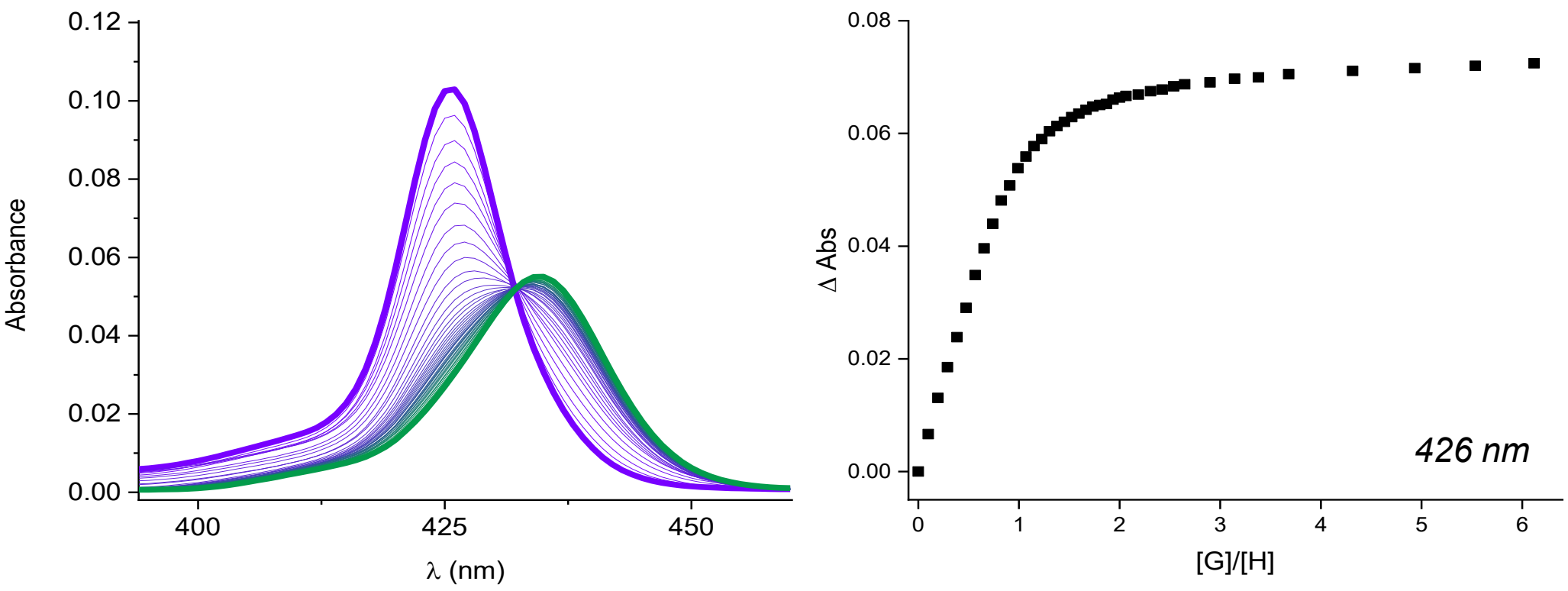

Figure S55. UV-Vis changes recorded along the titration of **3$_{Zn}$** at a constant concentration of 1.0 × 10^{-8} M with increasing amounts of **C$_{60}$** in toluene at 298 K.

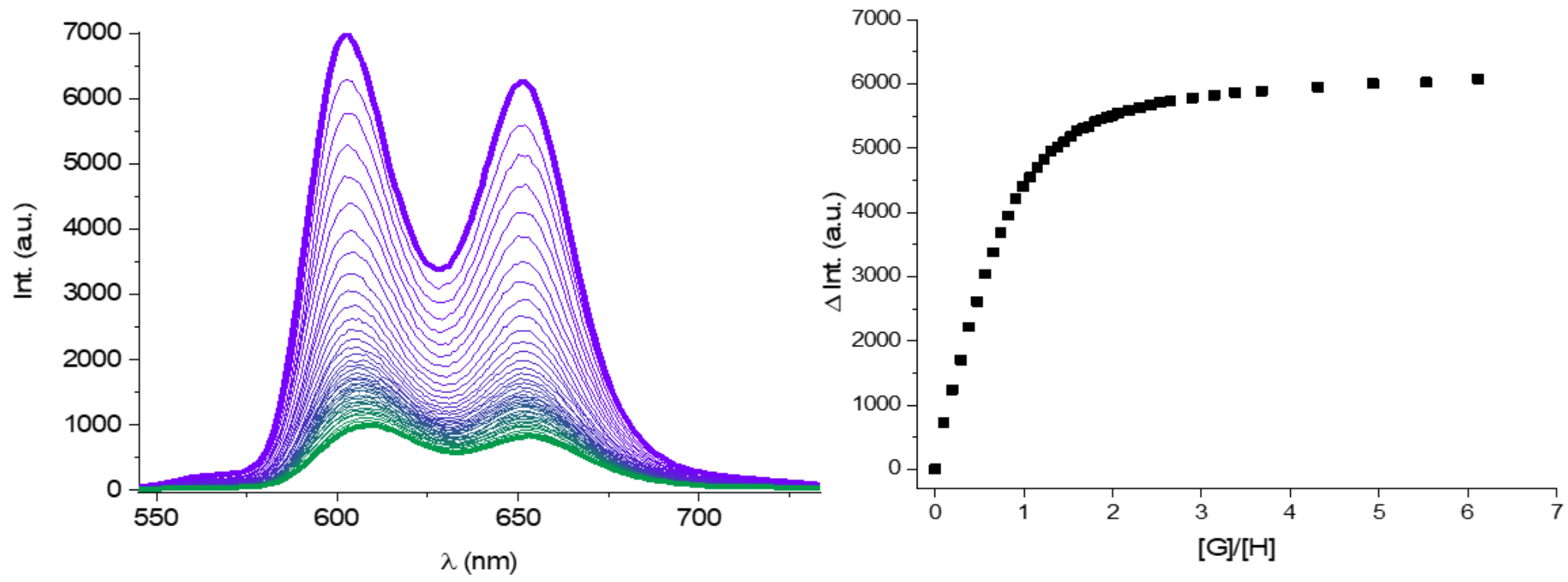

Figure S56. Fluorescence emission changes recorded along the titration of **3$_{Zn}$** at a constant concentration of 1.0 × 10^{-8} M with increasing amounts of **C$_{60}$** in toluene at 298 K. λ_{exc} = 432 nm.

The quantitative analysis of these two titration experiments led to an average value of $K_{C_{60}}$ = 9.4 ± 0.5 × 10^{7} M^{-1} for the complexation with **3$_{Zn}$** in toluene.

S12. Thermodynamic characterization of the 3_{Zn}·*bipy* host-guest complex.

The binding of ***bipy*** to $\mathbf{3_{Zn}}$ was studied in toluene at 298 K at high concentrations by ^{1}H NMR (Figures S57-59) and at low concentrations by fluorescence spectroscopy (Figure S60). The binding constant is too high to be calculated at the NMR concentration regime, and every addition of ***bipy*** produced quantitative amounts of the $\mathbf{3_{Zn}}$·***bipy*** complex (Figure S57).

^{1}H NMR Titration

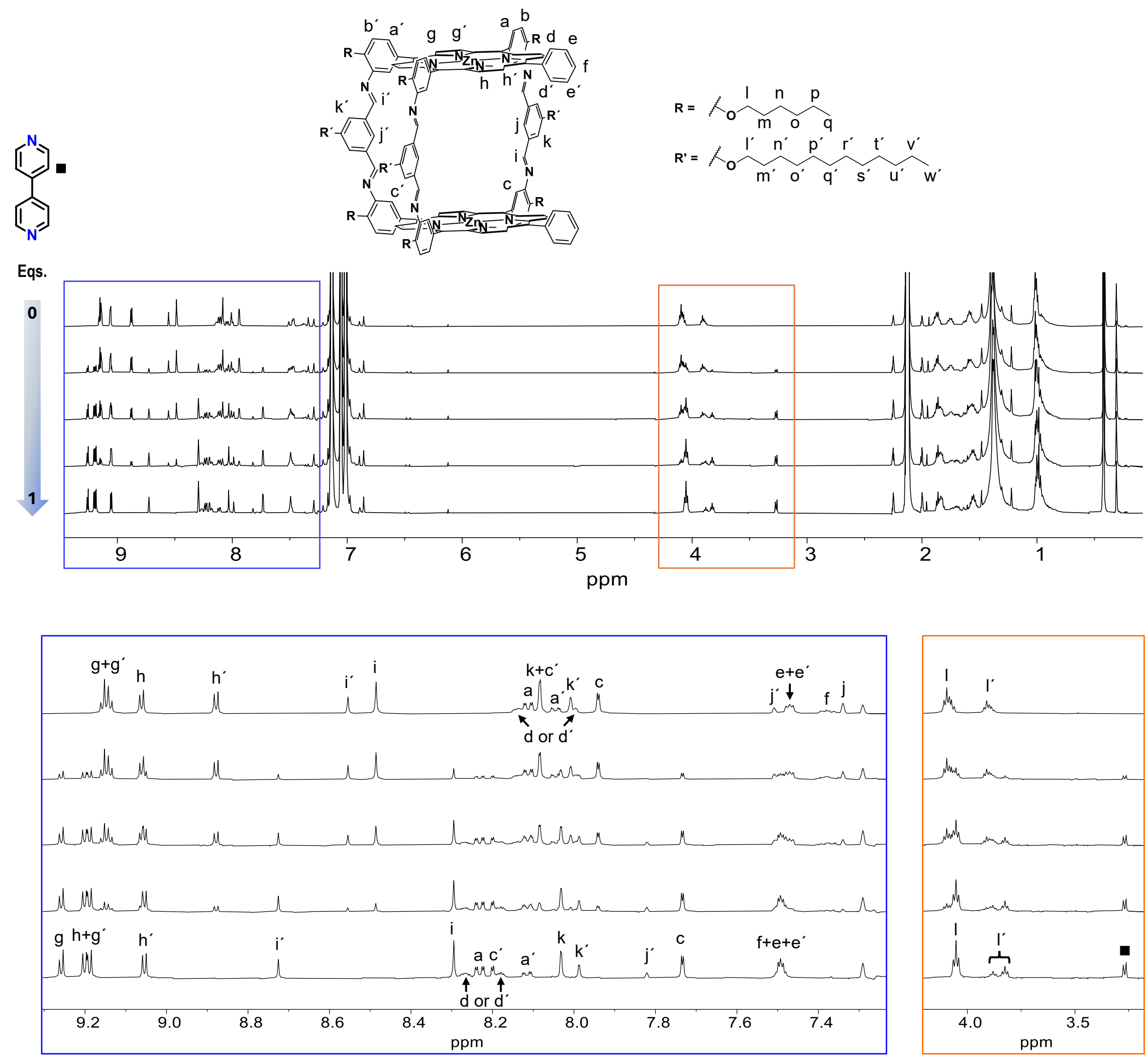

Figure S57. ^{1}H NMR changes recorded along the titration of $\mathbf{3_{Zn}}$ with increasing amounts of ***bipy*** in toluene-d_8 at 298 K.

Assignment of 3_{Zn}·*bipy* ^{1}H NMR signals – COSY and NOESY NMR experiments.

In order to assign correctly the ^{1}H NMR spectrum of complex **3_{Zn}·*bipy***, COSY (Figure S58) and NOESY (Figure S59) experiments were carried out.

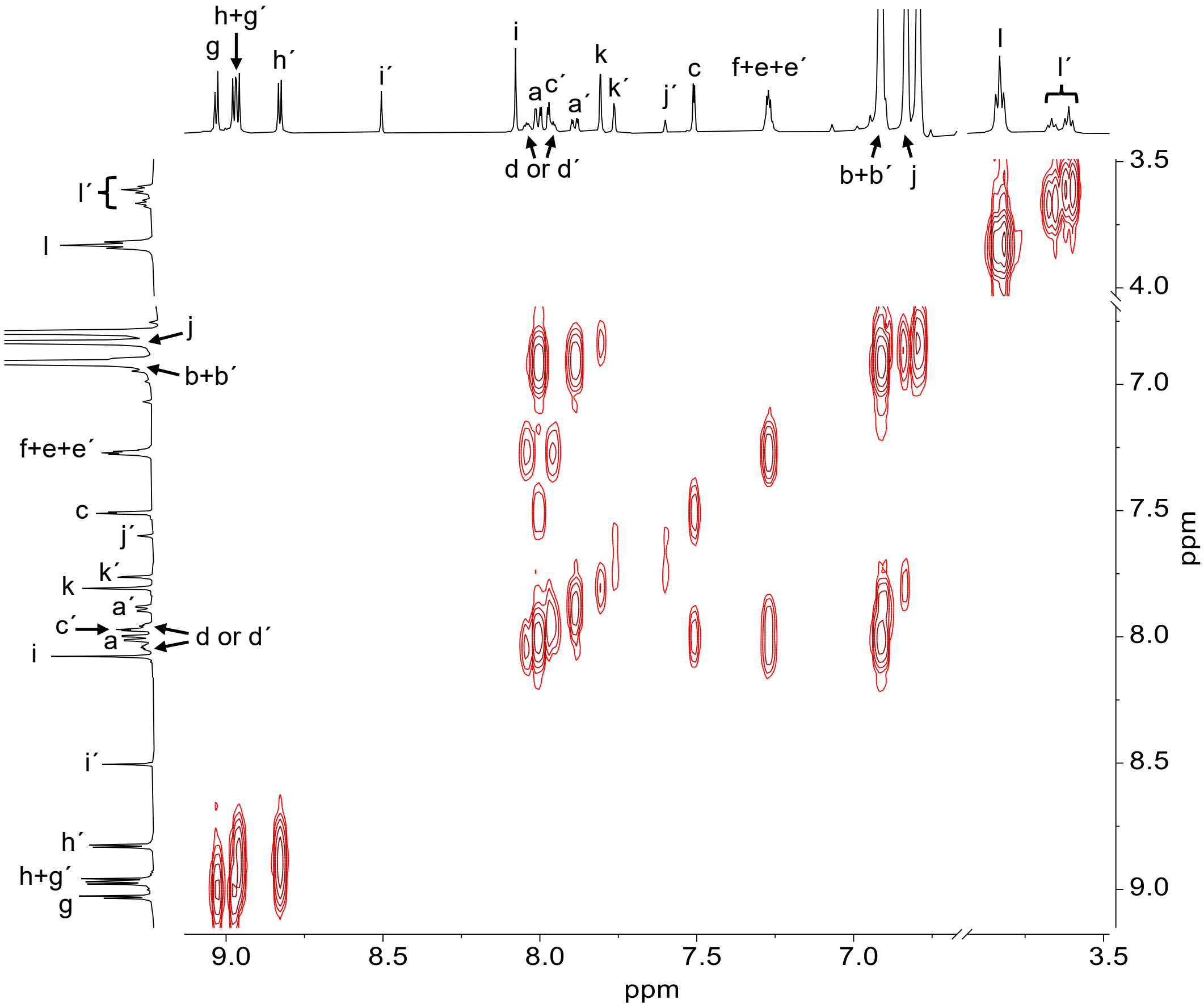

Figure S58. ^{1}H-^{1}H COSY NMR spectrum of the 1:1 complex **3_{Zn}·*bipy*** in toluene-d_8 at 298 K.

As for the NOESY spectrum of complex **3_{Zn}·*bipy***, the imine protons (H_i and $H_{i'}$) showed two types of cross-peaks that might indicate the coexistence of two types of relative arrangements of the imine bonds (please see our previous work)[1]: i) in an ***extended conformation*** (key cross-peaks highlighted with green dotted circles), and ii) in a ***compact conformation*** (key cross-peaks highlighted with orange dotted circles). In conclusion, both protons (H_i and $H_{i'}$) presented the two main conformations, demonstrating the flexibility of **3_{Zn}** to adapt its inner cavity to accommodate ***bipy***.

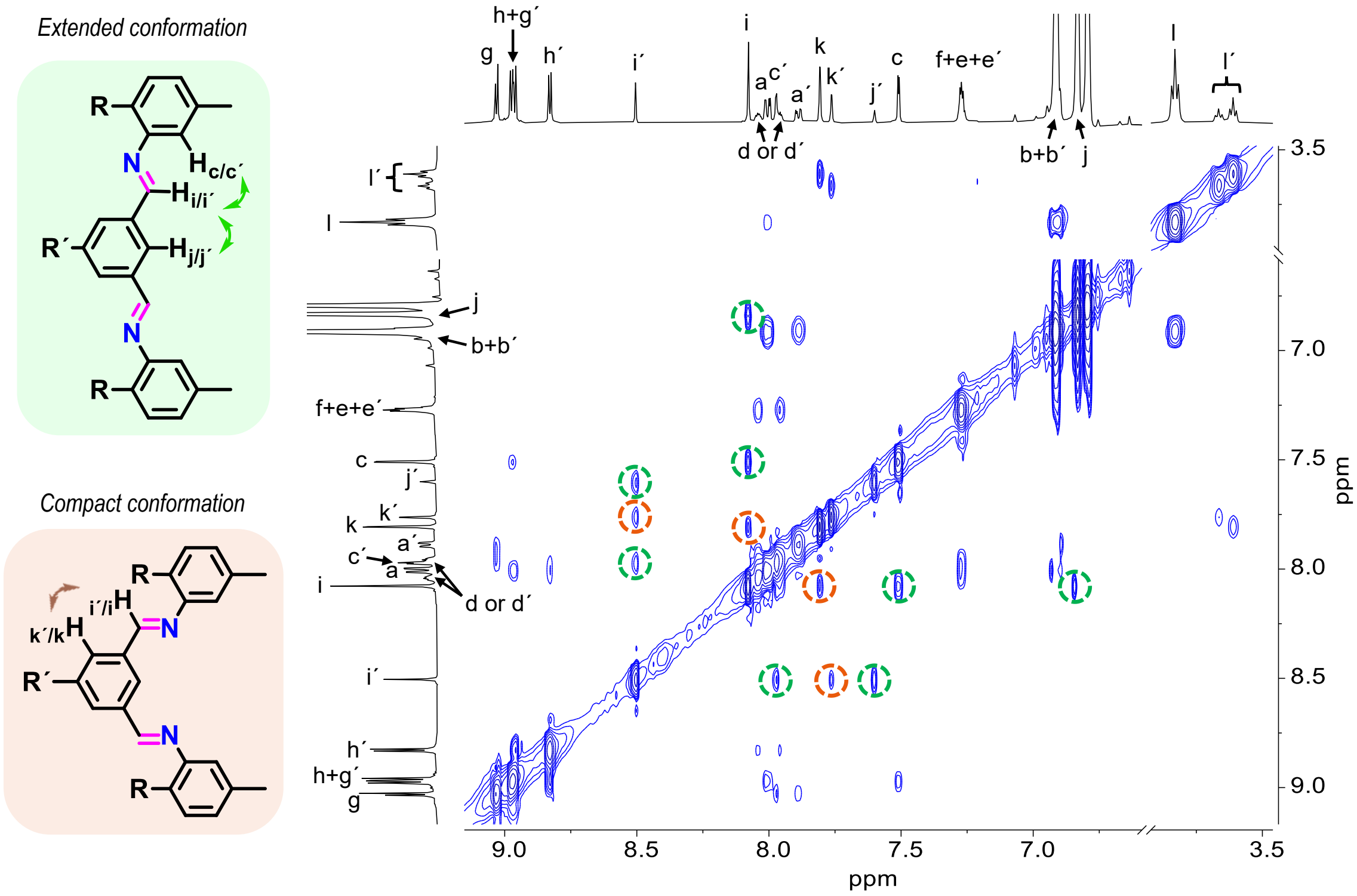

Figure S59. 2D ^{1}H-^{1}H NOESY NMR spectrum of 1:1 complex $\mathbf{3_{Zn}}$·***bipy*** in toluene-d$_8$ at 298 K.

Fluorescence Titrations

Fluorescence titration experiment demonstrated a too strong binding between ***bipy*** and $\mathbf{3_{Zn}}$, even at the lowest concentration we could test (5.0×10^{-9} M). Saturation was reached close to 1.0 equivalents and therefore the binding isotherm could not be properly fitted to a 1:1 model. Unfortunately, the use of lower concentrations, close to our emission detection limits, led to unreliable and non-reproducible results.

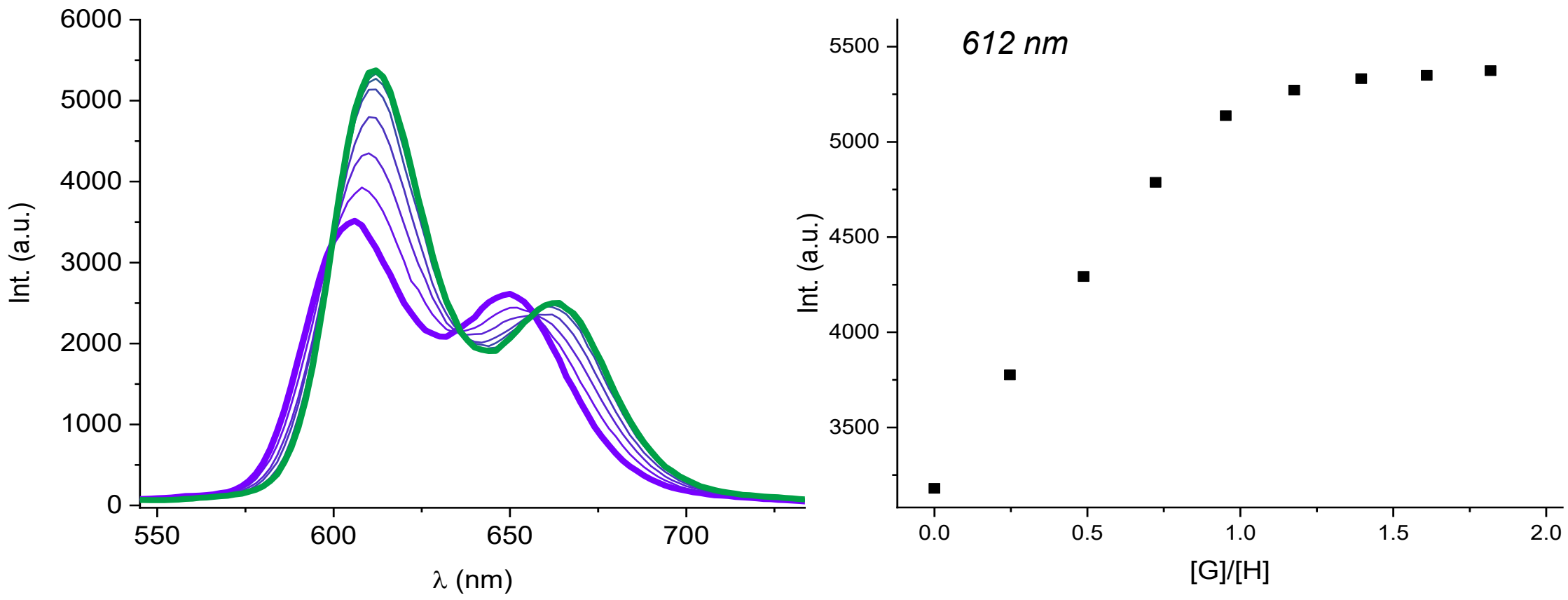

Figure S60. Fluorescence emission changes recorded along the titration of $\mathbf{3_{Zn}}$ at a constant concentration of 5.0×10^{-9} M with increasing amounts of ***bipy*** in toluene at 298 K.

Competition between *py* and *bipy* for 3$_{Zn}$.

Due to the extremely high association constant expected for **3$_{Zn}$·*bipy***, which could not be directly measured even by fluorescence spectroscopy, we turned our attention to competition experiments with pyridine-D$_5$ (***py***; see our previous work).[1] Increasing amounts of pyridine-D$_5$ (up to 100 eqs.) were added to the **3$_{Zn}$·*bipy*** complex in toluene, and the equilibrium constant established in this competition (K_C) was calculated and related to the association between **3$_{Zn}$** and ***bipy*** (K_{bipy}) according to the different equilibria and equations shown in Figure S61. The equilibrium constants K_{ref} and K_{py} were calculated by means of separate titration experiments in toluene between ***py*** and a reference tetraphenyl Zn(II)porphyrin or **3$_{Zn}$**, respectively, as $K_{ref} = 3.3 \times 10^4$ M^{-1} and $K_{py} = 1.1 \times 10^7$ M^{-2}.[1] ***K*** is the microscopic equilibrium constant of a pyridine ligand and a Zn(II) metal center.

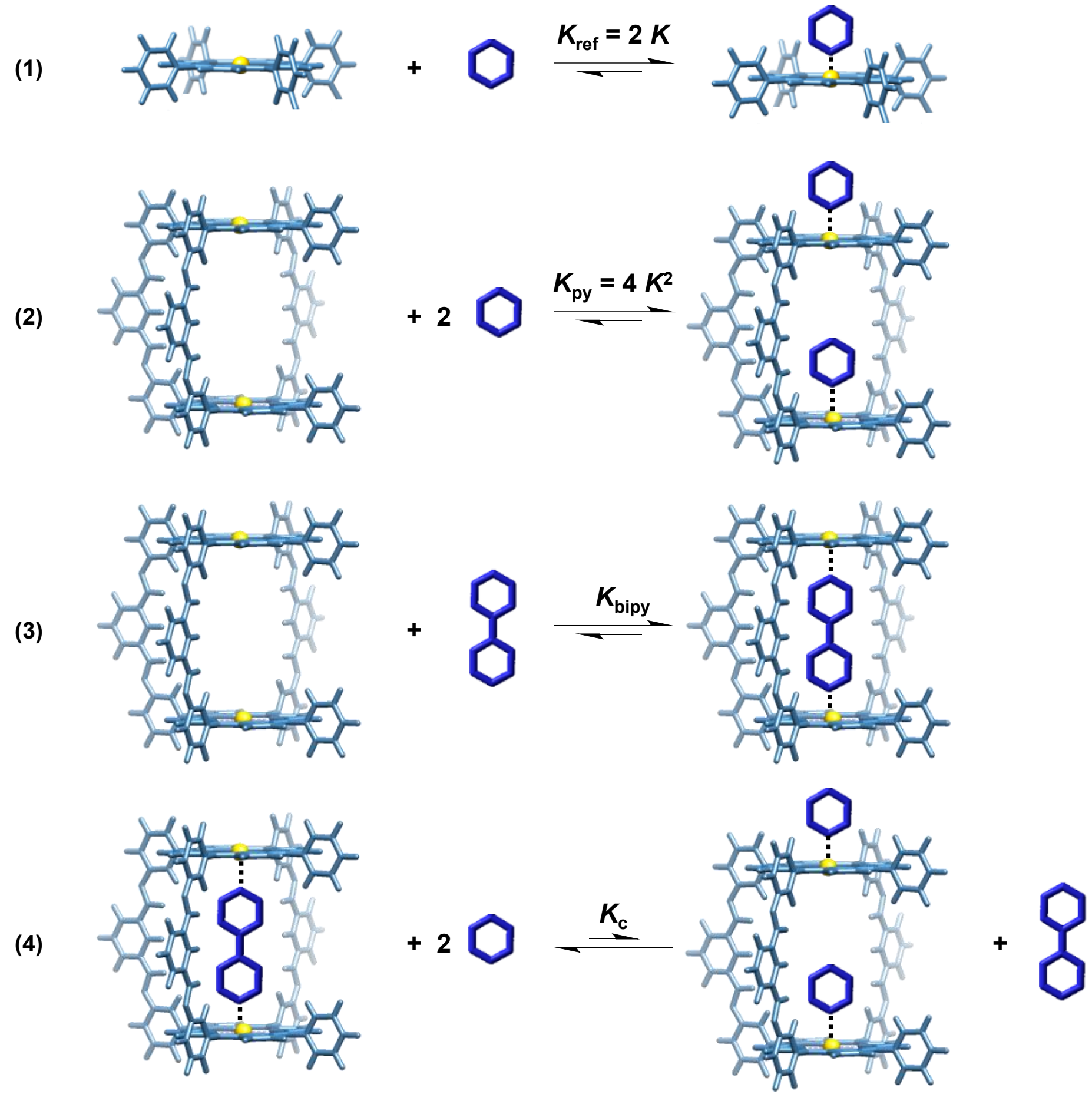

$$(4) = (2) - (3) \Rightarrow K_c = \frac{K_{py}}{K_{bipy}} = \frac{[3_{Zn}\cdot py]\cdot[bipy]}{[3_{Zn}\cdot bipy]\cdot[py]^2}$$

Figure S61. Scheme showing the determination of K_{bipy} and from a competition experiment where ***py*** can displace ***bipy*** in **3$_{Zn}$**, leading to the competition equilibrium constant K_c, which can be determined experimentally. The equilibrium constants K_{ref} and K_{py} were determined in separate experiments.

Concretely, as shown in Figure S62, to a solution of the 1:1 complex **3_{Zn}·*bipy*** (1.0×10^{-3} M), increasing amounts of the guest ***py*** (50, 60, 70, and 80 eqs.) were added in toluene-d_8 at 298K. The addition of ***py*** to **3_{Zn}·*bipy*** results in identical ^{1}H NMR spectra for both the **3_{Zn}·*bipy*** and **3_{Zn}·*py*$_2$** complexes. The most relevant difference is the progressive disappearance of the proton signals of encapsulated ***bipy***, one of them appearing at 3.26 ppm. In order to calculate the relative abundance of each complex, two main signals were monitored and integrated: the β-pyrrolic protons (purple circles) associated to both **3_{Zn}·*bipy*** and **3_{Zn}·*py*$_2$**, and the aromatic signal of bound ***bipy*** (black squares), related only to **3_{Zn}·*bipy***.

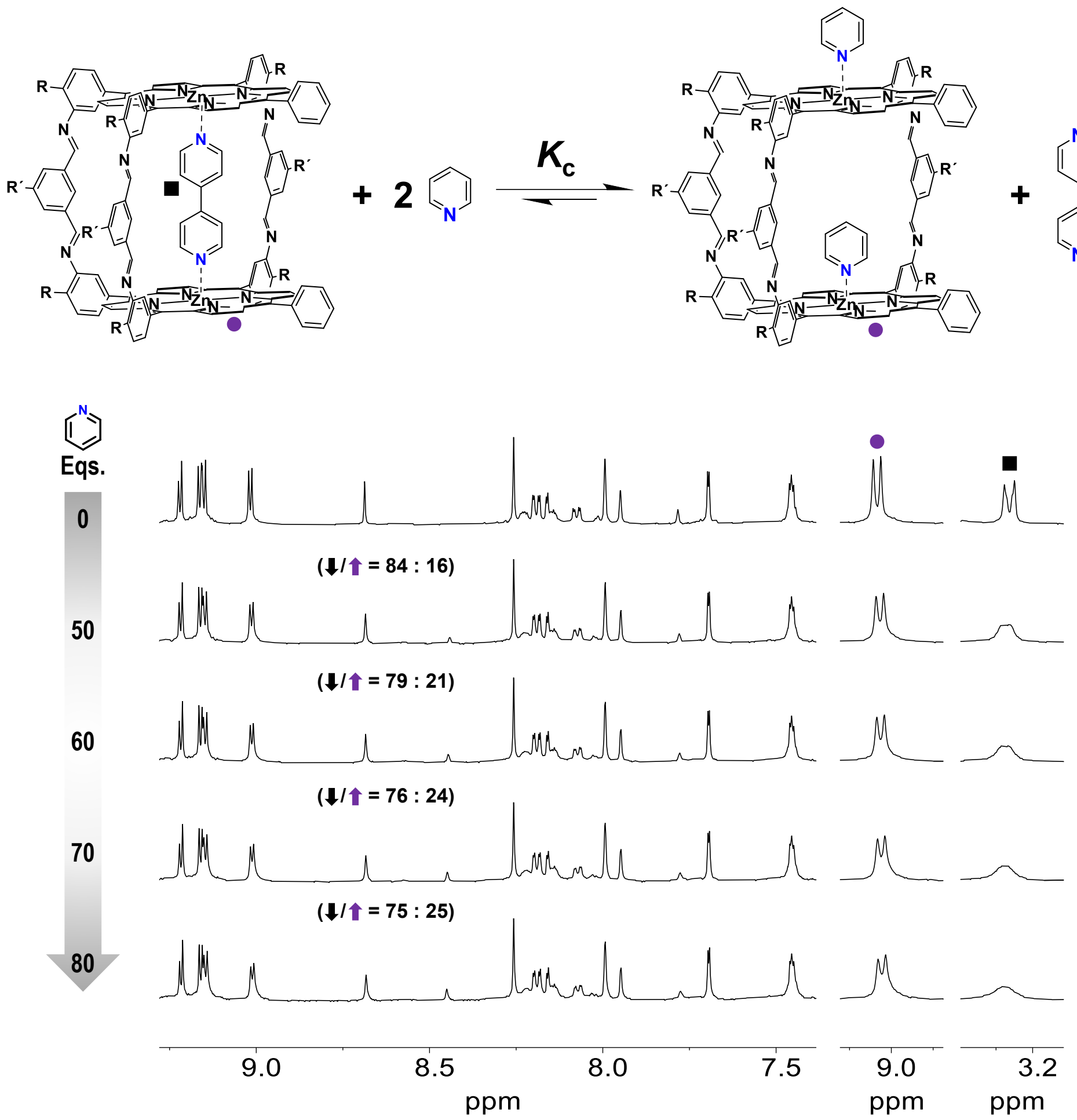

Figure S62. ^{1}H NMR spectra acquired in the competition experiment between ***bipy*** and ***py*** for the cavity of **3_{Zn}** in toluene-d_8 at 298 K.

Consequently, by ^{1}H NMR integration of the already shown NMR spectra, average values of $K_c = 0.015$ M^{-1} and, thereof, $K_{bipy} = 7.1 \times 10^8$ M^{-1} were calculated.

S13. Competition experiments between *bipy* and C_{60} to bind inside 3_{Zn}.

Different competition experiments monitored by ^{1}H NMR were performed to assess the relative binding affinity of ***bipy*** and $\mathbf{C_{60}}$ for the cavity of the $\mathbf{3_{Zn}}$ *basket*. From the average integration of several proton signals in the ^{1}H NMR spectra obtained at different ***bipy***:$\mathbf{C_{60}}$ ratios, the competition equilibrium constant (K_c) was calculated in each case. Two different approaches were used:

1) *Addition of* $\mathbf{C_{60}}$ *to the preformed* $\mathbf{3_{Zn}\cdot}$***bipy*** *complex.* To a solution of the 1:1 complex $\mathbf{3_{Zn}\cdot}$***bipy*** (1.1×10^{-4} mmol; 3.0×10^{-4} M), increasing amounts of the guest $\mathbf{C_{60}}$ (1.0, 1.5, and 2.0 eqs; 1.1, 1.7, and 2.2×10^{-4} mmol, respectively) were added in toluene-d_8 at 298K. Figure S63 shows the equilibria considered to determine K_c from the previously determined K_{bipy} and $K_{C_{60}}$, as well as the ^{1}H NMR spectra with the integrations of the two different signals selected to calculate the $\mathbf{3_{Zn}\cdot}$***bipy*** : $\mathbf{3_{Zn}\cdot C_{60}}$ ratio. In this way, the competition equilibrium constant was determined as $K_c = 0.041$.

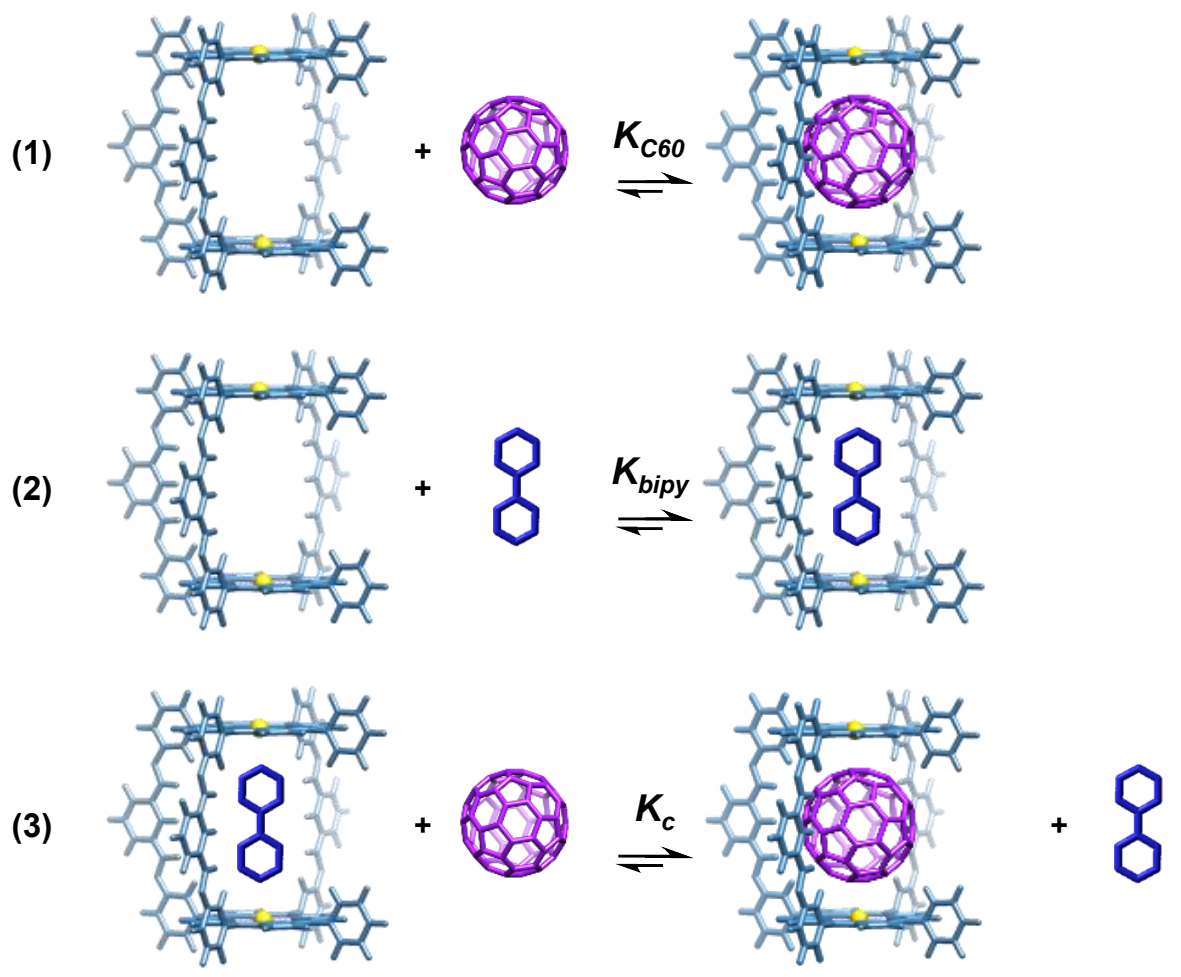

$$(3) = (1) - (2) \Rightarrow K_c = \frac{K_{C60}}{K_{bipy}}$$

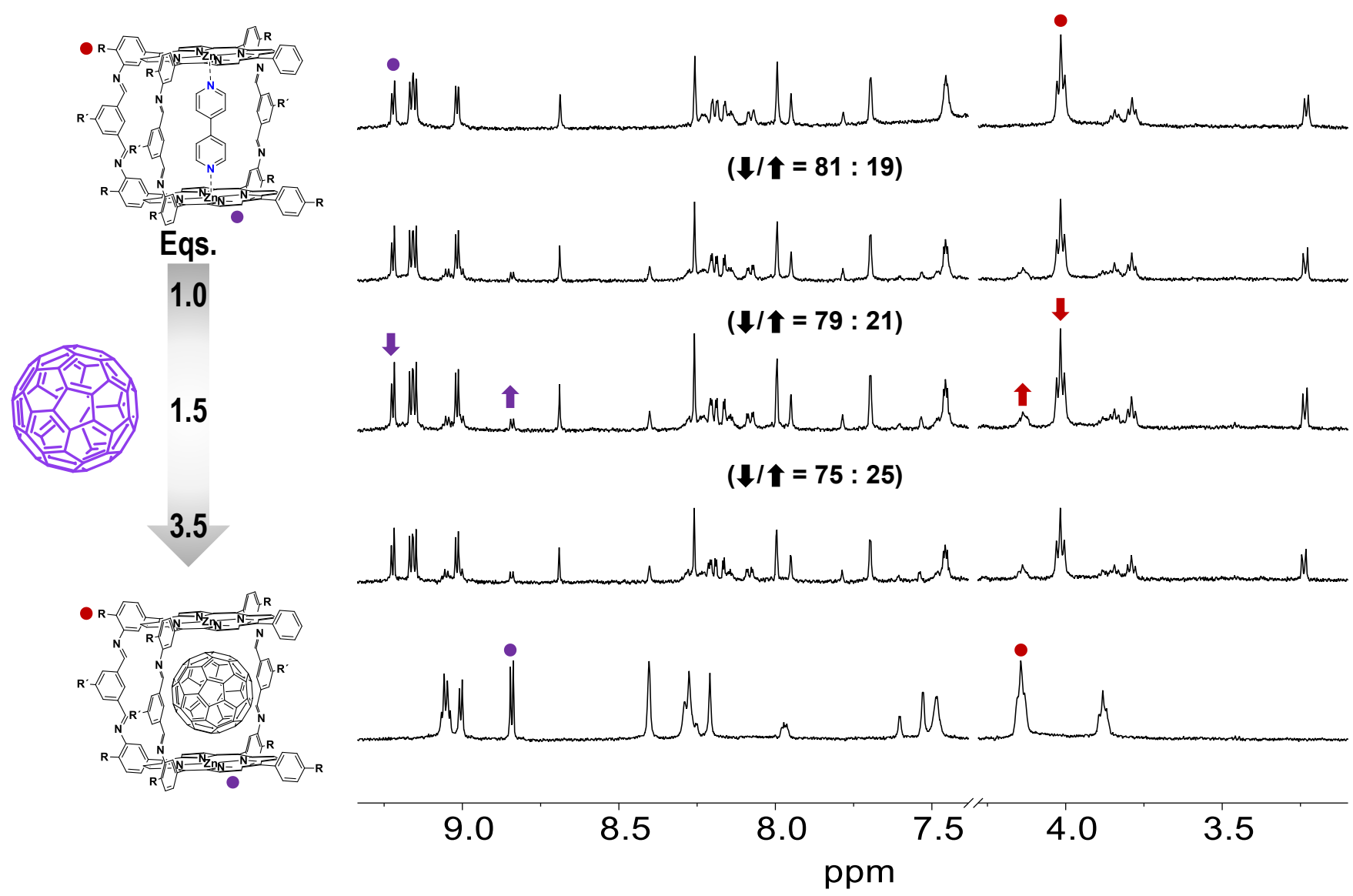

Figure S63. ^{1}H NMR spectra acquired in the competition experiment between $\mathbf{C_{60}}$ and ***bipy*** for the cavity of $\mathbf{3_{Zn}}$ in toluene-d_8 at 298 K.

2) *Addition of* ***bipy*** *to the preformed* $\mathbf{3_{Zn}{\cdot}C_{60}}$ *complex.* To a solution of the 1:1 complex $\mathbf{3_{Zn}{\cdot}C_{60}}$ (1.2×10^{-4} mmol; 3.2×10^{-4} M), increasing amounts of the guest ***bipy*** (0.50, 0.75, and 1.00 eqs; 0.6, 0.9, and 1.2×10^{-4} mmol, respectively) were added in toluene-d_8 at 298K. Figure S64 shows the equilibria considered to determine K_c from the previously determined K_{bipy} and $K_{C_{60}}$, as well as the ^{1}H NMR spectra with the integrations of the two different signals selected to calculate the $\mathbf{3_{Zn}}{\cdot}$***bipy*** : $\mathbf{3_{Zn}{\cdot}C_{60}}$ ratio. In this way, the competition equilibrium constant was determined as K_c = 23.065.

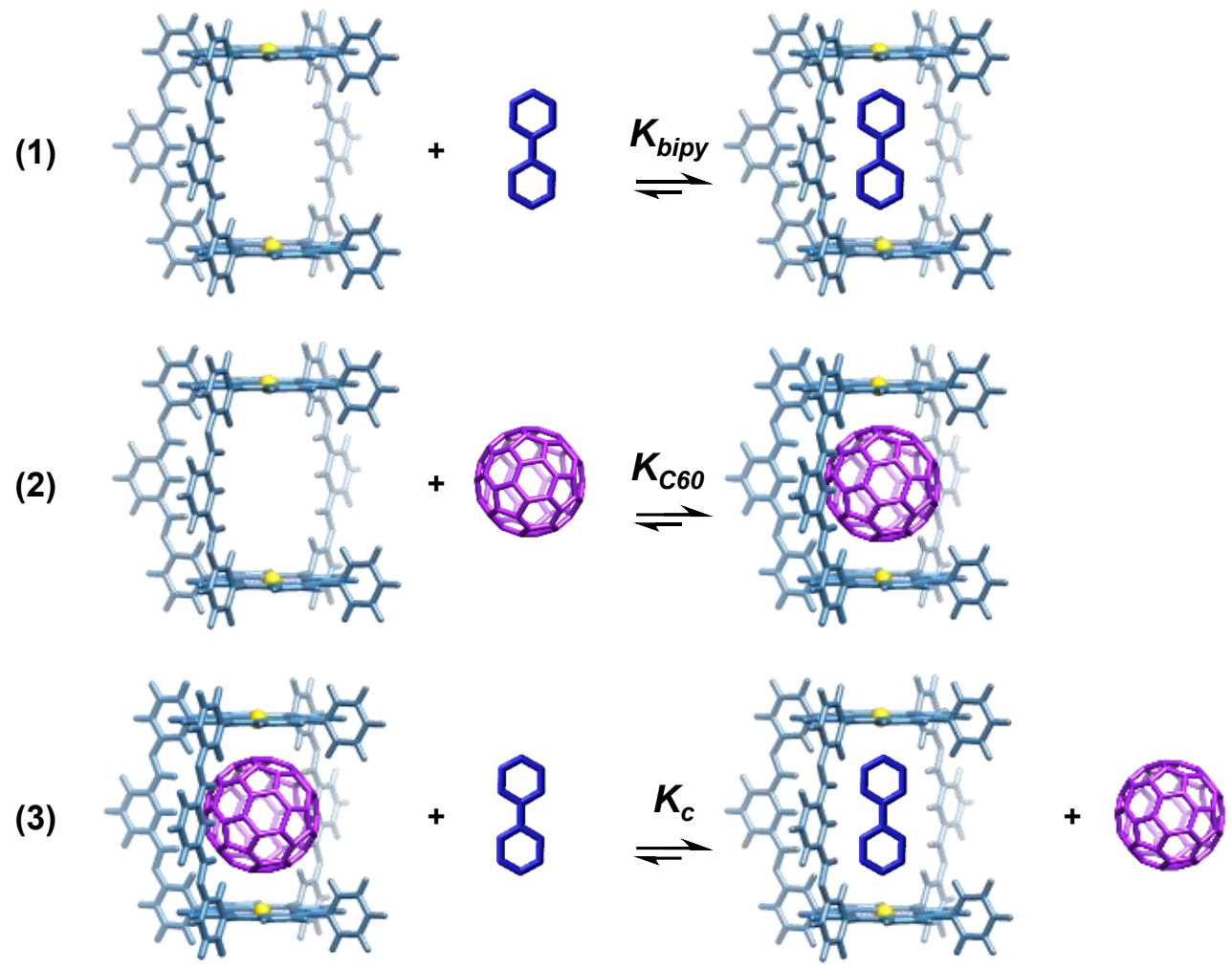

$$(3) = (1) - (2) \Rightarrow K_c = \frac{K_{bipy}}{K_{C60}}$$

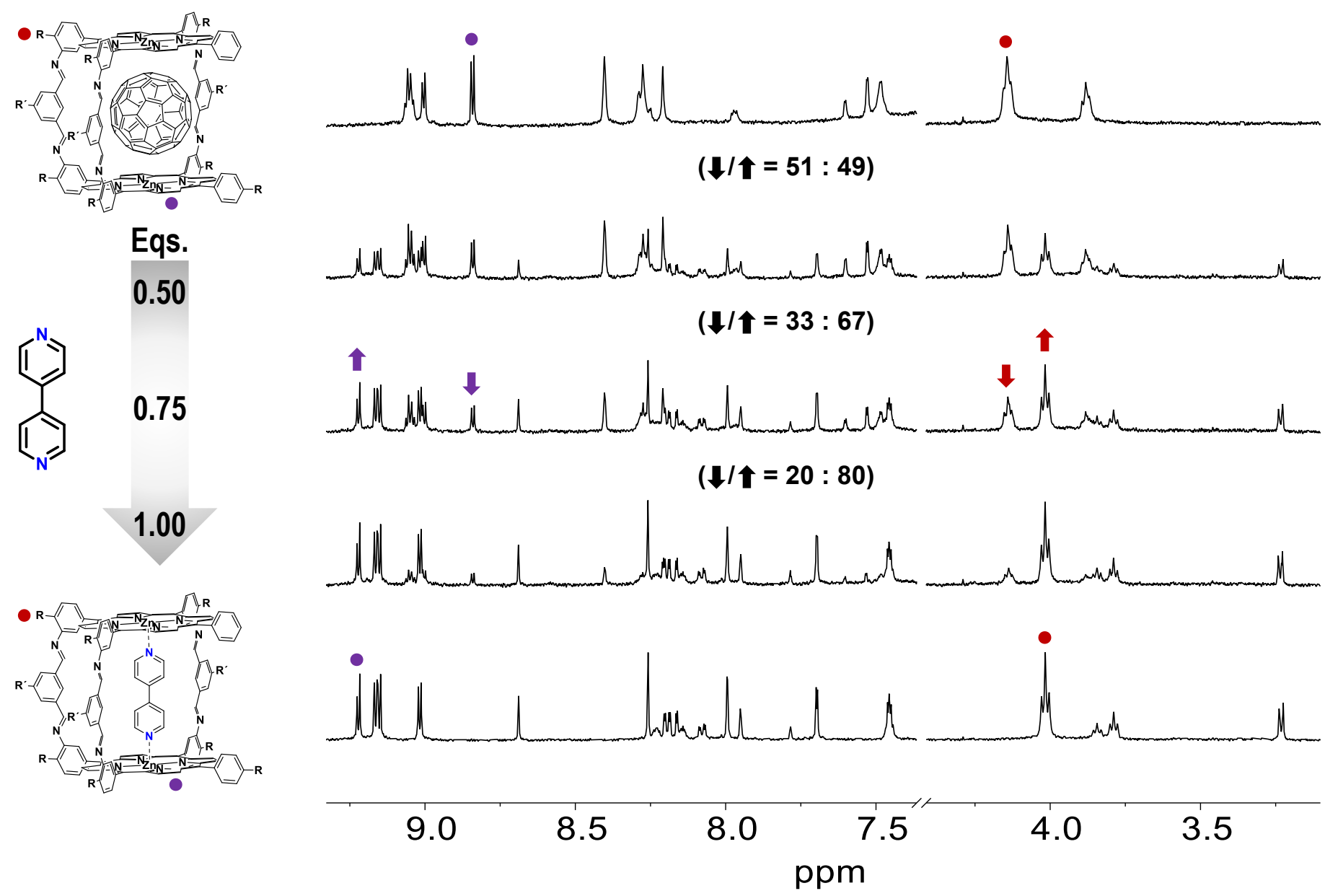

Figure S64. ^{1}H NMR spectra acquired in the competition experiment between $\mathbf{C_{60}}$ and ***bipy*** for the cavity of $\mathbf{3_{Zn}}$ in toluene-d_8 at 298 K.

The two approaches used to calculate K_c (the ratio between K_{bipy} and $K_{C_{60}}$) converged in coherent results, which indicate that $K_{bipy} \approx 20{\cdot}K_{C_{60}}$.

S14. Competition experiments between *bipy* and C_{60} to bind inside $\mathbf{1}_{Zn}$ in the presence of TFA.

The association constant of $\mathbf{C_{60}}$ to $\mathbf{1}_{Zn}$ ($K_{C_{60}}$; Figure S65) was determined by means of a competition experiment with a previously studied guest (***bipy***). The association constant of ***bipy*** within $\mathbf{1}_{Zn}$ was calculated in separate experiments as K_{bipy} = 2.1 × 10^9 M^{-1}. Then, different conditions were employed to determine the equilibrium constant of the competition established between $\mathbf{C_{60}}$ and ***bipy*** to bind with the cavity of $\mathbf{1}_{Zn}$. In order to reach the equilibrium in a reasonable timescale (*i.e.*, < 1 month), the mixtures were either heated to 413 K in *o*-xylene-D_{10} or a small amount of TFA was added to speed up the encapsulation of $\mathbf{C_{60}}$ within the cavity of $\mathbf{1}_{Zn}$. The addition of such a small amount of TFA did not have a significant influence on K_{bipy} and, thereof, on the equilibrium composition. Binding of excess ***bipy*** through Zn(II) coordination at the exterior of the cage was not considered for the indirect determination of $K_{C_{60}}$.

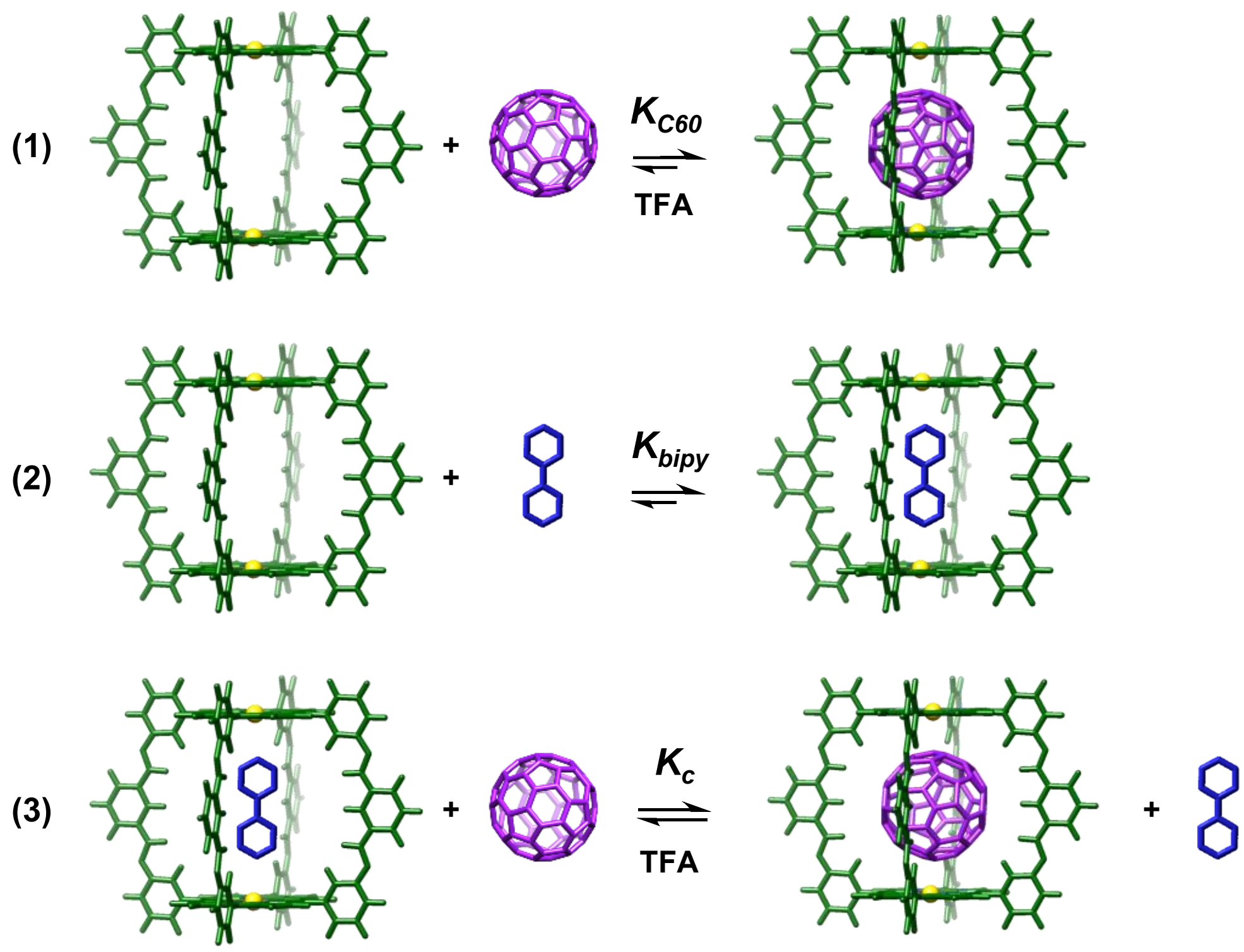

$$(3) = (1) - (2) \Rightarrow \boldsymbol{K_c} = \frac{\boldsymbol{K_{C60}}}{\boldsymbol{K_{bipy}}} = \frac{[\boldsymbol{C_{60}@1_{Zn}}]\cdot[\boldsymbol{bipy}]}{[\boldsymbol{1_{Zn}\cdot bipy}]\cdot[\boldsymbol{C_{60}}]}$$

Figure S65. Scheme showing all equilibria considered in the competition experiment between $\mathbf{C_{60}}$ and ***bipy*** to bind within the cavity of $\mathbf{1}_{Zn}$.

Experimentally, to a toluene-d_8 (Figure S66a) or *o*-xylene-d_{10} (Figure S66b) solution containing the preformed **C_{60}@1$_{Zn}$** complex (C = 5 × 10^{-4} M), one equivalent of ***bipy*** (C = 5 × 10^{-4} M) was added in a sealed NMR tube at 298 and 413 K, respectively. Interestingly, instantaneous changes were observed in the ^{1}H NMR spectrum of the **C_{60}@1$_{Zn}$** complex in both assays upon the addition of ***bipy*** due to its external coordination to the *jail*. However, to reach the equilibrium in the competition experiment extremely long times (*ca*. > 30 days), as well as high temperatures, were required, resulting in a 41:59 mixture of **1$_{Zn}$·*bipy*** and **C_{60}@1$_{Zn}$**. Note that in the experiments carried out at room temperature the supramolecular exchange is almost negligible even after *ca.* 10 days.

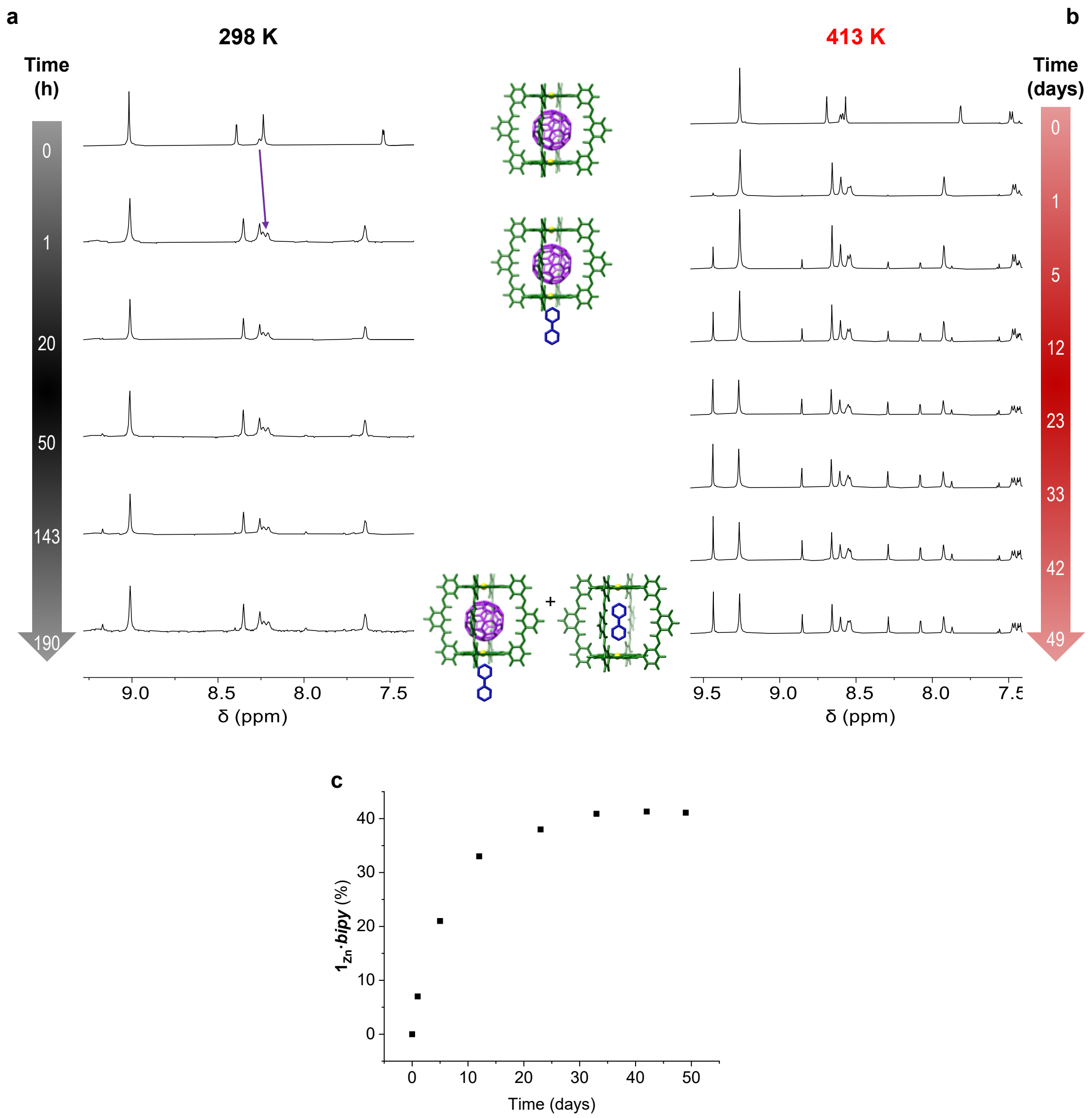

Figure S66. ^{1}H NMR spectra region showing the progressive exchange between **C_{60}** and ***bipy*** (C = 5 × 10^{-4} M) in the supramolecular complex **C_{60}@1$_{Zn}$** (C = 5 × 10^{-4} M) leading to **1$_{Zn}$·*bipy*** in toluene-d_8 (a) and *o-xylene*-d_{10} (b) at 298 and 413 K, respectively. (c) Progress of the exchange process shown in (**b**).

Alternatively, the equilibrium could be reached in less than 1 day by addition of TFA. Three experiments were conducted, starting from a mixture of fullerene **C_{60}** and the preformed complex **1_{Zn}·*bipy*** in different proportions in toluene-d_8 at 298 K. Subsequently, a small portion of TFA was added in order to enable cleavage and re-formation of imine bonds. The competition experiments were monitored via ^{1}H NMR over several hours until the equilibrium was reached, the integration of β-pyrrolic protons (blue and red circles) allowed the determination of the ratio between **1_{Zn}·*bipy*** and **C_{60}@1_{Zn}**. The association constant between ***bipy*** and **1_{Zn}** was calculated in our previous work as K_{bipy} = 2.1 × 10^9 M^{-1}.[1] We previously confirmed that the order of TFA addition did not affect the final equilibrium.

Experiment 1. To a mixture of fullerene **C_{60}** (2.0 × 10^{-4} mmol), **1_{Zn}** (2.0 × 10^{-4} mmol) and ***bipy*** (2.0 × 10^{-4} mmol) in toluene-d_8 at 298 K, 0.1 eq. of TFA (2.0 × 10^{-5} mmol) was added. The calculated value of the competition constant was K_C = 0.887, leading to $K_{C_{60}}$ = 2.4 × 10^9 M^{-1}.

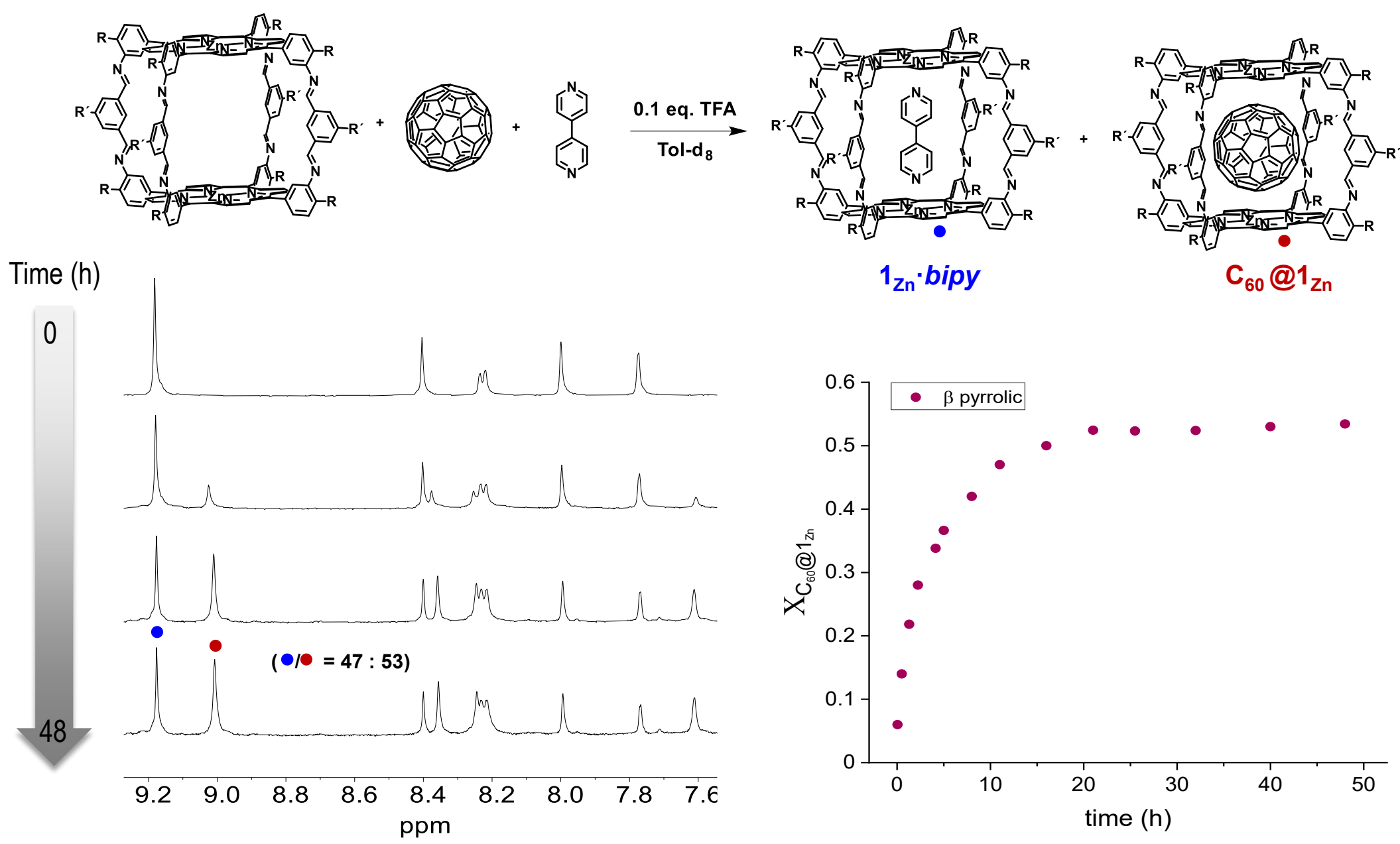

Figure S67. ^{1}H NMR spectra acquired in the competition experiment between **C_{60}** and ***bipy*** for the cavity of **1_{Zn}** in the presence of TFA at a **1_{Zn}**:***bipy***:**C_{60}** 1:1:1 ratio. The inset at the right represents the molar fraction of **C_{60}@1_{Zn}** as a function of time.

Experiment 2. To a mixture of fullerene **C_{60}** (2.0×10^{-4} mmol), **1_{Zn}** (1.0×10^{-4} mmol) and ***bipy*** (2.0×10^{-4} mmol) in toluene-d_8 at 298 K, 0.1 eq. of TFA (1.0×10^{-5} mmol) was added. The calculated value of the competition constant was K_C = 0.852, leading to $K_{C_{60}} = 2.5 \times 10^9$ M^{-1}.

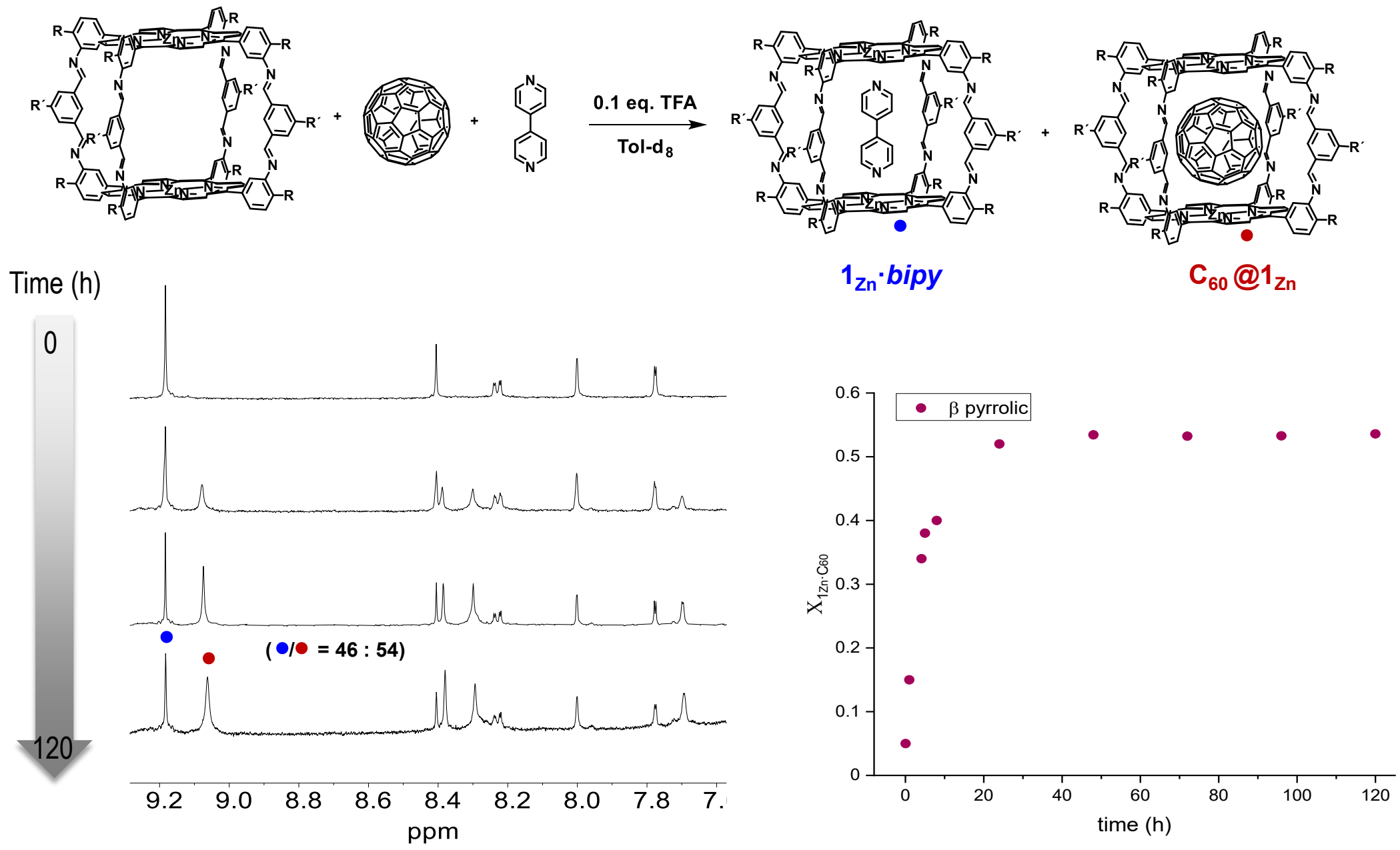

Figure S68. ^{1}H NMR spectra acquired in the competition experiment between **C_{60}** and ***bipy*** for the cavity of **1_{Zn}** in the presence of TFA at a **1_{Zn}**:***bipy***:**C_{60}** 1:2:2 ratio. The inset at the right represents the molar fraction of **C_{60}@1_{Zn}** as a function of time.

Experiment 3. To a mixture of fullerene **C_{60}** (1.0×10^{-4} mmol), **1_{Zn}** (1.0×10^{-4} mmol) and ***bipy*** (2.0×10^{-4} mmol) in toluene-d_8 at 298 K, 0.1 eq. of TFA (1.0×10^{-5} mmol) was added. The calculated value of the competition constant was $K_C = 0.658$, leading to $K_{C_{60}} = 3.3 \times 10^9$ M^{-1}.

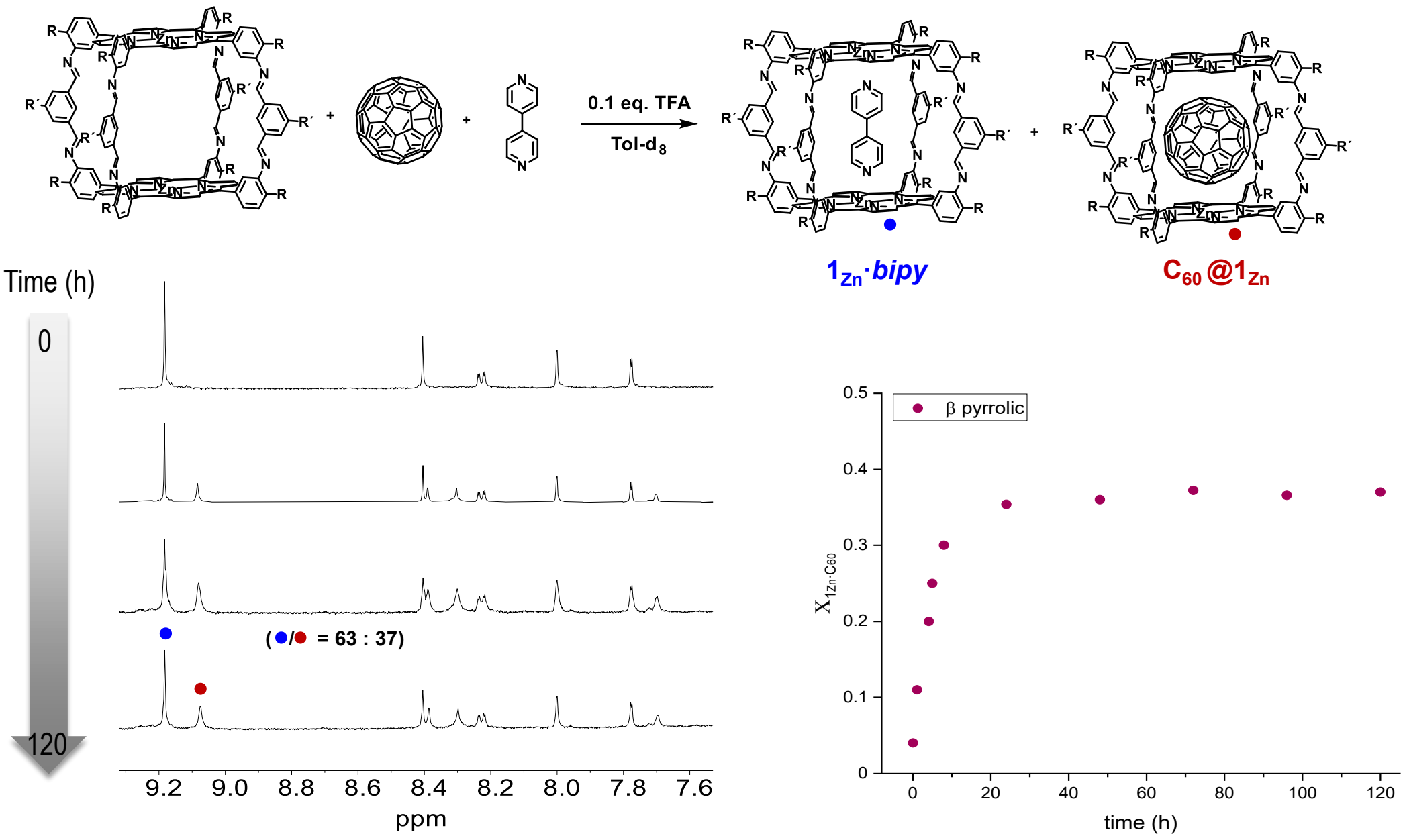

Figure S69. ^{1}H NMR spectra acquired in the competition experiment between **C_{60}** and ***bipy*** for the cavity of **1_{Zn}** in the presence of TFA at a **1_{Zn}**:***bipy***:**C_{60}** 1:2:1 ratio. The inset at the right represents the molar fraction of **C_{60}@1_{Zn}** as a function of time.

Remarkably, in the competition experiments performed at both high temperature (Figure S66) and catalyzed by acid (Figure S67), employing equimolecular amounts of **1_{Zn}**, ***bipy***, and **C_{60}** (5.0×10^{-4} M), led to very similar ratios of **1_{Zn}·*bipy*** and **C_{60}@1_{Zn}**: 59:41 and 47:53, respectively. However, ***experiments 1-3*** were chosen to calculate the association constant between **1_{Zn}** and fullerene **C_{60}** in toluene-d_8. The binding constants calculated from these three competition experiments converged in similar values: 2.4×10^9, 2.5×10^9, and 3.3×10^9 M^{-1}. Thus, an average value of $K_{C_{60}} = 2.7 \pm 0.5 \times 10^9$ M^{-1}, where the error is the standard deviation, was considered.

References

1- A. P. Gia, A. de Juan, D. Aranda, F. G. Guijarro, J. Aragó, E. Ortí, M. García-Iglesias, D. González-Rodríguez, *J. Am. Chem. Soc.* **2025**, *147*, 918–931.

2- V. Církva, P. Jakubík, T. Strašák, J. Hrbáč, J. Sýkora, I. Císařová, J. Vacek, J. Žádný, J. Storch, *J. Org. Chem.* **2019**, *84*, 1980–1993.

3- A. D. Becke, *J. Chem. Phys.* **1993**, *98*, 5648–5652.

4- S. Grimme, J. Antony, S. Ehrlich, H. Krieg, *J. Chem. Phys.* **2010**, *132*, 154104.

5- S. Grimme, S. Ehrlich, L. Goerigk, *J. Comp. Chem.* **2011**, *32*, 1456–1465.

6- T. H. Dunning Jr., *J. Chem. Phys.* **1989**, *90*, 1007–1023.

7- M. J. Frisch, G. W. Trucks, H. B. Schlegel, G. E. Scuseria, M. A. Robb, J. R. Cheeseman, et al. Gaussian 16, Revision A. 03. Wallingford, CT: Gaussian In, 2016.

8- V. Martí-Centelles, T. K. Piskorz, F. Duarte, *J. Chem. Inf. Model.* **2024**, *64*, 5604–5616

9- O. V. Dolomanov, L. J. Bourhis, R. J. Gildea, J. A. K. Howard, H. Puschmann, *J. Appl. Crystallogr.* **2009**, *42*, 339–341.

10- E. V. Anslyn, D. A. Dougherty, Modern Physical Organic Chemistry; University Science Books: Sausalito, CA, 2006; Chapter 7.

11- M. L. Tiberti, A. Barducci, M. Parrinello, *J. Phys. Chem. B*. **2010**, *114*, 15309–15315.

12- M. Bonomi, A. Barducci, M. Parrinello, *J. Comp. Chem.* **2009**, *30*,1615–1621.

13- M. J. Abraham, T. Murtola, R. Schulz, S. Páll, J. C. Smith, B. Hess, E. Lindahl, *SoftwareX*. **2015**, *1–2*, 19–25.

14- G. A. Tribello, M. Bonomi, D. Branduardi, C. Camilloni, G. Bussi, *Comput. Phys. Commun.* **2014**, *185*, 604–613.

15- M. Bonomi; D. Branduardi, G. Bussi, C. Camilloni, D. Provasi, P. Raiteri, D. Donadio, F. Marinelli, F. Pietrucci, R. A. Broglia, et al., *Comput. Phys. Commun.* **2009**, *180*, 1961–1972.

16- S. Giannini, P. M. Martinez, A. Semmeq, J. P. Galvez, A. Piras, A. Landi, D. Padula, J. G. Vilhena, J. Cerezo, G. Prampolini, *J. Chem. Theor. Comput.* **2025**, *21*, 3156–3175.

17- U. C. Signh, P. A. Kollman, *J. Comp. Chem.* **1984**, *5*, 129–145.

18- B. H. Besler, K. M. Merz, P. A. Kollman, *J. Comp. Chem.* **1990**, *11*, 431–439.

19- W.L. Jorgensen, D.S. Maxwell, J. Tirado-Rives, *J. Am. Chem. Soc.* **1996**, *118*, 11225–11236.
